# Enantioselective semireduction of allenes

**DOI:** 10.1038/s41467-017-00793-0

**Published:** 2017-10-04

**Authors:** Zhiwei Chen, Vy M. Dong

**Affiliations:** Department of Chemistry, University of California, Irvine, 4403 Natural Sciences 1, Irvine, CA 92697 USA

## Abstract

Rh-hydride catalysis solves a synthetic challenge by affording the enantioselective reduction of allenes, thereby yielding access to motifs commonly used in medicinal chemistry. A designer Josiphos ligand promotes the generation of chiral benzylic isomers, when combined with a Hantzsch ester as the reductant. This semireduction proceeds chemoselectively in the presence of other functional groups, which are typically reduced using conventional hydrogenations. Isotopic labelling studies support a mechanism where the hydride is delivered to the branched position of a Rh-allyl intermediate.

## Abstract (web summary)

Reduction of allenes poses several challenges in terms of chemo-, regio- and enantio-selectivity. Here, the authors report a rhodium-Josiphos catalytic system that reduces a variety of aryl allenes to chiral benzylic compounds with excellent selectivity and functional group tolerance.

## Introduction

**Fig. 1:**
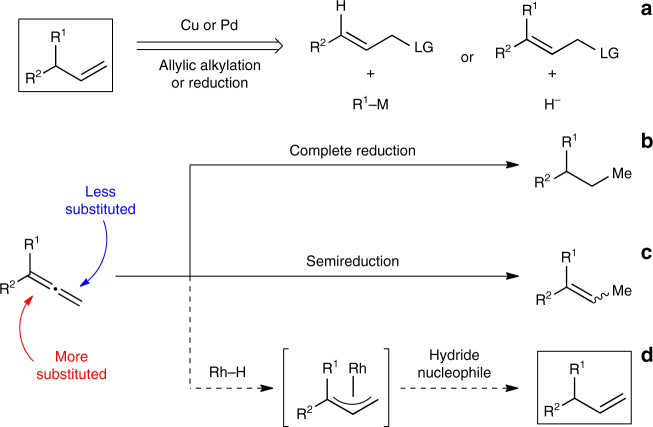
Challenges in the selective reduction of allenes. **a** Traditional methods to access chiral allylic motifs. **b** Complete reduction affords alkanes. **c** Existing allene semireductions favour formation of the internal alkene. **d** Proposed strategy for regio- and enantioselective semireduction to afford the complementary terminal alkene

In nature, chemo- and stereocontrolled reduction of unsaturated bonds are catalysed by enzymes and mediated by cofactors such as nicotinamide adenine dinucleotide phosphate (NAD(P)H)^1^. Inspired by this cofactor, chemists have used Hantzsch esters as mild reagents to solve various challenges in asymmetric reductions^2^. It occurred to us that this cofactor mimic could be combined with Rh-hydride catalysis to enable a valuable strategy for reducing allenes to generate benzylic motifs, which are traditionally made by an allylic substitution between an allylic electrophile and an organometallic reagent^3–7^ or a hydride source^8–14^ (Fig. 1a). As allenes are readily accessible^15^, a method to access these motifs through a semireduction of allenes would avoid the pre-installation of a suitable leaving group. Allenes are challenging functional groups for reduction because of problems with chemo-, regio-, and stereoselectivity. Both *π*-bonds can be reduced to the corresponding alkane (Fig. 1b), or one *π*-bond can be reduced to afford one or a mixture of alkene isomers (Fig. 1c). Before studies in the regioselective semireduction of allenes have shown that the less substituted *π*-bond is typically reduced to afford the achiral internal alkene^16–18^. Existing methods that reduce the more substituted *π*-bond are limited to monosubstituted and symmetrical allenes, which give rise to achiral terminal alkenes^19, 20^.

The generation of electrophilic metal-allyl species from allenes using iridium- and rhodium-hydrides is an emerging strategy in allene hydrofunctionalisation^21, 22^. These intermediates can undergo allylic substitution with various nucleophiles to afford branched allylated products. We envisioned that a Rh-hydride catalyst would transform an allene to an electrophilic Rh-allyl intermediate, which can then be trapped with a hydride nucleophile^23–26^. Given that allenes are known to isomerise to dienes in the presence of transition metal-hydrides^27^, we recognise that a key challenge would be identifying a catalyst that promotes semireduction over isomerisation.

Herein, we demonstrate an asymmetric semireduction of allenes enabled by Rh-hydride catalysis as a complementary approach to allylic alkylation and allylic reduction to generate chiral benzylic motifs. Using a designed Josiphos ligand and a Hantzsch ester reductant, various allenes are reduced to the corresponding chiral terminal alkenes with high selectivities.

## Results

### Reaction development

**Fig. 2:**
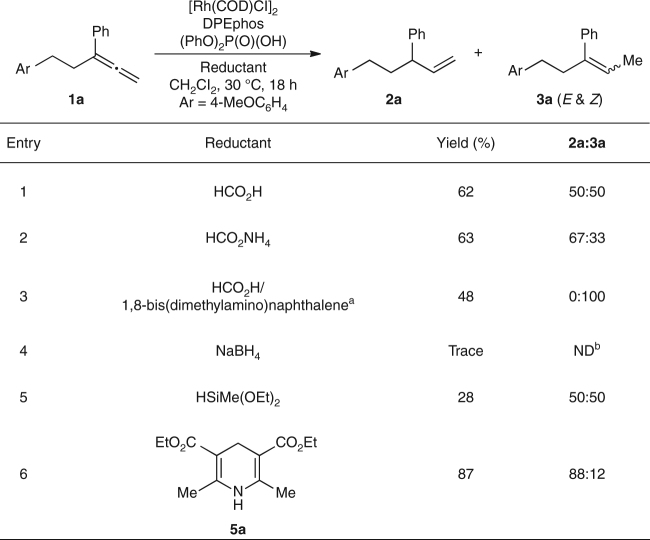
Evaluation of reductants. Reaction conditions: **1a** (0.050 mmol), reductant (0.10 mmol), [Rh(COD)Cl]_2_ (4 mol%), DPEphos (8 mol%), (PhO)_2_P(O)(OH) (8 mol%), CH_2_Cl_2_ (0.1 mL), 30 °C, 18 h. Yields and regioselectivities were determined by ^1^H NMR analysis of the unpurified reaction mixture using dimethyl terephthalate as an internal standard. ^a^HCO_2_H (0.11 mmol), 1,8-bis(dimethylamino)naphthalene (0.060 mmol). ^b^ND, not determined

To test our hypothesis (Fig. 1d), we chose 1-methoxy-4-(3-phenylpenta-3,4-dien-1-yl)benzene (**1a**) as the model substrate for semireduction in the presence of [Rh(COD)Cl]_2_, (PhO)_2_P(O)(OH), and DPEphos (Fig. 2). Through a survey of achiral bidentate phosphine ligands, we found DPEphos to be the most promising scaffold for suppressing diene formation, in the presence of various reductants. Tsuji and Mandai^8^ demonstrated that formic acid and formates are competent reductants in the reduction of allylic carbonates. However, these reagents led to semireductions with little to no regiocontrol (50:50 to 67:33 **2a**:**3a**, Fig. 2, entries 1 and 2). Hayashi and Kawabata showed that a combination of formic acid and an amine base, such as 1,8-bis(dimethylamino)naphthalene, reduced allylic carbonates and esters^9–12^. In our system, this combination suppressed semireduction of the more substituted *π*-bond (Fig. 2, entry 3). NaBH_4_, a classical nucleophilic hydride source, gave trace reactivity (Fig. 2, entry 4), and a silanes^13, 14^ afforded unselective semireduction in low conversion (28%, 50:50 **2a**:**3a**, Fig. 2, entry 5). When Hantzsch ester **5a** was used as the reductant (Fig. 2, entry 6), the reactivity increased (87% yield), and the desired terminal alkene was obtained as the major product (88:12 **2a**:**3a**).

**Fig. 3:**
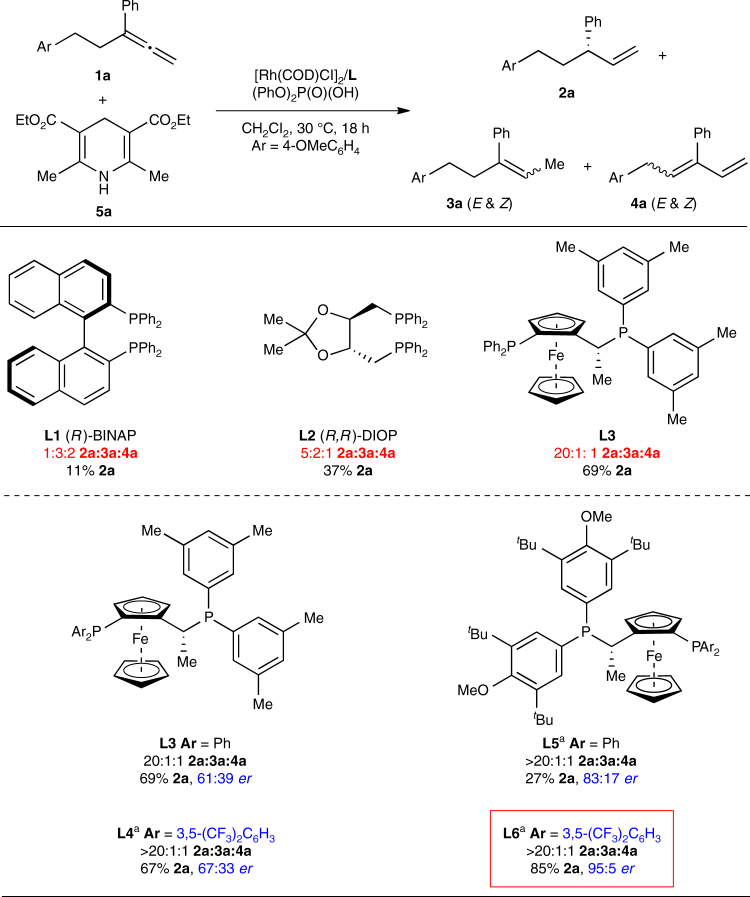
Evaluation of chiral ligands. Reaction conditions: **1a** (0.10 mmol), **5a** (0.20 mmol), [Rh(COD)Cl]_2_ (4 mol%), L (8 mol%), (PhO)_2_P(O)(OH) (8 mol%), CH_2_Cl_2_ (0.2 mL), 30 °C, 18 h. Yields and product ratios were determined by ^1^H NMR analysis of the unpurified reaction mixture using dimethyl terephthalate as an internal standard. Enantioselectivities (*er*’s) were determined by chiral SFC analysis. ^a^Using [Rh(COD)Cl]_2_ (2 mol%), L (4 mol%), (PhO)_2_P(O)(OH) (4 mol%), CH_2_Cl_2_ (0.1 mL)

Next, we searched for a chiral ligand that could enable high enantio- and regioselectivities, in combination with Hantzsch ester **5a** as the reductant (Fig. 3). Axially chiral bisphosphine ligands, such as (*R*)-BINAP (**L1**), afforded a mixture of alkenes **2a** and **3a**, as well as competitive isomerisation to diene **4a** (1:3:2 **2a**:**3a**:**4a**). Ligands bearing point chirality, such as (*R*,*R*)-DIOP (**L2**), promoted semireduction over isomerisation, but with moderate regioselectivity (5:2 **2a**:**3a**). We discovered that the all-aryl substituted Josiphos ligand scaffold gave high selectivity for **2a**. A significant increase in the reaction selectivity (20:1:1 **2a**:**3a**:**4a**) was observed when commercially available ligand **L3** was employed. Josiphos ligand **L4**, where one phosphine is more electron-deficient, afforded an increase in the reaction rate, so the catalyst loading can be reduced two-fold. In addition, **L4** further improved selectivity for **2a** (>20:1:1 **2a**:**3a**:**4a**), but the enantioselectivity remained low (67:33 *er*). To improve the enantioselectivity, we replaced the 3,5-xylyl groups of **L3** and **L4** with the more electron-rich and sterically encumbered 3,5-di-*tert*-butyl-4-methoxyphenyl groups to afford new Josiphos ligands **L5** and **L6**. With **L5**, the enantioselectivity increased (83:17 *er*), but low reactivity (27%) was observed. However, **L6** afforded the desired terminal alkene in 85% yield and 95:5 *er* while maintaining the high selectivity for **2a** (>20:1:1 **2a**:**3a**:**4a**).

### Reaction scope

**Fig. 4:**
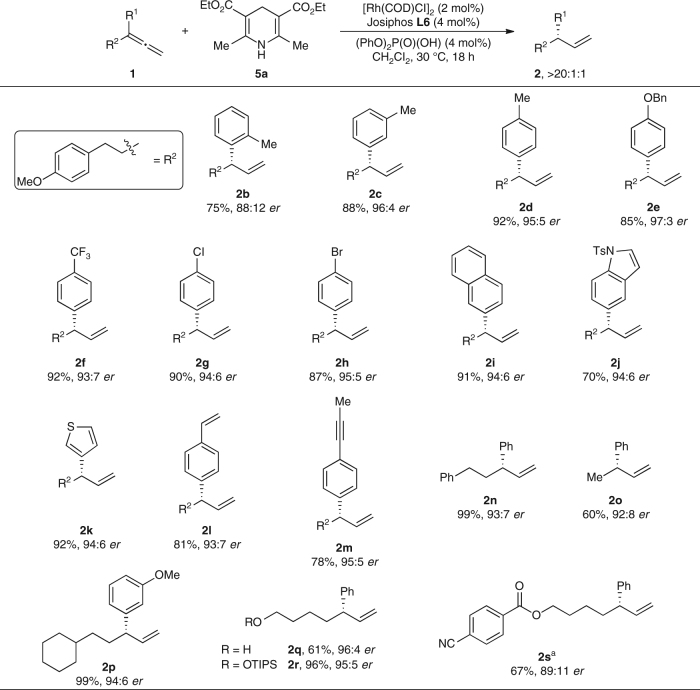
Enantioselective semireduction of allenes. Reaction conditions: **1** (0.20 mmol), **5a** (0.40 mmol), [Rh(COD)Cl]_2_ (2 mol%), **L6** (4 mol%), (PhO)_2_P(O)(OH) (4 mol%), CH_2_Cl_2_ (0.2 mL), 30 °C, 18 h. Isolated yields. Product ratios were determined by ^1^H NMR analysis of the unpurified reaction mixture. Enantioselectivities (*er’*s) were determined by chiral SFC analysis. ^a^Reaction performed with 1,2-dichloroethane at 60 °C

With this protocol, we examined the generality of enantioselective semireduction using other allenes (Fig. 4). Generally, the terminal alkene was obtained as the sole product; no internal alkene or diene was observed. An allene with an *ortho* substituent (**2b**) on the phenyl group underwent semireduction with lower enantioselectivity (75%, 88:12 *er*). Substrates with *meta* (**2c**) and *para* (**2d**) substituents on the phenyl group reacted with similar efficiencies as the model substrate (88%, 96:4 *er* and 92%, 95:5 *er*, respectively). Allenes bearing electron-rich (**2e**) and electron-deficient (**2f**) substituents underwent semireduction (85–92%, 93:7–97:3 *er*). A benzyl ether is labile under typical hydrogenation conditions, but this protecting group was stable under our semireduction conditions (**2e**). Substrates bearing aryl halide bonds (**2g** and **2h**) were tolerated (87–90%, 94:6–95:5 *er*). Extended aromatic systems, such as a naphthyl group (**2i**), reacted (91%, 94:6 *er*). The semireduction tolerates allenes with heteroaromatic moieties, such as an *N*-tosyl indole (**2j**, 70%, 94:6 *er*) and a thiophene (**2k**, 82%, 94:6 *er*). Chemoselective reduction occurred with substrates containing alkenes (**2l**), alkynes (**2m**), esters (**2s**) and nitriles (**2s**), affording the terminal alkenes selectively (67–81%, 89:11–95:5 *er*). 1-Aryl-1-propynes (**2m**) are reactive substrates towards isomerisation and hydrofunctionalisation^22^, but only the allene functionality reacted. Allenes bearing other alkyl groups were accommodated (**2n**–**2s**, 60–99%, 89:11–96:4 *er*). At last, the semireduction occurs chemoselectively in the presence of other nucleophiles, such as an alcohol (**2q**, 61%, 96:4 *er*). Allenes bearing dialkyl or diaryl substituents were unreactive under the present conditions. Notably, the semireduction tolerates acidic and electrophilic functionalities, such as an alcohol (**2q**), ester (**2s**), and nitrile (**2s**). Thus, this method to access benzylic motifs complements allylic substitutions using organometallic reagents. The absolute configuration of **2n** was determined to be (*S*) by comparison of its optical rotation with literature data^28^.

### Mechanistic studies

**Fig. 5:**
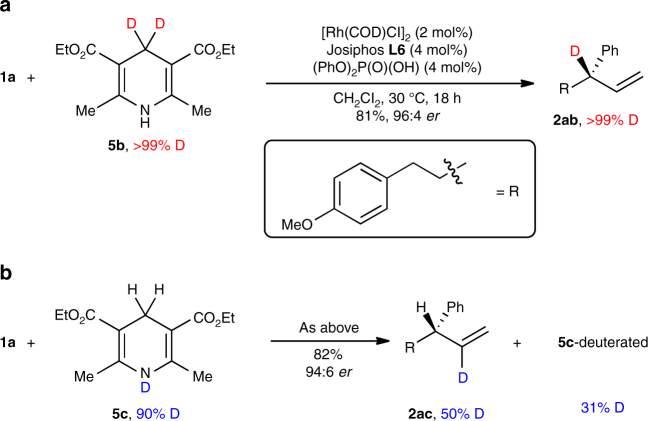
Deuterium-labelling studies. **a** Treatment of allene **1a** with deuterated Hantzsch ester **5b** afforded deuteration in the allylic position. **b** Analogous experiment with **5c** gave deuteration in the vinylic position

To shed light on the mechanism of this semireduction, we performed deuterium-labelling experiments using deuterated analogues of Hantzsch ester **5a**. Semireduction of **1a** with **5b** afforded **2ab**, where the deuterium label was completely transferred to the allylic carbon (Fig. 5a). In addition to its mechanistic significance, this experiment demonstrates a method to prepare chiral isotopically labelled stereogenic centres that complements allylic deuteration using formic acid-*d*_2_^29, 30^. Using **5c**, **2ac** was obtained, where the deuterium label was incorporated into the internal vinylic carbon (Fig. 5b). The remaining deuteriums were incorporated into the vinylic methyl groups of the Hantzsch ester **5c** (31% D) and the pyridine byproduct (6% D) presumably as a statistical mixture of products^31^.

**Fig. 6:**
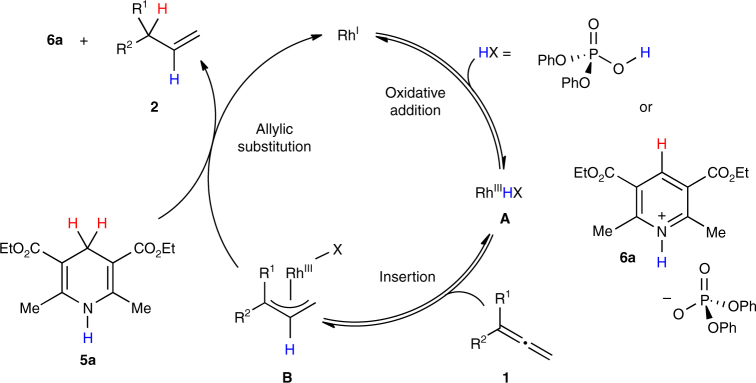
Proposed mechanism. Mechanistic pathway of the allene semireduction

On the basis of our observations and literature precedence, we propose the mechanism shown in Fig. 6. To initiate catalysis, the Rh(I) precursor undergoes oxidative addition to generate a Rh(III)-hydride species **A**. The insertion of allene **1** with **A** forms an electrophilic Rh(III)-allyl intermediate **B**, which undergoes allylic substitution with Hantzsch ester **5a** to furnish the terminal alkene **2** and regenerate the catalyst.

## Discussion

As a complementary approach to allylic alkylation and allylic reduction, we have demonstrated a Rh-catalysed regio- and enantioselective semireduction of allenes as a strategy to generate chiral benzylic motifs. The high reaction selectivities are enabled by a designed Josiphos ligand and a Hantzsch ester reductant. Given the significance of deuterated pharmaceuticals^32–34^, new strategies for asymmetric hydride delivery are especially relevant. Our approach allows access to isotopically labelled stereogenic centres and occurs with excellent chemo- and stereocontrol in the presence of functional groups that are sensitive to conventional hydrogenations.

## Methods

### General procedure for the semireduction of allenes

In a N_2_-filled glovebox, [Rh(COD)Cl]_2_ (2.0 mg, 0.0040 mmol, 2 mol%), (PhO)_2_P(O)(OH) (2.0 mg, 0.0080 mmol, 4 mol%), Josiphos **L6** (9.1 mg, 0.0080 mmol, 4 mol%), Hantzsch ester **5a** (101.3 mg, 0.40 mmol, 2.0 equiv), allene **1** (0.20 mmol, 1 equiv), and anhydrous CH_2_Cl_2_ (0.20 mL, 1 M) were added to a 1 dram vial equipped with a magnetic stir bar. The vial was then sealed with a Teflon-lined screw cap and stirred at 30 °C for 18 h. The reaction mixture was cooled to rt and concentrated in vacuo. Regioselectivities were determined by ^1^H NMR analysis of the unpurified reaction mixture. The terminal alkene product **2** was purified by preparatory thin-layer chromatography. The enantioselectivities were determined by chiral SFC analysis. All new products were fully characterised. For the full experimental procedures and characterisation, including NMR spectra, of the new compounds in this article, see Supplementary Figs. 1–167.

### Data availability

The authors declare that the data supporting the conclusions of this study are available within the article and its Supplementary Information file or from the authors upon reasonable request.

## Electronic supplementary material

Supplementary Information
