# Supplementary material for: Enantioselective semireduction of allenes
Source: Nat Commun. 2017 Oct 4;8:784. doi: 10.1038/s41467-017-00793-0 (PMC5627242; doi:10.1038/s41467-017-00793-0)
Supplement: Supplementary file 1 — Supplementary Information [file 41467_2017_793_MOESM1_ESM.pdf]

Supplementary Methods

General Information

Commercially reagents were purchased from Sigma Aldrich, Strem, Acros Organics, TCI or Alfa Aesar and used without further purification. All experiments were performed in oven-dried or flame-dried glassware under an atmosphere of N_2. Tetrahydrofuran, diethyl ether, toluene, and dichloromethane were purified using an Innovative Technologies Pure Solv system, degassed by three freeze-pump-thaw cycles, and stored over 3A MS within a N_2 filled glove box. Reactions were monitored either *via* gas chromatography using an Agilent Technologies 7890A GC system equipped with an Agilent Technologies 5975C inert XL EI/CI MSD or by analytical thin-layer chromatography on EMD Silica Gel 60 F_{254} plates. Visualization of the developed plates was performed under UV light (254 nm) or using $KMnO_4$ stain. Purification and isolation of products were performed via silica gel chromatography (both column and preparative thin-layer chromatography). Column chromatography was performed with Silicycle Silia-P Flash Silica Gel using glass columns. ^{1}H, ^{13}C, ^{19}F, and ^{31}P NMR spectra were recorded on a Bruker DRX-400 (400 MHz ^{1}H, 100 MHz ^{13}C, 376.5 MHz ^{19}F, 162 MHz ^{31}P), GN-500 (500 MHz ^{1}H, 125.7 MHz ^{13}C, 202 MHz ^{31}P), or CRYO-500 (500 MHz ^{1}H, 125.7 MHz ^{13}C) spectrometer. ^{1}H NMR spectra were internally referenced to the residual solvent signal or TMS. ^{13}C NMR spectra were internally referenced to the residual solvent signal. Data for ^{1}H NMR are reported as follows: chemical shift (δ ppm, δ 7.27 for $CDCl_3$), multiplicity (s = singlet, d = doublet, t = triplet, q = quartet, m = multiplet, br = broad), coupling constant (Hz), integration. Data for ^{13}C NMR are reported in terms of chemical shift (δ ppm, δ 77.16 for $CDCl_3$). Infrared spectra were obtained on a Thermo Scientific Nicolet iS5 FT-IR spectrometer equipped with an iD5 ATR accessory, and were reported in terms of frequency of absorption (cm^{-1}). Enantioselectivities were determined by chiral SFC analysis using an Agilent Technologies HPLC (1200 series) system and Aurora A5 Fusion. High-resolution mass spectra (HRMS) were obtained on a micromass 70S-250 spectrometer (EI) or an ABI/Sciex QStar Mass Spectrometer (ESI), performed by the University of California, Irvine Mass Spectrometry Centre. Allenes **1o**, **1q**, and **1r** are known compounds and were prepared according to literature procedures[1,2]. Deuterated Hantzsch esters **5b** and **5c** were prepared according to literature procedures[3,4].

General Procedure for the Semireduction of Allenes

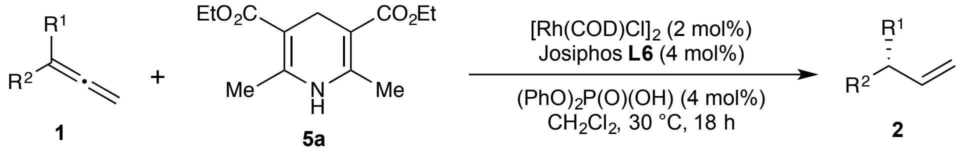

In a N_2-filled glovebox, $[Rh(COD)Cl]_2$ (2.0 mg, 0.0040 mmol, 2 mol%), $(PhO)_2P(O)(OH)$ (2.0 mg, 0.0080 mmol, 4 mol%), Josiphos **L6** (9.1 mg, 0.0080 mmol, 4 mol%), Hantzsch ester **5a** (101.3 mg, 0.40 mmol, 2.0 equiv), allene **1** (0.20 mmol, 1 equiv), and CH_2Cl_2 (0.20 mL, 1 M) were added to a 1 dram vial equipped with a magnetic stir bar. The vial was then sealed with a Teflon-lined screw cap and stirred at 30 °C for 18 h. The reaction mixture was cooled to rt and concentrated *in vacuo*. Regioselectivities were determined by ^{1}H NMR analysis of the unpurified reaction mixture.

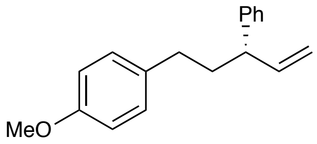

(*S*)-1-methoxy-4-(3-phenylpent-4-en-1-yl)benzene (2a): The title compound was synthesized according to the general procedure and isolated by preparatory TLC (5% EtOAc in hexanes) as a colorless oil (21.4 mg (from 0.10 mmol of starting material), 85% yield, >20:1 *rr*, 95:5 *er*, $[\alpha]^{24}_D$ = +9.1 (*c* 1.3, $CHCl_3$)). **^{1}H NMR** (400 MHz, $CDCl_3$) δ 7.37 – 7.29 (m, 2H), 7.25 – 7.17 (m, 3H), 7.08 (d, *J* = 8.8 Hz, 2H), 6.84 (d, *J* = 8.7 Hz, 2H), 6.05 – 5.92 (m, 1H), 5.10 – 5.01 (m, 2H), 3.80 (s, 3H), 3.29 (q, *J* = 7.6 Hz, 1H), 2.62 – 2.44 (m, 2H), 2.02 (ddd, *J* = 8.7, 8.2, 4.5 Hz, 2H). **^{13}C NMR** (126 MHz, $CDCl_3$) δ 157.8, 144.3, 142.3, 134.4, 129.5, 128.6, 127.8, 126.4, 114.4, 113.9, 55.4, 49.3, 37.3, 32.8. **IR** (ATR): 3027, 2933, 1611, 1511, 1452, 1243, 1176, 1035, 913, 826 cm^{-1}. **HRMS** calculated for $C_{18}H_{20}O$ $[M]^+$ 252.1514, found 252.1514. **Chiral SFC**: 100 mm CHIRALCEL OJ-H, 10% *i*-PrOH, 3.0 mL/min, 220 nm, 44 °C, nozzle pressure = 200 bar CO_2, t_{R1} (minor) = 2.1 min, t_{R2} (major) = 2.5 min.

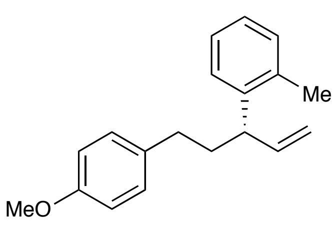

(*S*)-1-(5-(4-methoxyphenyl)pent-1-en-3-yl)-2-methylbenzene (2b): The title compound was synthesized according to the general procedure and isolated by preparatory TLC (5% EtOAc in hexanes) as a colorless oil (40.0 mg, 75% yield, >20:1 *rr*, 88:12 *er*, $[\alpha]^{24}_D$ = +23.8 (*c* 0.87, $CHCl_3$)). **^{1}H NMR** (400 MHz, $CDCl_3$) δ 7.25 – 7.18 (m, 2H), 7.18 – 7.10 (m, 2H), 7.07 (d, *J* = 8.6 Hz, 2H), 6.84 (d, *J* = 8.6 Hz, 2H), 5.93 (ddd, *J* = 17.4, 10.2, 7.4 Hz, 1H), 5.03 (ddt, *J* = 25.9, 17.1, 1.5 Hz, 2H), 3.81 (s, 3H), 3.53 (q, *J* = 7.4 Hz, 1H),

2.66 – 2.48 (m, 2H), 2.26 (s, 3H), 2.04 (dtd, *J* = 8.9, 7.1, 3.5 Hz, 2H). **^{13}C NMR** (101 MHz, $CDCl_3$) δ 157.9, 142.1, 141.8, 136.1, 134.4, 130.5, 129.5, 126.5, 126.3, 126.1, 114.4, 113.9, 55.4, 44.3, 36.9, 32.9, 19.7. **IR** (ATR): 2933, 2833, 1611, 1511, 1441, 1300, 1244, 1176, 1036, 913, 827, 752 cm^{-1}. **HRMS** calculated for $C_{19}H_{22}O$ $[M]^+$ 266.1671, found 266.1670. **Chiral SFC**: 100 mm CHIRALCEL OJ-H, 1% *i*-PrOH, 2.0 mL/min, 220 nm, 44 °C, nozzle pressure = 200 bar CO_2, t_{R1} (minor) = 9.3 min, t_{R2} (major) = 10.8 min.

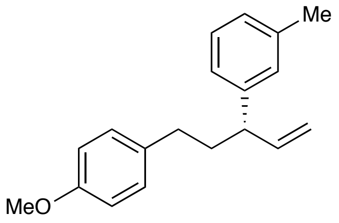

(*S*)-1-(5-(4-methoxyphenyl)pent-1-en-3-yl)-3-methylbenzene (2c): The title compound was synthesized according to the general procedure and isolated by preparatory TLC (5% EtOAc in hexanes) as a colorless oil (46.6 mg, 88% yield, >20:1 *rr*, 96:4 *er*, $[\alpha]^{24}_D$ = +11.5 (*c* 0.95, $CHCl_3$)). **^{1}H NMR** (400 MHz, CD_2Cl_2) δ 7.19 (t, *J* = 7.8 Hz, 1H), 7.07 (d, *J* = 8.5 Hz, 2H), 7.01 (dd, *J* = 11.1, 5.0 Hz, 3H), 6.81 (d, *J* = 8.6 Hz, 2H), 5.98 (ddd, *J* = 16.9, 10.5, 7.9 Hz, 1H), 5.10 – 4.99 (m, 2H), 3.76 (s, 3H), 3.23 (q, *J* = 7.5 Hz, 1H), 2.60 – 2.41 (m, 2H), 2.33 (s, 3H), 1.98 (td, *J* = 8.7, 1.5 Hz, 2H). **^{13}C NMR** (101 MHz, CD_2Cl_2) δ 158.6, 145.1, 143.3, 138.9, 135.2, 130.1, 129.2, 129.1, 127.7, 125.4, 114.7, 114.4, 56.0, 50.2, 38.2, 33.6, 22.0. **IR** (ATR): 2931, 2858, 1610, 1511, 1455, 1300, 1244, 1176, 1037, 912, 821, 785, 703 cm^{-1}. **HRMS** calculated for $C_{19}H_{22}ONH_4$ $[M+NH_4]^+$ 284.2014, found 284.2005. **Chiral SFC**: 100 mm CHIRALCEL OJ-H, 1% *i*-PrOH, 2.0 mL/min, 220 nm, 44 °C, nozzle pressure = 200 bar CO_2, t_{R1} (major) = 8.4 min, t_{R2} (minor) = 9.0 min.

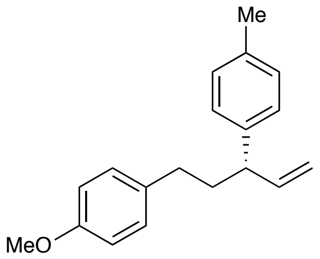

(*S*)-1-methoxy-4-(3-(*p*-tolyl)pent-4-en-1-yl)benzene (2d): The title compound was synthesized according to the general procedure and isolated by preparatory TLC (5% EtOAc in hexanes) as a colorless oil (49.0 mg, 92% yield, >20:1 *rr*, 95:5 *er*, $[\alpha]^{24}_D$ = +10.2 (*c* 0.65, $CHCl_3$)). **^{1}H NMR** (400 MHz, CD_2Cl_2) δ 7.17 – 7.02 (m, 6H), 6.81 (d, *J* = 8.7 Hz, 2H), 6.03 – 5.90 (m, 1H), 5.07 – 4.99 (m, 2H), 3.76 (s, 3H), 3.24 (q, *J* = 7.5 Hz, 1H), 2.61 – 2.40 (m, 2H), 2.32 (s, 3H), 2.05 – 1.89 (m, 2H). **^{13}C NMR** (101 MHz, CD_2Cl_2) δ 158.6, 143.4, 142.1, 136.6, 135.2, 130.1, 129.9, 128.3, 114.5, 114.4, 56.0, 49.8, 38.2, 33.6, 21.5. **IR** (ATR): 2921, 2857, 1611, 1511, 1455, 1300, 1243, 1176, 1036, 912, 815 cm^{-1}. **HRMS** calculated for $C_{19}H_{22}O$ $[M]^+$ 266.1671, found 266.1664. **Chiral SFC**: 100 mm CHIRALCEL OJ-H, 20% *i*-PrOH, 1.0 mL/min, 220 nm, 44 °C, nozzle pressure = 200 bar CO_2, t_{R1} (minor) = 3.8 min, t_{R2} (major) = 4.0 min

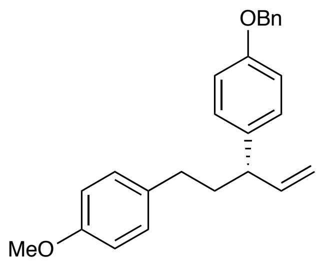

(*S*)-1-(benzyloxy)-4-(5-(4-methoxyphenyl)pent-1-en-3-yl)benzene (2e): The title compound was synthesized according to the general procedure and isolated by preparatory TLC (5% EtOAc in hexanes) as a colorless oil (61.1 mg, 85% yield, >20:1 *rr*, 97:3 *er*, $[\alpha]^{24}_D$ = +8.8 (*c* 0.83, $CHCl_3$)). **^{1}H NMR** (400 MHz, $CDCl_3$) δ 7.50 – 7.46 (m, 2H), 7.43 (ddt, *J* = 9.5, 7.9, 1.6 Hz, 2H), 7.39 – 7.33 (m, 1H), 7.16 (d, *J* = 8.5 Hz, 2H), 7.11 (d, *J* = 8.7 Hz, 2H), 6.98 (d, *J* = 8.8 Hz, 2H), 6.86 (d, *J* = 8.7 Hz, 2H), 5.99 (ddd, *J* = 16.4, 10.9, 7.5 Hz, 1H), 5.11 – 5.02 (m, 4H), 3.82 (s, 3H), 3.27 (q, *J* = 7.4 Hz, 1H), 2.63 – 2.47 (m, 2H), 2.09 – 1.96 (m, 2H). **^{13}C NMR** (101 MHz, $CDCl_3$) δ 157.8, 157.4, 142.6, 137.3, 136.6, 134.4, 129.4, 128.70, 128.68, 128.0, 127.6, 115.0, 114.1, 113.9, 70.2, 55.4, 48.4, 37.4, 32.8. **IR** (ATR): 3031, 2932, 1609, 1509, 1453, 1300, 1241, 1175, 1035, 912, 826, 734 cm^{-1}. **HRMS** calculated for $C_{19}H_{22}O$ $[M]^+$ 358.1933, found 358.1942. **Chiral SFC**: 100 mm CHIRALCEL OJ-H, 10% *i*-PrOH, 2.0 mL/min, 220 nm, 44 °C, nozzle pressure = 200 bar CO_2, t_{R1} (major) = 16.1 min, t_{R2} (minor) = 17.7 min.

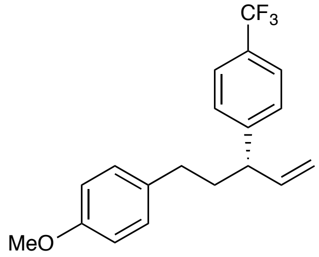

(*S*)-1-methoxy-4-(3-(4-(trifluoromethyl)phenyl)pent-4-en-1-yl)benzene (2f): The title compound was synthesized according to the general procedure and isolated by preparatory TLC (5% EtOAc in hexanes) as a colorless oil (59.1 mg, 92% yield, >20:1 *rr*, 93:7 *er*, $[\alpha]^{24}_D$ = +7.8 (*c* 1.0, $CHCl_3$)). **^{1}H NMR** (400 MHz, $CDCl_3$) δ 7.60 (d, *J* = 8.0 Hz, 2H), 7.36 – 7.30 (m, 2H), 7.09 (d, *J* = 8.7 Hz, 2H), 6.86 (d, *J* = 8.7 Hz, 2H), 5.98 (ddd, *J* = 17.1, 10.3, 7.5 Hz, 1H), 5.11 (ddt, *J* = 19.7, 17.1, 1.4 Hz, 2H), 3.82 (s, 3H), 3.37 (q, *J* = 7.4 Hz, 1H), 2.63 – 2.46 (m, 2H), 2.15 – 1.95 (m, 2H). **^{13}C NMR** (101 MHz, $CDCl_3$) δ 158.0, 148.4 (q, *J* = 1.3 Hz), 141.3, 133.9, 129.4, 128.7 (q, *J* = 32.4 Hz), 128.2, 125.6 (q, *J* = 3.8 Hz), 124.6 (q, *J* = 271.8 Hz), 115.3, 114.0, 55.4, 49.1, 37.1, 32.7. **^{19}F NMR** (376 MHz, $CDCl_3$) δ -62.3. **IR** (ATR): 2936, 1615, 1511, 1324, 1301, 1245, 1162, 1118, 1067, 1036, 1017, 918, 827 cm^{-1}. **HRMS** calculated for $C_{19}H_{19}F_3O$ $[M]^+$ 320.1388, found 320.1398. **Chiral SFC**: 100 mm CHIRALCEL OJ-H, 1% *i*-PrOH, 1.5 mL/min, 220 nm, 44 °C, nozzle pressure = 200 bar CO_2, t_{R1} (minor) = 4.7 min, t_{R2} (major) = 5.1 min.

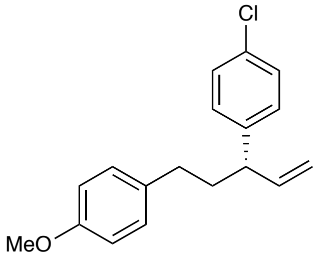

(*S*)-1-chloro-4-(5-(4-methoxyphenyl)pent-1-en-3-yl)benzene (2g): The title compound was synthesized according to the general procedure and isolated by preparatory TLC (5% EtOAc in hexanes) as a colorless oil (51.2 mg, 90% yield, >20:1 *rr*, 94:6 *er*, $[\alpha]^{24}_D$ = +8.1 (*c* 0.81, $CHCl_3$)). **^{1}H NMR** (400 MHz, $CDCl_3$) δ 7.31 (d, *J* = 8.4 Hz, 2H), 7.15 (d, *J* = 8.4 Hz, 2H), 7.08 (d, *J* = 8.6 Hz, 2H), 6.85 (d, *J* = 8.6 Hz, 2H), 6.01 – 5.87 (m, 1H), 5.12 – 5.01 (m, 2H), 3.81 (s, 3H), 3.27 (q, *J* = 7.4 Hz, 1H), 2.60 – 2.44 (m, 2H), 2.11 – 1.91 (m, 2H). **^{13}C NMR** (101 MHz, $CDCl_3$) δ 157.9, 142.7, 141.8, 134.1, 132.0, 129.4, 129.2, 128.7, 114.8, 113.9, 55.4, 48.6, 37.2, 32.7. **IR** (ATR): 2932, 2833, 1611, 1511, 1490, 1300, 1243, 1176, 1090, 1036, 1014, 915, 822 cm^{-1}. **HRMS** calculated for $C_{18}H_{19}ClO$ $[M]^+$ 286.1125, found 286.1137. **Chiral SFC**: 100 mm CHIRALCEL OJ-H, 0% *i*-PrOH, 2.0 mL/min, 220 nm, 44 °C, nozzle pressure = 200 bar CO_2, t_{R1} (minor) = 18.8 min, t_{R2} (major) = 19.9 min.

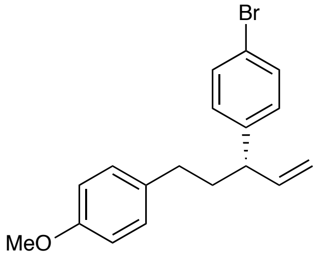

(*S*)-1-bromo-4-(5-(4-methoxyphenyl)pent-1-en-3-yl)benzene (2h): The title compound was synthesized according to the general procedure and isolated by preparatory TLC (5% EtOAc in hexanes) as a colorless oil (57.6 mg, 87% yield, >20:1 *rr*, 95:5 *er*, $[\alpha]^{24}_D$ = +6.3 (*c* 0.91, $CHCl_3$)). **^{1}H NMR** (400 MHz, $CDCl_3$) δ 7.46 (d, *J* = 8.4 Hz, 2H), 7.12 – 7.04 (m, 4H), 6.85 (d, *J* = 8.7 Hz, 2H), 5.95 (ddd, *J* = 17.2, 10.3, 7.4 Hz, 1H), 5.07 (ddt, *J* = 19.9, 17.1, 1.3 Hz, 2H), 3.81 (s, 3H), 3.26 (q, *J* = 7.5 Hz, 1H), 2.61 – 2.43 (m, 2H), 2.11 – 1.91 (m, 2H). **^{13}C NMR** (101 MHz, $CDCl_3$) δ 157.9, 143.2, 141.7, 134.0, 131.7, 129.6, 129.4, 120.1, 114.9, 113.9, 55.4, 48.6, 37.2, 32.7. **IR** (ATR): 2932, 2833, 1611, 1511, 1486, 1300, 1243, 1176, 1073, 1035, 1009, 915, 820 cm^{-1}. **HRMS** calculated for $C_{18}H_{19}BrO$ $[M]^+$ 330.0619, found 330.0616. **Chiral SFC**: 100 mm CHIRALCEL OJ-H, 1% *i*-PrOH, 3.0 mL/min, 220 nm, 44 °C, nozzle pressure = 200 bar CO_2, t_{R1} (minor) = 10.9 min, t_{R2} (major) = 11.6 min.

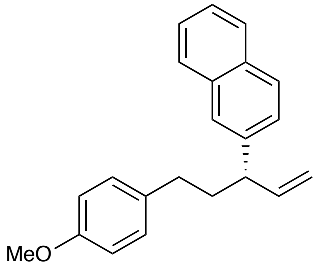

(*S*)-2-(5-(4-methoxyphenyl)pent-1-en-3-yl)naphthalene (2i): The title compound was synthesized according to the general procedure and isolated by preparatory TLC (5% EtOAc in hexanes) as a colorless oil (54.9 mg, 91% yield, >20:1 *rr*, 94:6 *er*, $[\alpha]^{24}_D$ = +9.4 (*c* 0.71, $CHCl_3$)). **^{1}H NMR** (400 MHz, $CDCl_3$) δ 7.88 – 7.82 (m, 3H), 7.68

(d, *J* = 1.4 Hz, 1H), 7.54 – 7.45 (m, 2H), 7.40 (dd, *J* = 8.5, 1.7 Hz, 1H), 7.12 (d, *J* = 8.7 Hz, 2H), 6.87 (d, *J* = 8.7 Hz, 2H), 6.16 – 6.04 (m, 1H), 5.17 – 5.09 (m, 2H), 3.83 (s, 3H), 3.49 (q, *J* = 7.5 Hz, 1H), 2.69 – 2.51 (m, 2H), 2.16 (q, *J* = 7.8 Hz, 2H). **^{13}C NMR** (101 MHz, $CDCl_3$) δ 157.9, 142.2, 141.7, 134.4, 133.8, 132.4, 129.5, 128.3, 127.8, 127.7, 126.4, 126.2, 126.1, 125.5, 114.7, 113.9, 55.4, 49.4, 37.2, 32.9. **IR** (ATR): 3054, 2932, 2833, 1611, 1511, 1454, 1300, 1243, 1176, 1035, 913, 817, 746 cm^{-1}. **HRMS** calculated for $C_{22}H_{22}O$ $[M]^+$ 302.1671, found 302.1658. **Chiral SFC**: 100 mm CHIRALCEL OJ-H, 10% *i*-PrOH, 3.0 mL/min, 220 nm, 44 °C, nozzle pressure = 200 bar CO_2, t_{R1} (major) = 5.7 min, t_{R2} (minor) = 6.3 min.

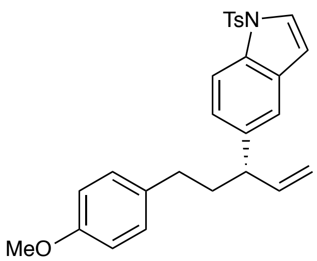

(*S*)-5-(5-(4-methoxyphenyl)pent-1-en-3-yl)-1-tosyl-1*H*-indole (2j): The title compound was synthesized according to the general procedure and isolated by preparatory TLC (5% EtOAc in hexanes) as a colorless oil (62.4 mg, 70% yield, >20:1 *rr*, 94:6 *er*, $[\alpha]^{24}_D$ = +8.4 (*c* 0.98, $CHCl_3$)). **^{1}H NMR** (400 MHz, $CDCl_3$) δ 7.92 (d, *J* = 8.6 Hz, 1H), 7.78 (d, *J* = 8.4 Hz, 2H), 7.55 (d, *J* = 3.7 Hz, 1H), 7.35 (d, *J* = 1.3 Hz, 1H), 7.23 (d, *J* = 8.3 Hz, 2H), 7.16 (dd, *J* = 8.6, 1.6 Hz, 1H), 7.05 (d, *J* = 8.6 Hz, 2H), 6.82 (d, *J* = 8.6 Hz, 2H), 6.61 (d, *J* = 3.6 Hz, 1H), 5.98 (ddd, *J* = 17.5, 9.8, 7.6 Hz, 1H), 5.10 – 4.98 (m, 2H), 3.79 (s, 3H), 3.34 (q, *J* = 7.4 Hz, 1H), 2.61 – 2.40 (m, 2H), 2.35 (s, 3H), 2.12 – 1.93 (m, 2H). **^{13}C NMR** (101 MHz, $CDCl_3$) δ 157.9, 145.0, 142.5, 139.5, 135.6, 134.3, 133.6, 131.2, 130.0, 129.4, 127.0, 126.6, 124.7, 120.1, 114.4, 113.9, 113.6, 109.1, 55.4, 49.2, 37.6, 32.9, 21.7. **IR** (ATR): 2931, 1611, 1596, 1511, 1456, 1369, 1243, 1170, 1126, 1034, 995, 810, 725, 703, 667 cm^{-1}. **HRMS** calculated for $C_{27}H_{27}NO_3SNa$ $[M+Na]^+$ 468.1609, found 468.1622. **Chiral SFC**: 100 mm CHIRALCEL OJ-H, 10% *i*-PrOH, 2.0 mL/min, 220 nm, 44 °C, nozzle pressure = 200 bar CO_2, t_{R1} (major) = 13.3 min, t_{R2} (minor) = 14.1 min.

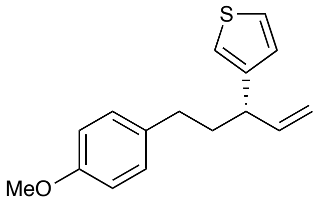

(*S*)-3-(5-(4-methoxyphenyl)pent-1-en-3-yl)thiophene (2k): The title compound was synthesized according to the general procedure and isolated by preparatory TLC (5% EtOAc in hexanes) as a colorless oil (42.4 mg, 82% yield, >20:1 *rr*, 94:6 *er*, $[\alpha]^{24}_D$ = +23.4 (*c* 1.1, $CHCl_3$)). **^{1}H NMR** (400 MHz, $CDCl_3$) δ 7.38 (d, *J* = 8.1 Hz, 2H), 7.20 – 7.15 (m, 2H), 7.08 (d, *J* = 8.8 Hz, 2H), 6.83 (d, *J* = 8.7 Hz, 2H), 6.72 (dd, *J* = 17.6, 10.9 Hz, 1H), 6.03 – 5.91 (m, 1H), 5.74 (dd, *J* = 17.6, 1.0 Hz, 1H), 5.22 (dd, *J* = 10.9, 1.0 Hz, 1H), 5.09 – 5.01 (m, 2H), 3.80 (s, 3H), 3.28 (q, *J* = 7.5 Hz, 1H), 2.62 – 2.44 (m, 2H), 2.08 – 1.96 (m, 2H). **^{13}C NMR**

(101 MHz, $CDCl_3$) δ 157.9, 145.0, 141.7, 134.3, 129.5, 127.3, 125.6, 120.1, 114.7, 113.9, 55.4, 44.7, 37.2, 32.8. **IR** (ATR): 2932, 2833, 1611, 1510, 1441, 1300, 1243, 1176, 1035, 915, 829, 781 cm^{-1}. **HRMS** calculated for $C_{16}H_{18}OS$ $[M]^+$ 258.1078, found 258.1074. **Chiral SFC**: 100 mm CHIRALCEL OJ-H, 5% *i*-PrOH, 2.0 mL/min, 220 nm, 44 °C, nozzle pressure = 200 bar CO_2, t_{R1} (minor) = 4.9 min, t_{R2} (major) = 5.4 min.

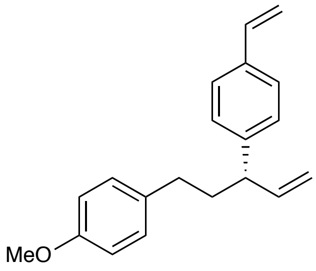

(*S*)-1-methoxy-4-(3-(4-vinylphenyl)pent-4-en-1-yl)benzene (2l): The title compound was synthesized according to the general procedure and isolated by preparatory TLC (5% EtOAc in hexanes) as a colorless oil (45.0 mg, 81% yield, >20:1 *rr*, 93:7 *er*, $[\alpha]^{24}_D$ = +7.0 (*c* 0.65, $CHCl_3$)). **^{1}H NMR** (400 MHz, $CDCl_3$) δ 7.38 (d, *J* = 8.1 Hz, 2H), 7.20 – 7.14 (m, 2H), 7.08 (d, *J* = 8.8 Hz, 2H), 6.83 (d, *J* = 8.7 Hz, 2H), 6.72 (dd, *J* = 17.6, 10.9 Hz, 1H), 5.97 (ddd, *J* = 16.5, 10.8, 7.5 Hz, 1H), 5.74 (dd, *J* = 17.6, 1.0 Hz, 1H), 5.22 (dd, *J* = 10.9, 1.0 Hz, 1H), 5.10 – 5.00 (m, 2H), 3.80 (s, 3H), 3.28 (q, *J* = 7.5 Hz, 1H), 2.61 – 2.44 (m, 2H), 2.07 – 1.96 (m, 2H). **^{13}C NMR** (101 MHz, $CDCl_3$) δ 157.9, 144.0, 142.2, 136.8, 135.8, 134.4, 129.5, 128.0, 126.5, 114.5, 113.9, 113.3, 55.4, 49.0, 37.2, 32.8. **IR** (ATR): 3001, 2933, 2833, 1611, 1510, 1441, 1300, 1243, 1176, 1036, 990, 908, 827 cm^{-1}. **HRMS** calculated for $C_{20}H_{22}O$ $[M]^+$ 278.1671, found 278.1658. **Chiral SFC**: 100 mm CHIRALCEL AS-H, 1% *i*-PrOH, 2.0 mL/min, 220 nm, 44 °C, nozzle pressure = 200 bar CO_2, t_{R1} (major) = 2.8 min, t_{R2} (minor) = 3.3 min.

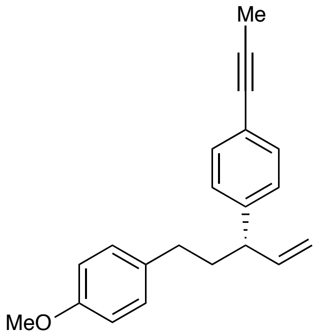

(*S*)-1-methoxy-4-(3-(4-(prop-1-yn-1-yl)phenyl)pent-4-en-1-yl)benzene (2m): The title compound was synthesized according to the general procedure and isolated by preparatory TLC (5% EtOAc in hexanes) as a colorless oil (45.4 mg, 78% yield, >20:1 *rr*, 95:5 *er*, $[\alpha]^{24}_D$ = +6.0 (*c* 0.99, $CHCl_3$)). **^{1}H NMR** (400 MHz, $CDCl_3$) δ 7.37 (d, *J* = 8.3 Hz, 2H), 7.14 (d, *J* = 8.1 Hz, 2H), 7.08 (d, *J* = 8.8 Hz, 2H), 6.85 (d, *J* = 8.7 Hz, 2H), 5.97 (ddd, *J* = 17.0, 10.3, 7.5 Hz, 1H), 5.12 – 5.00 (m, 2H), 3.81 (s, 3H), 3.27 (q, *J* = 7.4 Hz, 1H), 2.60 – 2.45 (m, 2H), 2.07 (s, 3H), 2.06 – 1.92 (m, 2H). **^{13}C NMR** (101 MHz, $CDCl_3$) δ 157.9, 143.7, 141.9, 134.2, 131.8, 129.4, 127.7, 122.0, 114.6, 113.9, 85.4, 79.8, 55.4, 49.1, 37.2, 32.8, 4.5. **IR** (ATR): 2915, 2833, 1611, 1510, 1441, 1300, 1243, 1176, 1036, 915, 830 cm^{-1}. **HRMS** calculated for $C_{21}H_{22}O$ $[M]^+$ 290.1671, found

290.1667. **Chiral SFC**: 100 mm CHIRALCEL OJ-H, 5% *i*-PrOH, 2.0 mL/min, 220 nm, 44 °C, nozzle pressure = 200 bar CO_2, t_{R1} (major) = 10.2 min, t_{R2} (minor) = 10.8 min.

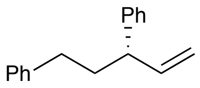

(*S*)-pent-4-ene-1,3-diyldibenzene (2n): The title compound was synthesized according to the general procedure and isolated by preparatory TLC (hexanes) as a colorless oil (50.2 mg, 99% yield, >20:1 *rr*, 93:7 *er*, $[\alpha]^{24}_{D}$ = +12.7 (*c* 1.6, $CHCl_3$)). **^{1}H NMR** (400 MHz, CD_2Cl_2) δ 7.35 – 7.25 (m, 4H), 7.24 – 7.20 (m, 3H), 7.20 – 7.14 (m, 3H), 6.01 (ddd, *J* = 17.6, 9.8, 7.7 Hz, 1H), 5.12 – 5.00 (m, 2H), 3.30 (q, *J* = 7.6 Hz, 1H), 2.69 – 2.45 (m, 2H), 2.11 – 1.98 (m, 2H). **^{13}C NMR** (101 MHz, $CDCl_3$) δ 145.1, 143.2, 143.1, 129.3, 129.2, 129.1, 128.5, 127.0, 126.5, 114.9, 50.3, 38.0, 34.5. **IR** (ATR): 3026, 2923, 1636, 1601, 1494, 1452, 1029, 993, 912, 765, 746, 697 cm^{-1}. **HRMS** calculated for $C_{17}H_{18}$ $[M]^+$ 222.1409, found 222.1418. **Chiral SFC**: 100 mm CHIRALCEL OJ-H, 4% *i*-PrOH, 2.3 mL/min, 220 nm, 44 °C, nozzle pressure = 200 bar CO_2, t_{R1} (minor) = 2.5 min, t_{R2} (major) = 2.7 min.

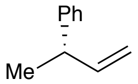

(*S*)-but-3-en-2-ylbenzene (2o): The title compound was synthesized according to the general procedure and isolated by column chromatography (pentanes) as a colorless liquid (15.9 mg, 60% yield, >20:1 *rr*, 92:8 *er*, $[\alpha]^{24}_{D}$ = +3.0 (*c* 0.84, $CHCl_3$)). The ^{1}H and ^{13}C NMR were in accordance with the literature[5]. **^{1}H NMR** (500 MHz, $CDCl_3$) δ 7.34 (t, *J* = 7.6 Hz, 2H), 7.28 – 7.21 (m, 3H), 6.05 (ddd, *J* = 16.9, 10.3, 6.4 Hz, 1H), 5.15 – 5.02 (m, 2H), 3.51 (p, *J* = 7.0 Hz, 1H), 1.41 (d, *J* = 7.1 Hz, 3H). **^{13}C NMR** (126 MHz, $CDCl_3$) δ 145.7, 143.4, 128.5, 127.4, 126.3, 113.2, 43.3, 20.9. **Chiral SFC**: 100 mm CHIRALCEL OJ-H, 0.1% *i*-PrOH, 2.0 mL/min, 220 nm, 44 °C, nozzle pressure = 200 bar CO_2, t_{R1} (minor) = 1.7 min, t_{R2} (major) = 1.9 min.

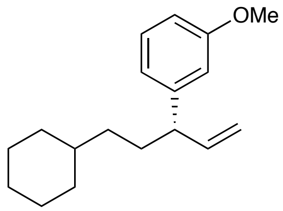

(*S*)-1-(5-cyclohexylpent-1-en-3-yl)-3-methoxybenzene (2p): The title compound was synthesized according to the general procedure and isolated by preparatory TLC (5% EtOAc in hexanes) as a colorless oil (51.2 mg, 99% yield, >20:1 *rr*, 94:6 *er*, $[\alpha]^{24}_{D}$ = +20.8 (*c* 0.55, $CHCl_3$)). **^{1}H NMR** (400 MHz, $CDCl_3$) δ 7.27 – 7.20 (m, 1H), 6.85 – 6.79 (m, 1H), 6.79 – 6.71 (m, 2H), 5.96 (ddd, *J* = 17.1, 10.3, 7.7 Hz, 1H), 5.09 – 4.99 (m, 2H), 3.83 (s, 3H), 3.19 (q, *J* = 7.5 Hz, 1H), 1.75 – 1.63 (m, 7H), 1.28 – 1.08 (m, 6H), 0.95 – 0.81 (m, 2H). **^{13}C NMR** (101 MHz, $CDCl_3$) δ 159.8, 146.6, 142.6, 129.4, 120.1, 114.0, 113.7, 111.2, 55.3, 50.4, 37.9, 35.4, 33.51, 33.49, 32.8, 26.9, 26.56, 26.55. **IR** (ATR): 3026, 2923, 1636, 1601, 1494, 1452,

1029, 993, 912, 765, 746, 697 cm^{-1}. **HRMS** calculated for $C_{18}H_{26}OH$ $[M+H]^+$ 259.2062, found 259.2054. **Chiral SFC**: 100 mm CHIRALCEL OJ-H, 1% *i*-PrOH, 2.0 mL/min, 220 nm, 44 °C, nozzle pressure = 200 bar CO_2, t_{R1} (minor) = 2.2 min, t_{R2} (major) = 2.4 min.

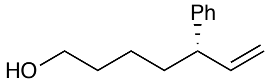

(*S*)-5-phenylhept-6-en-1-ol (2q): The title compound was synthesized according to the general procedure and isolated by preparatory TLC (25% EtOAc in hexanes) as a colorless oil (23.2 mg, 61% yield, >20:1 *rr*, 96:4 *er*, $[\alpha]^{24}_{D}$ = +30.1 (*c* 1.0, $CHCl_3$)). **^{1}H NMR** (400 MHz, $CDCl_3$) δ 7.34 – 7.28 (m, 2H), 7.24 – 7.17 (m, 3H), 6.03 – 5.89 (m, 1H), 5.09 – 4.97 (m, 2H), 3.62 (dd, *J* = 11.9, 6.5 Hz, 2H), 3.26 (q, *J* = 7.5 Hz, 1H), 1.80 – 1.68 (m, 2H), 1.63 – 1.50 (m, 2H), 1.46 – 1.34 (m, 1H), 1.34 – 1.21 (m, 2H). **^{13}C NMR** (101 MHz, $CDCl_3$) δ 144.5, 142.4, 128.6, 127.7, 126.3, 114.2, 63.0, 50.0, 35.3, 32.8, 23.9. **IR** (ATR): 3331, 3026, 2932, 2860, 1636, 1600, 1492, 1452, 1054, 911, 756, 698 cm^{-1}. **HRMS** calculated for $C_{13}H_{18}O$ $[M]^+$ 190.1358, found 190.1356. **Chiral SFC**: 100 mm CHIRALCEL OJ-H, 5% *i*-PrOH, 2.0 mL/min, 220 nm, 44 °C, nozzle pressure = 200 bar CO_2, t_{R1} (minor) = 4.1 min, t_{R2} (major) = 4.4 min.

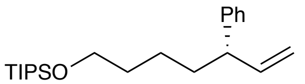

(*S*)-triisopropyl((5-phenylhept-6-en-1-yl)oxy)silane (2r): The title compound was synthesized according to the general procedure and isolated by preparatory TLC (hexanes) as a colorless oil (66.5 mg, 96% yield, >20:1 *rr*, 95:5 *er*, $[\alpha]^{24}_{D}$ = +12.8 (*c* 0.81, $CHCl_3$)). **^{1}H NMR** (400 MHz, $CDCl_3$) δ 7.36 – 7.28 (m, 2H), 7.25 – 7.17 (m, 3H), 5.99 (ddd, *J* = 16.9, 10.5, 7.6 Hz, 1H), 5.11 – 5.00 (m, 2H), 3.68 (t, *J* = 6.6 Hz, 2H), 3.28 (q, *J* = 7.5 Hz, 1H), 1.83 – 1.69 (m, 2H), 1.59 (ddt, *J* = 13.3, 7.5, 3.8 Hz, 2H), 1.46 – 1.36 (m, 1H), 1.36 – 1.24 (m, 1H), 1.11 – 1.05 (m, 21H). **^{13}C NMR** (101 MHz, $CDCl_3$) δ 144.7, 142.5, 128.5, 127.7, 126.2, 114.1, 63.5, 50.1, 35.4, 33.1, 24.0, 18.2, 12.2. **IR** (ATR): 2940, 2964, 1637, 1462, 1382, 1104, 1068, 994, 911, 881, 698 cm^{-1}. **HRMS** calculated for $C_{22}H_{38}OSiH$ $[M+H]^+$ 347.2770, found 347.2783. The enantioselectivity was determined using the corresponding alcohol **2q** after desilyation with TBAF. **Chiral SFC**: 100 mm CHIRALCEL OJ-H, 5% *i*-PrOH, 2.0 mL/min, 220 nm, 44 °C, nozzle pressure = 200 bar CO_2, t_{R1} (minor) = 4.1 min, t_{R2} (major) = 4.3 min.

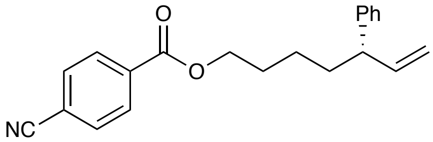

(*S*)-5-phenylhept-6-en-1-yl 4-cyanobenzoate (2s): The title compound was synthesized according to the general procedure using 1,2-dichloroethane as the solvent and heating at 60 °C. It was isolated by preparatory TLC (10% EtOAc in hexanes) as a colorless oil

(43.0 mg, 67% yield, >20:1 *rr*, 89:11 *er*, $[\alpha]^{24}_{D}$ = +7.9 (*c* 0.47, $CHCl_3$)). **^{1}H NMR** (400 MHz, $CDCl_3$) δ 8.09 (d, *J* = 8.7 Hz, 2H), 7.73 (d, *J* = 8.7 Hz, 2H), 7.34 – 7.28 (m, 2H), 7.21 (td, *J* = 6.9, 1.5 Hz, 3H), 6.03 – 5.90 (m, 1H), 5.09 – 5.00 (m, 2H), 4.33 (td, *J* = 6.6, 1.0 Hz, 2H), 3.28 (q, *J* = 7.5 Hz, 1H), 1.86 – 1.73 (m, 4H), 1.54 – 1.42 (m, 1H), 1.42 – 1.31 (m, 1H). **^{13}C NMR** (101 MHz, $CDCl_3$) δ 165.0, 144.2, 142.2, 134.3, 132.3, 130.1, 128.6, 127.6, 126.4, 118.1, 116.4, 114.3, 65.7, 49.8, 34.9, 28.5, 23.9. **IR** (ATR): 2924, 2231, 1721, 1636, 1452, 1272, 1107, 913, 860, 767 cm^{-1}. **HRMS** calculated for $C_{21}H_{20}NO_2H$ $[M+H]^+$ 319.1572, found 319.1571. **Chiral SFC**: 100 mm CHIRALCEL OJ-H, 5% *i*-PrOH, 2.0 mL/min, 220 nm, 44 °C, nozzle pressure = 200 bar CO_2, t_{R1} (minor) = 5.9 min, t_{R2} (major) = 7.0 min.

Synthesis of Josiphos Ligand L6

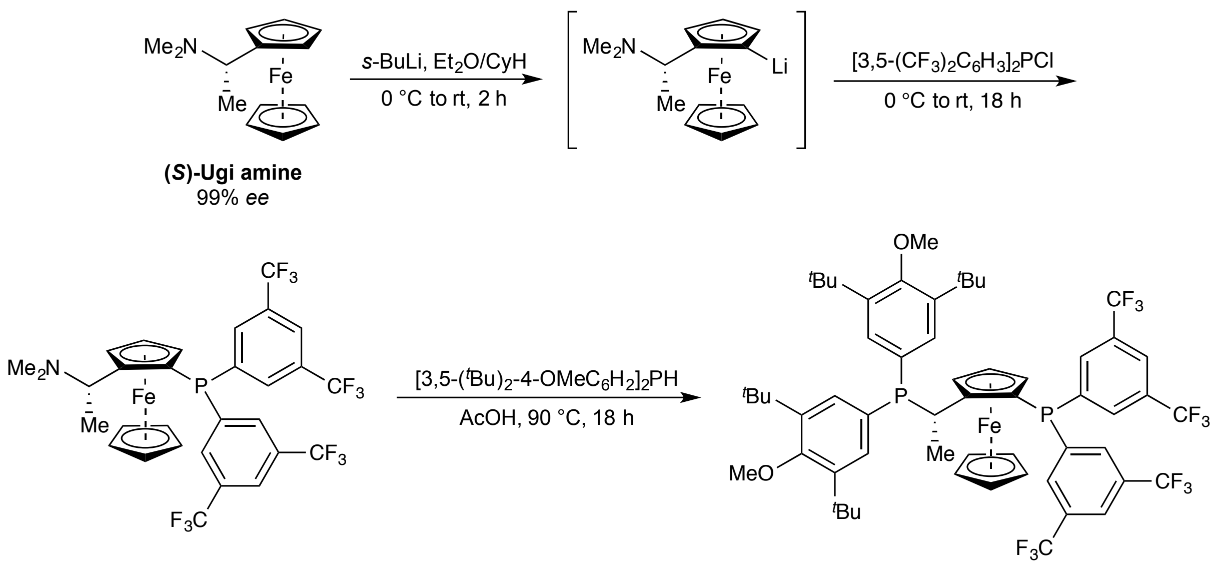

(*S*)-1-{[(*R*$_p$)-2-[Bis[3,5-bis(trifluoromethyl)phenyl]phosphino]ferrocenyl}ethylbis(3,5-di-*tert*-butyl-4-methoxyphenyl)phosphine (L6): To a flame-dried round bottom flask was charged (*S*)-Ugi amine[6-9] (262.3 mg, 1.02 mmol, 1 equiv) and anhydrous Et_2O (2 mL). The resulting suspension was cooled to 0 °C, and *s*-BuLi (1.4 M in cyclohexane, 0.80 mL, 1.12 mmol, 1.1 equiv) was added dropwise. The resulting red solution was stirred at rt for 2 h. The solution was cooled to 0 °C, and a solution of bis(3,5-di(trifluoromethyl)phenyl)chlorophosphine (502.5 mg, 1.02 mmol, 1 equiv) in anhydrous Et_2O (1 mL) was added dropwise. The resulting mixture was stirred at rt for 18 h. The reaction mixture was quenched with a saturated aqueous solution of $NaHCO_3$ (5 mL). The resulting mixture was extracted with Et_2O (3 x 10 mL). The combined organic layers were washed with brine, dried with anhydrous Na_2SO_4, filtered, and concentrated

in vacuo. Purification of the resulting residue by silica gel column chromatography (10% EtOAc in hexanes) gave the ferrocenyl monophosphine as a red oil (563 mg, 77% yield).

A flame-dried round bottom flask equipped with a condenser was charged with the ferrocenyl monophosphine (562.9 mg, 0.789 mmol, 1 equiv), bis(3,5-di-*tert*-butyl-4-methoxyphenyl)phosphine (408.6 mg, 0.868 mmol, 1.1 equiv), and glacial acetic acid (distilled, degassed, 2.6 mL). The resulting mixture was heated at 90 °C for 18 h. The reaction mixture was cooled to rt, and most of the acetic acid was removed under reduced pressure. The resulting residue was purified by silica gel column chromatography (10% CH_2Cl_2 in hexanes) to give the title compound as an orange solid (781 mg, 87% yield). **^{1}H NMR** (500 MHz, CD_2Cl_2) δ 8.16 (d, *J* = 7.1 Hz, 2H), 8.03 (s, 1H), 7.83 – 7.79 (m, 2H), 7.77 (s, 1H), 7.24 (dd, *J* = 6.6, 0.9 Hz, 2H), 6.96 (dd, *J* = 7.2, 0.9 Hz, 2H), 4.46 (t, *J* = 2.5 Hz, 1H), 4.00 (t, *J* = 1.8 Hz, 1H), 3.93 (s, 1H), 3.87 (s, 5H), 3.70 (s, 3H), 3.68 – 3.67 (m, 4H), 1.42 (s, 18H), 1.35 (s, 18H), 1.30 (dd, *J* = 6.9, 4.6 Hz, 3H). **^{13}C NMR** (126 MHz, CD_2Cl_2) δ 161.1, 160.3, 144.3 (d, *J* = 5.1 Hz), 143.5 (d, *J* = 6.9 Hz), 142.6 (d, *J* = 2.9 Hz), 142.5 (d, *J* = 3.0 Hz), 136.1 (d, *J* = 23.7 Hz), 133.8 (d, *J* = 20.8 Hz), 133.3 (d, *J* = 17.6 Hz), 132.2 (qd, *J* = 33.1, 7.8 Hz), 131.4 (qd, *J* = 33.2, 5.1 Hz), 131.0 (d, *J* = 17.3 Hz), 128.0 (d, *J* = 1.8 Hz), 127.9 (d, *J* = 1.8 Hz), 124.3 (dt, *J* = 7.7, 3.9 Hz), 124.2 (q, *J* = 273.0 Hz), 124.1 (q, *J* = 273.3 Hz), 122.7 (p, *J* = 3.6 Hz), 101.6 (d, *J* = 22.1 Hz), 101.3 (d, *J* = 22.2 Hz), 71.19, 71.17 – 70.9 (m), 71.1 – 70.8 (m), 70.3, 65.1, 65.0, 36.5, 36.4, 32.63, 32.55, 32.1 (dd, *J* = 20.0, 10.3 Hz), 15.8 (d, *J* = 3.3 Hz). **^{31}P NMR** (162 MHz, CD_2Cl_2) δ 12.1 (d, *J* = 34.9 Hz), -21.9 (d, *J* = 36.2 Hz). **^{19}F NMR** (376 MHz, CD_2Cl_2) δ -63.17, -63.22. **IR** (ATR): 2961, 1352, 1275, 1172, 1136, 1095, 1009, 892, 703, 681 cm^{-1}. **HRMS** calculated for $C_{58}H_{64}F_{12}FeO_2P_2$ $[M]^+$ 1138.3541, found 1138.3544. $[\alpha]^{24}_D$ = +184.1 (*c* 0.67, $CHCl_3$)).

Preparation of Allenes 1

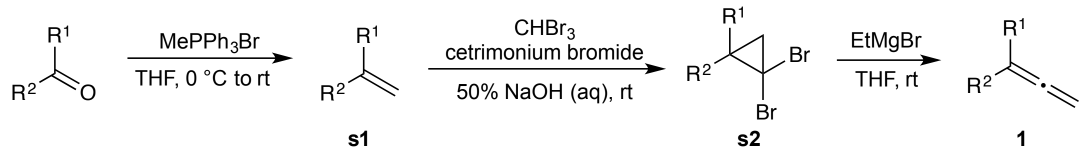

General Procedure for the Wittig Olefination

To a flame-dried round bottom flask was added methyltriphenylphosphonium bromide (1.5 equiv) and THF (0.5 M). KO*t*-Bu (1.5 equiv) was added, and the resulting mixture was stirred for 45 minutes at rt. A solution of the ketone (1 equiv) in THF (0.5 M) was added dropwise at 0 °C, and the reaction mixture was stirred at rt for 1 h. The reaction mixture was filtered through celite

and concentrated *in vacuo*. The residue was purified by column chromatography to afford the pure 1, 1-disubstituted alkene **s1**.

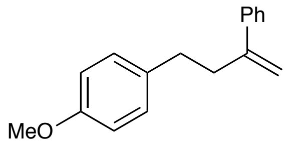

1-methoxy-4-(3-phenylbut-3-en-1-yl)benzene (s1a): The title compound was prepared using the general procedure for the Wittig olefination from 3-(4-methoxyphenyl)-1-phenylpropan-1-one (1.82 g, 7.59 mmol, 1 equiv), methyltriphenylphosphonium bromide (4.07 g, 11.4 mmol, 1.5 equiv), KO*t*-Bu (1.28 g, 11.4 mmol, 1.5 equiv), and THF (30.4 mL, 0.25 M). Isolated by column chromatography (5% EtOAc in hexanes) as a colorless oil (1.78 g, 99% yield). **^{1}H NMR** (400 MHz, $CDCl_3$) δ 7.48 (dt, *J* = 3.2, 1.9 Hz, 2H), 7.43 – 7.36 (m, 2H), 7.33 (ddd, *J* = 7.2, 3.7, 1.3 Hz, 1H), 7.14 (d, *J* = 8.6 Hz, 2H), 6.87 (d, *J* = 8.6 Hz, 2H), 5.34 (d, *J* = 1.2 Hz, 1H), 5.10 (d, *J* = 1.2 Hz, 1H), 3.83 (s, 3H), 2.86 – 2.80 (m, 2H), 2.78 – 2.72 (m, 2H). **^{13}C NMR** (101 MHz, $CDCl_3$) δ 157.9, 148.0, 141.3, 134.2, 129.4, 128.5, 127.5, 126.3, 113.9, 112.8, 55.4, 37.7, 34.0. **IR** (ATR): 3030, 2933, 2833, 1611, 1511, 1299, 1243, 1176, 1036, 894, 822, 778, 701 cm^{-1}. **HRMS** calculated for $C_{17}H_{18}O$ $[M]^+$ 238.1358, found 238.1358.

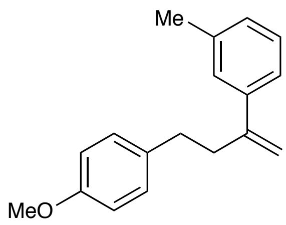

1-(4-(4-methoxyphenyl)but-1-en-2-yl)-3-methylbenzene (s1b): The title compound was prepared using the general procedure for the Wittig olefination from 3-(4-methoxyphenyl)-1-(*m*-tolyl)propan-1-one (763 mg, 3.0 mmol, 1 equiv), methyltriphenylphosphonium bromide (1.68 g, 4.5 mmol, 1.5 equiv), KO*t*-Bu (505 mg, 4.5 mmol, 1.5 equiv), and THF (12.0 mL, 0.25 M). Isolated by column chromatography (5% EtOAc in hexanes) as a yellow oil (745 mg, 98% yield). **^{1}H NMR** (400 MHz, $CDCl_3$) δ 7.34 – 7.33 (m, 3H), 7.21 – 7.18 (m, 3H), 6.93 (d, *J* = 8.5 Hz, 2H), 5.39 (s, 1H), 5.14 (s, 1H), 3.87 (s, 3H), 2.92 – 2.85 (m, 2H), 2.82 (dd, *J* = 8.3, 5.4 Hz, 2H), 2.47 (s, 3H). **^{13}C NMR** (101 MHz, $CDCl_3$) δ 157.9, 148.1, 141.3, 137.9, 134.2, 129.4, 128.34, 128.26, 127.0, 123.4, 113.8, 112.5, 55.3, 37.7, 34.0, 21.6. **IR** (ATR): 2932, 2833, 1611, 1582, 1511, 1454, 1299, 1243, 1176, 1037, 893, 791 cm^{-1}. **HRMS** calculated for $C_{18}H_{20}O$ $[M]^+$ 252.1514, found 252.1510.

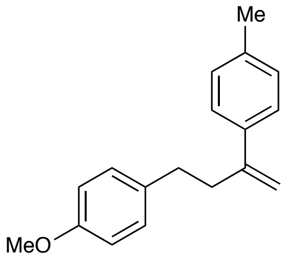

1-methoxy-4-(3-(*p*-tolyl)but-3-en-1-yl)benzene (s1c): The title compound was prepared using the general procedure for the Wittig olefination from 3-(4-methoxyphenyl)-1-(*p*-tolyl)propan-1-one (763 mg, 3.0 mmol, 1 equiv), methyltriphenylphosphonium bromide (1.68 g, 4.5 mmol, 1.5 equiv), KO*t*-Bu (505 mg, 4.5 mmol, 1.5 equiv), and THF (12.0 mL, 0.25 M). Isolated by column chromatography (5% EtOAc in hexanes) as a colorless oil (705 mg, 93% yield). **^{1}H NMR** (400 MHz, CD_2Cl_2) δ 7.33 (d, *J* = 8.1 Hz, 2H), 7.15 (d, *J* = 7.9 Hz, 2H), 7.08 (d, *J* = 8.8 Hz, 2H), 6.80 (d, *J* = 8.7 Hz, 2H), 5.25 (d, *J* = 1.4 Hz, 1H), 4.99 (d, *J* = 1.3 Hz, 1H), 3.75 (s, 3H), 2.79 – 2.71 (m, 2H), 2.71 – 2.64 (m, 2H), 2.34 (s, 3H). **^{13}C NMR** (101 MHz, CD_2Cl_2) δ 158.7, 148.6, 138.9, 138.1, 134.9, 130.1, 129.8, 126.7, 114.4, 112.3, 55.9, 38.3, 34.6, 21.6. **IR** (ATR): 2932, 2833, 1611, 1511, 1454, 1299, 1243, 1176, 1037, 891, 821 cm^{-1}. **HRMS** calculated for $C_{18}H_{20}OH$ $[M+H]^+$ 253.1592, found 253.1583.

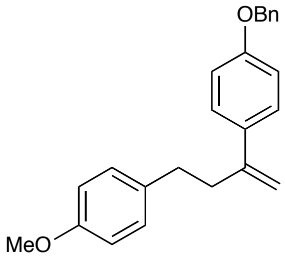

1-(benzyloxy)-4-(4-(4-methoxyphenyl)but-1-en-2-yl)benzene (s1d): The title compound was prepared using the general procedure for the Wittig olefination from 1-(4-(benzyloxy)phenyl)-3-(4-methoxyphenyl)propan-1-one (749 mg, 2.16 mmol, 1 equiv), methyltriphenylphosphonium bromide (1.16 g, 3.24 mmol, 1.5 equiv), KO*t*-Bu (364 mg, 3.24 mmol, 1.5 equiv), and THF (8.7 mL, 0.25 M). Isolated by column chromatography (5% EtOAc in hexanes) as a white solid (723 mg, 97% yield). **^{1}H NMR** (400 MHz, $CDCl_3$) δ 7.49 – 7.45 (m, 2H), 7.45 – 7.32 (m, 5H), 7.12 (d, *J* = 8.6 Hz, 2H), 6.98 (d, *J* = 8.8 Hz, 2H), 6.85 (d, *J* = 8.6 Hz, 2H), 5.25 (d, *J* = 1.3 Hz, 1H), 5.11 (s, 2H), 5.00 (d, *J* = 1.0 Hz, 1H), 3.81 (s, 3H), 2.83 – 2.68 (m, 4H). **^{13}C NMR** (101 MHz, $CDCl_3$) δ 158.4, 157.9, 147.2, 137.1, 134.3, 133.9, 129.4, 128.7, 128.1, 127.6, 127.4, 114.8, 113.9, 111.3, 70.2, 55.4, 37.7, 34.0. **IR** (ATR): 3038, 2912, 2864, 1603, 1508, 1454, 1379, 1287, 1243, 1179, 1010, 891 cm^{-1}. **HRMS** calculated for $C_{24}H_{24}O_2Na$ $[M+Na]^+$ 367.1674, found 367.1686.

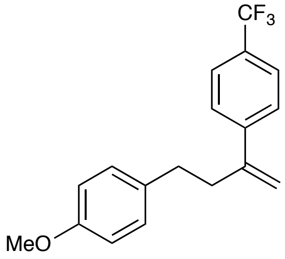

1-methoxy-4-(3-(4-(trifluoromethyl)phenyl)but-3-en-1-yl)benzene (s1e): The title compound was prepared using the general procedure for the Wittig olefination from 3-(4-methoxyphenyl)-1-(4-(trifluoromethyl)phenyl)propan-1-one (837 mg, 2.72 mmol, 1 equiv), methyltriphenylphosphonium bromide (1.46 g, 4.07 mmol, 1.5 equiv),

KO*t*-Bu (457 mg, 4.07 mmol, 1.5 equiv), and THF (10.9 mL, 0.25 M). Isolated by column chromatography (5% EtOAc in hexanes) as a colorless oil (800 mg, 96% yield). **^{1}H NMR** (400 MHz, $CDCl_3$) δ 7.61 (d, *J* = 8.2 Hz, 2H), 7.52 (d, *J* = 8.3 Hz, 2H), 7.09 (d, *J* = 8.5 Hz, 2H), 6.84 (d, *J* = 8.4 Hz, 2H), 5.36 (s, 1H), 5.17 (s, 1H), 3.81 (s, 3H), 2.80 (dd, *J* = 12.3, 4.6 Hz, 2H), 2.75 – 2.67 (m, 2H). **^{13}C NMR** (101 MHz, $CDCl_3$) δ 158.0, 147.0, 144.9 (q, $J_{C\text{-}F}$ = 1.3 Hz), 133.7, 129.5 (q, $J_{C\text{-}F}$ = 32.3 Hz), 129.4, 126.6, 125.5 (q, $J_{C\text{-}F}$ = 3.8 Hz), 124.2 (q, $J_{C\text{-}F}$ = 272.3 Hz), 114.8, 113.9, 55.4, 37.5, 33.8. **^{19}F NMR** (376 MHz, $CDCl_3$) δ -62.9. **IR** (ATR): 2936, 1615, 1512, 1323, 1301, 1244, 1163, 1115, 1066, 1037, 1014, 903 cm^{-1}. **HRMS** calculated for $C_{18}H_{17}F_3O$ $[M]^+$ 306.1231, found 306.1235.

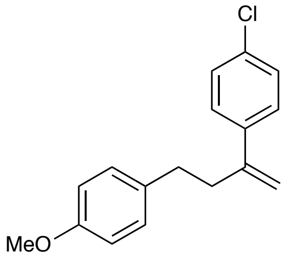

1-chloro-4-(4-(4-methoxyphenyl)but-1-en-2-yl)benzene (s1f): The title compound was prepared using the general procedure for the Wittig olefination from 1-(4-chlorophenyl)-3-(4-methoxyphenyl)propan-1-one (586 mg, 2.13 mmol, 1 equiv), methyltriphenylphosphonium bromide (1.14 g, 3.20 mmol, 1.5 equiv), KO*t*-Bu (359 mg, 3.20 mmol, 1.5 equiv), and THF (8.5 mL, 0.25 M). Isolated by column chromatography (5% EtOAc in hexanes) as a colorless oil (494 mg, 85% yield). **^{1}H NMR** (400 MHz, $CDCl_3$) δ 7.37 (d, *J* = 8.6 Hz, 2H), 7.32 (d, *J* = 8.6 Hz, 2H), 7.09 (d, *J* = 8.5 Hz, 2H), 6.84 (d, *J* = 8.5 Hz, 2H), 5.29 (s, 1H), 5.08 (s, 1H), 3.81 (s, 3H), 2.77 (dd, *J* = 9.7, 7.0 Hz, 2H), 2.74 – 2.66 (m, 2H). **^{13}C NMR** (126 MHz, $CDCl_3$) δ 158.0, 146.9, 139.7, 133.9, 133.3, 129.4, 128.6, 127.6, 113.9, 113.4, 55.4, 37.5, 33.8. **IR** (ATR): 2933, 2833, 1611, 1511, 1491, 1300, 1243, 1176, 1096, 1036, 1011, 897 cm^{-1}. **HRMS** calculated for $C_{17}H_{17}ClO$ $[M]^+$ 272.0968, found 272.0974.

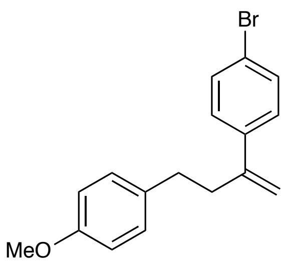

1-bromo-4-(4-(4-methoxyphenyl)but-1-en-2-yl)benzene (s1g): The title compound was prepared using the general procedure for the Wittig olefination from 1-(4-bromophenyl)-3-(4-methoxyphenyl)propan-1-one (580 mg, 1.82 mmol, 1 equiv), methyltriphenylphosphonium bromide (973 mg, 2.73 mmol, 1.5 equiv), KO*t*-Bu (306 mg, 2.73 mmol, 1.5 equiv), and THF (8.5 mL, 0.25 M). Isolated by column chromatography (5% EtOAc in hexanes) as a colorless oil (559 mg, 97% yield). **^{1}H NMR** (400 MHz, $CDCl_3$) δ 7.48 (d, *J* = 8.4 Hz, 2H), 7.30 (d, *J* = 8.4 Hz, 2H), 7.08 (d, *J* = 8.5 Hz, 2H), 6.84 (d, *J* = 8.5 Hz, 2H), 5.29 (s, 1H), 5.08 (s, 1H), 3.80 (s, 3H), 2.75 (d, *J* = 4.7 Hz, 2H), 2.73 – 2.65 (m, 2H). **^{13}C NMR** (126 MHz, $CDCl_3$) δ 158.0, 146.9, 140.2, 133.8, 131.6, 129.4, 128.0, 121.5, 113.9, 113.5, 55.4, 37.5,

33.8. **IR** (ATR): 2932, 2833, 1611, 1511, 1487, 1300, 1243, 1176, 1036, 1007, 897, 822 cm^{-1}. **HRMS** calculated for $C_{17}H_{17}BrO$ $[M]^+$ 316.0463, found 316.0451.

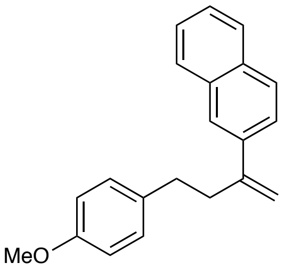

2-(4-(4-methoxyphenyl)but-1-en-2-yl)naphthalene (s1h): The title compound was prepared using the general procedure for the Wittig olefination from 3-(4-methoxyphenyl)-1-(naphthalen-2-yl)propan-1-one (1.18 g, 4.08 mmol, 1 equiv), methyltriphenylphosphonium bromide (2.19 g, 6.12 mmol, 1.5 equiv), KO*t*-Bu (687 mg, 6.12 mmol, 1.5 equiv), and THF (16.3 mL, 0.25 M). Isolated by column chromatography (5% EtOAc in hexanes) as a yellow oil (1.13 g, 96% yield). **^{1}H NMR** (400 MHz, $CDCl_3$) δ 7.93 – 7.80 (m, 4H), 7.64 (dd, *J* = 8.6, 1.5 Hz, 1H), 7.55 – 7.44 (m, 2H), 7.15 (d, *J* = 8.5 Hz, 2H), 6.87 (d, *J* = 8.5 Hz, 2H), 5.48 (s, 1H), 5.20 (s, 1H), 3.82 (s, 3H), 2.98 – 2.89 (m, 2H), 2.81 (dd, *J* = 9.5, 6.1 Hz, 2H). **^{13}C NMR** (126 MHz, $CDCl_3$) δ 157.9, 147.8, 138.5, 134.2, 133.5, 132.9, 129.5, 128.3, 128.0, 127.7, 126.3, 126.0, 124.9, 124.8, 113.9, 113.4, 55.4, 37.7, 34.0. **IR** (ATR): 3055, 2932, 2833, 1611, 1511, 1299, 1242, 1177, 1036, 890, 858, 818 cm^{-1}. **HRMS** calculated for $C_{21}H_{20}O$ $[M]^+$ 288.1514, found 288.1526.

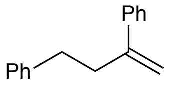

but-3-ene-1,3-diyldibenzene (s1i): The title compound was prepared using the general procedure for the Wittig olefination from 1,3-diphenylpropan-1-one (1.40 g, 6.68 mmol, 1 equiv), methyltriphenylphosphonium bromide (3.58 g, 10.0 mmol, 1.5 equiv), KO*t*-Bu (1.12 g, 10.0 mmol, 1.5 equiv), and THF (26.7 mL, 0.25 M). Isolated by column chromatography (hexanes) as a colorless oil (1.34 g, 96% yield). The ^{1}H NMR was in accordance with the literature[10]. **^{1}H NMR** (400 MHz, $CDCl_3$) δ 7.49 – 7.44 (m, 2H), 7.41 – 7.35 (m, 2H), 7.34 – 7.28 (m, 3H), 7.25 – 7.18 (m, 3H), 5.33 (d, *J* = 1.4 Hz, 1H), 5.10 (d, *J* = 1.3 Hz, 1H), 2.88 – 2.82 (m, 2H), 2.82 – 2.77 (m, 2H).

General Procedure for the Alkene Cyclopropanation

To a round bottom flask containing the 1,1-disubstituted alkene **s1** (1 equiv) was added cetrimonium bromide (2.0 mol%) and $CHBr_3$ (2.0 equiv). While stirring, a 50% aqueous solution of NaOH (1.3 M) was added dropwise. The resulting solution was vigorously stirred for 24 h at rt. The reaction mixture was quenched with water and extracted with CH_2Cl_2. The combined organic layers were washed with brine, dried with anhydrous Na_2SO_4, and concentrated *in*

vacuo. The residue was purified by column chromatography to afford the pure dibromocyclopropane **s2**.

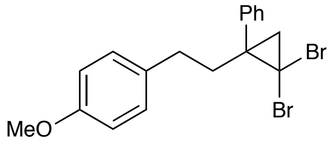

1-(2-(2,2-dibromo-1-phenylcyclopropyl)ethyl)-4-methoxybenzene (s2a): The title compound was prepared using the general procedure for the alkene cyclopropanation from 1,1-disubstituted alkene **s1a** (1.12 g, 4.69 mmol, 1 equiv), cetrimonium bromide (34.2 mg, 0.094 mmol, 2.0 mol%), $CHBr_3$ (0.82 mL, 9.39 mmol, 2.0 equiv), and 50% aqueous NaOH (3.5 mL, 1.3 M). Isolated by column chromatography (5% EtOAc in hexanes) as a yellow oil (1.78 g, 92% yield). **^{1}H NMR** (400 MHz, $CDCl_3$) δ 7.44 – 7.37 (m, 2H), 7.37 – 7.29 (m, 3H), 7.00 (d, *J* = 8.6 Hz, 2H), 6.80 (d, *J* = 8.7 Hz, 2H), 3.78 (s, 3H), 2.56 – 2.36 (m, 3H), 2.10 (dd, *J* = 7.6, 1.0 Hz, 1H), 2.07 – 1.97 (m, 1H), 1.73 (d, *J* = 7.6 Hz, 1H). **^{13}C NMR** (101 MHz, $CDCl_3$) δ 158.0, 140.4, 133.5, 129.6, 129.4, 128.5, 127.6, 113.9, 55.4, 42.8, 39.8, 36.4, 33.2, 32.6. **IR** (ATR): 3026, 2952, 2833, 1610, 1511, 1445, 1300, 1243, 1176, 1034, 820, 751 cm^{-1}. **HRMS** calculated for $C_{18}H_{18}Br_2O$ $[M]^+$ 407.9724, found 407.9727.

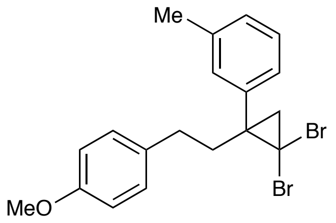

1-(2,2-dibromo-1-(4-methoxyphenethyl)cyclopropyl)-3-methylbenzene (s2b): The title compound was prepared using the general procedure for the alkene cyclopropanation from 1,1-disubstituted alkene **s1b** (505 mg, 2.0 mmol, 1 equiv), cetrimonium bromide (14.6 mg, 0.040 mmol, 2.0 mol%), $CHBr_3$ (0.35 mL, 4.0 mmol, 2.0 equiv), and 50% aqueous NaOH (1.5 mL, 1.3 M). Isolated by column chromatography (5% EtOAc in hexanes) as a yellow oil (622 mg, 73% yield). **^{1}H NMR** (400 MHz, $CDCl_3$) δ 7.30 (d, *J* = 7.8 Hz, 1H), 7.14 (d, *J* = 14.2 Hz, 3H), 7.01 (d, *J* = 8.5 Hz, 2H), 6.80 (d, *J* = 8.5 Hz, 2H), 3.78 (s, 3H), 2.50 (tdd, *J* = 20.6, 14.5, 6.3 Hz, 3H), 2.41 (s, 3H), 2.08 (d, *J* = 7.6 Hz, 1H), 2.06 – 1.96 (m, 1H), 1.72 (d, *J* = 7.6 Hz, 1H). **^{13}C NMR** (101 MHz, $CDCl_3$) δ 158.0, 140.2, 138.1, 133.6, 130.2, 129.4, 128.32, 128.28, 126.6, 113.9, 55.4, 42.8, 39.8, 36.7, 33.2, 32.6, 21.7. **IR** (ATR): 2952, 2833, 1609, 1511, 1453, 1300, 1243, 1176, 1035, 821, 788 cm^{-1}. **HRMS** calculated for $C_{19}H_{20}Br_2O$ $[M]^+$ 421.9881, found 421.9893.

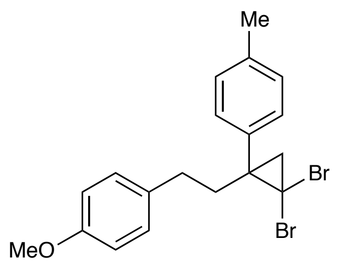

1-(2,2-dibromo-1-(4-methoxyphenethyl)cyclopropyl)-4-methylbenzene (s2c): The title compound was prepared using the general procedure for the alkene cyclopropanation from 1,1-disubstituted alkene **s1c** (685 mg, 2.71 mmol, 1 equiv), cetrimonium bromide (19.8 mg, 0.054 mmol, 2.0 mol%), $CHBr_3$ (0.47 mL, 5.43 mmol, 2.0 equiv), and 50% aqueous NaOH (2.0 mL, 1.3 M). Isolated by column chromatography (5% EtOAc in hexanes) as a yellow oil (1.01 g, 88% yield). **^{1}H NMR** (400 MHz, CD_2Cl_2) δ 7.23 (s, 4H), 7.00 (d, *J* = 8.7 Hz, 2H), 6.79 (d, *J* = 8.7 Hz, 2H), 2.52 – 2.41 (m, 3H), 2.39 (s, 3H), 2.09 (dd, *J* = 7.7, 1.3 Hz, 1H), 2.05 – 1.96 (m, 1H), 1.74 (d, *J* = 7.7 Hz, 1H). **^{13}C NMR** (101 MHz, CD_2Cl_2) δ 158.8, 138.02, 137.98, 134.3, 130.1, 130.0, 129.8, 114.5, 56.0, 43.4, 40.2, 37.8, 33.8, 33.2, 21.7. **IR** (ATR): 2952, 2833, 1611, 1511, 1454, 1300, 1243, 1176, 1035, 818, 689 cm^{-1}. **HRMS** calculated for $C_{19}H_{20}Br_2O$ $[M]^+$ 421.9881, found 421.9884.

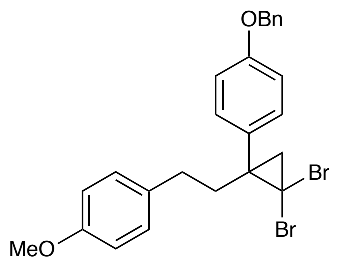

1-(benzyloxy)-4-(2,2-dibromo-1-(4-methoxyphenethyl)cyclopropyl)benzene (s2d): The title compound was prepared using the general procedure for the alkene cyclopropanation from 1,1-disubstituted alkene **s1d** (704 mg, 2.04 mmol, 1 equiv), cetrimonium bromide (14.9 mg, 0.041 mmol, 2.0 mol%), $CHBr_3$ (0.36 mL, 4.09 mmol, 2.0 equiv), and 50% aqueous NaOH (1.5 mL, 1.3 M). Isolated by column chromatography (5% EtOAc in hexanes) as a yellow oil (780 mg, 74% yield). **^{1}H NMR** (400 MHz, $CDCl_3$) δ 7.49 – 7.45 (m, 2H), 7.42 (ddd, *J* = 6.4, 2.6, 0.9 Hz, 2H), 7.36 (ddd, *J* = 7.0, 3.7, 1.5 Hz, 1H), 7.24 (d, *J* = 8.8 Hz, 2H), 7.04 – 6.98 (m, 4H), 6.80 (d, *J* = 8.7 Hz, 2H), 5.09 (s, 2H), 3.79 (s, 3H), 2.47 (qdd, *J* = 13.9, 11.8, 4.0 Hz, 3H), 2.08 – 1.96 (m, 2H), 1.70 (d, *J* = 7.6 Hz, 1H). **^{13}C NMR** (126 MHz, $CDCl_3$) δ 158.2, 158.0, 137.0, 133.5, 132.8, 130.6, 129.4, 128.8, 128.2, 127.8, 114.7, 113.9, 70.2, 55.4, 42.7, 39.2, 37.1, 33.3, 32.6. **IR** (ATR): 3032, 2951, 1608, 1510, 1453, 1299, 1241, 1175, 1034, 828 cm^{-1}. The title compound was unstable under HRMS conditions.

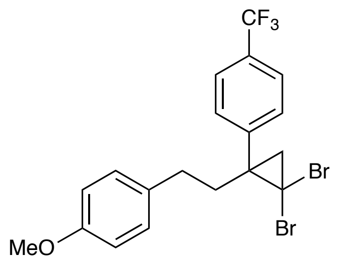

1-(2-(2,2-dibromo-1-(4-(trifluoromethyl)phenyl)cyclopropyl)ethyl)-4-methoxybenzene (s2e): The title compound was prepared using the general procedure for the alkene cyclopropanation from 1,1-disubstituted alkene **s1e** (800 mg, 2.61 mmol, 1 equiv), cetrimonium bromide

(19.0 mg, 0.052 mmol, 2.0 mol%), $CHBr_3$ (0.46 mL, 5.22 mmol, 2.0 equiv), and 50% aqueous NaOH (2.0 mL, 1.3 M). Isolated by column chromatography (5% EtOAc in hexanes) as a yellow oil (989 mg, 79% yield). **^{1}H NMR** (400 MHz, $CDCl_3$) δ 7.67 (d, *J* = 8.1 Hz, 2H), 7.44 (d, *J* = 8.0 Hz, 2H), 6.99 (d, *J* = 8.5 Hz, 2H), 6.80 (d, *J* = 8.5 Hz, 2H), 3.78 (s, 3H), 2.56 – 2.39 (m, 3H), 2.13 – 2.01 (m, 2H), 1.79 (d, *J* = 7.7 Hz, 1H). **^{13}C NMR** (101 MHz, $CDCl_3$) δ 158.1, 144.4 (q, $J_{C\text{-}F}$ = 1.3 Hz), 132.9, 130.0, 129.8 (q, $J_{C\text{-}F}$ = 32.5 Hz), 129.3, 125.5 (q, $J_{C\text{-}F}$ = 3.8 Hz), 124.2 (q, $J_{C\text{-}F}$ = 272.3 Hz), 114.0, 55.4, 42.6, 39.6, 34.9, 33.3, 32.5. **^{19}F NMR** (376 MHz, $CDCl_3$) δ -62.9. **IR** (ATR): 2955, 2835, 1616, 1511, 1322, 1301, 1244, 1163, 1113, 1065, 1035, 1016, 842 cm^{-1}. **HRMS** calculated for $C_{19}H_{17}Br_2F_3O$ $[M]^+$ 475.9598, found 475.9594.

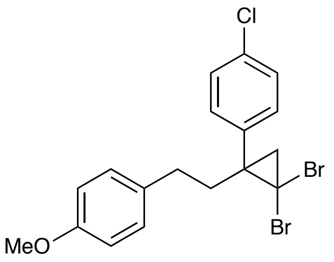

1-chloro-4-(2,2-dibromo-1-(4-methoxyphenethyl)cyclopropyl)benzene (s2f): The title compound was prepared using the general procedure for the alkene cyclopropanation from 1,1-disubstituted alkene **s1f** (467 mg, 1.71 mmol, 1 equiv), cetrimonium bromide (12.5 mg, 0.034 mmol, 2.0 mol%), $CHBr_3$ (0.30 mL, 3.43 mmol, 2.0 equiv), and 50% aqueous NaOH (1.3 mL, 1.3 M). Isolated by column chromatography (5% EtOAc in hexanes) as a yellow oil (560 mg, 73% yield). **^{1}H NMR** (400 MHz, $CDCl_3$) δ 7.38 (d, *J* = 8.2 Hz, 2H), 7.25 (d, *J* = 8.4 Hz, 2H), 6.99 (d, *J* = 8.5 Hz, 2H), 6.80 (d, *J* = 8.4 Hz, 2H), 3.78 (s, 3H), 2.59 – 2.32 (m, 3H), 2.08 – 1.97 (m, 2H), 1.74 (d, *J* = 7.7 Hz, 1H). **^{13}C NMR** (126 MHz, $CDCl_3$) δ 158.1, 138.9, 133.4, 133.1, 130.9, 129.3, 128.8, 114.0, 55.4, 42.6, 39.2, 35.7, 33.3, 32.5. **IR** (ATR): 2953, 2833, 1611, 1511, 1492, 1300, 1243, 1176, 1087, 1034, 1013, 825 cm^{-1}. **HRMS** calculated for $C_{18}H_{17}Br_2ClO$ $[M]^+$ 441.9335, found 441.9333.

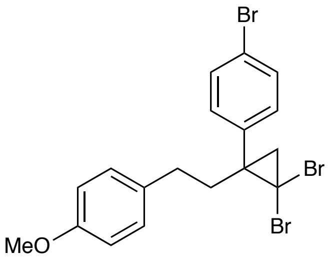

1-bromo-4-(2,2-dibromo-1-(4-methoxyphenethyl)cyclopropyl)benzene (s2g): The title compound was prepared using the general procedure for the alkene cyclopropanation from 1,1-disubstituted alkene **s1g** (534 mg, 1.69 mmol, 1 equiv), cetrimonium bromide (12.3 mg, 0.034 mmol, 2.0 mol%), $CHBr_3$ (0.29 mL, 3.37 mmol, 2.0 equiv), and 50% aqueous NaOH (1.3 mL, 1.3 M). Isolated by column chromatography (5% EtOAc in hexanes) as a yellow oil (675 mg, 82% yield). **^{1}H NMR** (400 MHz, $CDCl_3$) δ 7.53 (d, *J* = 7.7 Hz, 2H), 7.19 (d, *J* = 7.9 Hz, 2H), 6.99 (d, *J* = 8.3 Hz, 2H), 6.80 (d, *J* = 8.1 Hz, 2H), 3.78 (s, 3H), 2.58 – 2.32 (m, 3H),

2.02 (dd, J = 17.1, 6.7 Hz, 2H), 1.73 (d, J = 7.7 Hz, 1H). **^{13}C NMR** (126 MHz, $CDCl_3$) δ 158.1, 139.4, 133.1, 131.7, 131.3, 129.3, 121.6, 114.0, 55.4, 42.6, 39.3, 35.5, 33.3, 32.5. **IR** (ATR): 2952, 2833, 1610, 1511, 1489, 1300, 1243, 1177, 1070, 1034, 1009, 821 cm^{-1}. **HRMS** calculated for $C_{18}H_{17}Br_3O$ $[M]^+$ 485.8829, found 485.8834.

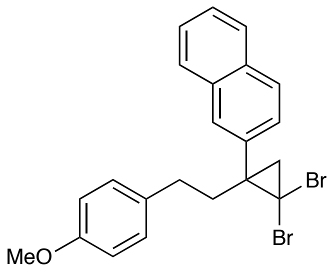

2-(2,2-dibromo-1-(4-methoxyphenethyl)cyclopropyl)naphthalene (s2h): The title compound was prepared using the general procedure for the alkene cyclopropanation from 1,1-disubstituted alkene **s1h** (1.10 g, 3.81 mmol, 1 equiv), cetrimonium bromide (27.8 mg, 0.076 mmol, 2.0 mol%), $CHBr_3$ (0.67 mL, 7.62 mmol, 2.0 equiv), and 50% aqueous NaOH (2.8 mL, 1.3 M). Isolated by column chromatography (5% EtOAc in hexanes) as a yellow oil (1.27 g, 73% yield). **^{1}H NMR** (400 MHz, $CDCl_3$) δ 7.94 – 7.83 (m, 3H), 7.70 (d, J = 1.7 Hz, 1H), 7.56 – 7.49 (m, 3H), 6.99 (d, J = 8.6 Hz, 2H), 6.79 (d, J = 8.6 Hz, 2H), 3.77 (s, 3H), 2.61 – 2.47 (m, 3H), 2.24 (dd, J = 7.6, 1.1 Hz, 1H), 2.17 – 2.07 (m, 1H), 1.82 (d, J = 7.6 Hz, 1H). **^{13}C NMR** (101 MHz, $CDCl_3$) δ 158.0, 138.0, 133.4, 133.3, 132.8, 129.4, 128.4, 128.3, 128.0, 127.9, 127.4, 126.4, 126.3, 113.9, 55.4, 42.6, 40.0, 36.3, 33.3, 32.7. **IR** (ATR): 2952, 2832, 1610, 1511, 1453, 1300, 1242, 1176, 1034, 818 cm^{-1}. **HRMS** calculated for $C_{22}H_{20}Br_2O$ $[M]^+$ 459.9862, found 459.9844.

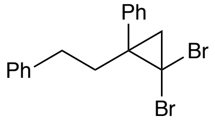

(2,2-dibromo-1-phenethylcyclopropyl)benzene (s2i): The title compound was prepared using the general procedure for the alkene cyclopropanation from 1,1-disubstituted alkene **s1i** (1.32 g, 6.36 mmol, 1 equiv), cetrimonium bromide (46.3 mg, 0.127 mmol, 2.0 mol%), $CHBr_3$ (1.1 mL, 12.7 mmol, 2.0 equiv), and 50% aqueous NaOH (4.7 mL, 1.3 M). Isolated by column chromatography (hexanes) as a yellow oil (2.21 g, 91% yield). **^{1}H NMR** (400 MHz, $CDCl_3$) δ 7.46 – 7.38 (m, 2H), 7.38 – 7.31 (m, 3H), 7.29 – 7.22 (m, 2H), 7.21 – 7.15 (m, 1H), 7.12 – 7.06 (m, 2H), 2.66 – 2.53 (m, 2H), 2.53 – 2.44 (m, 1H), 2.16 – 2.04 (m, 2H), 1.75 (d, J = 7.6 Hz, 1H). **^{13}C NMR** (101 MHz, $CDCl_3$) δ 141.4, 140.3, 129.6, 128.51, 128.50, 128.5, 127.6, 126.1, 42.6, 39.9, 36.3, 33.5, 33.2. **IR** (ATR): 3026, 2926, 1602, 1495, 1447, 1101, 1055, 1021, 1003, 777, 762, 744 cm^{-1}. **HRMS** calculated for $C_{17}H_{16}Br_2NH_4$ $[M+ NH_4]^+$ 395.9962, found 395.9979.

General Procedure for the Skattebøl Rearrangement

To a flame-dried round bottom flask was added dibromocyclopropane **s2** (1 equiv) and THF (0.5 M). EtMgBr (1.7 equiv, 1.0 M in THF) was added dropwise, and the resulting mixture was stirred for 1 h at rt. The reaction mixture was quenched with water and extracted with Et_2O. The combined organic layers were washed with brine, dried with anhydrous Na_2SO_4, and concentrated *in vacuo*. The residue was purified by column chromatography to afford the desired allene **1**.

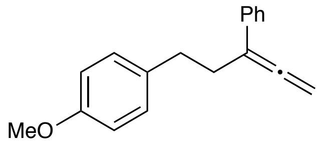

1-methoxy-4-(3-phenylpenta-3,4-dien-1-yl)benzene (1a): The title compound was prepared using the general procedure for the Skattebøl rearrangement from dibromocyclopropane **s2a** (1.75 g, 4.27 mmol, 1 equiv), EtMgBr (7.3 mL, 7.3 mmol, 1.7 equiv, 1.0 M in THF), and THF (8.5 mL, 0.50 M). Isolated by column chromatography (10% CH_2Cl_2 in hexanes) as a yellow oil (920 mg, 86% yield). **^{1}H NMR** (500 MHz, CD_2Cl_2) δ 7.44 (d, *J* = 7.7 Hz, 2H), 7.39 – 7.30 (m, 2H), 7.23 (t, *J* = 7.4 Hz, 1H), 7.17 (d, *J* = 6.8 Hz, 2H), 6.85 (d, *J* = 6.8 Hz, 2H), 5.10 (d, *J* = 3.0 Hz, 2H), 3.79 (s, 3H), 2.88 – 2.78 (m, 2H), 2.77 – 2.66 (m, 2H). **^{13}C NMR** (126 MHz, CD_2Cl_2) δ 209.4, 158.8, 137.1, 134.9, 130.2, 129.2, 127.4, 126.7, 114.5, 105.3, 79.2, 56.0, 34.1, 32.4. **IR** (ATR): 2931, 1939, 1611, 1511, 1493, 1450, 1300, 1243, 1176, 1035, 820 cm^{-1}. **HRMS** calculated for $C_{18}H_{17}O$ $[M-H]^-$ 249.1279, found 249.1273.

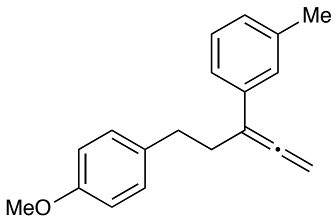

1-(5-(4-methoxyphenyl)penta-1,2-dien-3-yl)-3-methylbenzene (1c): The title compound was prepared using the general procedure for the Skattebøl rearrangement from dibromocyclopropane **s2b** (593 mg, 1.40 mmol, 1 equiv), EtMgBr (2.4 mL, 2.4 mmol, 1.7 equiv, 1.0 M in THF), and THF (2.8 mL, 0.50 M). Isolated by column chromatography (10% CH_2Cl_2 in hexanes) as a yellow oil (327 mg, 88% yield). **^{1}H NMR** (400 MHz, CD_2Cl_2) δ 7.30 – 7.22 (m, 3H), 7.18 (d, *J* = 8.8 Hz, 2H), 7.09 – 7.03 (m, 1H), 6.86 (d, *J* = 8.6 Hz, 2H), 5.10 (t, *J* = 3.3 Hz, 2H), 3.80 (s, 3H), 2.82 (dd, *J* = 9.3, 5.9 Hz, 2H), 2.70 (ttd, *J* = 8.4, 3.3, 1.0 Hz, 2H), 2.37 (s, 3H). **^{13}C NMR** (101 MHz, CD_2Cl_2) δ 209.4, 158.8, 138.8, 137.0, 135.0, 130.2, 129.1, 128.2, 127.5, 123.8, 114.5, 105.4, 79.1, 56.0, 34.1, 32.5, 22.0. **IR** (ATR): 2930, 2833, 1938, 1610, 1511, 1441, 1300, 1243, 1176, 1036, 851, 821, 786 cm^{-1}. **HRMS** calculated for $C_{19}H_{20}O$ $[M]^+$ 264.1514, found 264.1517.

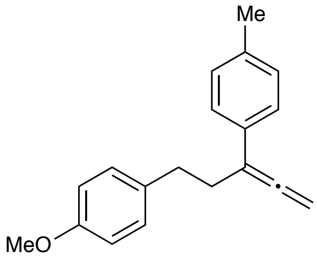

1-methoxy-4-(3-(*p*-tolyl)penta-3,4-dien-1-yl)benzene (1d): The title compound was prepared using the general procedure for the Skattebøl rearrangement from dibromocyclopropane **s2c** (509 mg, 1.20 mmol, 1 equiv), EtMgBr (2.0 mL, 2.0 mmol, 1.7 equiv, 1.0 M in THF), and THF (2.4 mL, 0.50 M). Isolated by column chromatography (10% CH_2Cl_2 in hexanes) as a yellow oil (264 mg, 83% yield). **^{1}H NMR** (400 MHz, CD_2Cl_2) δ 7.33 (d, *J* = 8.1 Hz, 2H), 7.23 – 7.11 (m, 4H), 6.85 (d, *J* = 8.6 Hz, 2H), 5.08 (t, *J* = 3.3 Hz, 2H), 3.80 (s, 3H), 2.81 (dd, *J* = 9.6, 6.1 Hz, 2H), 2.73 – 2.64 (m, 2H), 2.35 (s, 3H). **^{13}C NMR** (101 MHz, CD_2Cl_2) δ 209.2, 158.8, 137.3, 135.0, 134.0, 130.2, 129.9, 126.6, 114.4, 105.2, 79.0, 56.0, 34.1, 32.5, 21.6. **IR** (ATR): 2930, 2833, 1940, 1611, 1510, 1440, 1300, 1244, 1176, 1036, 849, 817 cm^{-1}. **HRMS** calculated for $C_{19}H_{20}OH$ $[M+H]^+$ 265.1592, found 265.1585.

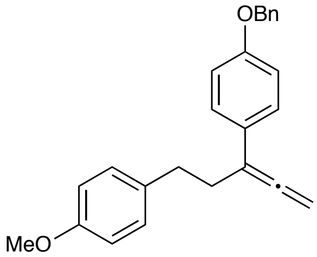

1-(benzyloxy)-4-(5-(4-methoxyphenyl)penta-1,2-dien-3-yl)benzene (1e): The title compound was prepared using the general procedure for the Skattebøl rearrangement from dibromocyclopropane **s2d** (757 mg, 1.47 mmol, 1 equiv), EtMgBr (2.5 mL, 2.5 mmol, 1.7 equiv, 1.0 M in THF), and THF (2.9 mL, 0.50 M). Isolated by column chromatography (10% CH_2Cl_2 in hexanes) as a yellow solid (393 mg, 75% yield). **^{1}H NMR** (400 MHz, CD_2Cl_2) δ 7.47 – 7.43 (m, 2H), 7.40 (ddd, *J* = 7.8, 6.8, 1.0 Hz, 2H), 7.37 – 7.33 (m, 3H), 7.18 – 7.13 (m, 2H), 6.95 (d, *J* = 8.9 Hz, 2H), 6.84 (d, *J* = 8.7 Hz, 2H), 5.08 – 5.05 (m, 4H), 3.78 (s, 3H), 2.79 (dd, *J* = 9.7, 6.4 Hz, 2H), 2.71 – 2.59 (m, 2H). **^{13}C NMR** (101 MHz, CD_2Cl_2) δ 209.1, 158.5, 138.0, 135.0, 130.1, 129.5, 129.3, 128.7, 128.3, 127.8, 124.6, 115.6, 114.4, 104.8, 79.1, 70.8, 56.0, 34.0, 32.6. **IR** (ATR): 2936, 2837, 1939, 1608, 1510, 1465, 1383, 1240, 1177, 1037, 999, 859, 837 cm^{-1}. **HRMS** calculated for $C_{25}H_{24}O_2$ $[M]^+$ 356.1776, found 356.1778.

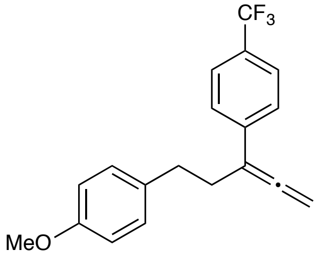

1-methoxy-4-(3-(4-(trifluoromethyl)phenyl)penta-3,4-dien-1-yl)benzene (1f): The title compound was prepared using the general procedure for the Skattebøl rearrangement from dibromocyclopropane **s2e** (937 mg, 1.96 mmol, 1 equiv), EtMgBr (3.3 mL, 3.3 mmol, 1.7 equiv, 1.0 M in THF), and THF (3.9 mL, 0.50 M). Isolated by column chromatography (10% CH_2Cl_2 in hexanes) as a

colorless oil (618 mg, 99% yield). ^{1}**H NMR** (400 MHz, CD_2Cl_2) δ 7.60 (d, *J* = 8.5 Hz, 2H), 7.55 (d, *J* = 8.5 Hz, 2H), 7.16 (d, *J* = 8.8 Hz, 2H), 6.85 (d, *J* = 8.6 Hz, 2H), 5.17 (d, *J* = 3.3 Hz, 2H), 3.79 (s, 3H), 2.87 – 2.79 (m, 2H), 2.75 – 2.65 (m, 2H). ^{13}**C NMR** (101 MHz, CD_2Cl_2) δ 210.0, 158.9, 141.3 (q, $J_{C\text{-}F}$ = 1.5 Hz), 134.6, 130.2, 129.1 (q, $J_{C\text{-}F}$ = 32.3 Hz), 127.0, 126.05 (q, $J_{C\text{-}F}$ = 3.9 Hz), 125.3 (q, $J_{C\text{-}F}$ = 271.8 Hz), 114.5, 104.7, 79.9, 56.0, 33.9, 32.2. ^{19}**F NMR** (376 MHz, CD_2Cl_2) δ -62.67. **IR** (ATR): 2934, 1939, 1614, 1512, 1324, 1301, 1245, 1163, 1110, 1068, 1036, 1015, 842, 821 cm^{-1}. **HRMS** calculated for $C_{19}H_{17}F_3OH$ $[M+H]^+$ 319.1310, found 319.1320.

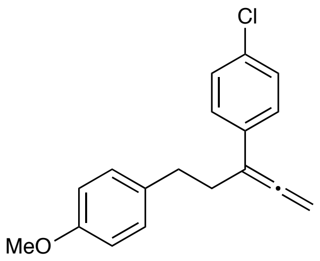

1-chloro-4-(5-(4-methoxyphenyl)penta-1,2-dien-3-yl)benzene (1g): The title compound was prepared using the general procedure for the Skattebøl rearrangement from dibromocyclopropane **s2f** (526 mg, 1.18 mmol, 1 equiv), EtMgBr (2.0 mL, 2.0 mmol, 1.7 equiv, 1.0 M in THF), and THF (2.4 mL, 0.50 M). Isolated by column chromatography (10% CH_2Cl_2 in hexanes) as a yellow oil (278 mg, 83% yield). ^{1}**H NMR** (400 MHz, CD_2Cl_2) δ 7.38 (d, *J* = 8.6 Hz, 2H), 7.31 (d, *J* = 8.7 Hz, 2H), 7.18 – 7.13 (m, 2H), 6.85 (d, *J* = 8.6 Hz, 2H), 5.11 (t, *J* = 3.3 Hz, 2H), 3.79 (s, 3H), 2.84 – 2.76 (m, 2H), 2.72 – 2.61 (m, 2H). ^{13}**C NMR** (101 MHz, CD_2Cl_2) δ 209.4, 158.8, 135.8, 134.7, 133.0, 130.1, 129.3, 128.1, 114.5, 104.6, 79.7, 56.0, 33.9, 32.3. **IR** (ATR): 2954, 2912, 1937, 1611, 1511, 1491, 1300, 1244, 1178, 1091, 1031, 1010, 859, 832 cm^{-1}. **HRMS** calculated for $C_{18}H_{17}ClONH_4$ $[M+NH_4]^+$ 302.1312, found 302.1313.

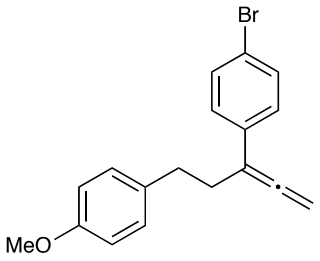

1-bromo-4-(5-(4-methoxyphenyl)penta-1,2-dien-3-yl)benzene (1h): The title compound was prepared using the general procedure for the Skattebøl rearrangement from dibromocyclopropane **s2g** (653 mg, 1.34 mmol, 1 equiv), EtMgBr (2.3 mL, 2.3 mmol, 1.7 equiv, 1.0 M in THF), and THF (2.7 mL, 0.50 M). Isolated by column chromatography (10% CH_2Cl_2 in hexanes) as a yellow oil (376 mg, 85% yield). ^{1}**H NMR** (400 MHz, CD_2Cl_2) δ 7.46 (d, *J* = 8.6 Hz, 2H), 7.31 (d, *J* = 8.6 Hz, 2H), 7.15 (d, *J* = 8.6 Hz, 2H), 6.84 (d, *J* = 8.6 Hz, 2H), 5.10 (t, *J* = 3.3 Hz, 2H), 3.79 (s, 3H), 2.80 (dd, *J* = 9.2, 5.9 Hz, 2H), 2.70 – 2.62 (m, 2H). ^{13}**C NMR** (101 MHz, CD_2Cl_2) δ 209.4, 158.8, 136.3, 134.7, 132.2, 130.1, 128.4, 121.1, 114.5, 104.6, 79.7, 56.0, 33.9, 32.3. **IR** (ATR): 2933, 1934, 1611, 1511, 1487, 1300, 1241, 1175, 1032, 1005, 950, 863 cm^{-1}. **HRMS** calculated for $C_{18}H_{17}BrO$ $[M]^+$ 328.0463, found 328.0458.

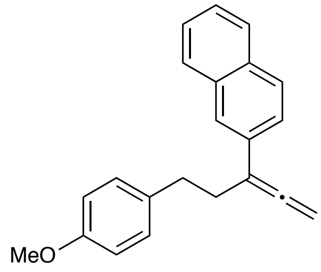

2-(5-(4-methoxyphenyl)penta-1,2-dien-3-yl)naphthalene (1i): The title compound was prepared using the general procedure for the Skattebøl rearrangement from dibromocyclopropane **s2h** (1.25 g, 2.71 mmol, 1 equiv), EtMgBr (4.6 mL, 4.6 mmol, 1.7 equiv, 1.0 M in THF), and THF (5.4 mL, 0.50 M). Isolated by column chromatography (10% CH_2Cl_2 in hexanes) as a yellow oil (807 mg, 99% yield). **^{1}H NMR** (500 MHz, CD_2Cl_2) δ 7.82 (t, *J* = 10.0 Hz, 4H), 7.66 (dd, *J* = 8.6, 1.8 Hz, 1H), 7.53 – 7.44 (m, 2H), 7.21 (d, *J* = 8.6 Hz, 2H), 6.88 (d, *J* = 8.4 Hz, 2H), 5.20 (t, *J* = 3.2 Hz, 2H), 3.80 (s, 3H), 2.90 (ddd, *J* = 8.9, 6.3, 1.9 Hz, 2H), 2.87 – 2.78 (m, 2H). **^{13}C NMR** (126 MHz, CD_2Cl_2) δ 210.1, 158.8, 135.0, 134.45, 134.44, 133.2, 130.2, 128.7, 128.6, 128.3, 126.9, 126.5, 126.0, 124.3, 114.5, 105.6, 79.7, 56.0, 34.1, 32.4. **IR** (ATR): 3055, 2931, 2833, 1936, 1611, 1511, 1300, 1243, 1177, 1036, 854, 817, 747 cm^{-1}. **HRMS** calculated for $C_{22}H_{20}O$ $[M]^+$ 300.1514, found 300.1521.

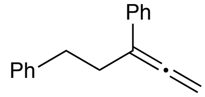

penta-3,4-diene-1,3-diyldibenzene (1n): The title compound was prepared using the general procedure for the Skattebøl rearrangement from dibromocyclopropane **s2i** (2.18 g, 5.73 mmol, 1 equiv), EtMgBr (9.7 mL, 9.7 mmol, 1.7 equiv, 1.0 M in THF), and THF (11.5 mL, 0.50 M). Isolated by column chromatography (hexanes) as a colorless oil (1.19 g, 94% yield). **^{1}H NMR** (400 MHz, CD_2Cl_2) δ 7.48 – 7.42 (m, 2H), 7.38 – 7.29 (m, 4H), 7.28 – 7.24 (m, 2H), 7.24 – 7.19 (m, 2H), 5.11 (t, *J* = 3.3 Hz, 2H), 2.93 – 2.83 (m, 2H), 2.80 – 2.70 (m, 2H). **^{13}C NMR** (101 MHz, CD_2Cl_2) δ 209.4, 142.9, 137.1, 129.3, 129.2, 129.1, 127.5, 126.73, 126.65, 105.4, 79.3, 35.0, 32.2. **IR** (ATR): 3026, 2924, 1940, 1596, 1494, 1452, 1076, 1029, 850, 758, 723, 693 cm^{-1}. **HRMS** calculated for $C_{17}H_{17}$ $[M+H]^+$ 221.1330, found 221.1325.

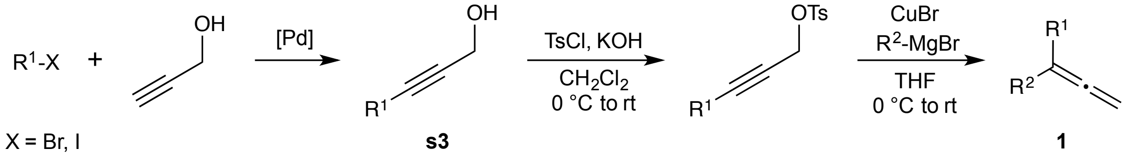

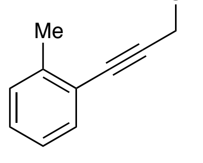

3-(*o*-tolyl)prop-2-yn-1-ol (s3a): Under a gentle flow of nitrogen, an oven-dried round bottom flask equipped with a magnetic stir bar was charged with $Pd(PPh_3)_2Cl_2$ (126 mg, 0.180 mmol, 3.0 mol%), CuI (68.6 mg, 0.360 mmol, 6.0 mol%), toluene (6.0 mL, 1.0 M), piperidine (1.2 mL, 12 mmol, 2.0 equiv), 2-iodotoluene (0.76 mL, 6.0 mmol, 1 equiv), and freshly distilled propargyl alcohol (0.36 mL, 6.24

mmol, 1.04 equiv). The resulting mixture was stirred at rt for 4 h. The reaction mixture was filtered through silica and concentrated *in vacuo*. The residue was purified by column chromatography (20% EtOAc in hexanes) to afford the title compound as a red oil (778 mg, 89% yield). The ^{1}H NMR was in accordance with the literature[11]. **^{1}H NMR** (400 MHz, $CDCl_3$) δ 7.42 (dd, *J* = 7.5, 1.3 Hz, 1H), 7.27 – 7.18 (m, 2H), 7.14 (td, *J* = 7.4, 2.1 Hz, 1H), 4.55 (d, *J* = 6.2 Hz, 2H), 2.44 (s, 3H), 1.65 (s, 1H).

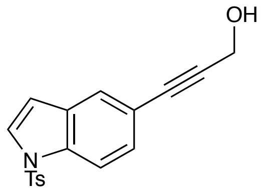

3-(1-tosyl-1*H*-indol-5-yl)prop-2-yn-1-ol (s3b): Under a gentle flow of nitrogen, an oven-dried round bottom flask equipped with a magnetic stir bar was charged with $Pd(PPh_3)_4$ (165 mg, 0.143 mmol, 6.0 mol%), pyrrolidine (6.0 mL, 0.40 M), 5-bromo-1-tosyl-1*H*-indole (835 mg, 2.38 mmol, 1 equiv), and freshly distilled propargyl alcohol (0.21 mL, 3.58 mmol, 1.50 equiv). The resulting mixture was stirred overnight at 50 °C. The reaction mixture was cooled to rt and concentrated *in vacuo*. The residue was purified by column chromatography (30% EtOAc in hexanes) to afford the title compound as a yellow oil (702 mg, 91% yield). **^{1}H NMR** (400 MHz, $CDCl_3$) δ 7.92 (d, *J* = 8.6 Hz, 1H), 7.74 (d, *J* = 8.4 Hz, 2H), 7.58 (dd, *J* = 12.2, 2.6 Hz, 2H), 7.37 (dd, *J* = 8.6, 1.6 Hz, 1H), 7.21 (d, *J* = 8.1 Hz, 2H), 6.59 (dd, *J* = 3.7, 0.8 Hz, 1H), 4.50 (s, 2H), 2.32 (s, 3H), 2.05 (s, 1H). **^{13}C NMR** (101 MHz, $CDCl_3$) δ 145.3, 135.1, 134.5, 130.8, 130.0, 128.2, 127.3, 126.9, 125.1, 117.6, 113.6, 108.9, 86.6, 85.8, 51.7, 21.7. **IR** (ATR): 3370, 2924, 1595, 1455, 1370, 1288, 1173, 1158, 1091, 1024, 995, 894, 725 cm^{-1}. **HRMS** calculated for $C_{18}H_{15}NO_3SNa$ $[M+Na]^+$ 348.0670, found 348.0685.

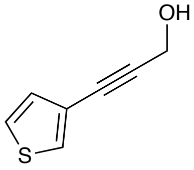

3-(thiophen-3-yl)prop-2-yn-1-ol (s3c): Under a gentle flow of nitrogen, an oven-dried round bottom flask equipped with a magnetic stir bar was charged with $Pd(PPh_3)_2Cl_2$ (126 mg, 0.180 mmol, 3.0 mol%), CuI (68.6 mg, 0.360 mmol, 6.0 mol%), Et_3N (6.0 mL, 1.0 M), 3-bromothiophene (0.56 mL, 6.0 mmol, 1 equiv), and freshly distilled propargyl alcohol (0.70 mL, 12.0 mmol, 2.0 equiv). The resulting mixture was stirred at 70 °C for 12 h. The reaction mixture was filtered through celite and concentrated *in vacuo*. The residue was purified by column chromatography (20% EtOAc in hexanes) to afford the title compound as a yellow oil (427 mg, 52% yield). The ^{1}H NMR was in accordance with the literature[12]. **^{1}H NMR** (400 MHz, $CDCl_3$) δ 7.46 (d, *J* = 3.0 Hz, 1H), 7.29 – 7.27 (m, 1H), 7.12 (dd, *J* = 4.9, 1.1 Hz, 1H), 4.49 (d, *J* = 5.0 Hz, 2H), 1.62 (s, 1H).

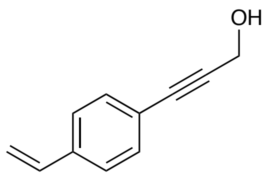

3-(4-vinylphenyl)prop-2-yn-1-ol (s3d): Under a gentle flow of nitrogen, an oven-dried round bottom flask equipped with a magnetic stir bar was charged with $Pd(OAc)_2$ (13.5 mg, 0.060 mmol, 1.0 mol%), PPh_3 (47.2 mg, 0.18 mmol, 3.0 mol%), CuI (11.4 mg, 0.060 mmol, 1.0 mol%), Et_3N (12.0 mL, 0.50 M), 4-bromostyrene (0.78 mL, 6.0 mmol, 1 equiv), and freshly distilled propargyl alcohol (0.35 mL, 6.0 mmol, 1.0 equiv). The resulting mixture was stirred overnight at 80 °C. The reaction mixture was cooled to rt, filtered through celite, and concentrated *in vacuo*. The residue was purified by column chromatography (20% EtOAc in hexanes) to afford the title compound as a yellow oil (366 mg, 39% yield). **^{1}H NMR** (400 MHz, $CDCl_3$) δ 7.41 (d, J = 8.5 Hz, 2H), 7.36 (d, J = 8.4 Hz, 2H), 6.70 (dd, J = 17.6, 10.9 Hz, 1H), 5.78 (dd, J = 17.6, 0.8 Hz, 1H), 5.30 (dd, J = 10.9, 0.8 Hz, 1H), 4.52 (d, J = 6.0 Hz, 2H), 1.66 (s, 1H). **^{13}C NMR** (101 MHz, $CDCl_3$) δ 137.8, 136.3, 132.0, 126.2, 121.9, 115.0, 88.0, 85.8, 51.8. **IR** (ATR): 3305, 1627, 1507, 1402, 1356, 1262, 1113, 1018, 998, 952, 911, 841 cm^{-1}. **HRMS** calculated for $C_{11}H_{10}O$ $[M]^+$ 158.0732, found 158.0729.

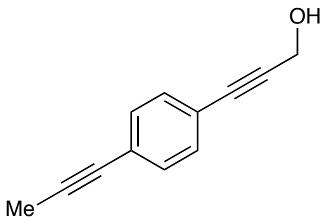

3-(4-(prop-1-yn-1-yl)phenyl)prop-2-yn-1-ol (s3e): Under a gentle flow of nitrogen, an oven-dried round bottom flask equipped with a magnetic stir bar was charged with $Pd(PPh_3)_2Cl_2$ (99.8 mg, 0.142 mmol, 3.0 mol%), CuI (54.1 mg, 0.284 mmol, 6.0 mol%), Et_3N (4.7 mL, 1.0 M), 1-bromo-4-(prop-1-yn-1-yl)benzene (924 mg, 4.7 mmol, 1 equiv), and freshly distilled propargyl alcohol (0.41 mL, 7.1 mmol, 1.5 equiv). The resulting mixture was stirred overnight at 80 °C. The reaction mixture was cooled to rt, filtered through celite, and concentrated *in vacuo*. The residue was purified by column chromatography (20% EtOAc in hexanes) to afford the title compound as a yellow solid (588 mg, 73% yield). **^{1}H NMR** (400 MHz, $CDCl_3$) δ 7.41 – 7.29 (m, 4H), 4.50 (s, 2H), 2.06 (s, 3H), 1.86 (s, 1H). **^{13}C NMR** (101 MHz, $CDCl_3$) δ 131.6, 131.5, 124.4, 121.7, 88.6, 88.1, 85.6, 79.5, 51.8, 4.6. **IR** (ATR): 3317, 2910, 2863, 1506, 1418, 1370, 1259, 1103, 1016, 950, 832 cm^{-1}. **HRMS** calculated for $C_{12}H_{10}O$ $[M]^+$ 170.0732, found 170.0733.

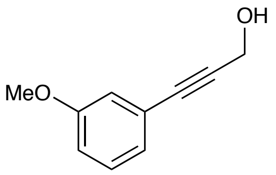

3-(3-methoxyphenyl)prop-2-yn-1-ol (s3f): Under a gentle flow of nitrogen, an oven-dried round bottom flask equipped with a magnetic stir bar was charged with $Pd(PPh_3)_4$ (173 mg, 0.150 mmol, 3.0 mol%), CuI (57.1 mg, 0.300 mmol, 6.0 mol%), Et_3N (10.0 mL, 0.50 M), 3-

bromoanisole (0.63 mL, 5.0 mmol, 1 equiv), and freshly distilled propargyl alcohol (0.35 mL, 6.0 mmol, 1.2 equiv). The resulting mixture was stirred overnight at 80 °C. The reaction mixture was cooled to rt, filtered through celite, and concentrated *in vacuo*. The residue was purified by column chromatography (20% EtOAc in hexanes) to afford the title compound as a yellow oil (652 mg, 80% yield). The ^{1}H NMR was in accordance with the literature[13]. **^{1}H NMR** (400 MHz, $CDCl_3$) δ 7.26 – 7.21 (m, 1H), 7.04 (dt, *J* = 7.6, 1.2 Hz, 1H), 6.98 (dd, *J* = 2.7, 1.4 Hz, 1H), 6.90 (ddd, *J* = 8.3, 2.6, 1.0 Hz, 1H), 4.51 (d, *J* = 6.2 Hz, 2H), 3.81 (s, 3H), 1.67 (t, *J* = 5.8 Hz, 1H).

General Procedure for Alcohol Tosylation

A flame-dried round bottom flask equipped with a magnetic stir bar was charged with alcohol **s3** (1 equiv) and CH_2Cl_2 (0.63 M). The resulting solution was cooled to 0 °C. TsCl (1.20 equiv) was added, followed by freshly crushed KOH (300 mg/mmol of alcohol) portionwise. The resulting mixture was stirred for 1 h at rt. The reaction mixture was poured into ice and extracted with CH_2Cl_2. The combined organic layers were washed with brine, dried with anhydrous Na_2SO_4, and concentrated *in vacuo*. The obtained crude propargyl tosylate was used without further purification.

General Procedure for the Cu-catalysed Nucleophilic Substitution

A flame-dried round bottom flask equipped with a magnetic stir bar was charged with propargyl tosylate (1 equiv), CuBr (10 mol%), and THF (0.50 M). The resulting solution was cooled to 0 °C, and a freshly prepared solution of the Grignard reagent (1.25 equiv, 1 M in THF) was added dropwise. (The Grignard reagent solution was prepared by stirring the appropriate alkyl bromide (1 equiv) in the presence of Mg (1.5 equiv) and a catalytic amount of I_2 in THF (1 M) at rt for 2 h). The resulting mixture was stirred for 2 h at rt. The reaction mixture was quenched with saturated aqueous NH_4Cl and extracted with Et_2O. The combined organic layers were washed with brine, dried with anhydrous Na_2SO_4, and concentrated *in vacuo*. The residue was purified by column chromatography to afford the desired allene **1**.

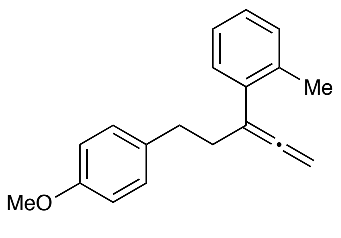

1-(5-(4-methoxyphenyl)penta-1,2-dien-3-yl)-2-methylbenzene (1b): The title compound was prepared according to the general procedures for alcohol tosylation and Cu-catalysed nucleophilic substitution using alcohol **s3a** (332 mg, 2.0 mmol, 1 equiv), TsCl

(458 mg, 2.4 mmol, 1.2 equiv), KOH (600 mg, 300 mg/mmol of alcohol) and CH_2Cl_2 (3.2 mL, 0.63 M). The crude propargyl tosylate was used without further purification.

Crude 3-(*o*-tolyl)prop-2-yn-1-yl 4-methylbenzenesulfonate (601 mg, 2.0 mmol, 1 equiv) was reacted with (4-methoxyphenethyl)magnesium bromide (2.5 mL, 1.25 equiv, 1 M in THF) using CuBr (28.7 mg, 0.20 mmol, 10 mol%) and THF (4.0 mL, 0.50 M). Purification by column chromatography (10% CH_2Cl_2 in hexanes) afforded the desired allene as a yellow oil (89.8 mg, 17% yield). **^{1}H NMR** (400 MHz, CD_2Cl_2) δ 7.25 – 7.16 (m, 4H), 7.13 (d, *J* = 8.8 Hz, 2H), 6.83 (d, *J* = 8.7 Hz, 2H), 4.83 (t, *J* = 3.2 Hz, 2H), 3.78 (s, 3H), 2.78 – 2.69 (m, 2H), 2.60 (ddtd, *J* = 9.6, 7.6, 3.2, 1.0 Hz, 2H), 2.32 (s, 3H). **^{13}C NMR** (101 MHz, CD_2Cl_2) δ 207.7, 158.7, 138.1, 137.0, 134.8, 131.2, 130.1, 128.7, 127.7, 126.6, 114.4, 104.2, 76.3, 56.0, 36.2, 33.8, 20.8. **IR** (ATR): 2930, 2833, 1950, 1611, 1511, 1440, 1300, 1244, 1176, 1036, 845, 821 cm^{-1}. **HRMS** calculated for $C_{19}H_{20}O$ $[M]^+$ 264.1514, found 264.1503.

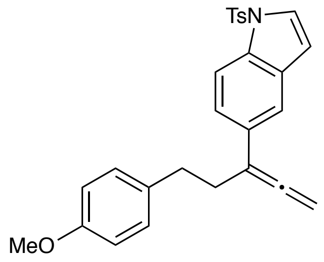

5-(5-(4-methoxyphenyl)penta-1,2-dien-3-yl)-1-tosyl-1*H*-indole (1j): The title compound was prepared according to the general procedures for alcohol tosylation and Cu-catalysed nucleophilic substitution using alcohol **s3b** (702 mg, 2.2 mmol, 1 equiv), TsCl (494 mg, 2.6 mmol, 1.2 equiv), KOH (647 mg, 300 mg/mmol of alcohol) and CH_2Cl_2 (3.4 mL, 0.63 M). The crude propargyl tosylate was used without further purification.

Crude 3-(1-tosyl-1*H*-indol-5-yl)prop-2-yn-1-yl 4-methylbenzenesulfonate (894 mg, 1.9 mmol, 1 equiv) was reacted with (4-methoxyphenethyl)magnesium bromide (2.3 mL, 1.25 equiv, 1 M in THF) using CuBr (26.8 mg, 0.19 mmol, 10 mol%) and THF (3.7 mL, 0.50 M). Purification by column chromatography (5% EtOAc in hexanes) afforded the desired allene as a colorless oil (159 mg, 19% yield). **^{1}H NMR** (400 MHz, CD_2Cl_2) δ 7.91 (d, *J* = 8.7 Hz, 1H), 7.76 (d, *J* = 8.4 Hz, 2H), 7.60 – 7.52 (m, 2H), 7.43 (dd, *J* = 8.8, 1.8 Hz, 1H), 7.26 (d, *J* = 8.2 Hz, 2H), 7.15 (d, *J* = 8.5 Hz, 2H), 6.83 (d, *J* = 8.6 Hz, 2H), 6.65 (d, *J* = 3.7 Hz, 1H), 5.09 (t, *J* = 3.3 Hz, 2H), 3.78 (s, 3H), 2.80 (dd, *J* = 9.7, 6.0 Hz, 2H), 2.70 (td, *J* = 7.5, 6.5, 3.6 Hz, 2H), 2.34 (s, 3H). **^{13}C NMR** (126 MHz, CD_2Cl_2) δ 209.4, 158.7, 146.2, 135.8, 134.8, 134.4, 132.5, 131.9, 130.7, 130.1, 127.54, 127.48, 124.1, 119.0, 114.4, 114.1, 110.0, 105.2, 79.2, 55.9, 34.0, 32.7, 22.1. **IR** (ATR): 2930, 1938, 1611, 1596, 1512, 1457, 1370, 1244, 1174, 1128, 1035, 994, 812 cm^{-1}. **HRMS** calculated for $C_{27}H_{25}NO_3SNa$ $[M+Na]^+$ 466.1453, found 466.1471.

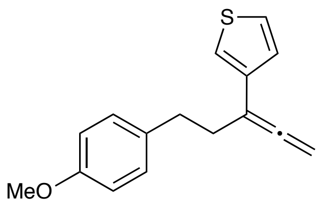

3-(5-(4-methoxyphenyl)penta-1,2-dien-3-yl)thiophene (1k): The title compound was prepared according to the general procedures for alcohol tosylation and Cu-catalysed nucleophilic substitution using alcohol **s3c** (542 mg, 3.9 mmol, 1 equiv), TsCl (897 mg, 4.7 mmol, 1.2 equiv), KOH (1.18 g, 300 mg/mmol of alcohol) and CH_2Cl_2 (6.2 mL, 0.63 M). The crude propargyl tosylate was used without further purification.

Crude 3-(thiophen-3-yl)prop-2-yn-1-yl 4-methylbenzenesulfonate (585 mg, 2.0 mmol, 1 equiv) was reacted with (4-methoxyphenethyl)magnesium bromide (2.5 mL, 1.25 equiv, 1 M in THF) using CuBr (28.7 mg, 0.20 mmol, 10 mol%) and THF (4.0 mL, 0.50 M). Purification by column chromatography (10% CH_2Cl_2 in hexanes) afforded the desired allene as a colorless oil (128 mg, 25% yield). **^{1}H NMR** (400 MHz, CD_2Cl_2) δ 77.31 (dd, *J* = 5.1, 2.9 Hz, 1H), 7.21 – 7.14 (m, 3H), 7.13 (dt, *J* = 3.0, 1.1 Hz, 1H), 6.86 (d, *J* = 8.6 Hz, 2H), 5.09 (t, *J* = 2.9 Hz, 2H), 3.80 (s, 3H), 2.84 (dd, *J* = 9.5, 6.3 Hz, 2H), 2.73 – 2.61 (m, 2H). **^{13}C NMR** (101 MHz, CD_2Cl_2) δ 209.6, 158.8, 138.9, 134.9, 130.2, 127.5, 126.2, 119.6, 114.5, 101.8, 79.1, 56.0, 33.9, 33.1. **IR** (ATR): 2923, 2855, 1940, 1611, 1511, 1440, 1300, 1244, 1176, 1036, 854 cm^{-1}. **HRMS** calculated for $C_{16}H_{16}OS$ $[M]^+$ 256.0922, found 256.0912.

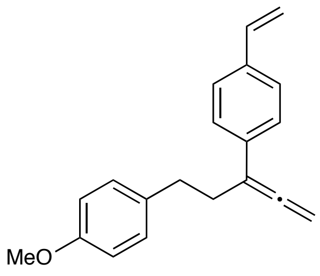

1-methoxy-4-(3-(4-vinylphenyl)penta-3,4-dien-1-yl)benzene (1l): The title compound was prepared according to the general procedures for alcohol tosylation and Cu-catalysed nucleophilic substitution using alcohol **s3d** (332 mg, 2.1 mmol, 1 equiv), TsCl (480 mg, 2.5 mmol, 1.2 equiv), KOH (630 mg, 300 mg/mmol of alcohol) and CH_2Cl_2 (3.3 mL, 0.63 M). The crude propargyl tosylate was used without further purification.

Crude 3-(4-vinylphenyl)prop-2-yn-1-yl 4-methylbenzenesulfonate (638 mg, 2.0 mmol, 1 equiv) was reacted with (4-methoxyphenethyl)magnesium bromide (2.5 mL, 1.25 equiv, 1 M in THF) using CuBr (28.7 mg, 0.20 mmol, 10 mol%) and THF (4.0 mL, 0.50 M). Purification by column chromatography (10% CH_2Cl_2 in hexanes) afforded the desired allene as a yellow oil (91 mg, 16% yield). **^{1}H NMR** (500 MHz, CD_2Cl_2) δ 7.39 (s, 4H), 7.16 (d, *J* = 8.6 Hz, 2H), 6.84 (d, *J* = 8.6 Hz, 2H), 6.72 (dd, *J* = 17.6, 10.9 Hz, 1H), 5.76 (dd, *J* = 17.5, 1.0 Hz, 1H), 5.23 (dd, *J* = 10.9, 1.0 Hz, 1H), 5.10 (t, *J* = 3.3 Hz, 2H), 3.78 (s, 3H), 2.80 (dd, *J* = 9.6, 6.2 Hz, 2H), 2.68 (ddt, *J* = 8.3, 4.2, 2.2 Hz, 2H). **^{13}C NMR** (101 MHz, CD_2Cl_2) δ 209.5, 158.7, 137.2, 136.7, 136.6, 134.8, 130.1, 127.0, 126.8, 114.4, 114.0, 105.1, 79.3, 56.0, 34.0, 32.3. **IR** (ATR): 3038, 2933, 2838, 1935,

1611, 1510, 1441, 1300, 1243, 1175, 1030, 905, 842 cm^{-1}. **HRMS** calculated for $C_{20}H_{20}ONH_4$ $[M+NH_4]^+$ 294.1858, found 294.1851.

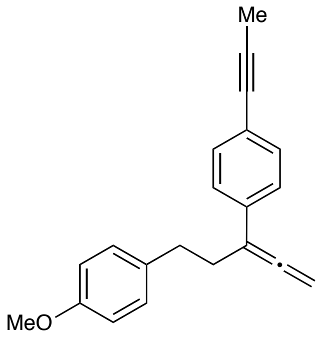

1-methoxy-4-(3-(4-(prop-1-yn-1-yl)phenyl)penta-3,4-dien-1-yl)benzene (1m): The title compound was prepared according to the general procedures for alcohol tosylation and Cu-catalysed nucleophilic substitution using alcohol **s3e** (340 mg, 2.0 mmol, 1 equiv), TsCl (458 mg, 2.4 mmol, 1.2 equiv), KOH (600 mg, 300 mg/mmol of alcohol) and CH_2Cl_2 (3.2 mL, 0.63 M). The crude propargyl tosylate was used without further purification.

Crude 3-(4-(prop-1-yn-1-yl)phenyl)prop-2-yn-1-yl 4-methylbenzenesulfonate (622 mg, 1.9 mmol, 1 equiv) was reacted with (4-methoxyphenethyl)magnesium bromide (2.4 mL, 1.25 equiv, 1 M in THF) using CuBr (27.5 mg, 0.19 mmol, 10 mol%) and THF (3.8 mL, 0.50 M). Purification by column chromatography (10% CH_2Cl_2 in hexanes) afforded the desired allene as a colorless oil (256 mg, 46% yield). **^{1}H NMR** (500 MHz, CD_2Cl_2) δ 7.35 (s, 4H), 7.15 (d, *J* = 8.6 Hz, 2H), 6.84 (d, *J* = 8.6 Hz, 2H), 5.11 (t, *J* = 3.3 Hz, 2H), 3.78 (s, 3H), 2.80 (dd, *J* = 9.6, 6.2 Hz, 2H), 2.71 – 2.63 (m, 2H), 2.06 (s, 3H). **^{13}C NMR** (126 MHz, CD_2Cl_2) δ 209.6, 158.8, 136.4, 134.8, 132.3, 130.1, 126.6, 123.0, 114.5, 105.1, 86.9, 80.2, 79.5, 56.0, 34.0, 32.2, 4.8. **IR** (ATR): 2914, 2833, 1937, 1611, 1510, 1440, 1300, 1244, 1176, 1107, 1036, 838, 821 cm^{-1}. **HRMS** calculated for $C_{21}H_{20}O$ $[M]^+$ 288.1514, found 288.1513.

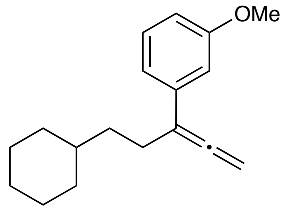

1-(5-cyclohexylpenta-1,2-dien-3-yl)-3-methoxybenzene (1p): The title compound was prepared according to the general procedures for alcohol tosylation and Cu-catalysed nucleophilic substitution using alcohol **s3f** (652 mg, 4.0 mmol, 1 equiv), TsCl (920 mg, 4.8 mmol, 1.2 equiv), KOH (1.21 g, 300 mg/mmol of alcohol) and CH_2Cl_2 (6.4 mL, 0.63 M). The crude propargyl tosylate was used without further purification.

Crude 3-(3-methoxyphenyl)prop-2-yn-1-yl 4-methylbenzenesulfonate (1.27 g, 4.0 mmol, 1 equiv) was reacted with (2-cyclohexylethyl)magnesium bromide (5.0 mL, 1.25 equiv, 1 M in THF) using CuBr (57.7 mg, 0.40 mmol, 10 mol%) and THF (8.0 mL, 0.50 M). Purification by column chromatography (10% CH_2Cl_2 in hexanes) afforded the desired allene as a colorless oil (167 mg, 16% yield). **^{1}H NMR** (400 MHz, CD_2Cl_2) δ 7.24 (t, *J* = 8.0 Hz, 1H), 7.06 – 7.00 (m, 1H), 6.97 (t, *J* = 2.1 Hz, 1H), 6.76 (ddd, *J* = 8.1, 2.6, 0.9 Hz, 1H), 5.08 (t, *J* = 3.3 Hz, 2H), 3.81 (s, 3H), 2.47 –

2.38 (m, 2H), 1.83 – 1.63 (m, 5H), 1.51 – 1.43 (m, 2H), 1.43 – 1.31 (m, 1H), 1.31 – 1.13 (m, 3H), 1.03 – 0.90 (m, 2H). **^{13}C NMR** (101 MHz, CD_2Cl_2) δ 209.4, 160.6, 139.0, 130.0, 119.3, 112.63, 112.61, 106.0, 78.7, 55.9, 38.4, 36.4, 34.2, 27.8, 27.6, 27.3. **IR** (ATR): 2919, 2849, 1939, 1597, 1580, 1487, 1448, 1286, 1262, 1245, 1165, 1051, 846 cm^{-1}. **HRMS** calculated for $C_{18}H_{24}OH$ $[M+H]^+$ 257.1906, found 257.1902.

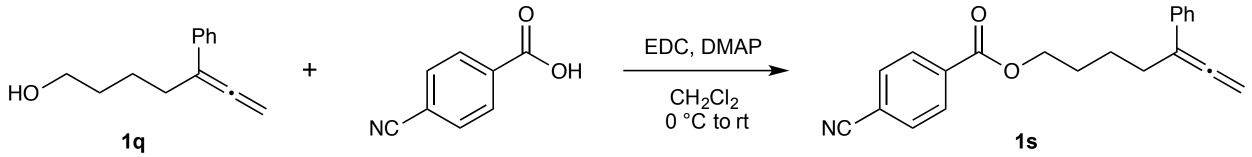

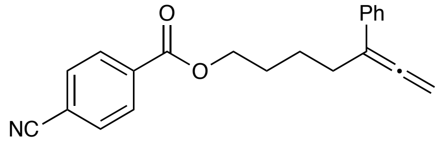

5-phenylhepta-5,6-dien-1-yl 4-cyanobenzoate (1s): An oven-dried round bottom flask equipped with a magnetic stir bar was charged with EDC hydrochloride (249 mg, 1.30 mmol, 1.3 equiv), DMAP (12.2 mg, 0.10 mmol, 10 mol%), and CH_2Cl_2 (2.5 mL, 0.40 M). After cooling the resulting mixture to 0 °C, 4-cyanobenzoic acid (147 mg, 1.0 mmol, 1 equiv) and allene **1q** (226 mg, 1.2 mmol, 1.2 equiv) were added. The resulting mixture was warmed to rt and stirred for 12 h. The reaction mixture was diluted with ether (10 mL) and washed with 1 M HCl (10 mL). The organic layer was washed with brine, dried with anhydrous Na_2SO_4 and concentrated *in vacuo*. The residue was purified by column chromatography (10% EtOAc in hexanes) to afford the title compound as a yellow solid (265 mg, 83% yield). **^{1}H NMR** (400 MHz, CD_2Cl_2) δ 8.12 (d, *J* = 8.7 Hz, 2H), 7.75 (d, *J* = 8.7 Hz, 2H), 7.48 – 7.40 (m, 2H), 7.34 (dddd, *J* = 8.3, 6.8, 1.3, 0.7 Hz, 2H), 7.27 – 7.20 (m, 1H), 5.13 (t, *J* = 3.4 Hz, 2H), 4.40 (t, *J* = 6.5 Hz, 2H), 2.58 – 2.49 (m, 2H), 1.97 – 1.87 (m, 2H), 1.82 – 1.70 (m, 2H). **^{13}C NMR** (101 MHz, CD_2Cl_2) δ 209.2, 165.6, 137.0, 135.1, 133.0, 130.7, 129.1, 127.4, 126.7, 118.8, 117.0, 105.3, 79.0, 66.4, 29.7, 29.0, 24.9. **IR** (ATR): 2915, 2229, 1937, 1716, 1452, 1278, 1122, 1110, 1032, 1022, 855, 762 cm^{-1}. **HRMS** calculated for $C_{21}H_{19}NO_2H$ $[M+H]^+$ 318.1494, found 318.1497.

Deuterium Labelling Experiments

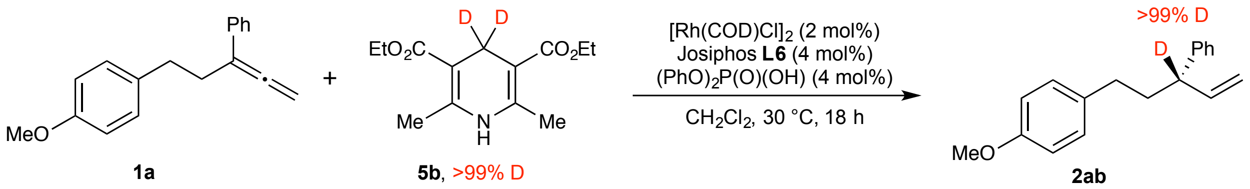

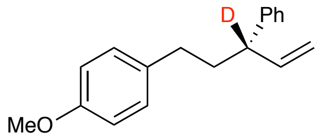

(*S*)-1-methoxy-4-(3-phenylpent-4-en-1-yl-3-*d*)benzene (2ab): In a N_2-filled glovebox, $[Rh(COD)Cl]_2$ (1.0 mg, 0.0020 mmol, 2 mol%), $(PhO)_2P(O)(OH)$ (1.0 mg, 0.0040 mmol, 4 mol%), Josiphos **L6** (4.6 mg, 0.0040 mmol, 4 mol%), Hantzsch ester **5b** (51.1 mg, 0.20 mmol, 2.0 equiv), allene **1a** (25.0 mg, 0.10 mmol, 1 equiv), and CH_2Cl_2 (0.10 mL, 1 M) were added to a 1 dram vial equipped with a magnetic stir bar. The vial was then sealed with a Teflon-lined screw cap and stirred at 30 °C for 18 h. The reaction mixture was cooled to rt and concentrated *in vacuo*. The regioselectivity was determined by ^{1}H NMR analysis of the unpurified reaction mixture. The title compound was isolated by preparatory TLC (5% EtOAc in hexanes) as a colorless oil (20.5 mg, 81% yield, >20:1 *rr*, 96:4 *er*, $[\alpha]^{24}_D$ = +11.6 (*c* 1.3, $CHCl_3$)). **^{1}H NMR** (400 MHz, $CDCl_3$) δ 7.37 – 7.31 (m, 2H), 7.26 – 7.20 (m, 3H), 7.10 (d, *J* = 8.8 Hz, 2H), 6.85 (d, *J* = 8.7 Hz, 2H), 6.06 – 5.94 (m, 1H), 5.12 – 5.03 (m, 2H), 3.81 (s, 3H), 2.63 – 2.45 (m, 2H), 2.10 – 1.97 (m, 2H). **^{13}C NMR** (101 MHz, $CDCl_3$) δ 157.9, 144.3, 142.3, 134.4, 129.4, 128.6, 127.8, 126.4, 114.4, 113.9, 55.4, 49.1 – 48.7 (m), 37.2, 32.8. **IR** (ATR): 2932, 2833, 1611, 1511, 1447, 1300, 1243, 1176, 1035, 913, 828, 750 cm^{-1}. **HRMS** calculated for $C_{18}H_{19}DONH_4$ $[M+NH_4]^+$ 271.1921, found 271.1922. **Chiral SFC**: 100 mm CHIRALCEL OJ-H, 10% *i*-PrOH, 3.0 mL/min, 220 nm, 44 °C, nozzle pressure = 200 bar CO_2, t_{R1} (minor) = 2.2 min, t_{R2} (major) = 2.6 min.

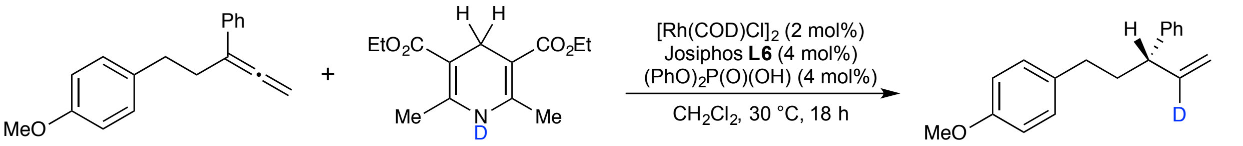

1a **5c**, 90% D **2ac** 50% D

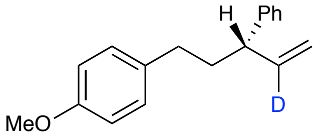

(*S*)-1-methoxy-4-(3-phenylpent-4-en-1-yl-4-*d*)benzene (2ac): In a N_2-filled glovebox, $[Rh(COD)Cl]_2$ (1.0 mg, 0.0020 mmol, 2 mol%), $(PhO)_2P(O)(OH)$ (1.0 mg, 0.0040 mmol, 4 mol%), Josiphos **L6** (4.6 mg, 0.0040 mmol, 4 mol%), Hantzsch ester **5c** (50.9 mg, 0.20 mmol, 2.0 equiv), allene **1a** (25.0 mg, 0.10 mmol, 1 equiv), and CH_2Cl_2 (0.10 mL, 1 M) were added to a 1 dram vial equipped with a magnetic stir bar. The vial was then sealed with a Teflon-lined screw cap and stirred at 30 °C for 18 h. The reaction mixture was cooled to rt and concentrated *in vacuo*. The regioselectivity was determined by ^1H NMR analysis of the unpurified reaction mixture. The title compound was isolated by preparatory TLC (5% EtOAc in hexanes) as a colorless oil (20.7 mg, 82% yield, >20:1 *rr*, 94:6 *er*, $[\alpha]^{24}_D$ = +10.8 (*c* 1.3, $CHCl_3$)). **^{1}H NMR** (400 MHz, $CDCl_3$) δ 7.34 (dd, *J* = 8.7, 6.8 Hz, 2H), 7.26 – 7.19 (m, 3H), 7.12 – 7.06 (m, 2H), 6.85 (d, *J* = 8.7 Hz, 2H), 6.06 – 5.94 (m, 0.5H), 5.12 – 5.01 (m, 2H), 3.81 (s, 3H), 3.30 (q, *J* = 7.2 Hz, 1H), 2.63 – 2.46 (m, 2H), 2.04 (td, *J* = 8.7, 8.2, 6.9 Hz, 2H). **^{13}C NMR** (101 MHz, $CDCl_3$) δ 157.9, 144.3, 142.3, 134.4, 129.4, 128.6, 127.8, 126.4, 114.4, 113.9, 55.4, 49.3, 37.3, 32.9. **IR** (ATR): 3027, 2933, 2833, 1611, 1510, 1452, 1243, 1176, 1035, 913, 824, 752 cm^{-1}. **HRMS** calculated for $C_{18}H_{19}DONH_4$ $[M+NH_4]^+$ 271.1921, found 271.1909. **Chiral SFC**: 100 mm CHIRALCEL OJ-H, 10% *i*-PrOH, 3.0 mL/min, 220 nm, 44 °C, nozzle pressure = 200 bar CO_2, t_{R1} (minor) = 2.2 min, t_{R2} (major) = 2.6 min.

Supplementary Figures

Supplementary Figure 1. ^{1}H NMR spectrum of 2a

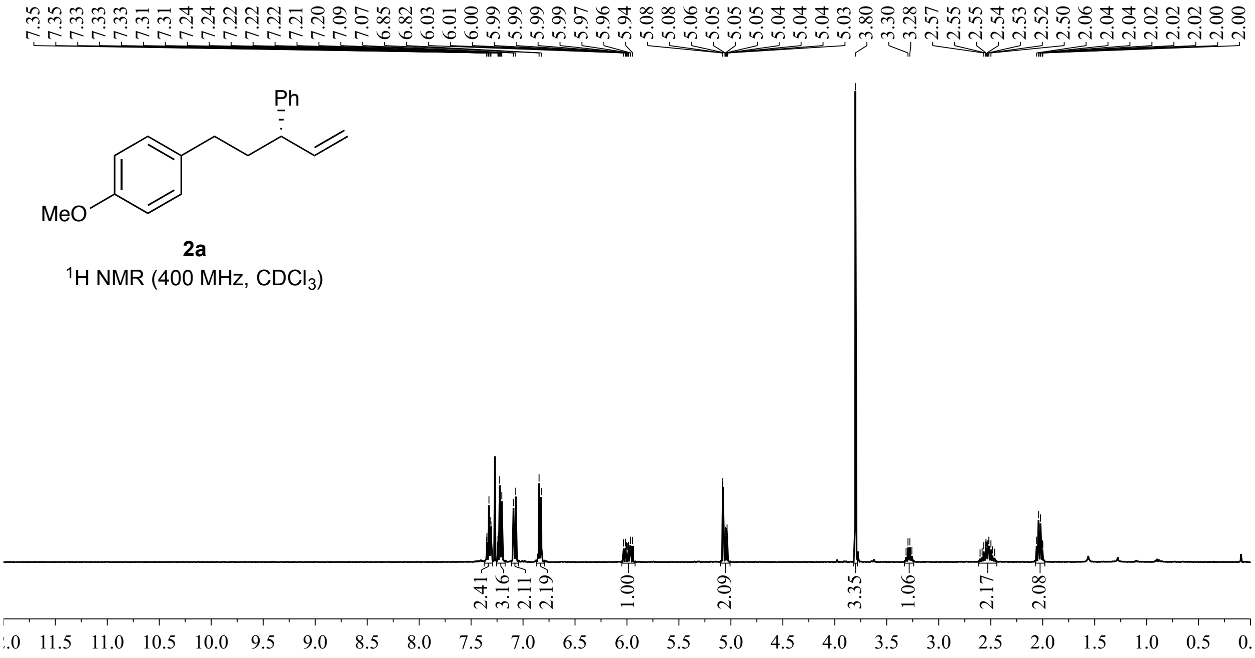

Supplementary Figure 2. ^{13}C NMR spectrum of 2a

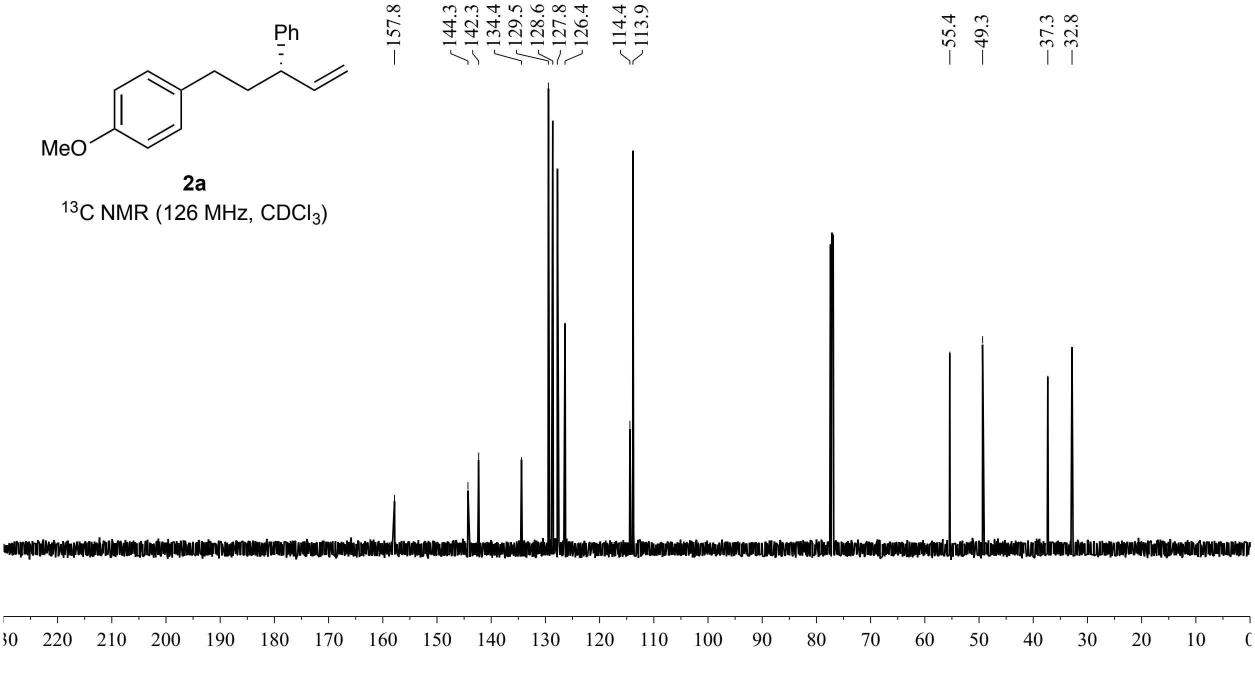

Supplementary Figure 3. ^{1}H NMR spectrum of 2b

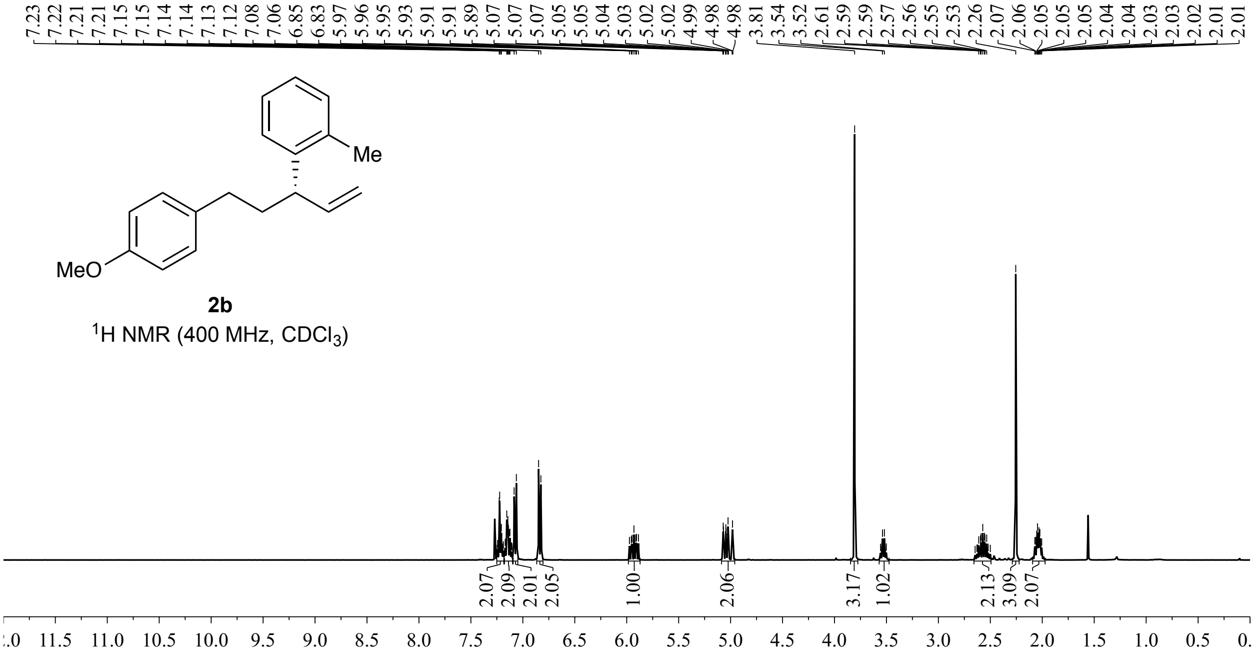

Supplementary Figure 4. ^{13}C NMR spectrum of 2b

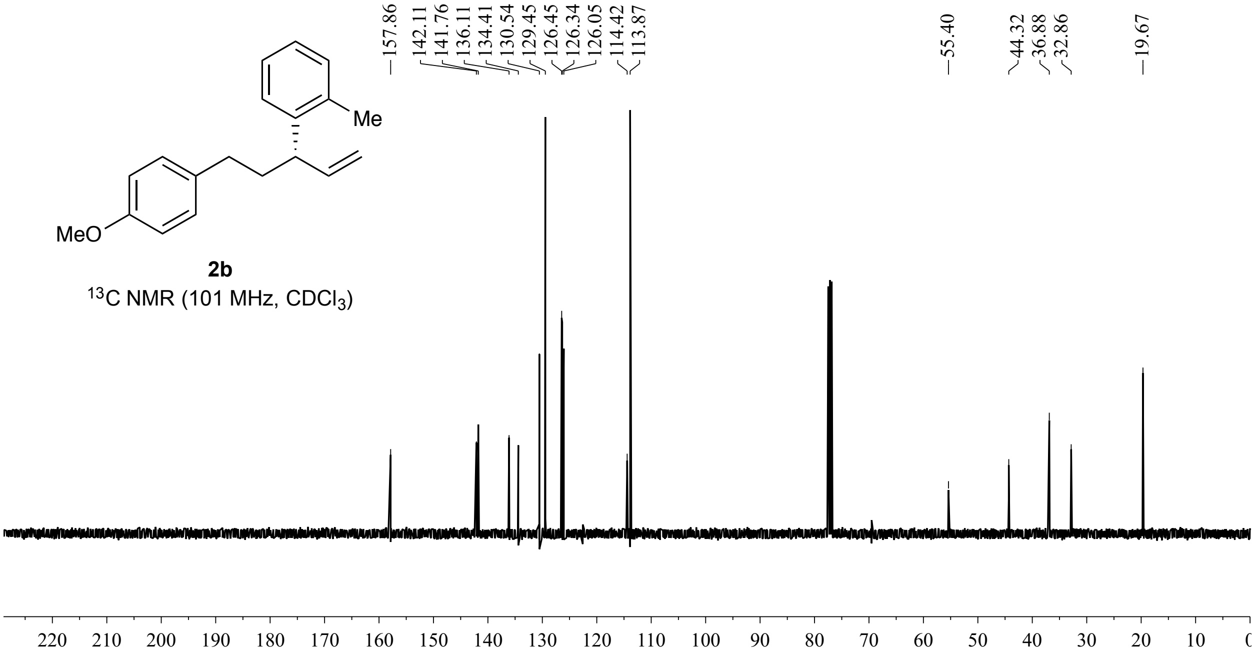

Supplementary Figure 5. ^{1}H NMR spectrum of 2c

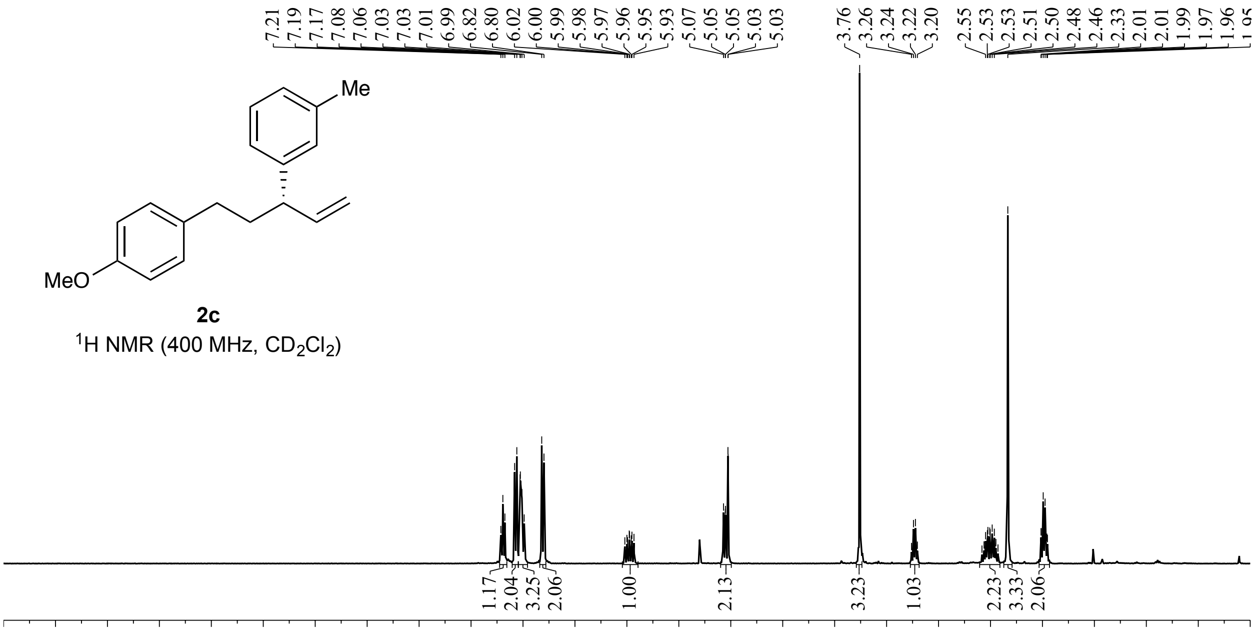

Supplementary Figure 6. ^{13}C NMR spectrum of 2c

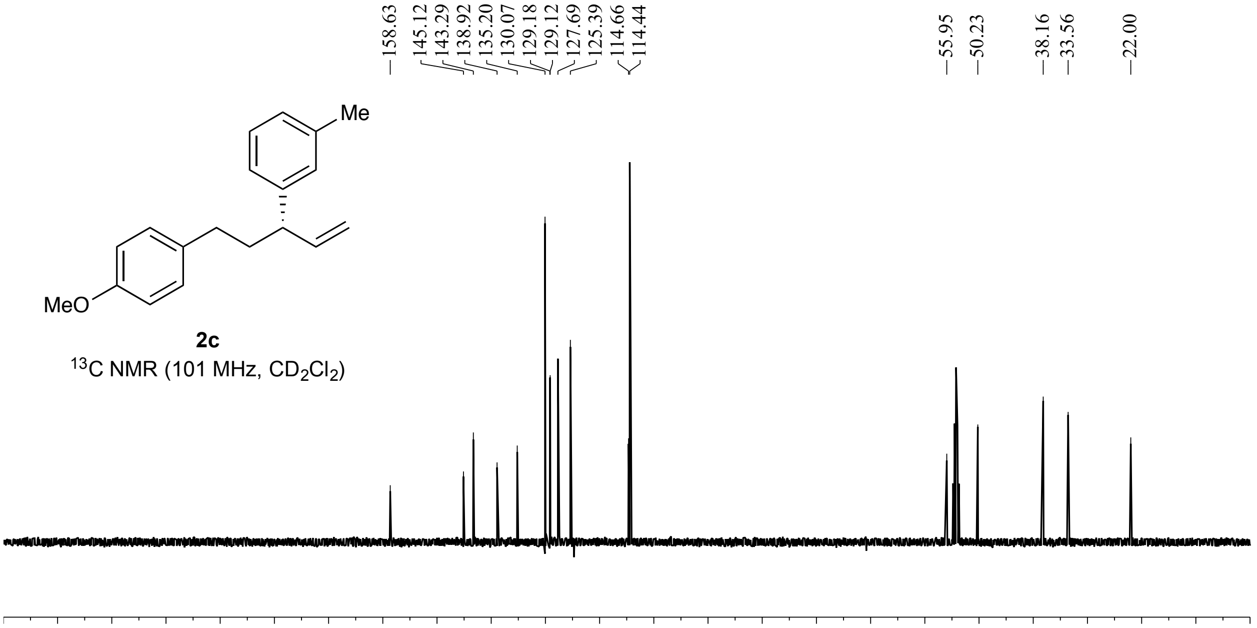

Supplementary Figure 7. ^{1}H NMR spectrum of 2d

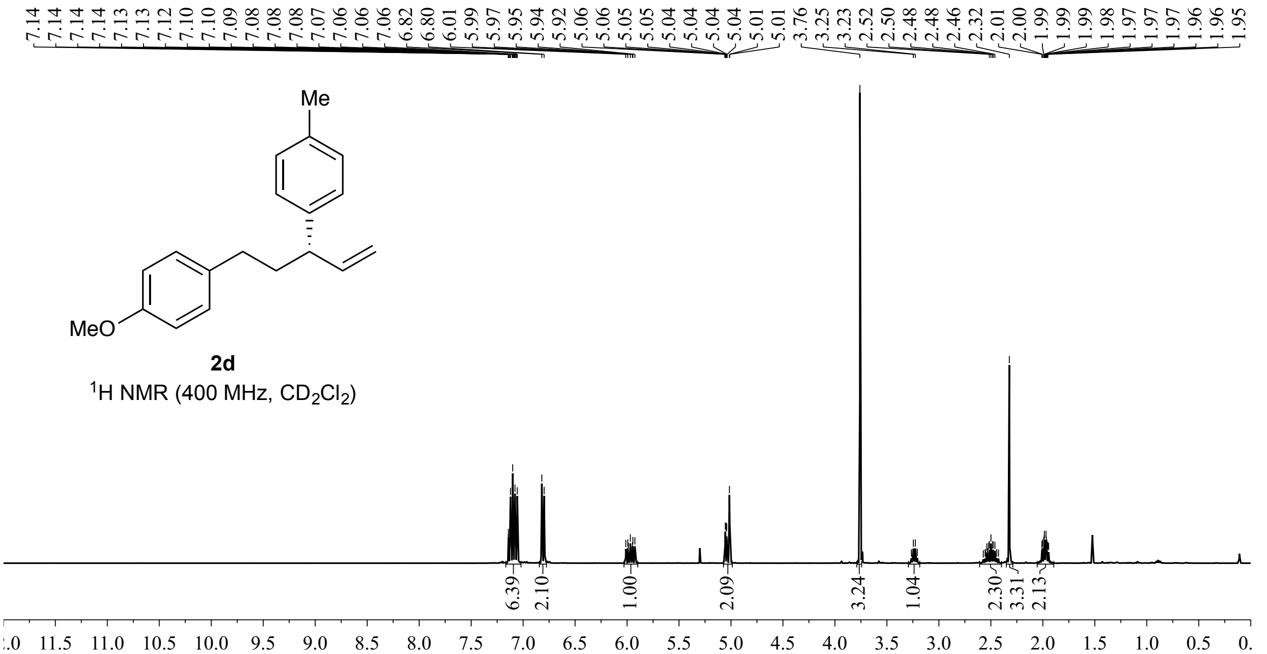

Supplementary Figure 8. ^{13}C NMR spectrum of 2d

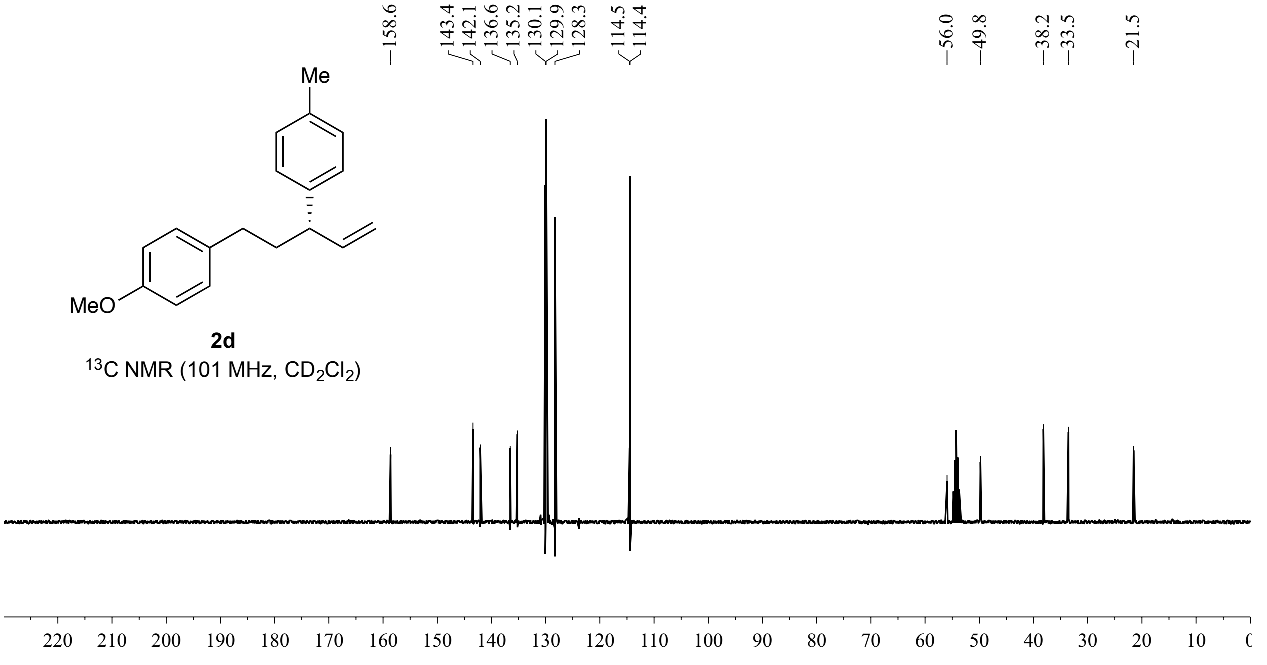

Supplementary Figure 9. ^{1}H NMR spectrum of 2e

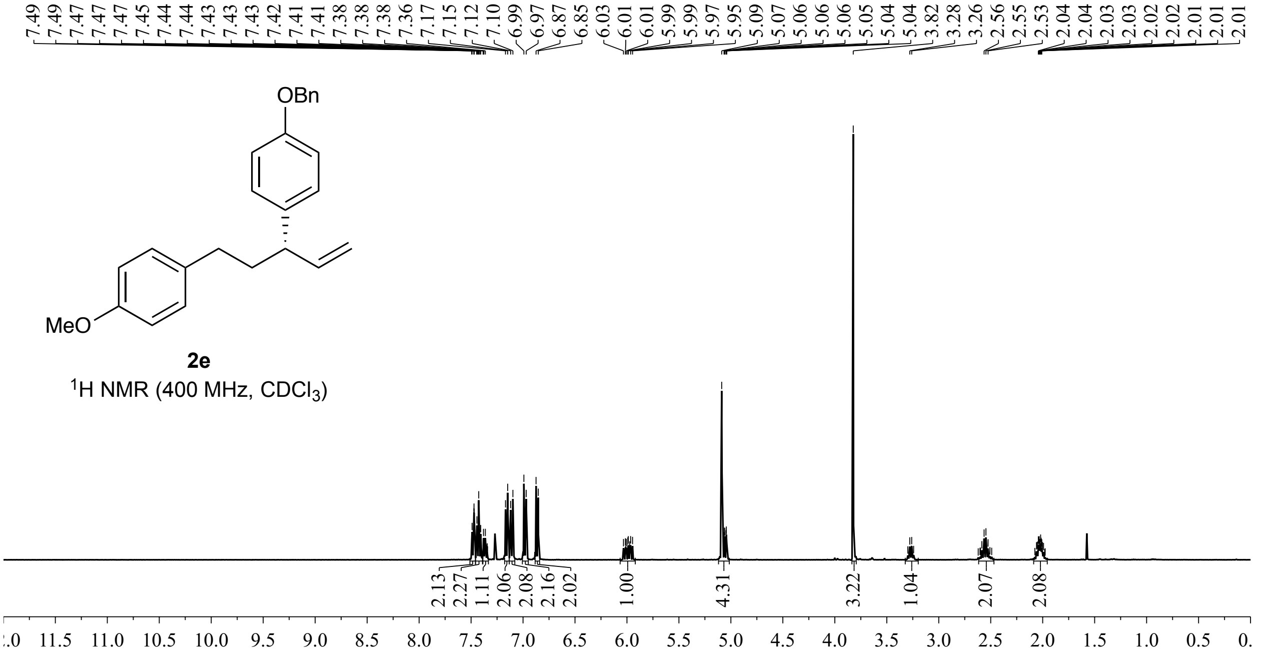

Supplementary Figure 10. ^{13}C NMR spectrum of 2e

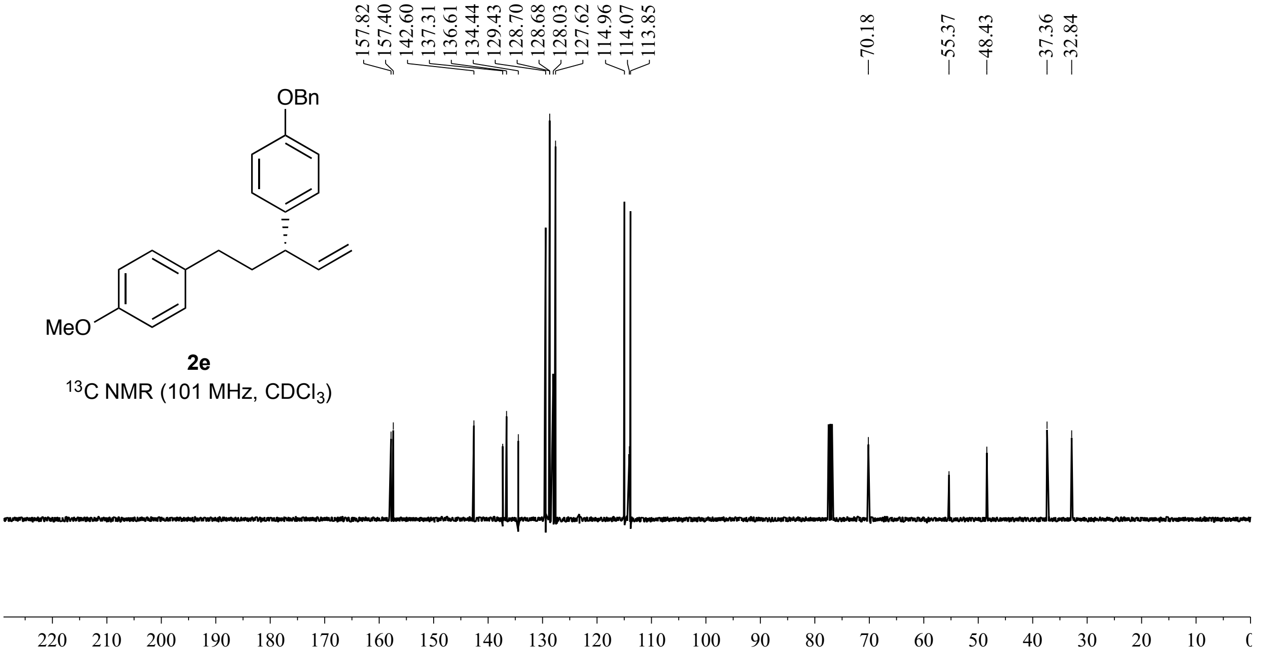

Supplementary Figure 11. ^{1}H NMR spectrum of 2f

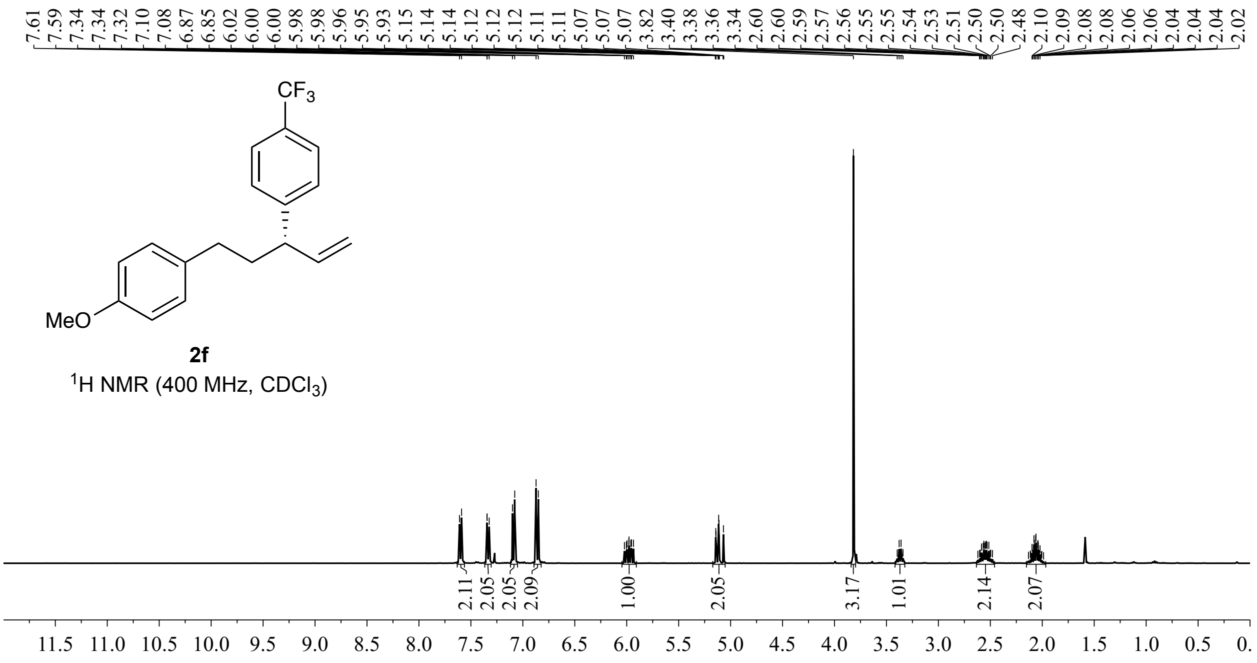

Supplementary Figure 12. ^{13}C NMR spectrum of 2f

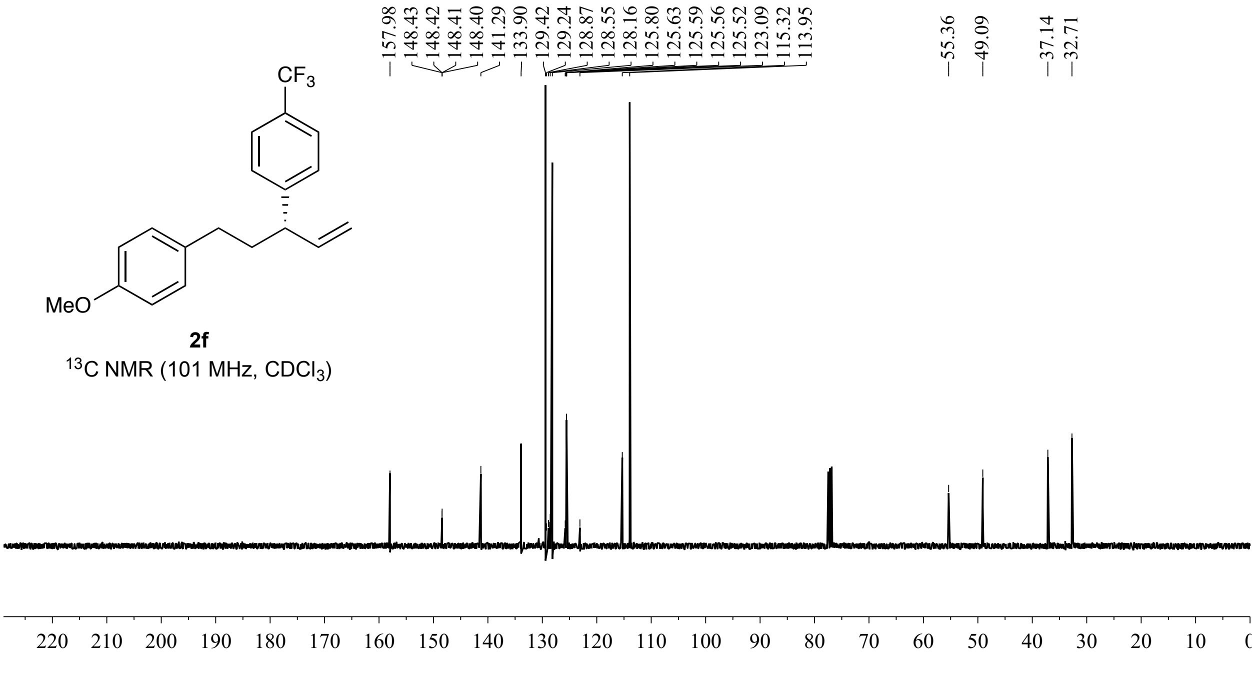

Supplementary Figure 13. ^{19}F NMR spectrum of 2f

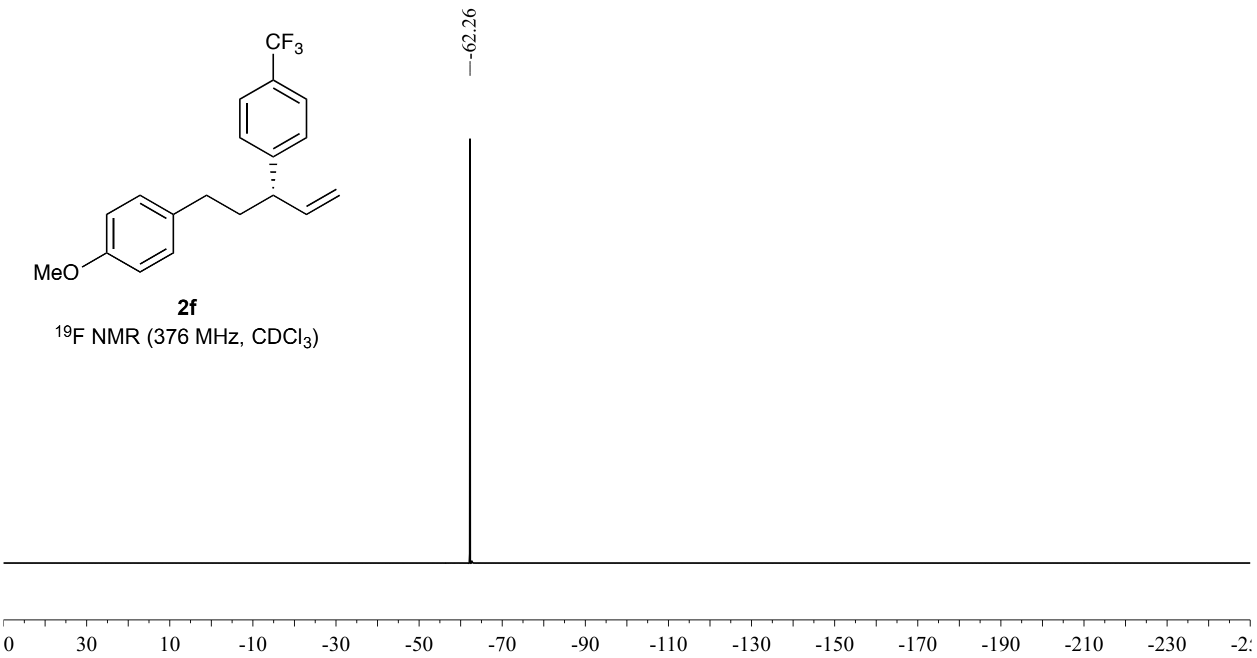

Supplementary Figure 14. ^{1}H NMR spectrum of 2g

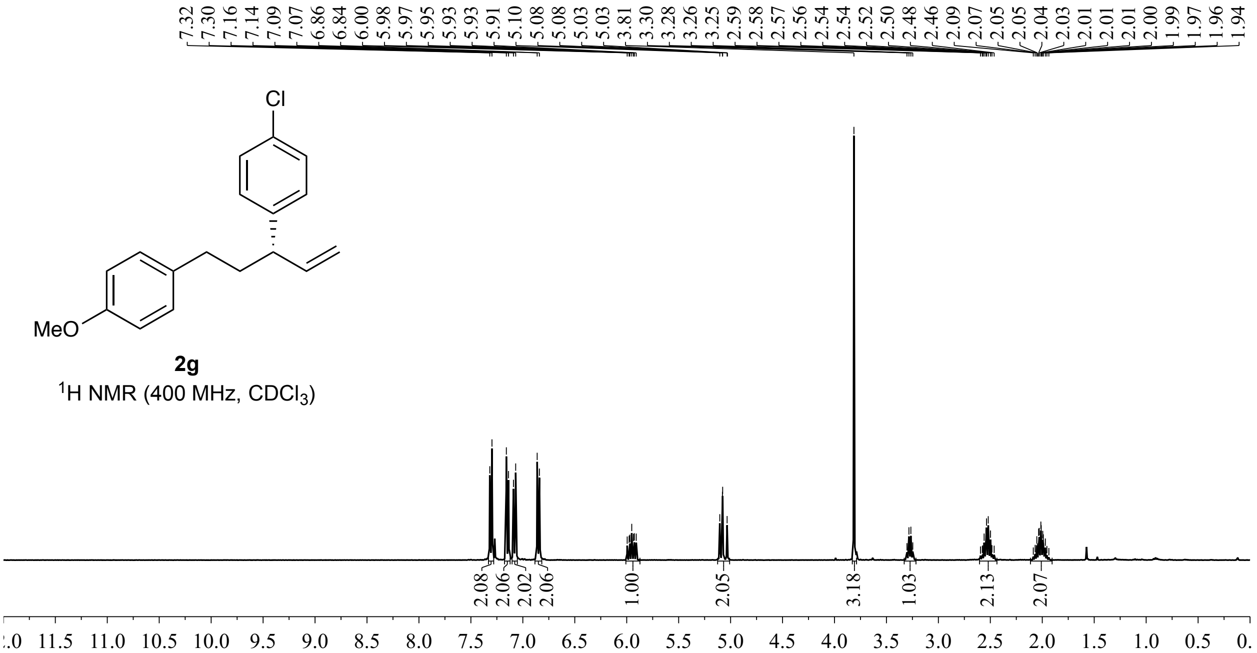

Supplementary Figure 15. ^{13}C NMR spectrum of 2g

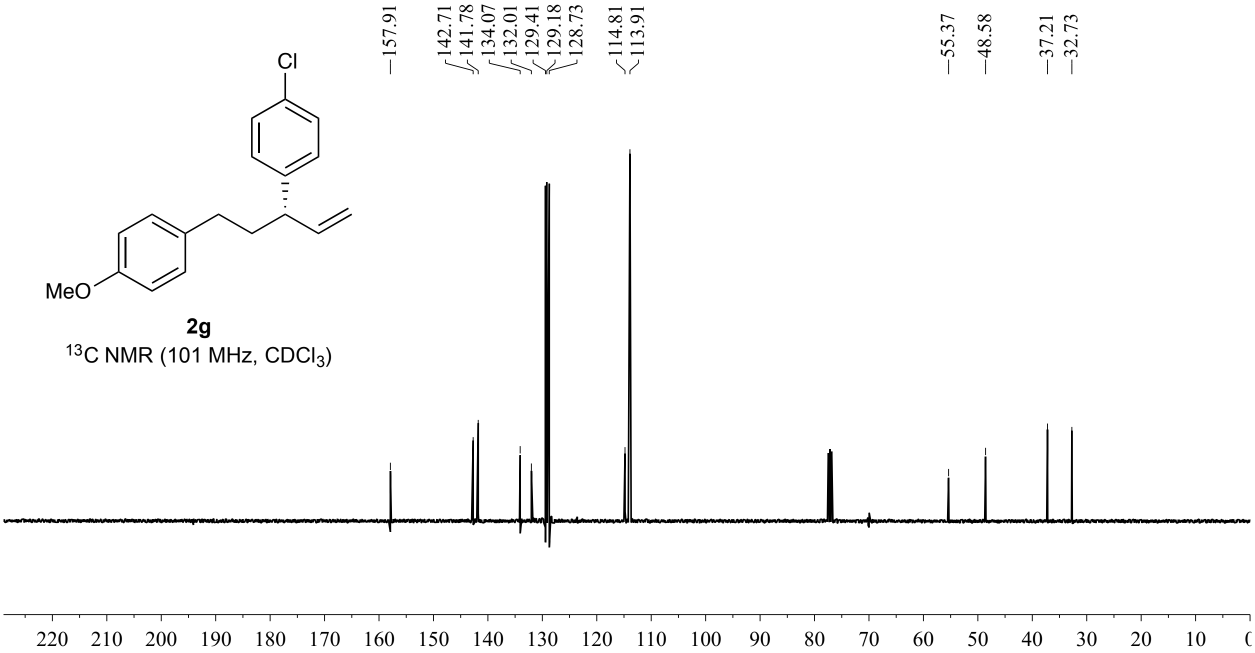

Supplementary Figure 16. ^{1}H NMR spectrum of 2h

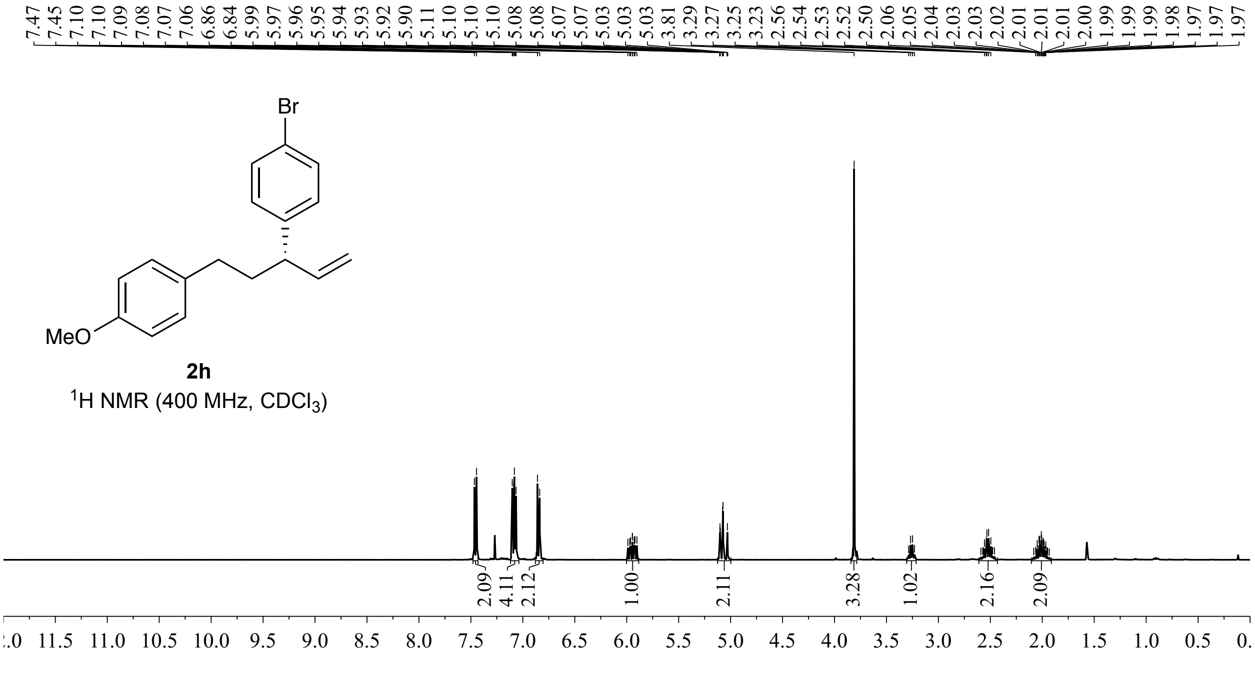

Supplementary Figure 17. ^{13}C NMR spectrum of 2h

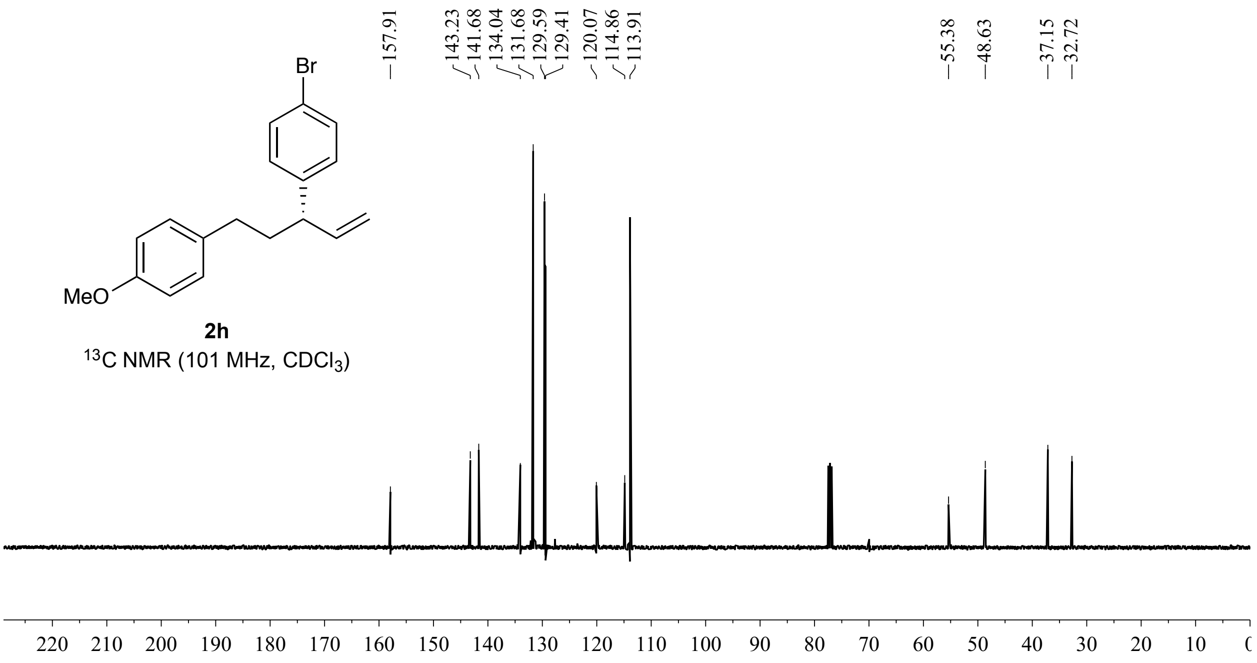

Supplementary Figure 18. ^{1}H NMR spectrum of 2i

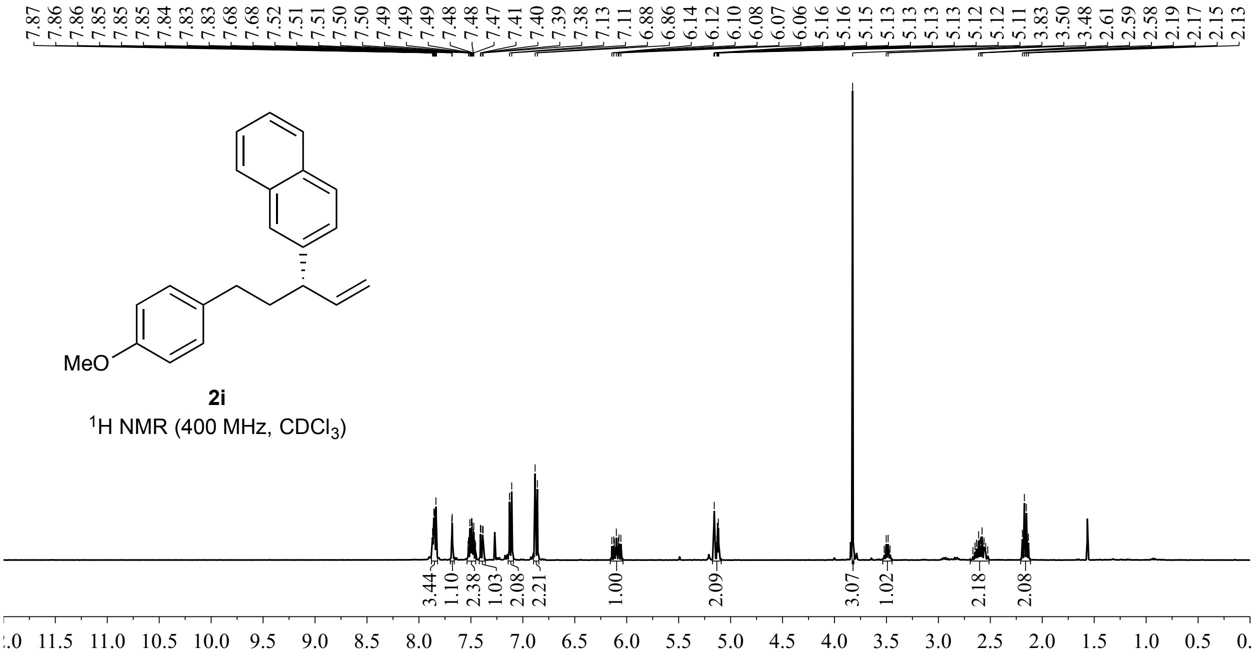

Supplementary Figure 19. ^{13}C NMR spectrum of 2i

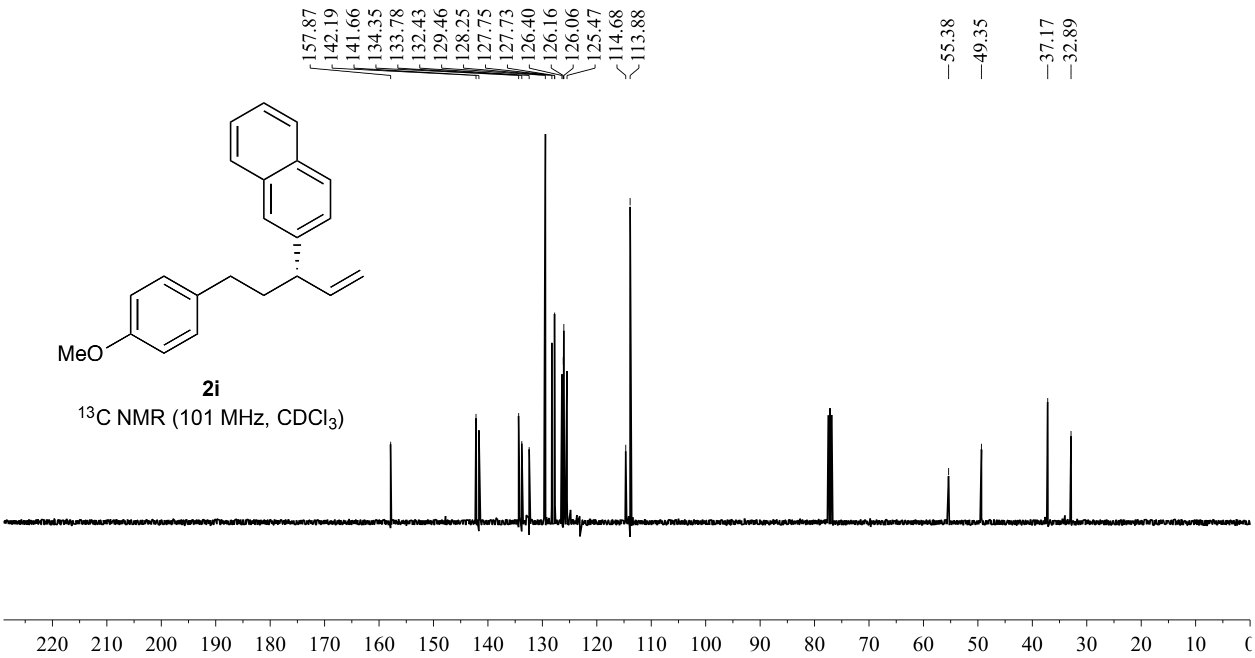

Supplementary Figure 20. ^{1}H NMR spectrum of 2j

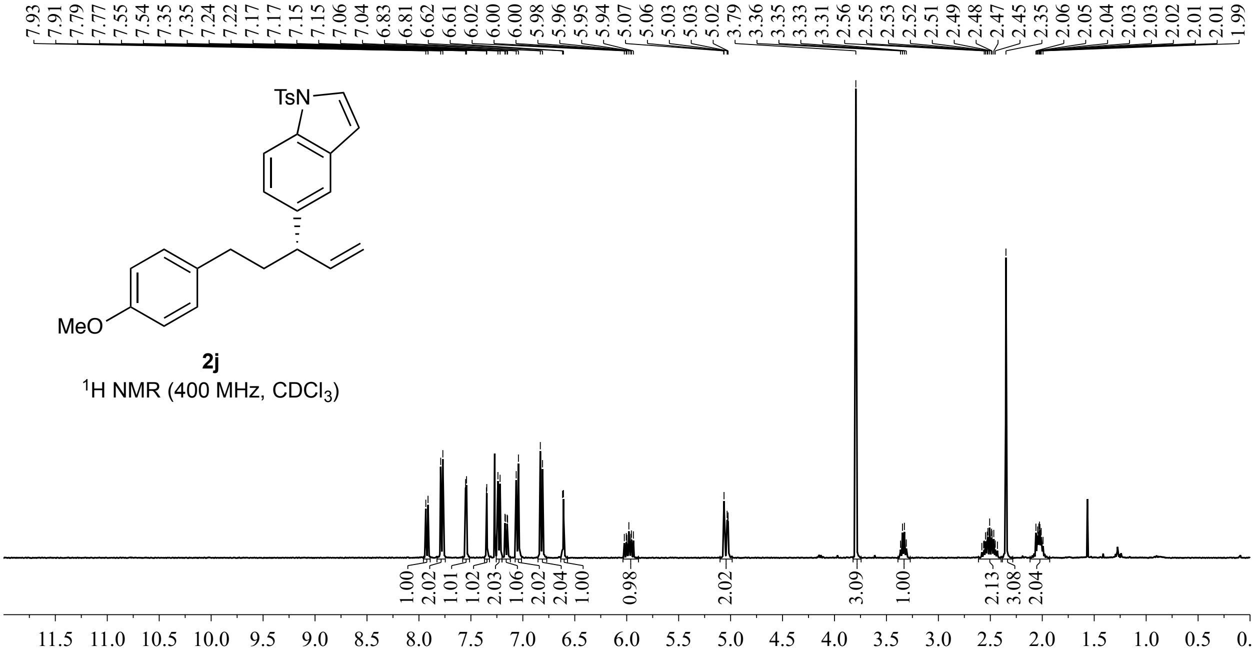

Supplementary Figure 21. ^{13}C NMR spectrum of 2j

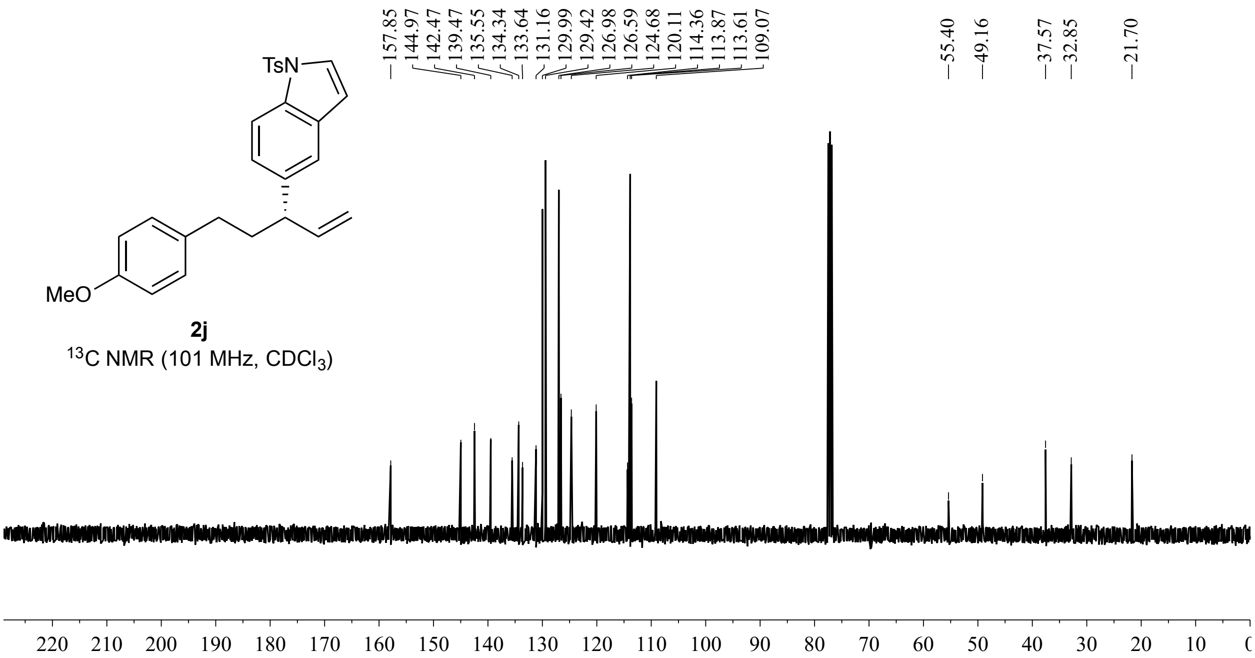

2j

^{13}C NMR (101 MHz, $CDCl_3$)

Supplementary Figure 22. ^{1}H NMR spectrum of 2k

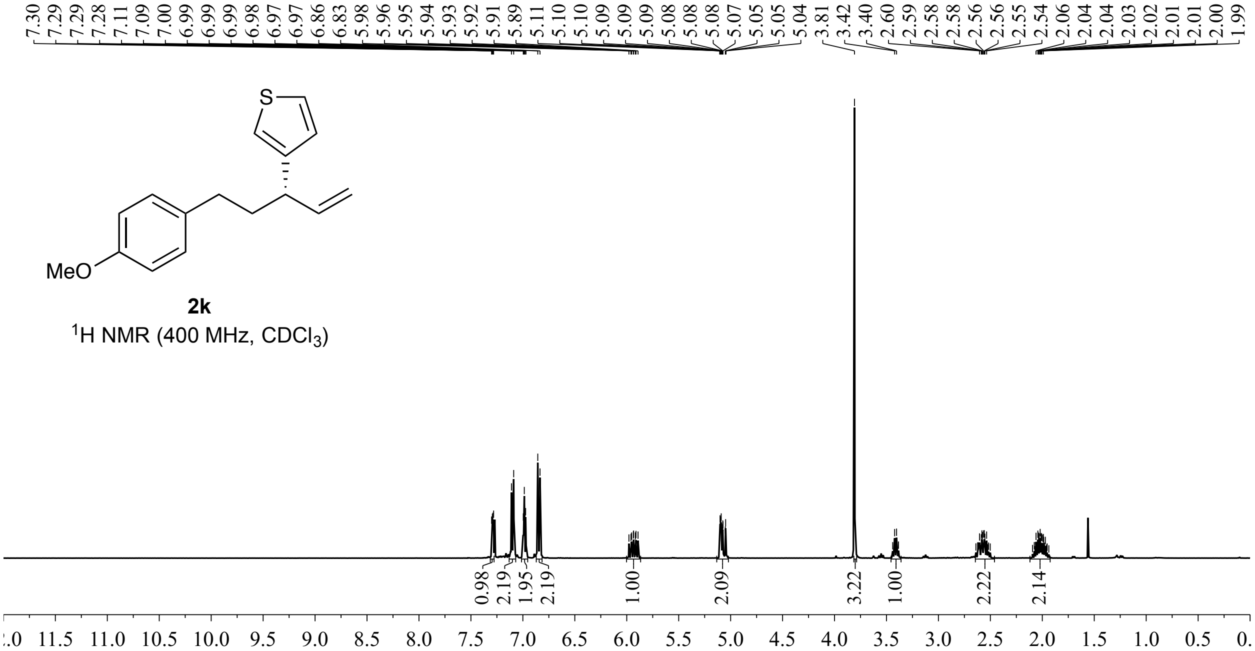

2k

^{1}H NMR (400 MHz, $CDCl_3$)

Supplementary Figure 23. ^{13}C NMR spectrum of 2k

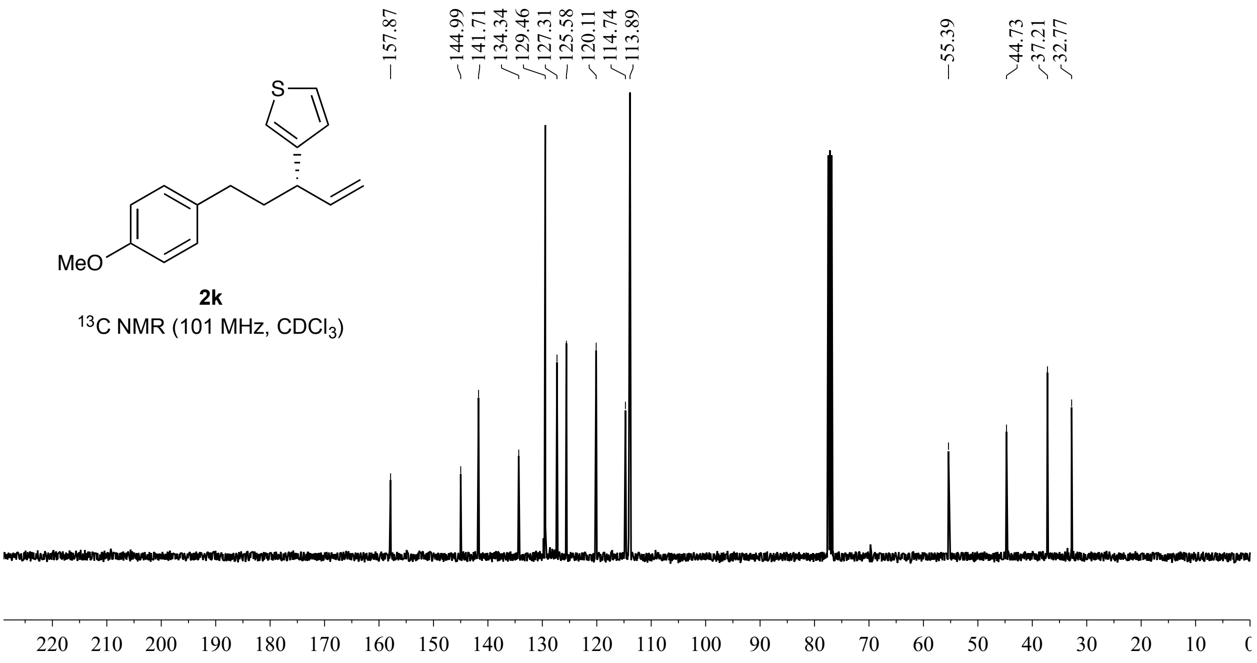

Supplementary Figure 24. ^{1}H NMR spectrum of 2l

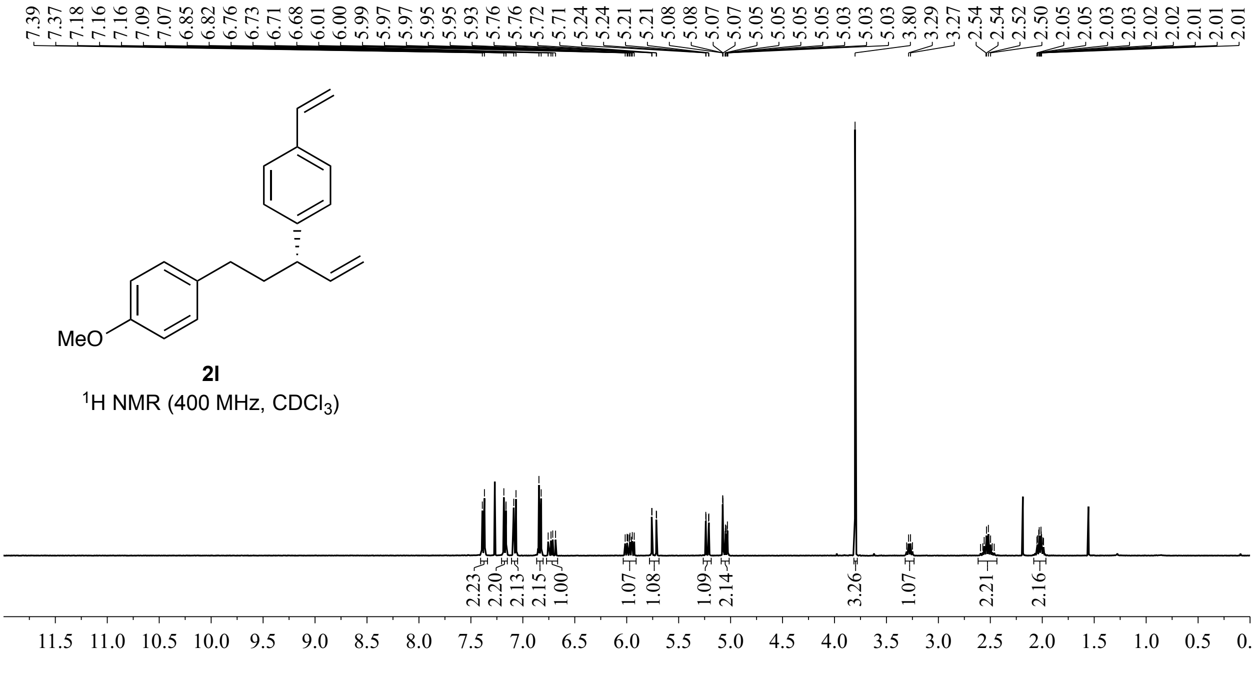

Supplementary Figure 25. ^{13}C NMR spectrum of 2l

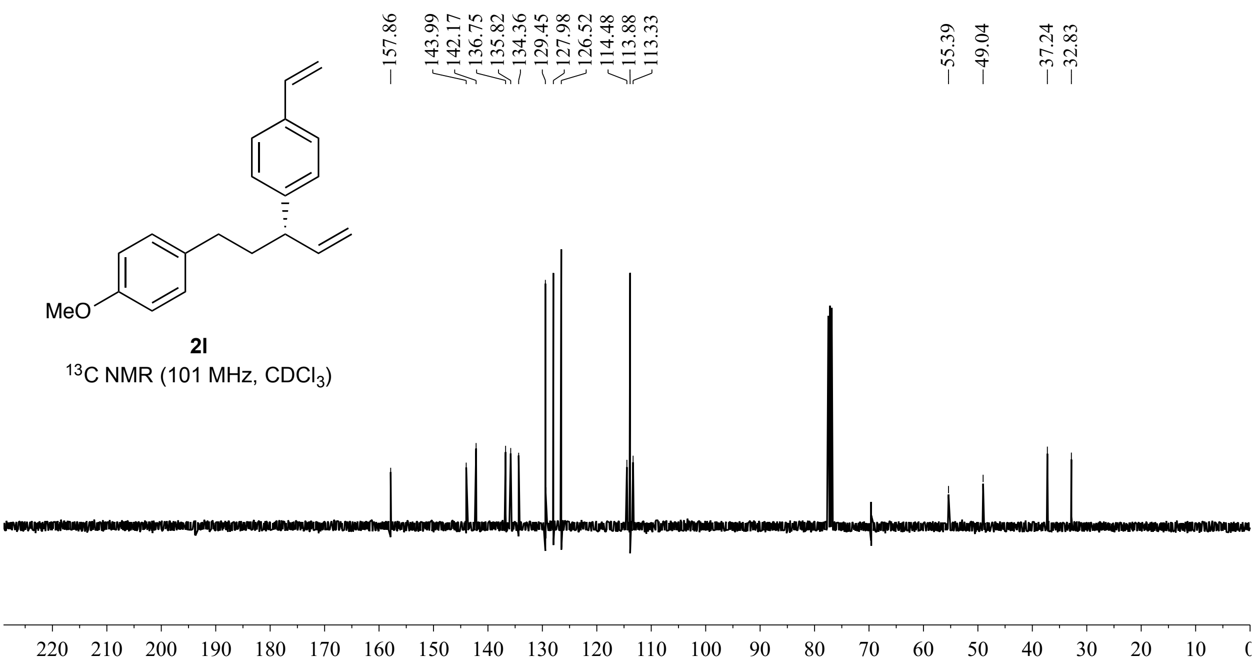

Supplementary Figure 26. ^{1}H NMR spectrum of 2m

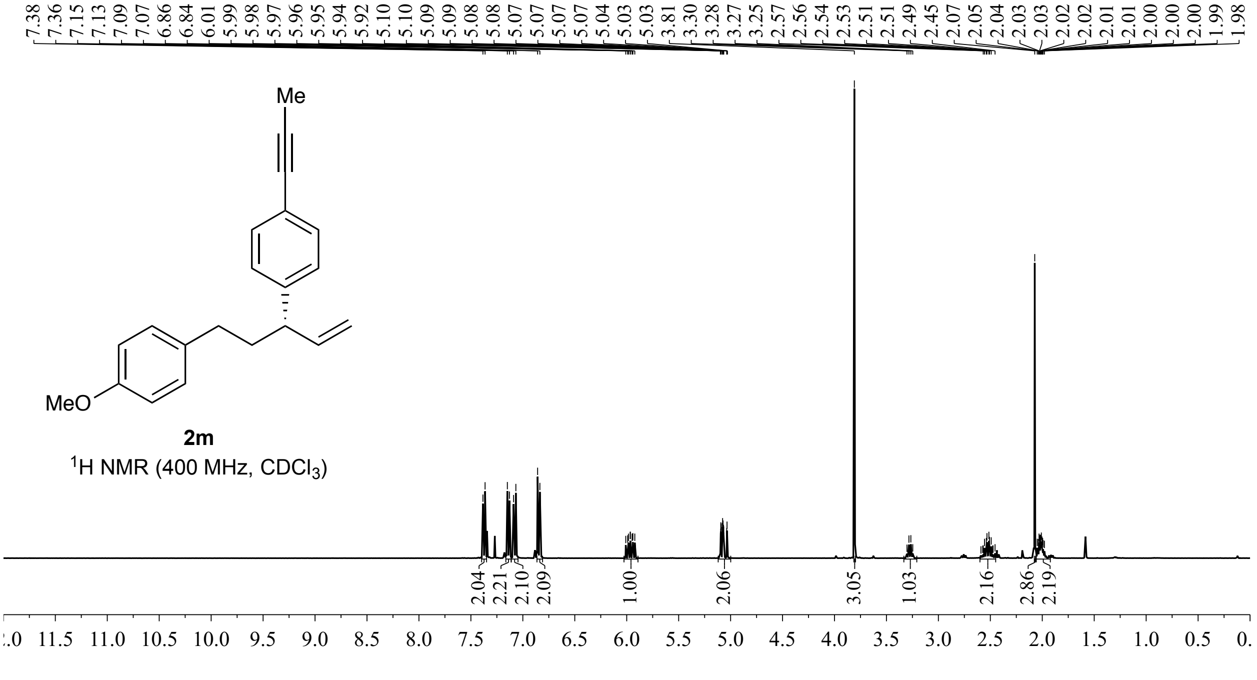

Supplementary Figure 27. ^{13}C NMR spectrum of 2m

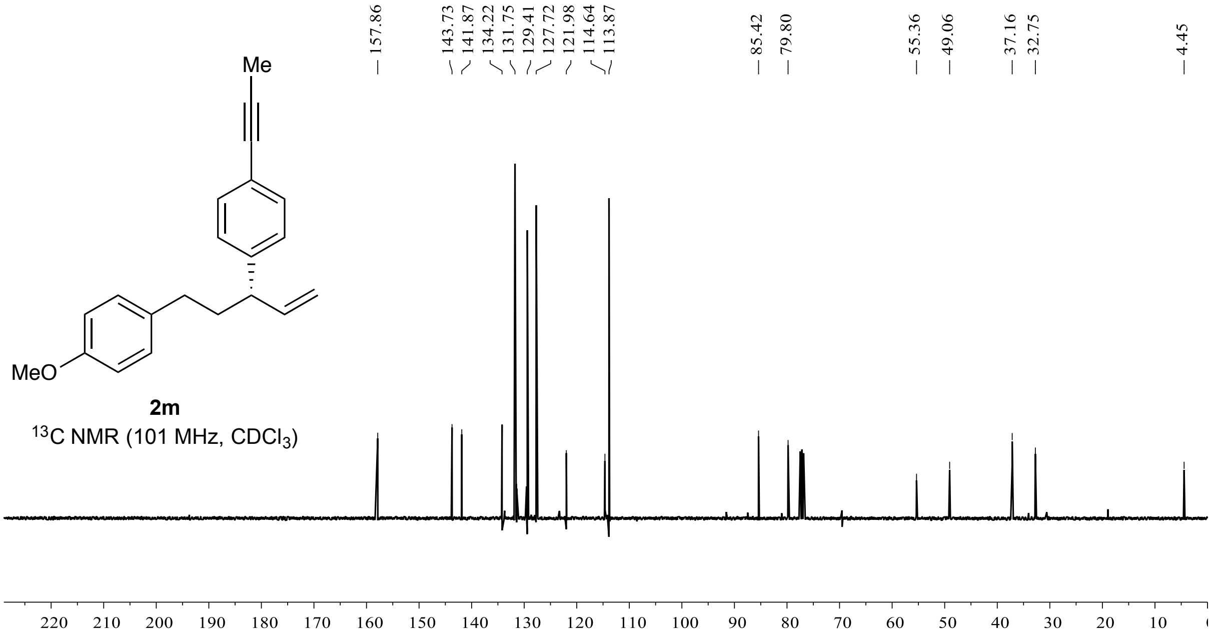

Supplementary Figure 28. ^{1}H NMR spectrum of 2n

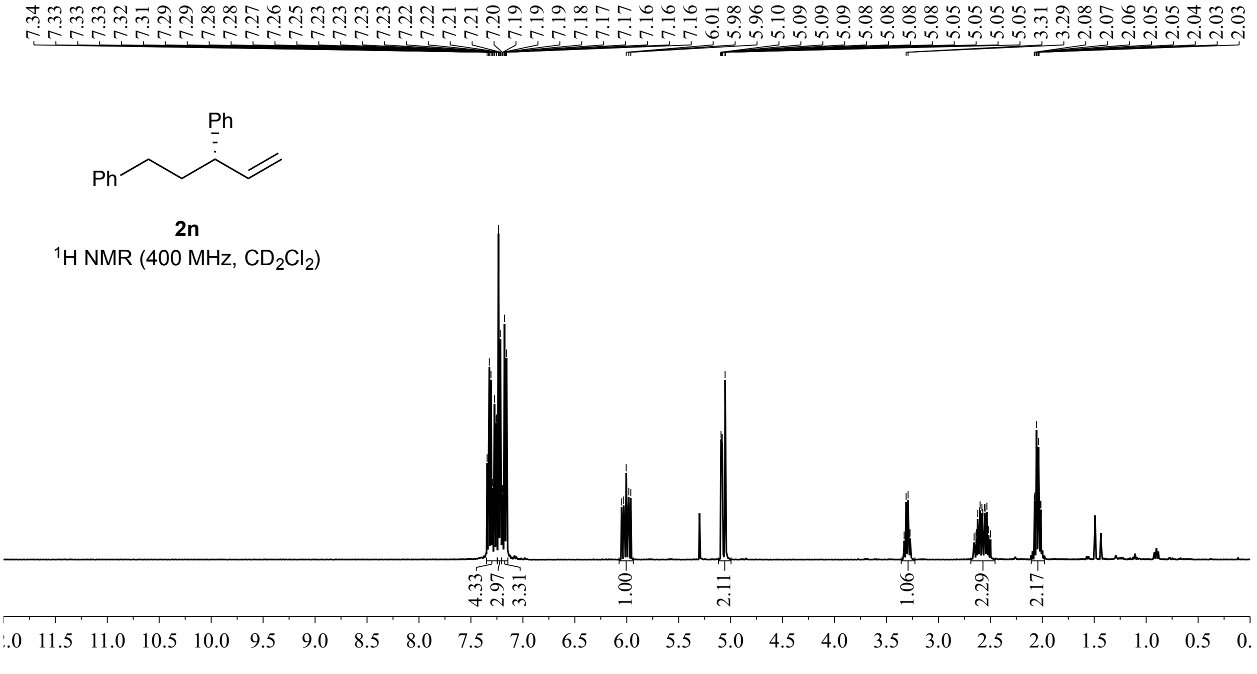

Supplementary Figure 29. ^{13}C NMR spectrum of 2n

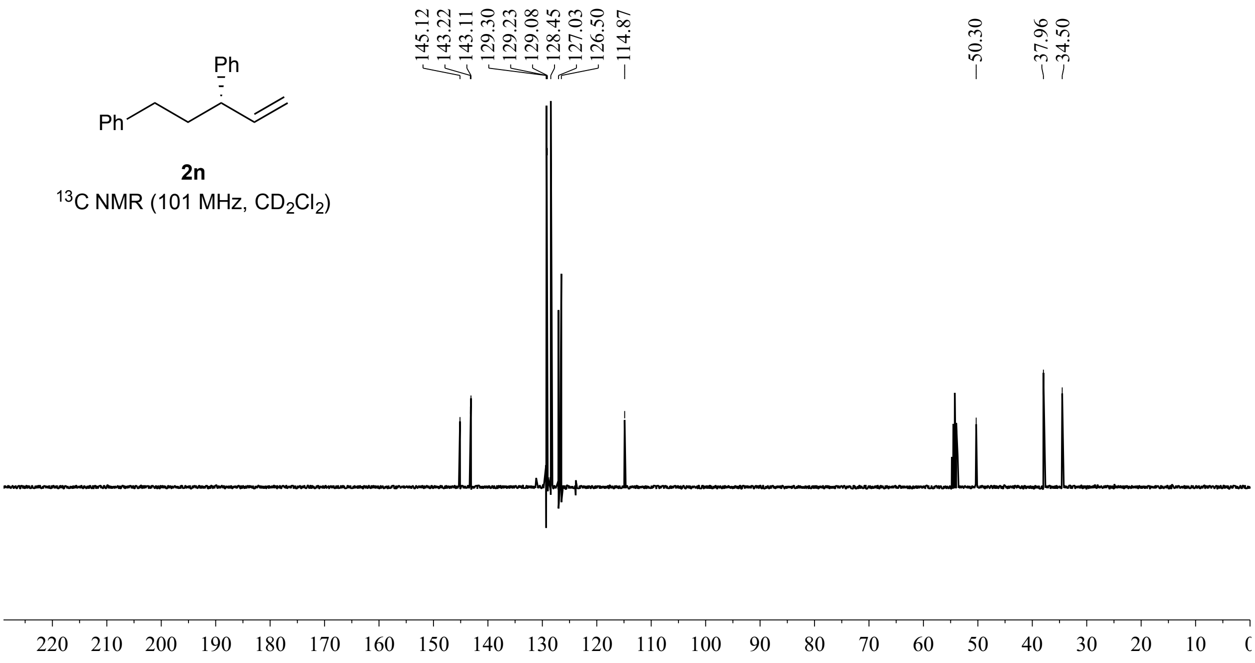

Supplementary Figure 30. ^{1}H NMR spectrum of 2o

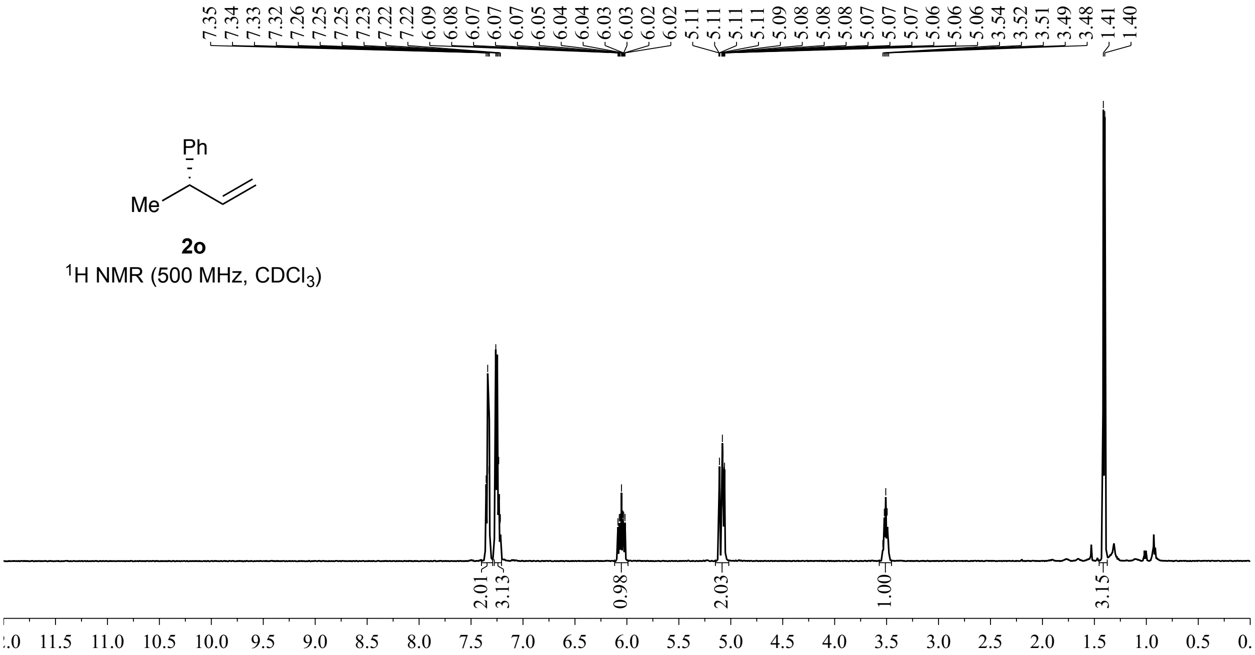

Supplementary Figure 31. ^{13}C NMR spectrum of product 2o

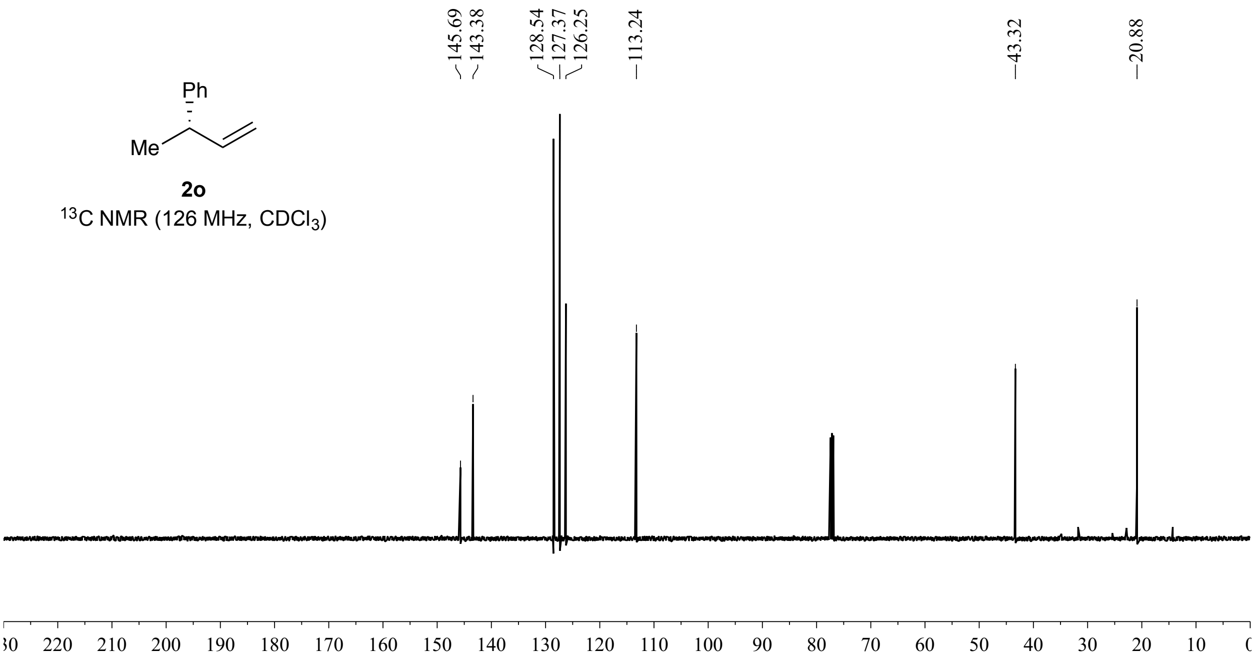

Supplementary Figure 32. ^{1}H NMR spectrum of 2p

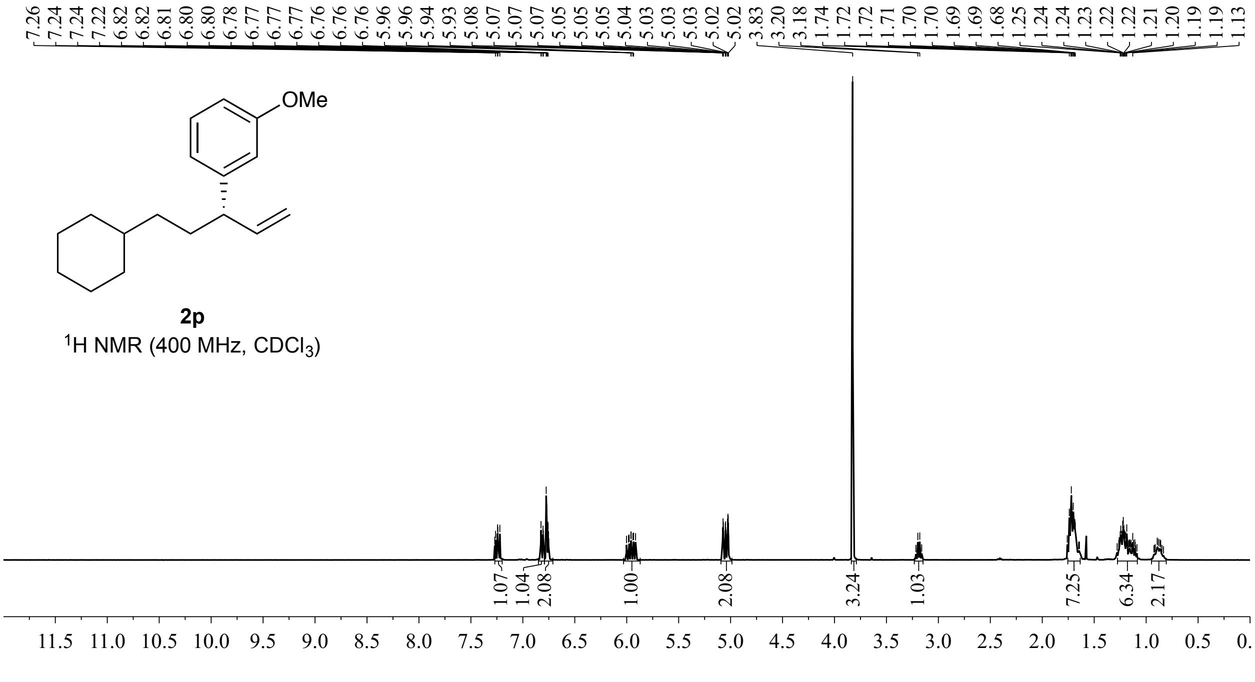

Supplementary Figure 33. ^{13}C NMR spectrum of 2p

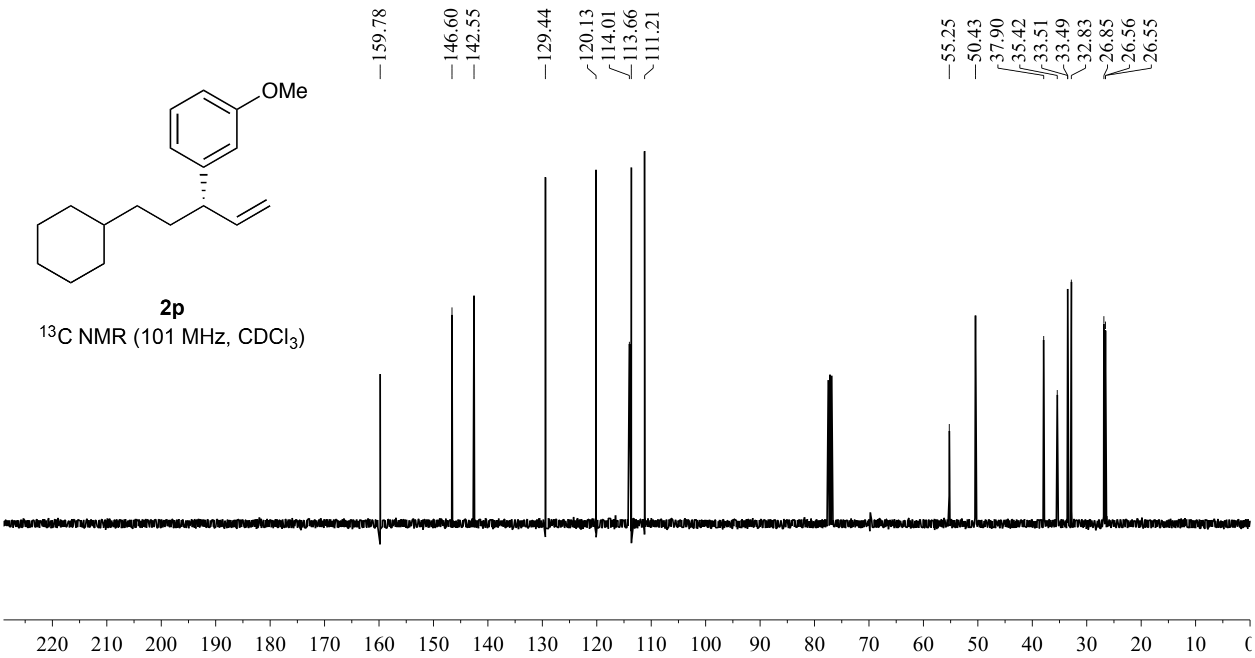

Supplementary Figure 34. ^{1}H NMR spectrum of 2q

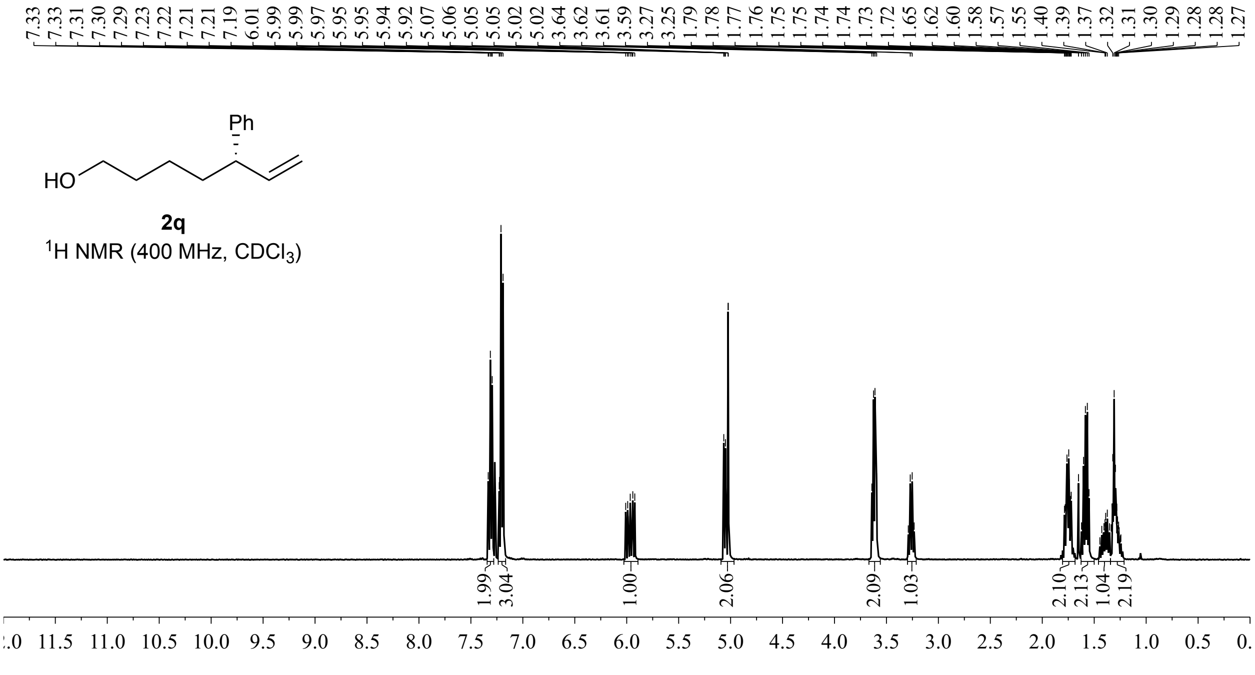

Supplementary Figure 35. ^{13}C NMR spectrum of 2q

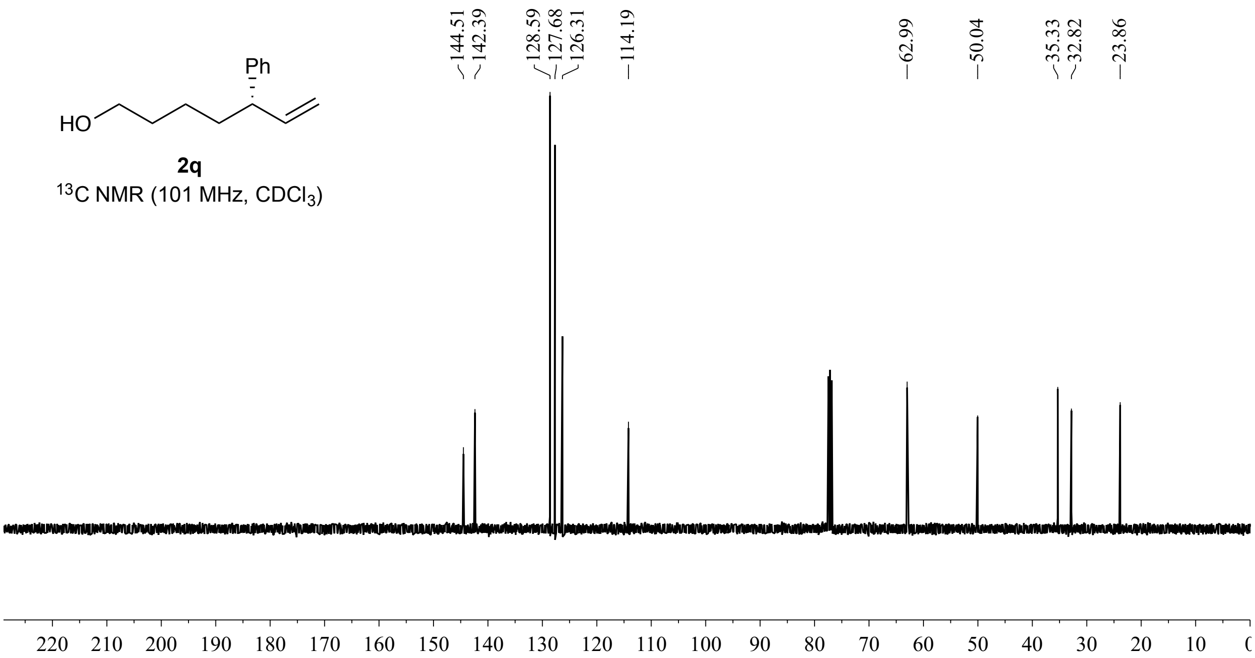

Supplementary Figure 36. ^{1}H NMR spectrum of 2r

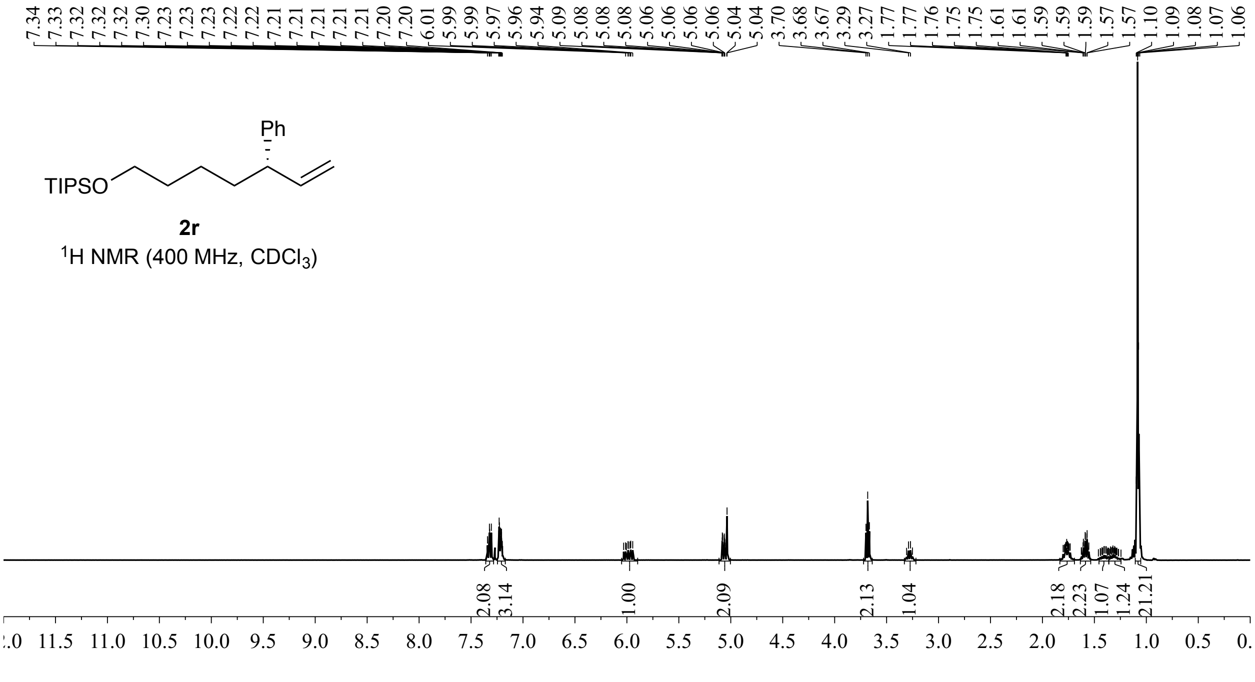

Supplementary Figure 37. ^{13}C NMR spectrum of 2r

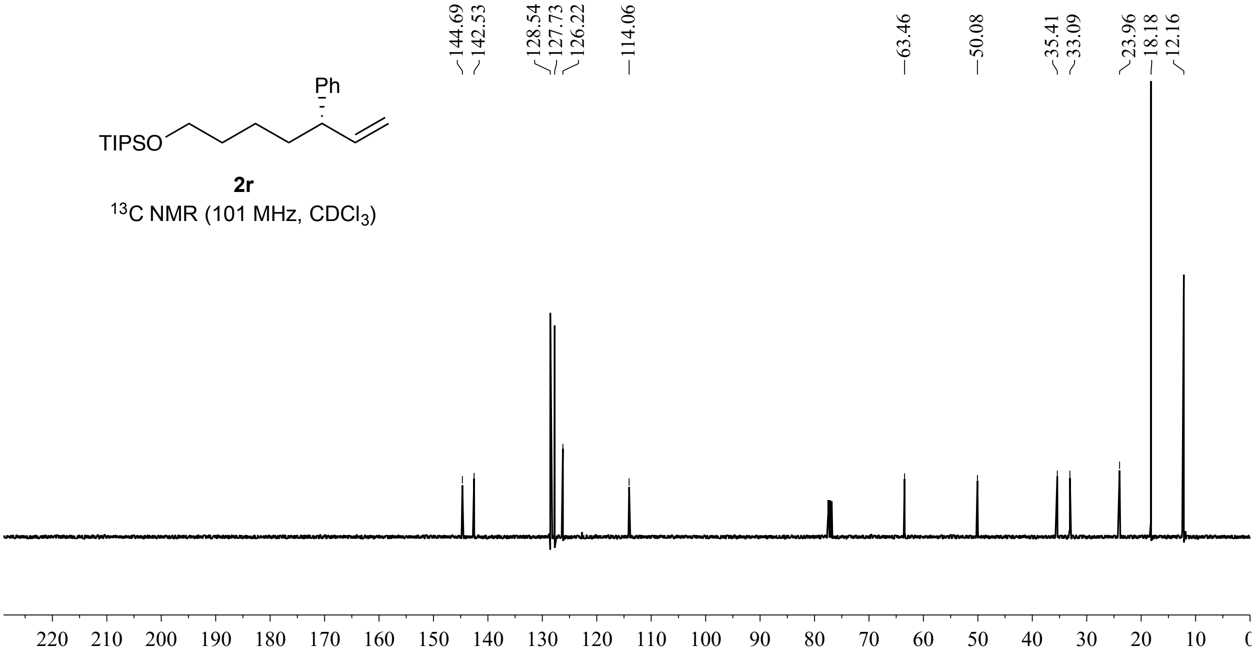

Supplementary Figure 38. ^{1}H NMR spectrum of 2s

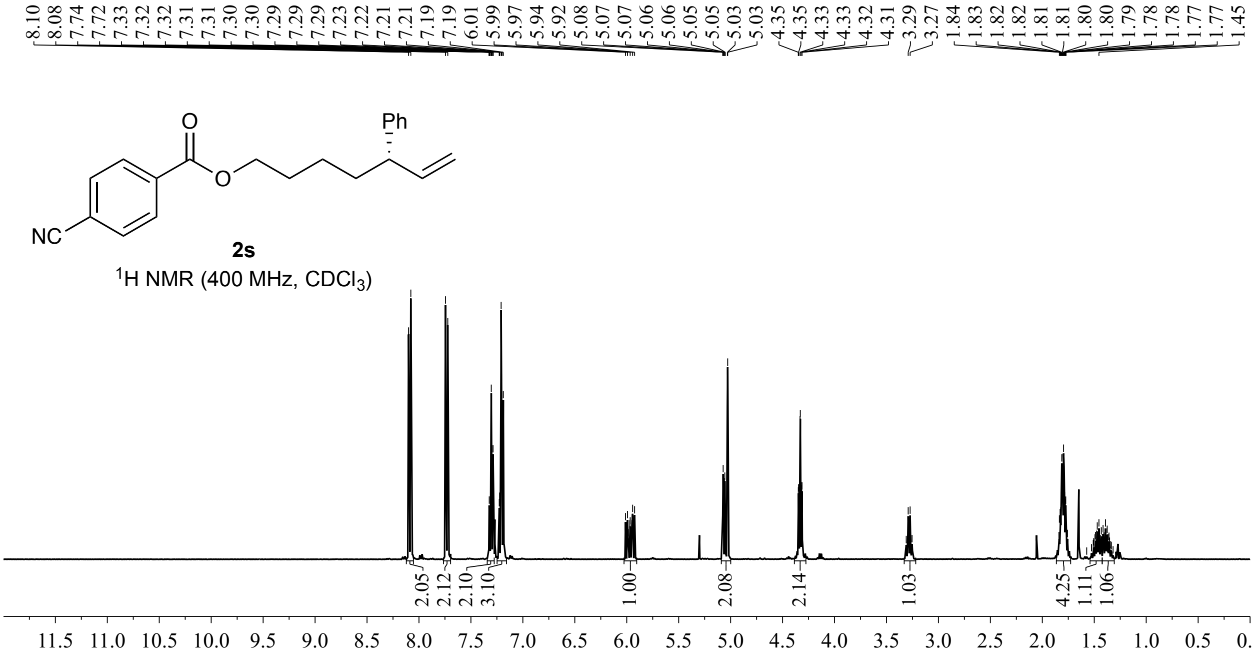

Supplementary Figure 39. ^{13}C NMR spectrum of 2s

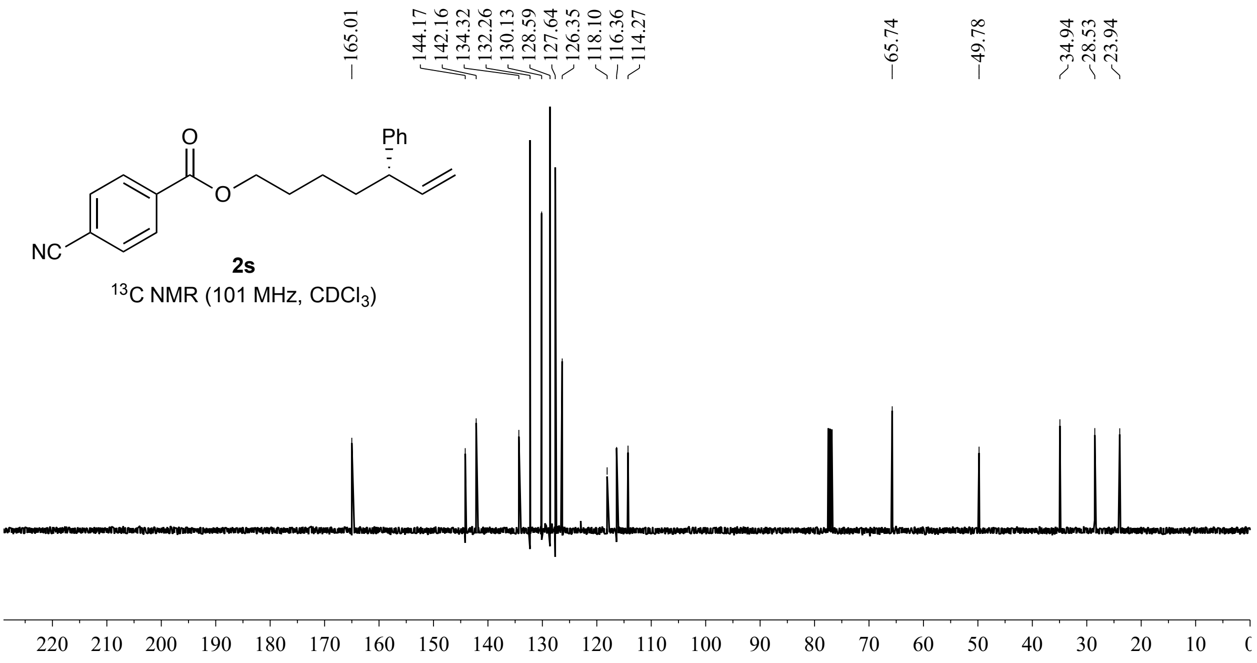

Supplementary Figure 40. ^{1}H NMR spectrum of s1a

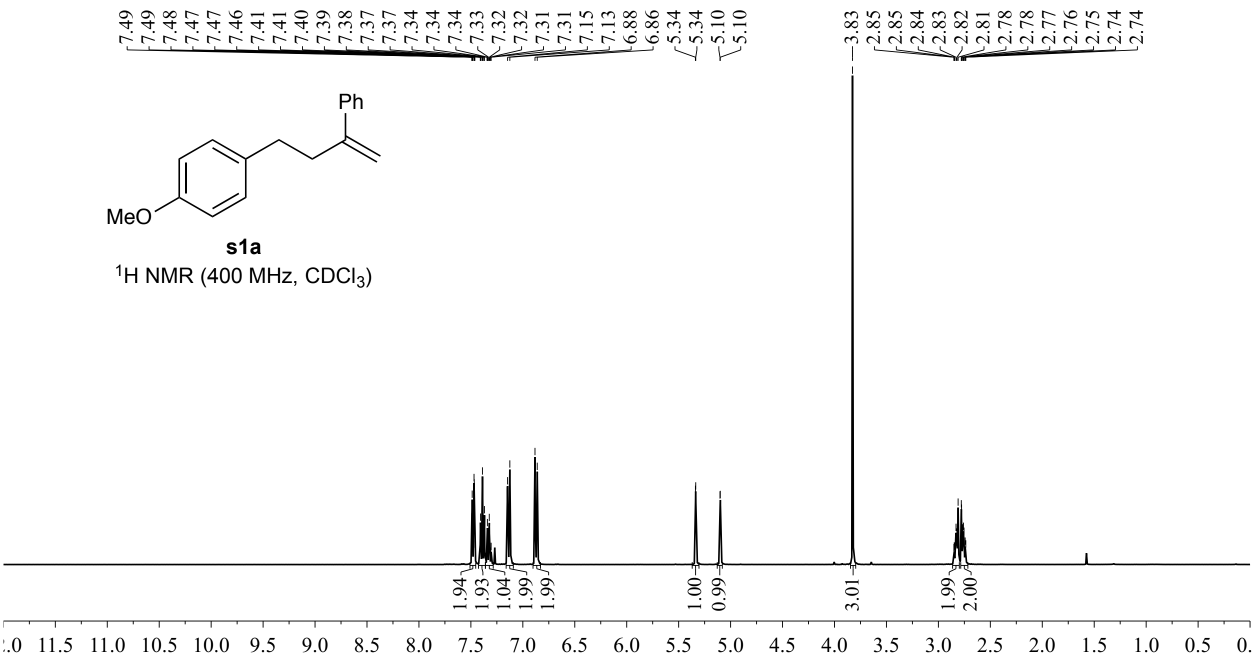

Supplementary Figure 41. ^{13}C NMR spectrum of s1a

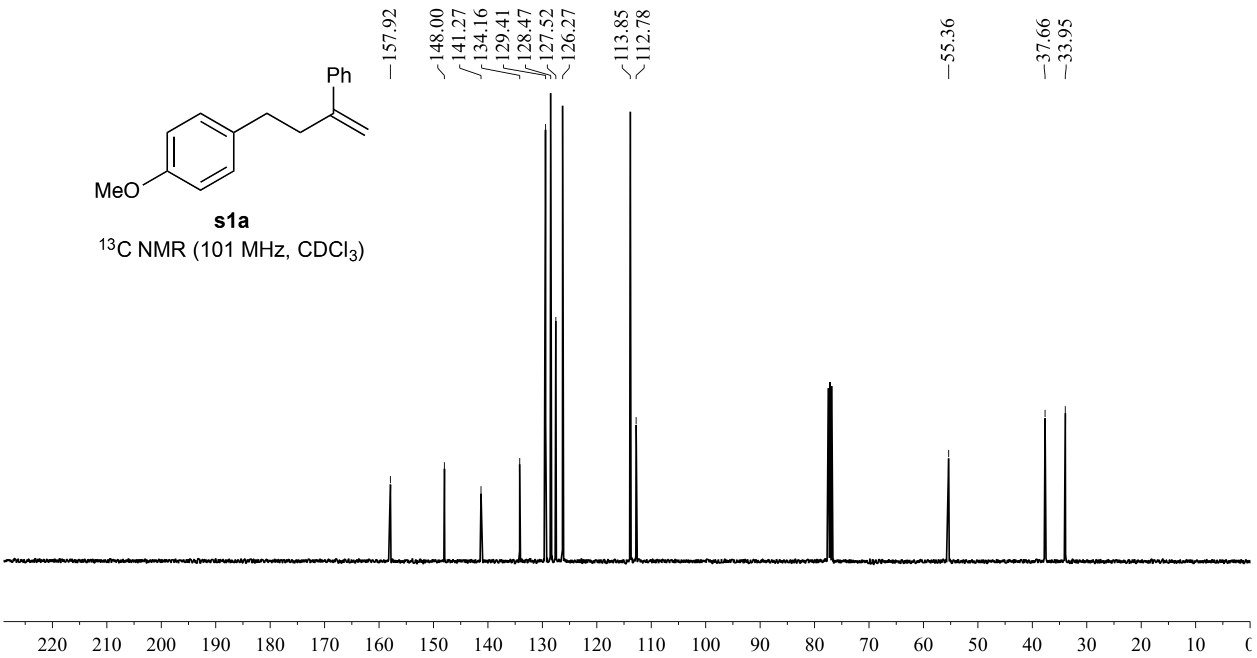

Supplementary Figure 42. ^{1}H NMR spectrum of s1b

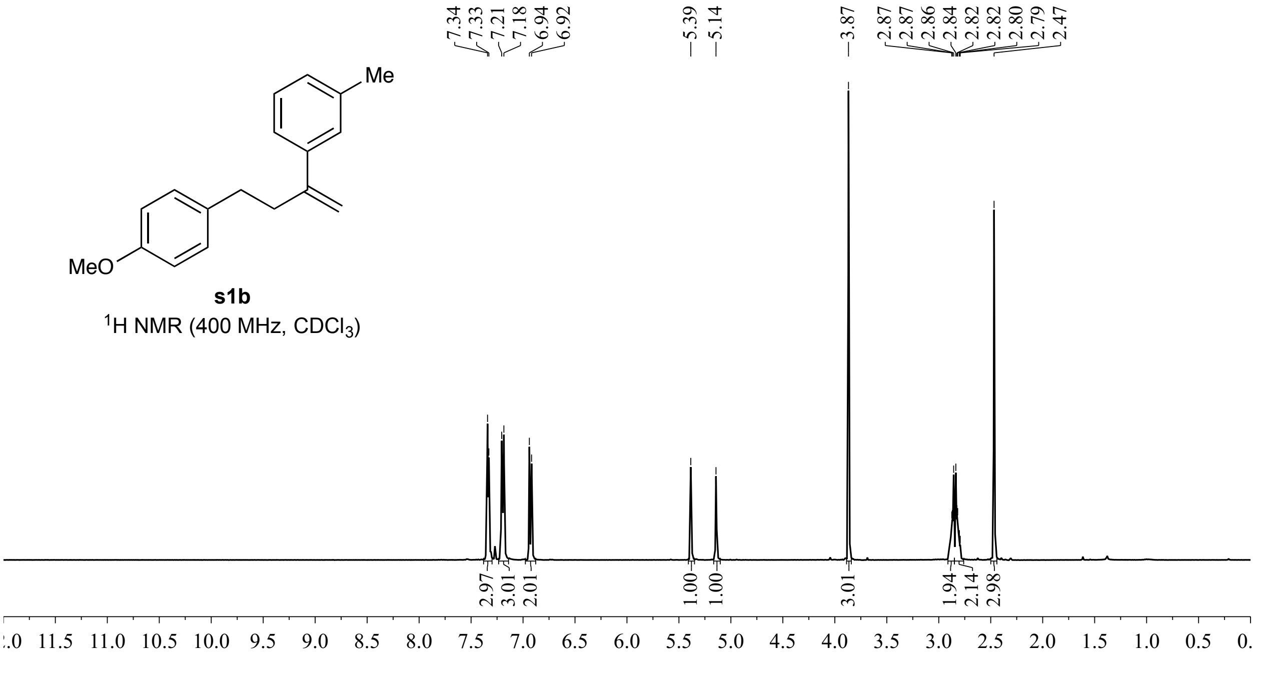

Supplementary Figure 43. ^{13}C NMR spectrum of s1b

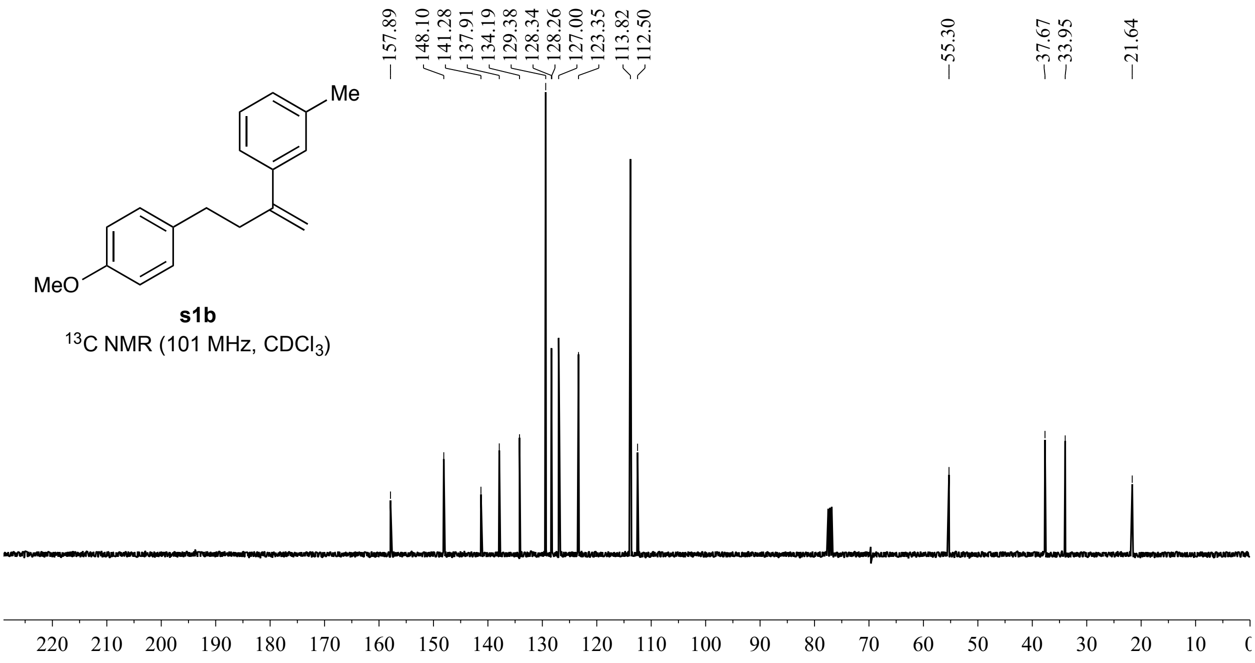

Supplementary Figure 44. ^{1}H NMR spectrum of s1c

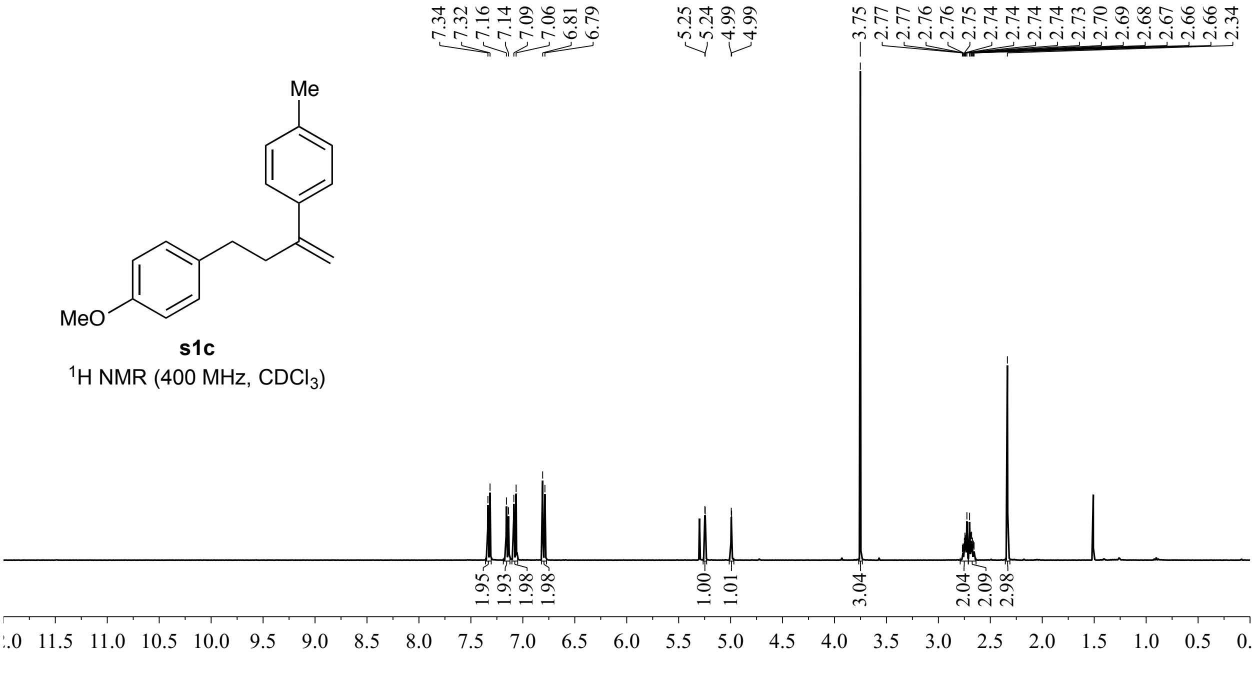

Supplementary Figure 45. ^{13}C NMR spectrum of s1c

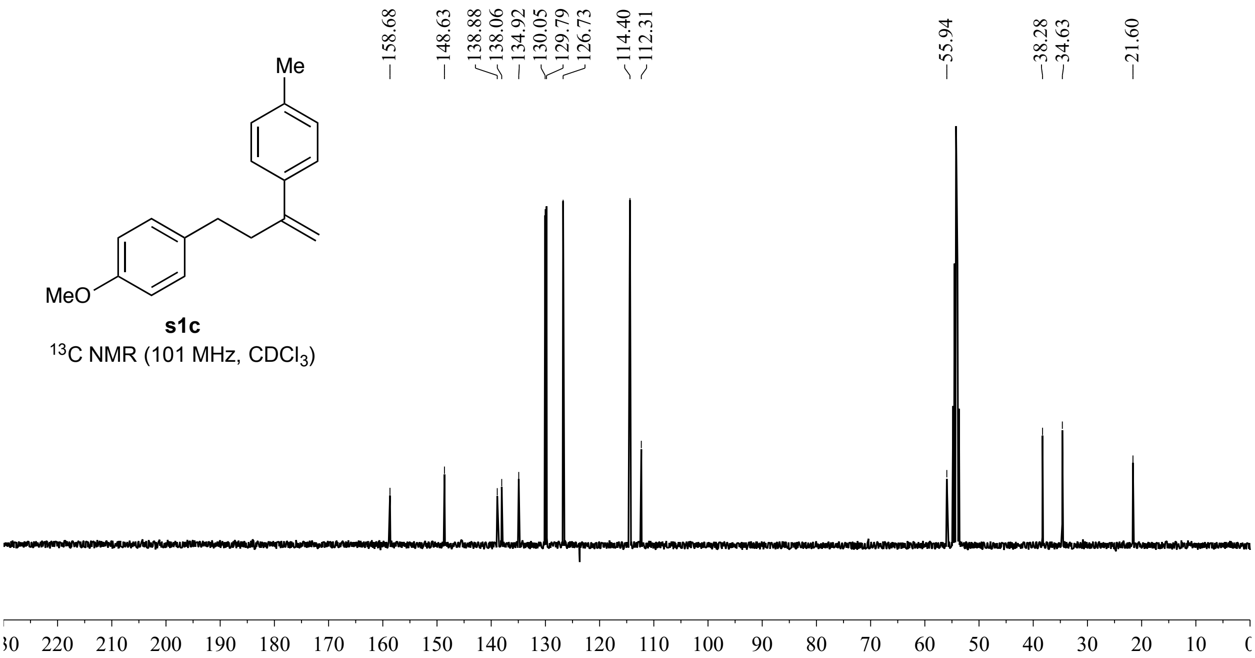

Supplementary Figure 46. ^{1}H NMR spectrum of s1d

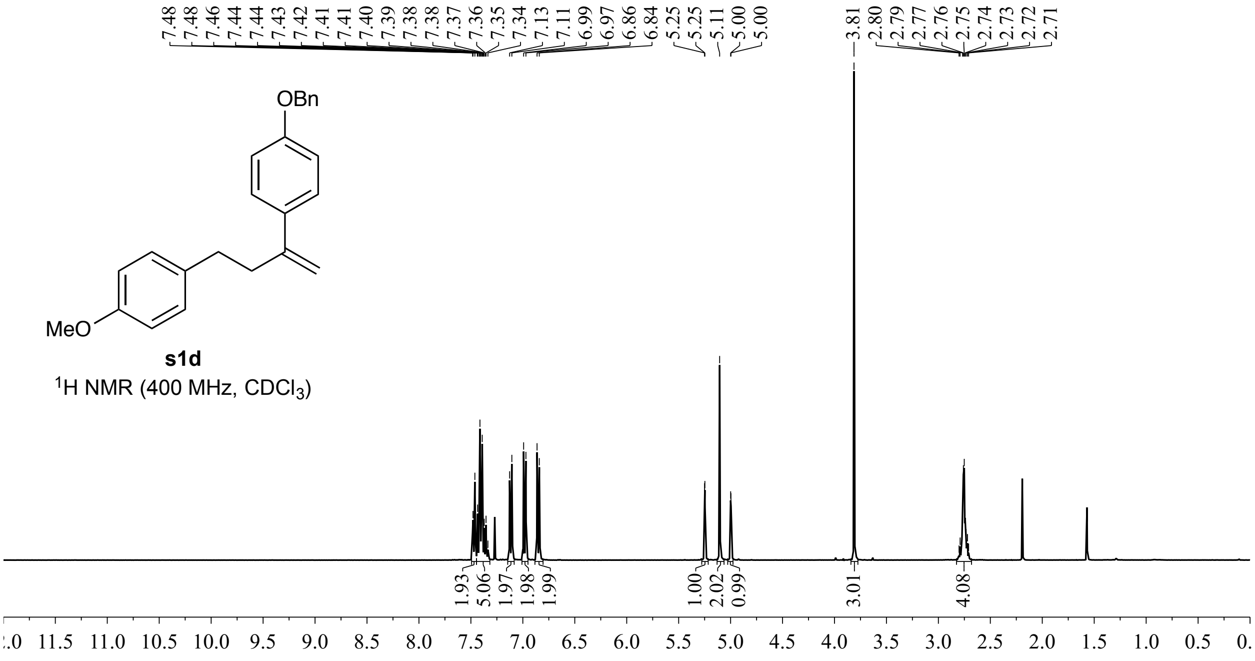

Supplementary Figure 47. ^{13}C NMR spectrum of s1d

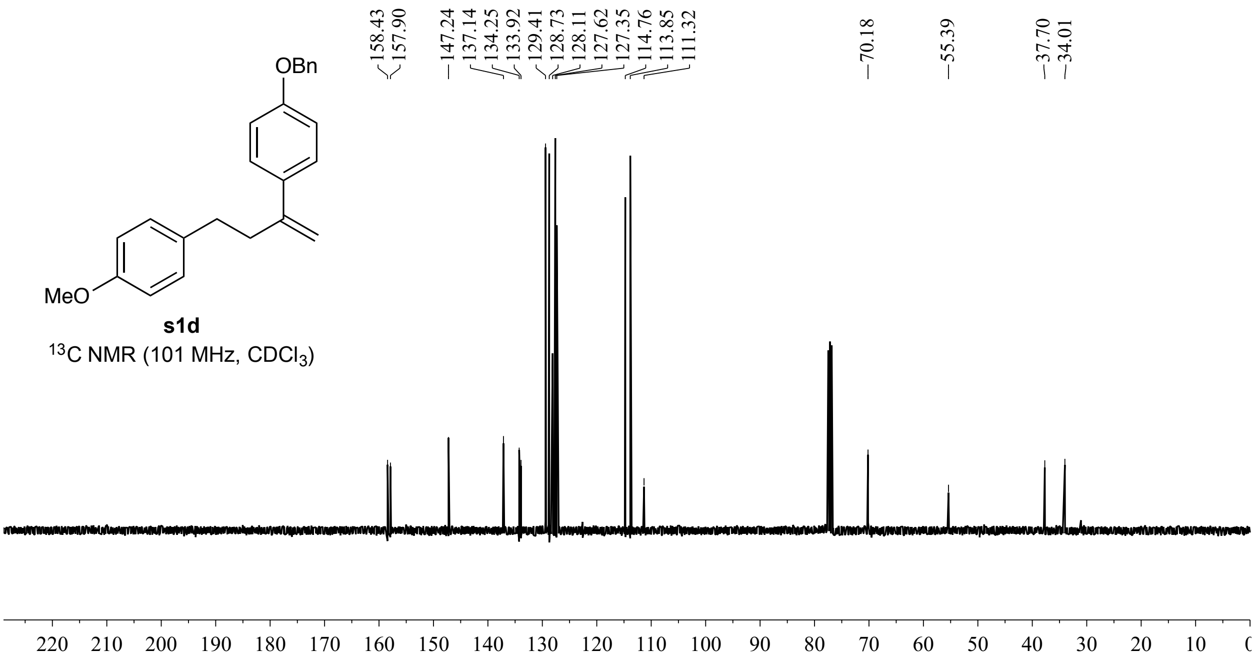

Supplementary Figure 48. ^{1}H NMR spectrum of s1e

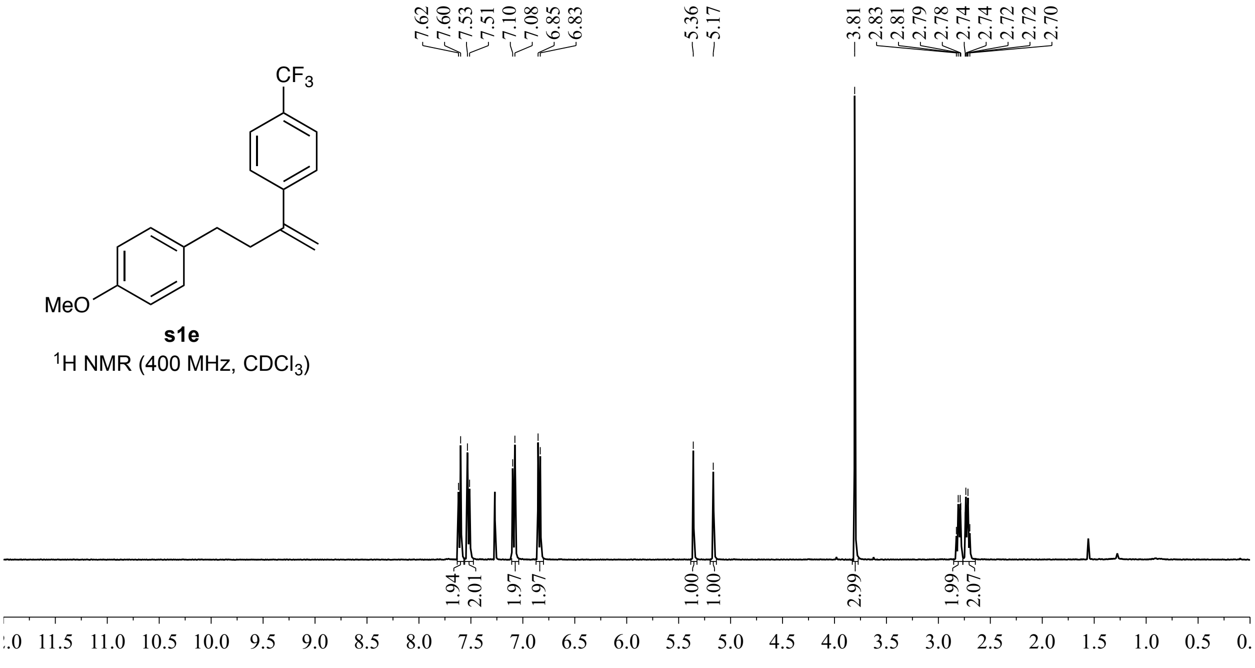

Supplementary Figure 49. ^{13}C NMR spectrum of s1e

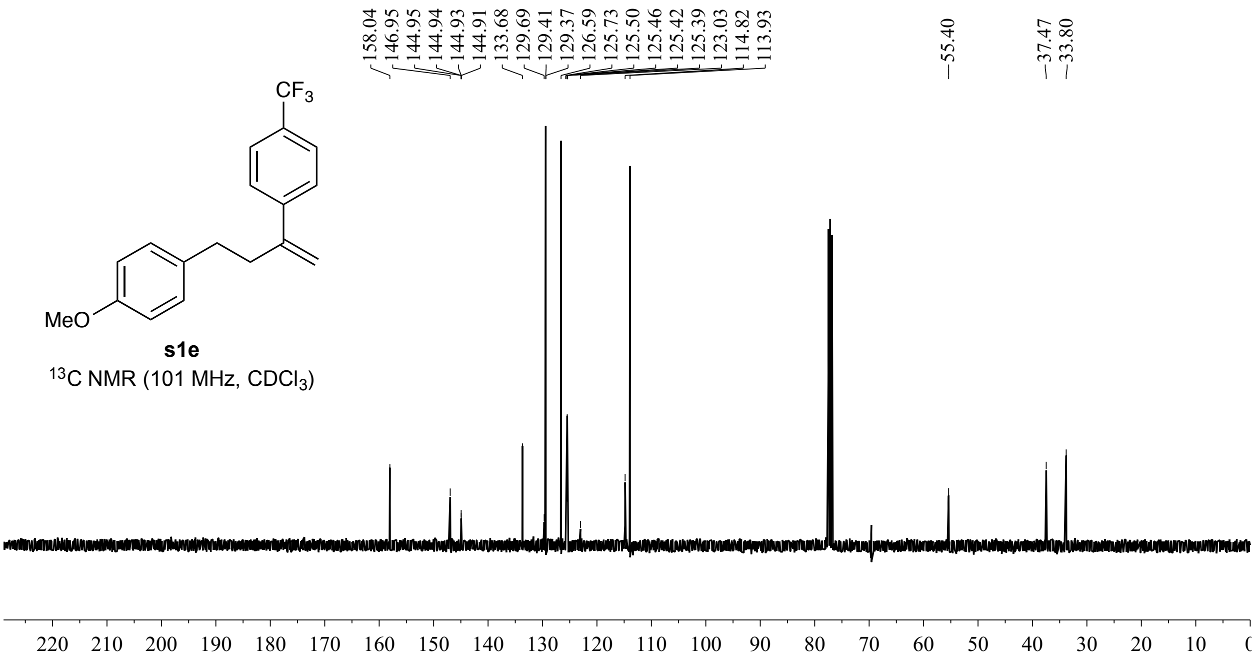

Supplementary Figure 50. ^{19}F NMR spectrum of s1e

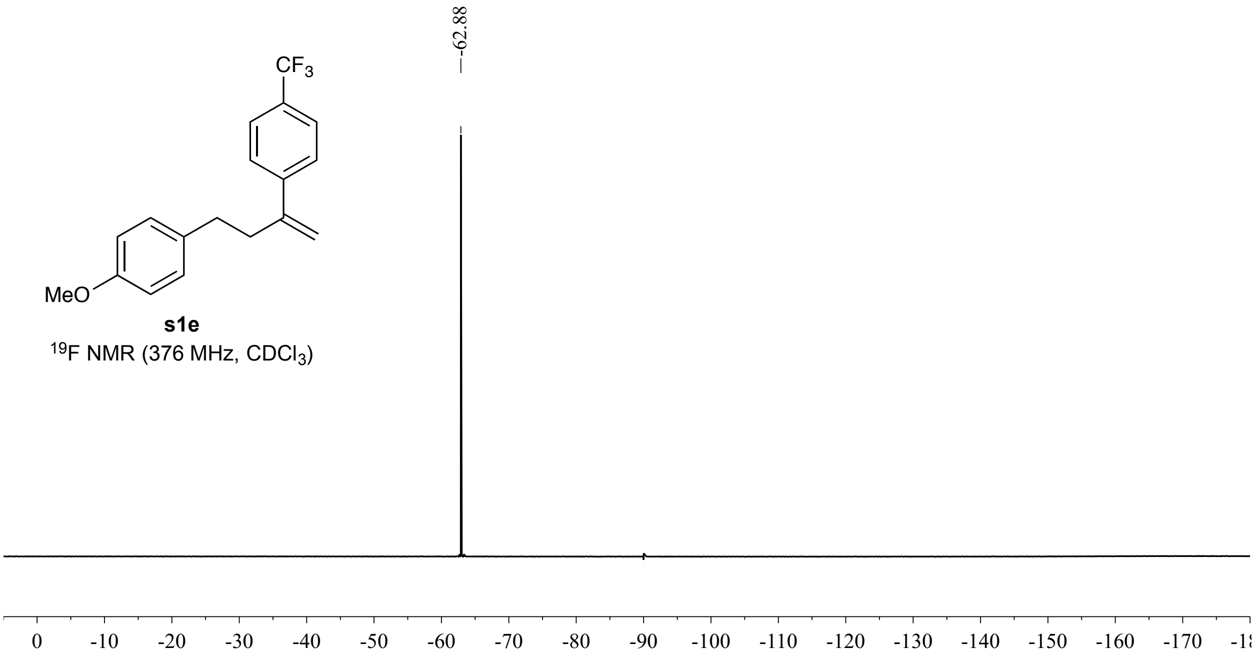

Supplementary Figure 51. ^{1}H NMR spectrum of s1f

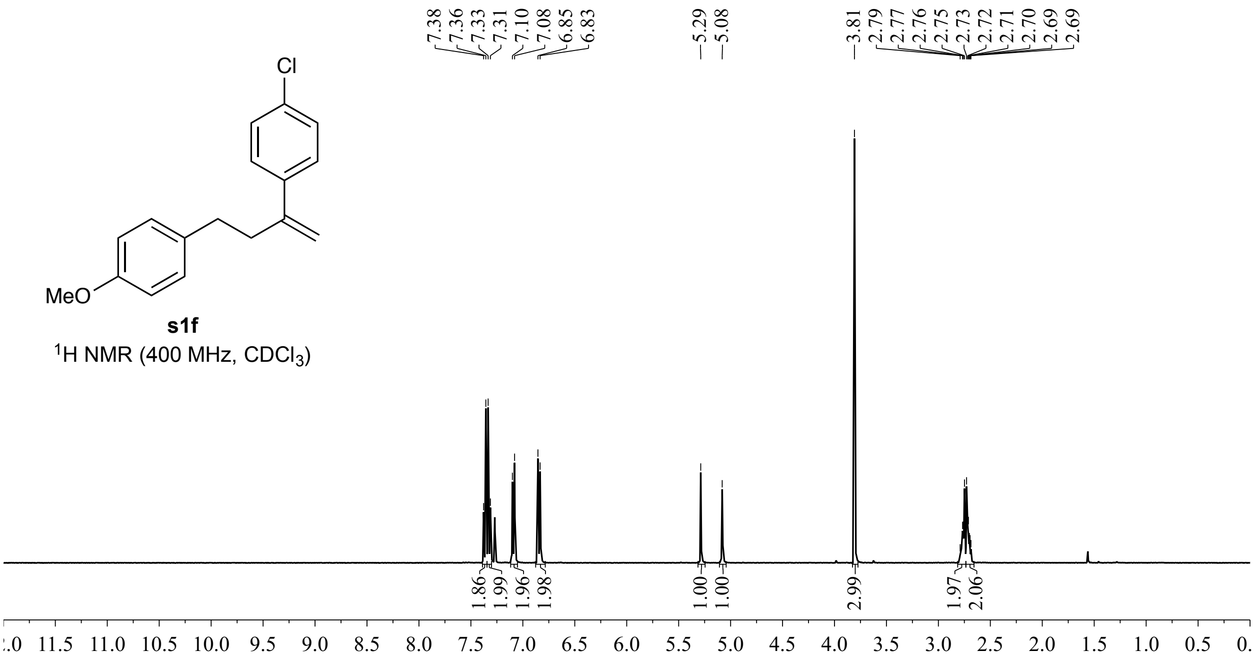

Supplementary Figure 52. ^{13}C NMR spectrum of s1f

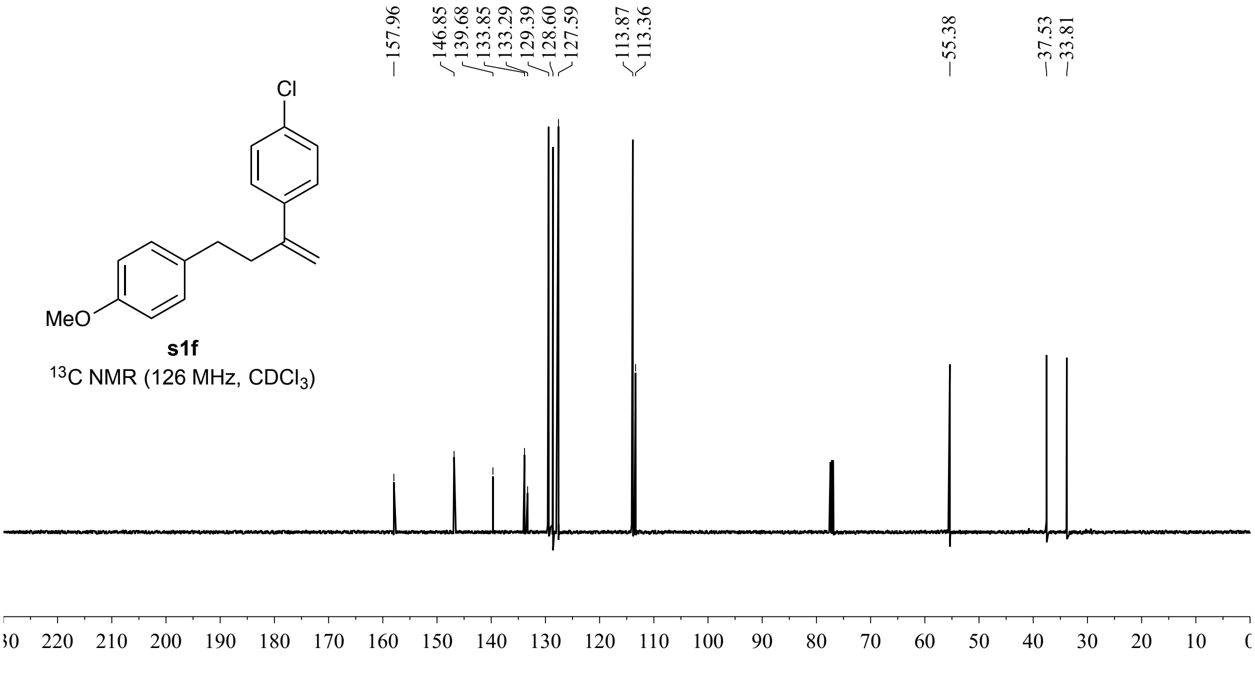

Supplementary Figure 53. ^{1}H NMR spectrum of s1g

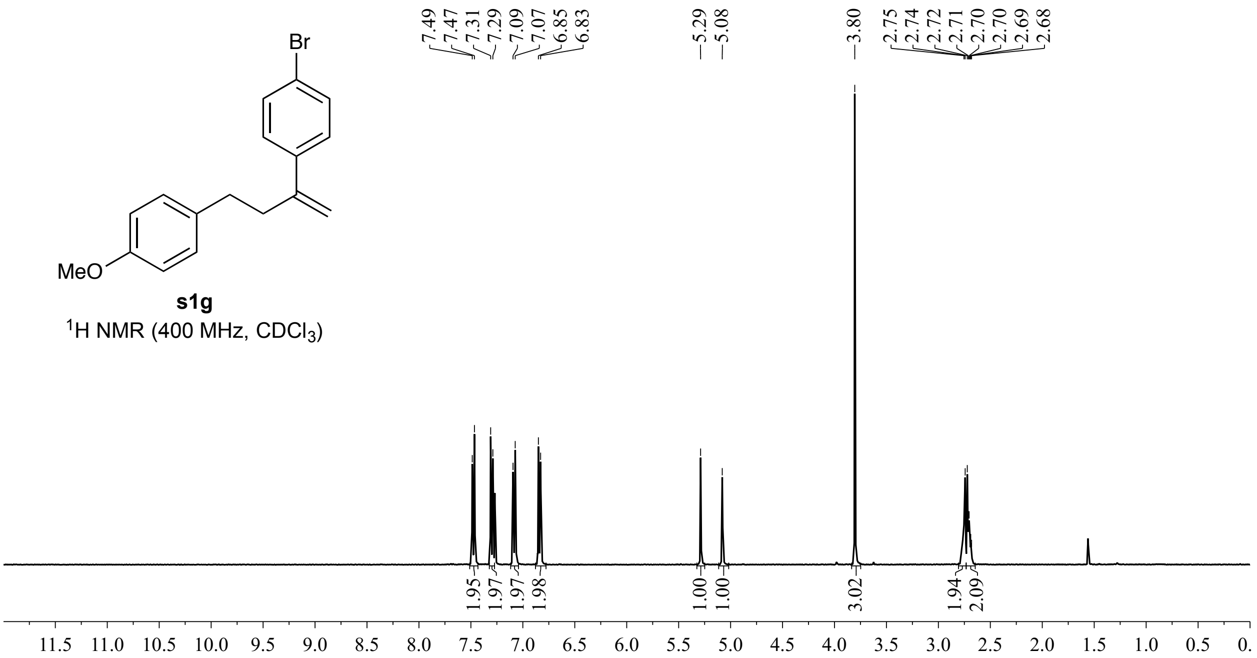

Supplementary Figure 54. ^{13}C NMR spectrum of s1g

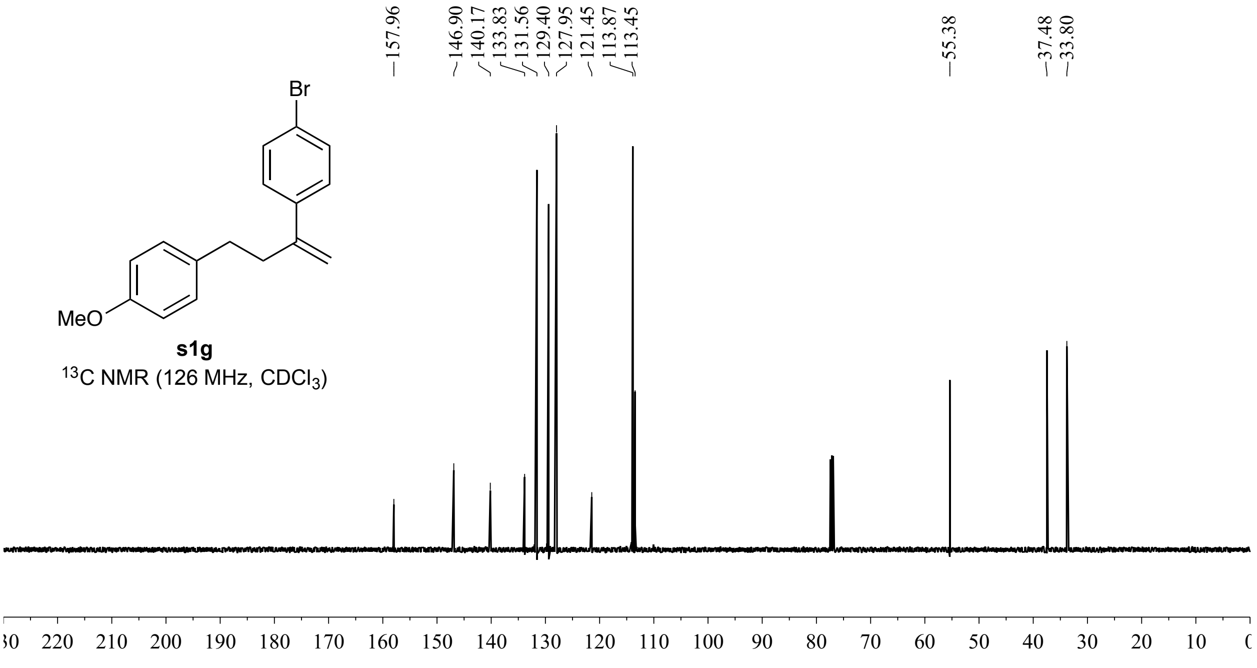

Supplementary Figure 55. ^{1}H NMR spectrum of s1h

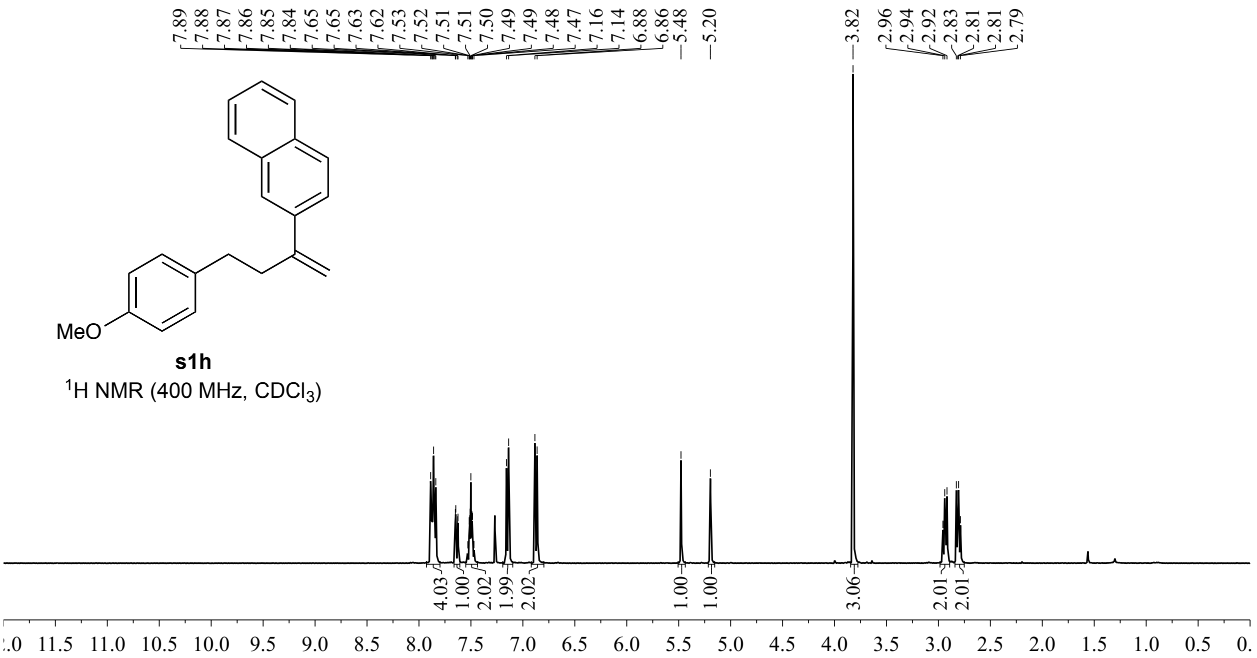

Supplementary Figure 56. ^{13}C NMR spectrum of s1h

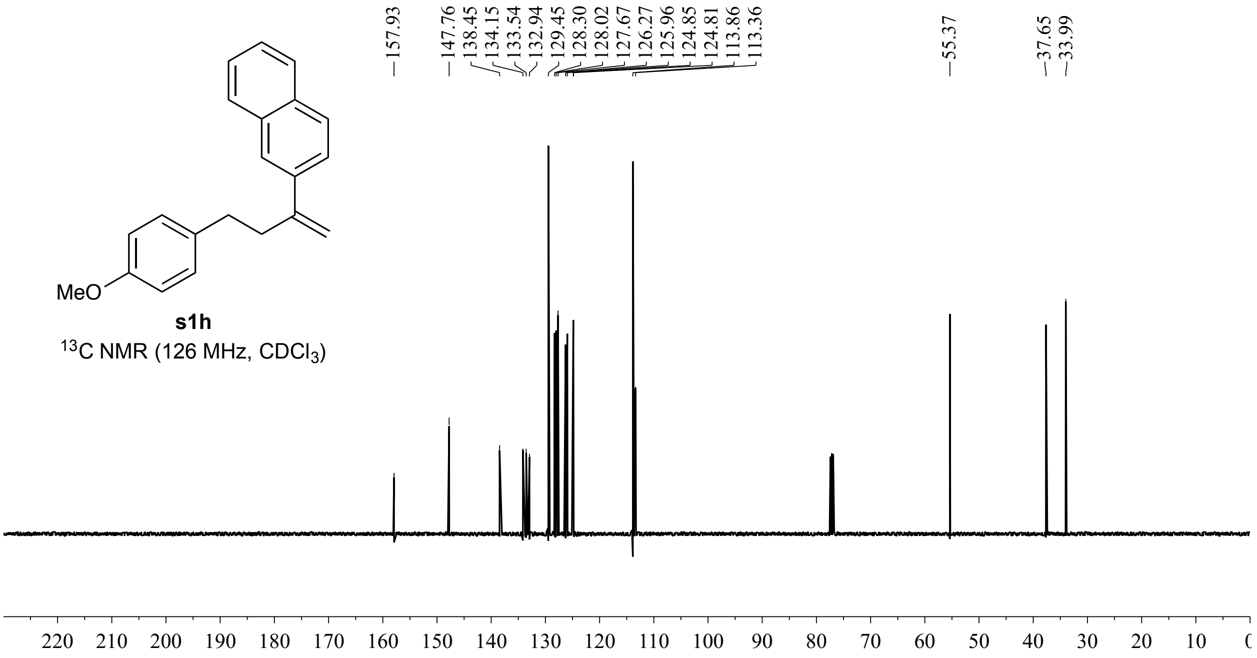

Supplementary Figure 57. ^{1}H NMR spectrum of s1i

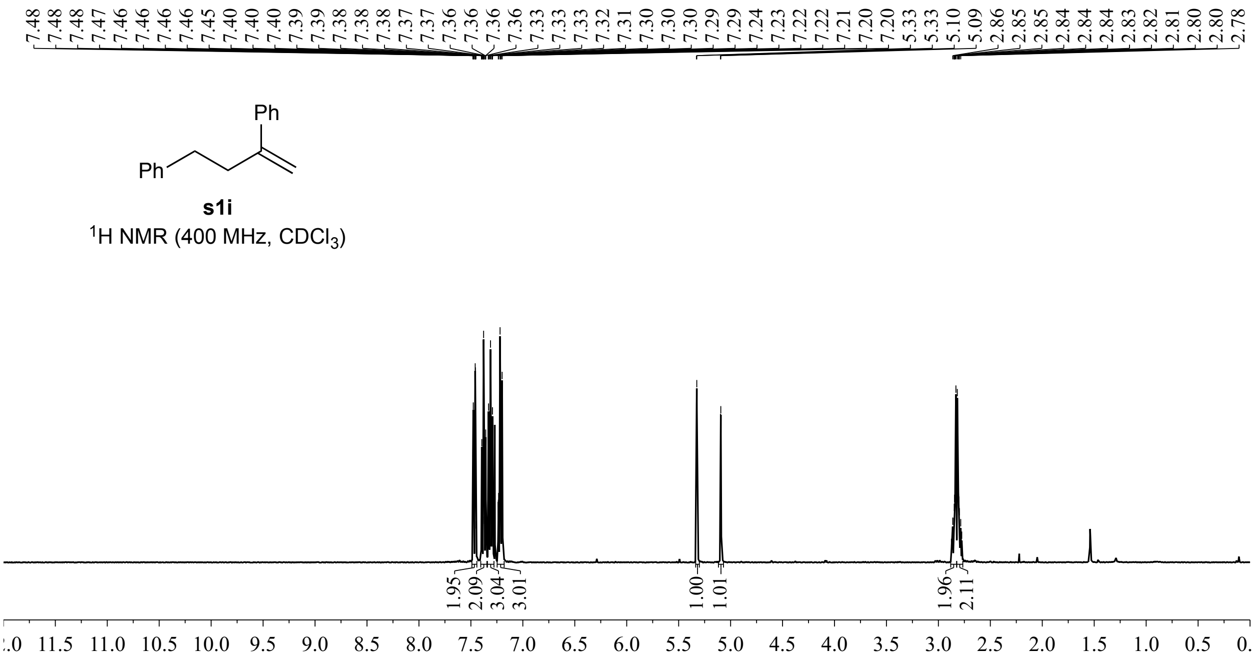

Supplementary Figure 58. ^{1}H NMR spectrum of s2a

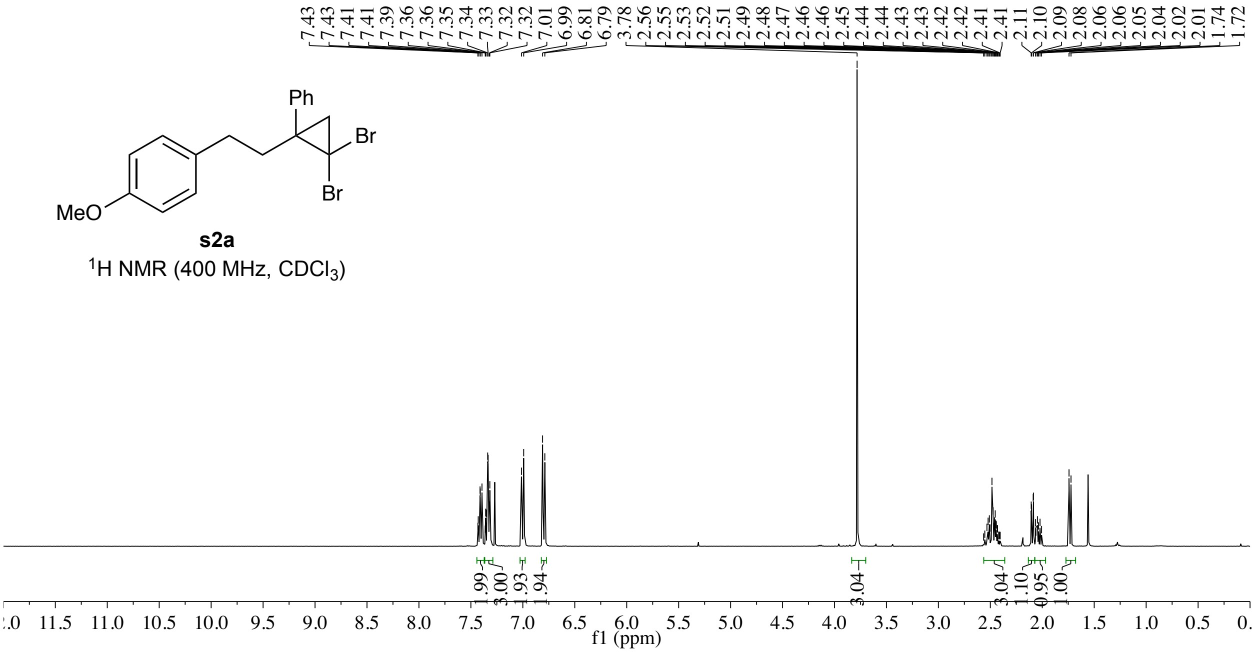

Supplementary Figure 59. ^{13}C NMR spectrum of s2a

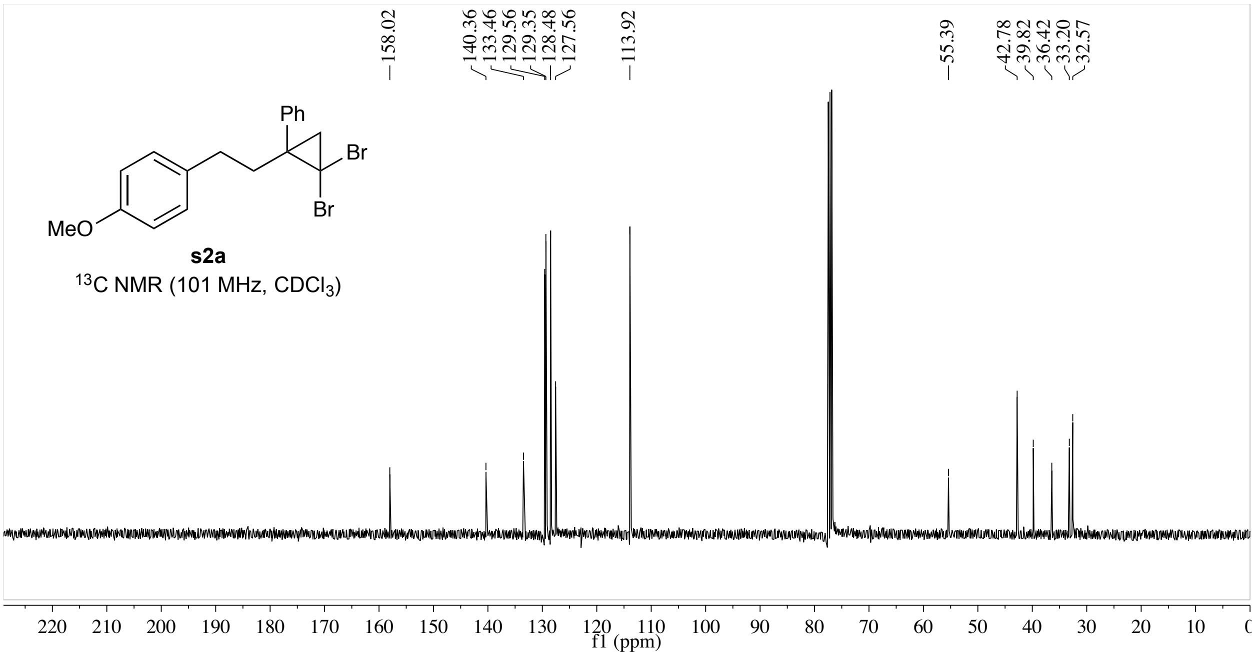

Supplementary Figure 60. ^{1}H NMR spectrum of s2b

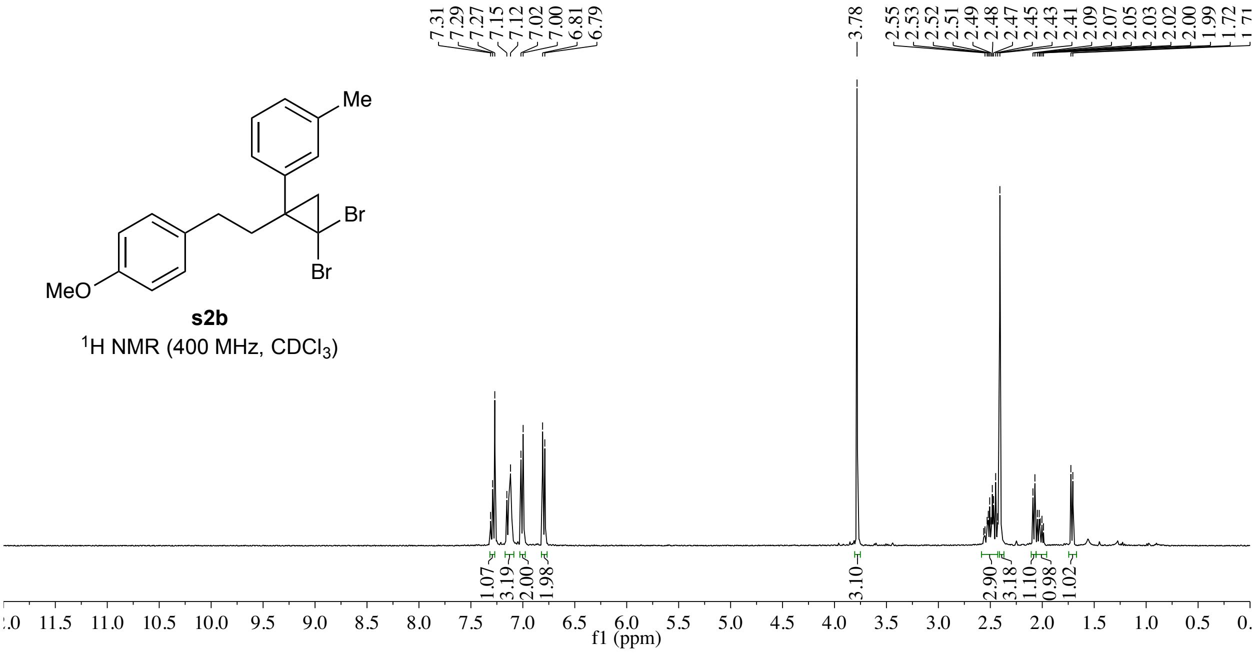

Supplementary Figure 61. ^{13}C NMR spectrum of s2b

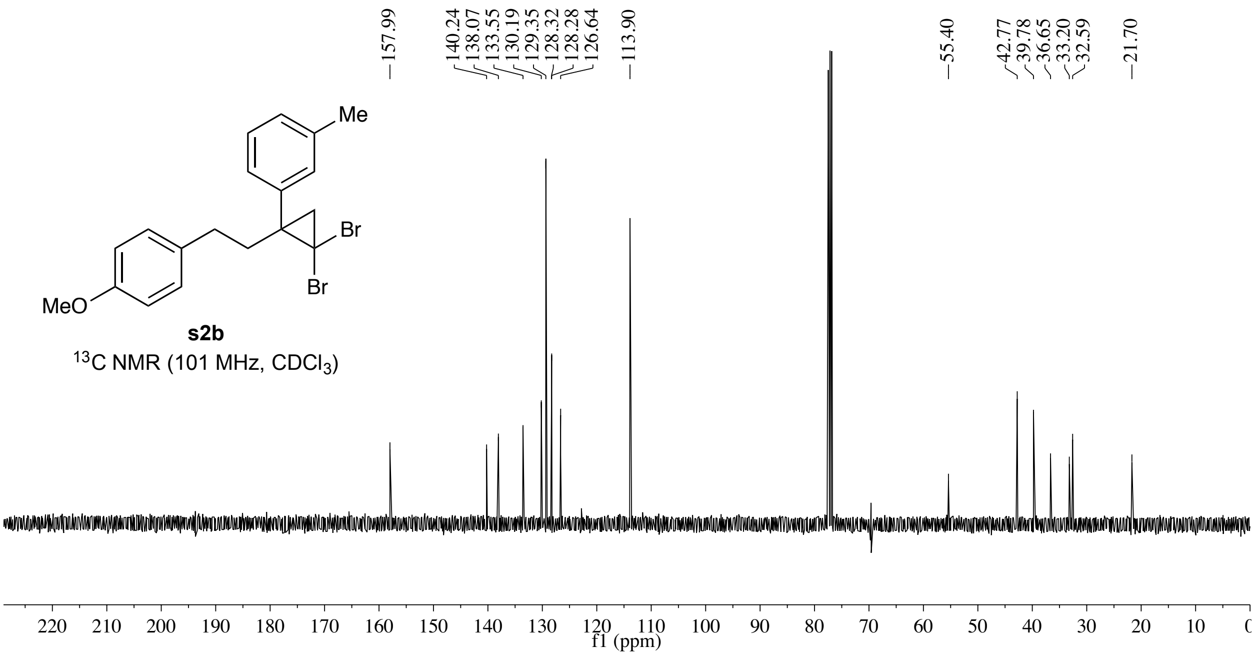

Supplementary Figure 62. ^{1}H NMR spectrum of s2c

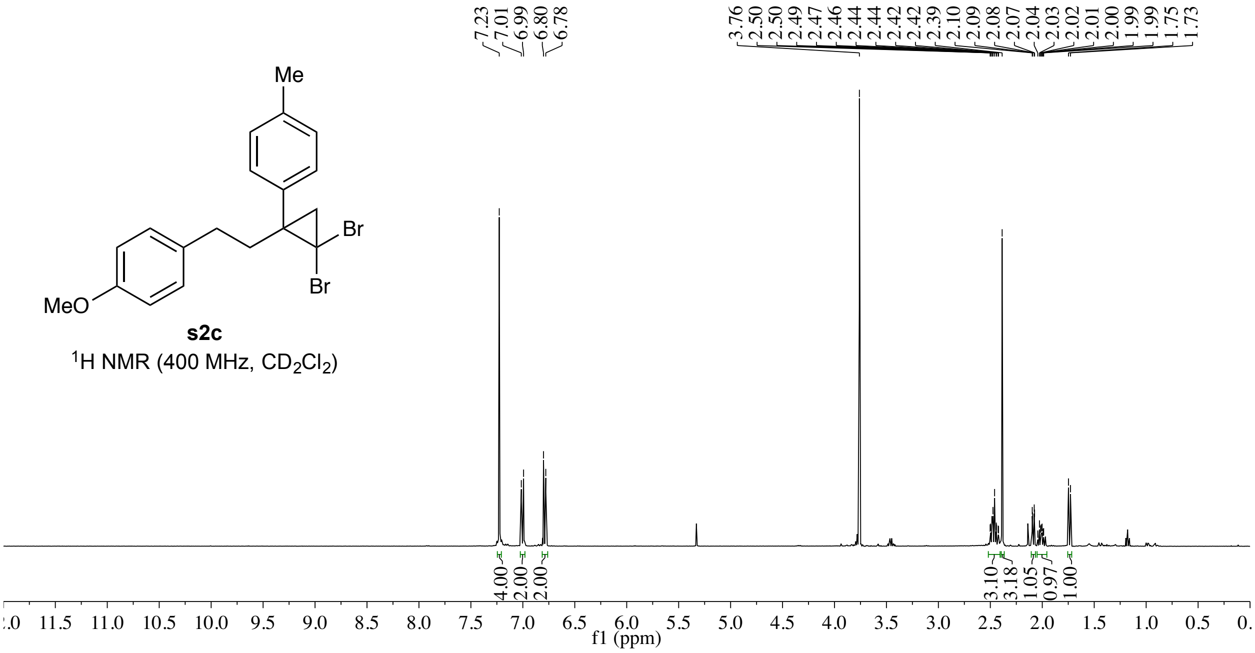

Supplementary Figure 63. ^{13}C NMR spectrum of s2c

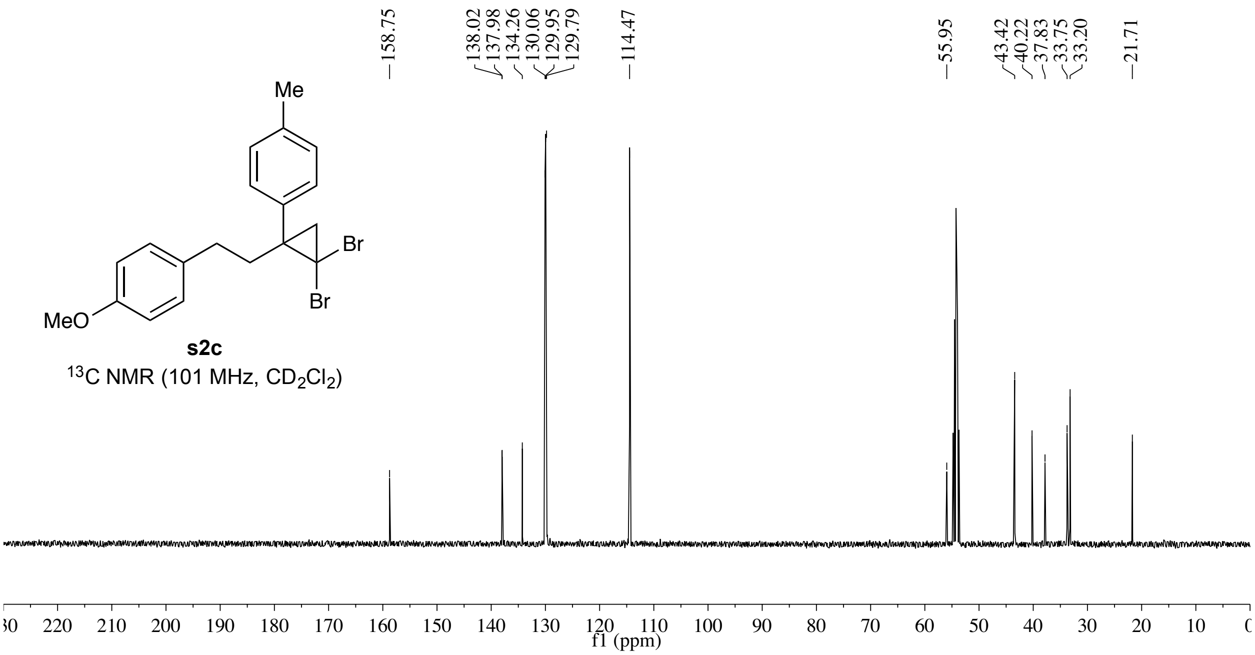

Supplementary Figure 64. ^{1}H NMR spectrum of s2d

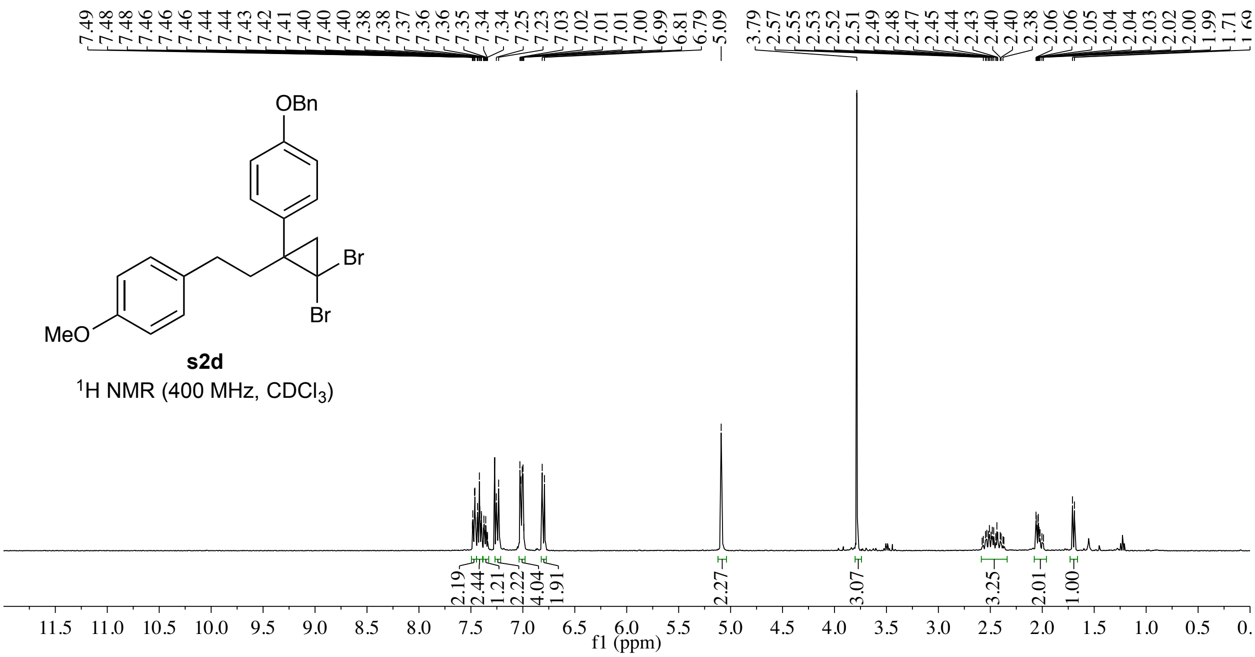

Supplementary Figure 65. ^{13}C NMR spectrum of s2d

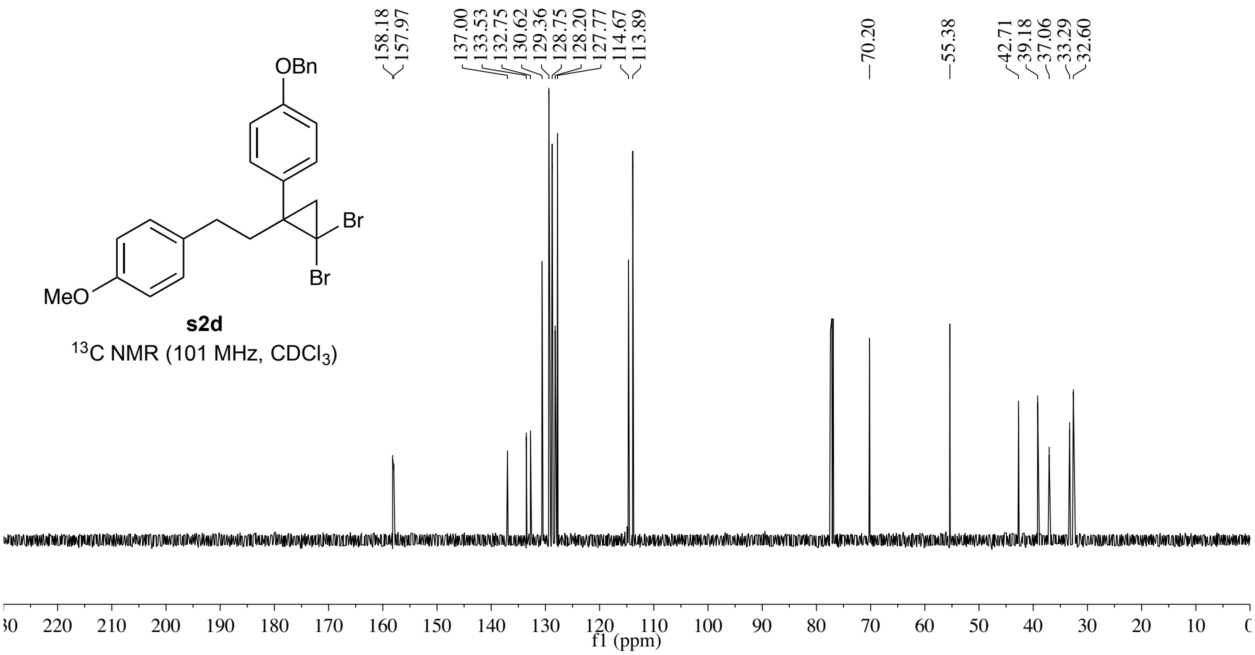

Supplementary Figure 66. ^{1}H NMR spectrum of s2e

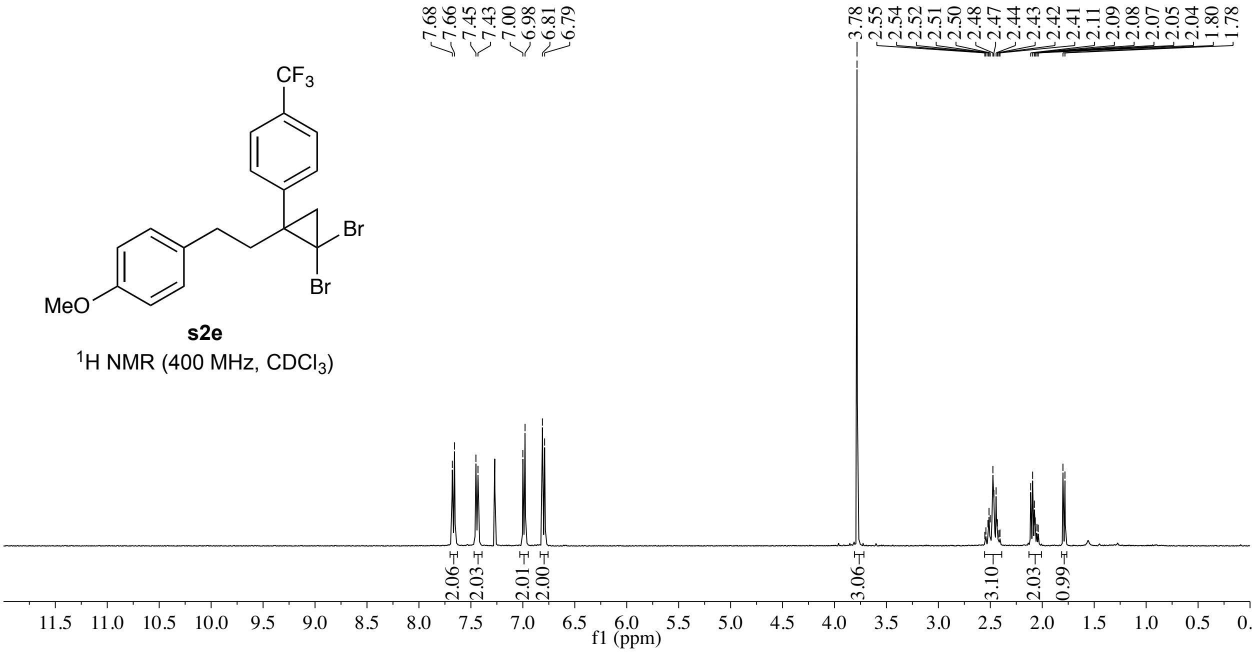

Supplementary Figure 67. ^{13}C NMR spectrum of s2e

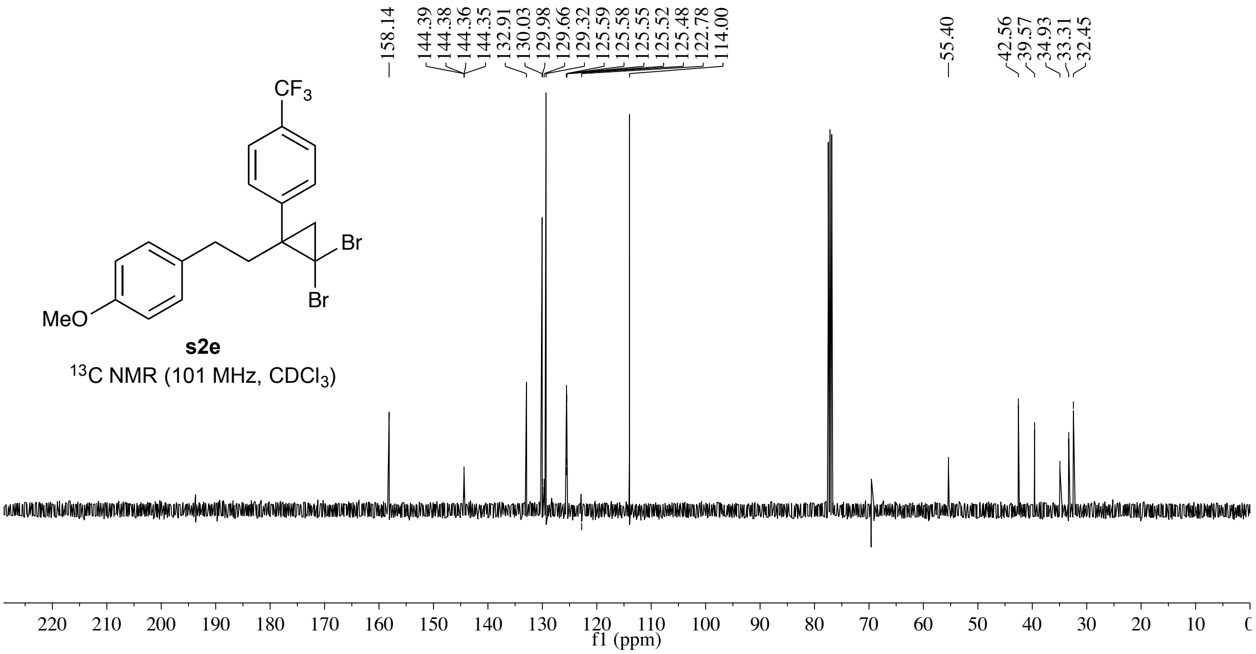

Supplementary Figure 68. ^{19}F NMR spectrum of s2e

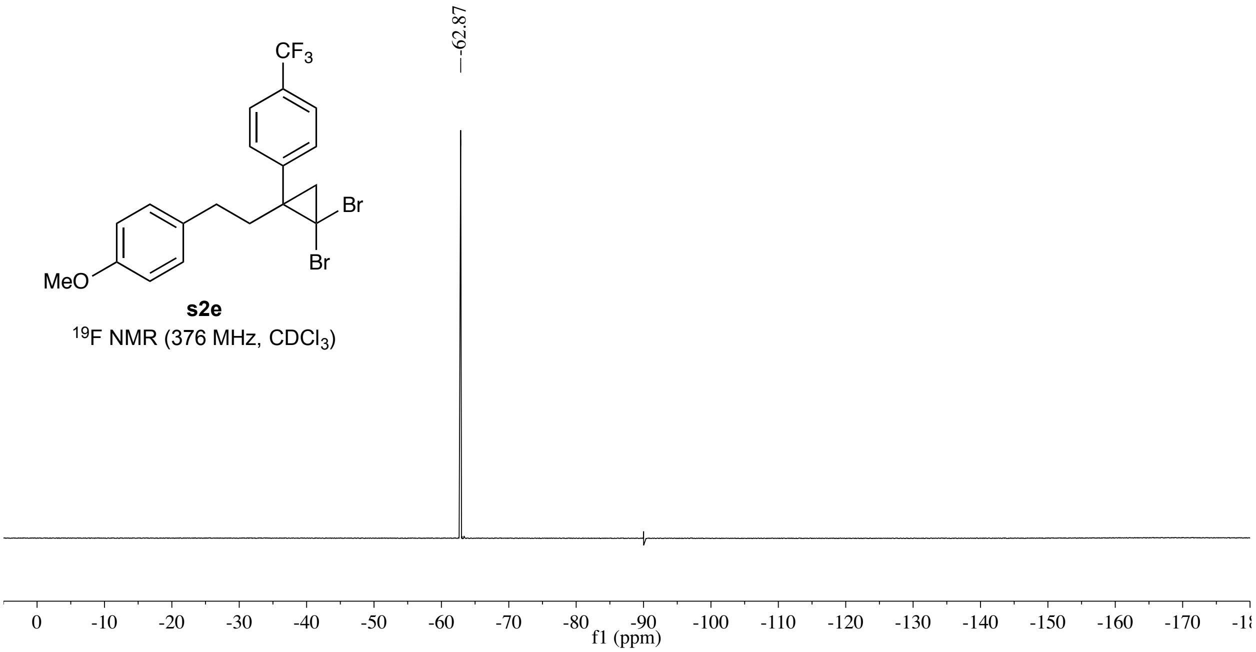

Supplementary Figure 69. ^{1}H NMR spectrum of s2f

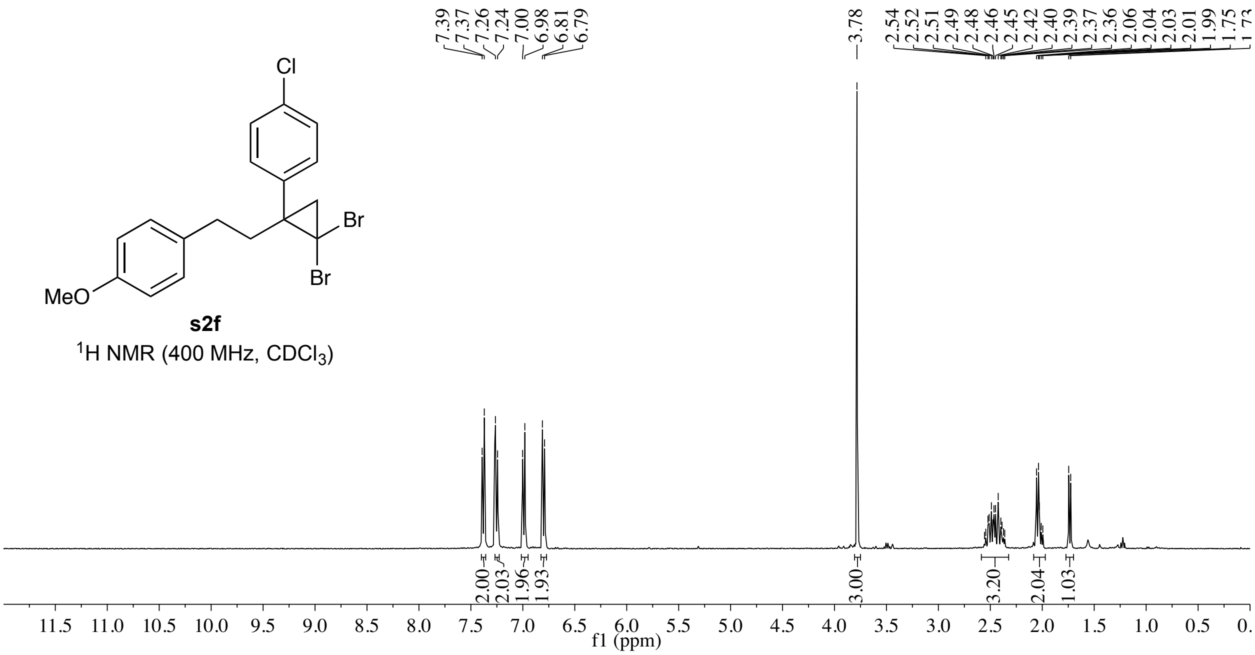

Supplementary Figure 70. ^{13}C NMR spectrum of s2f

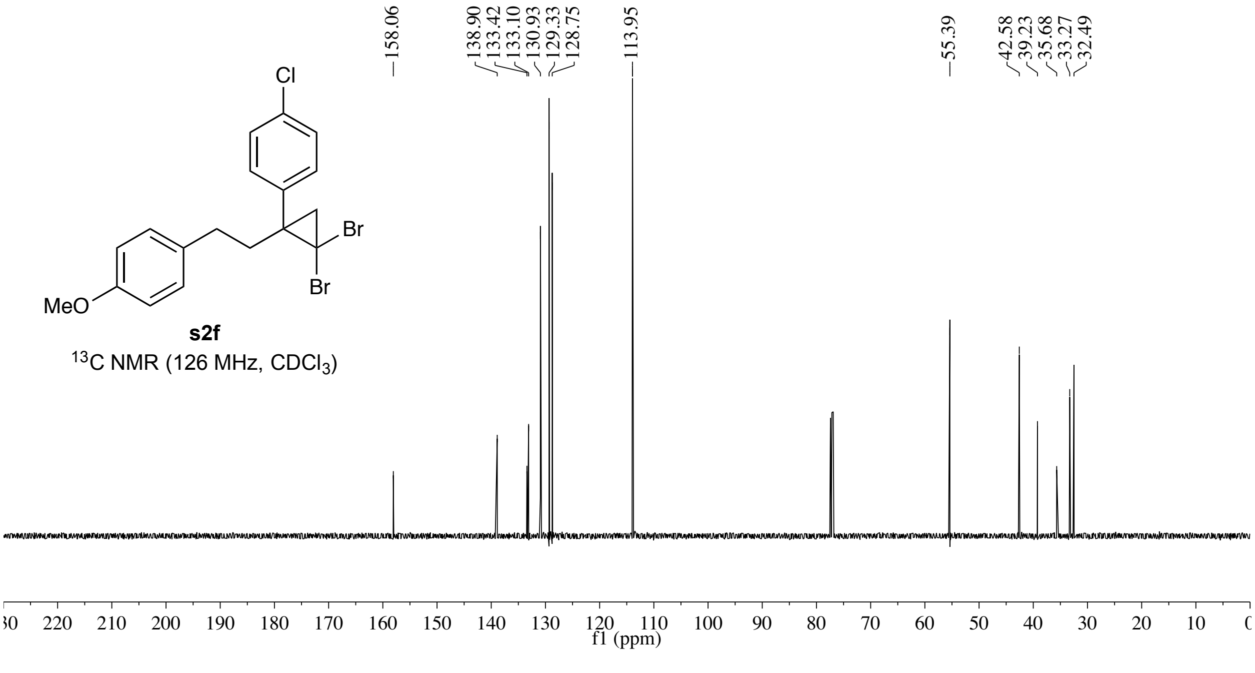

Supplementary Figure 71. ^{1}H NMR spectrum of s2g

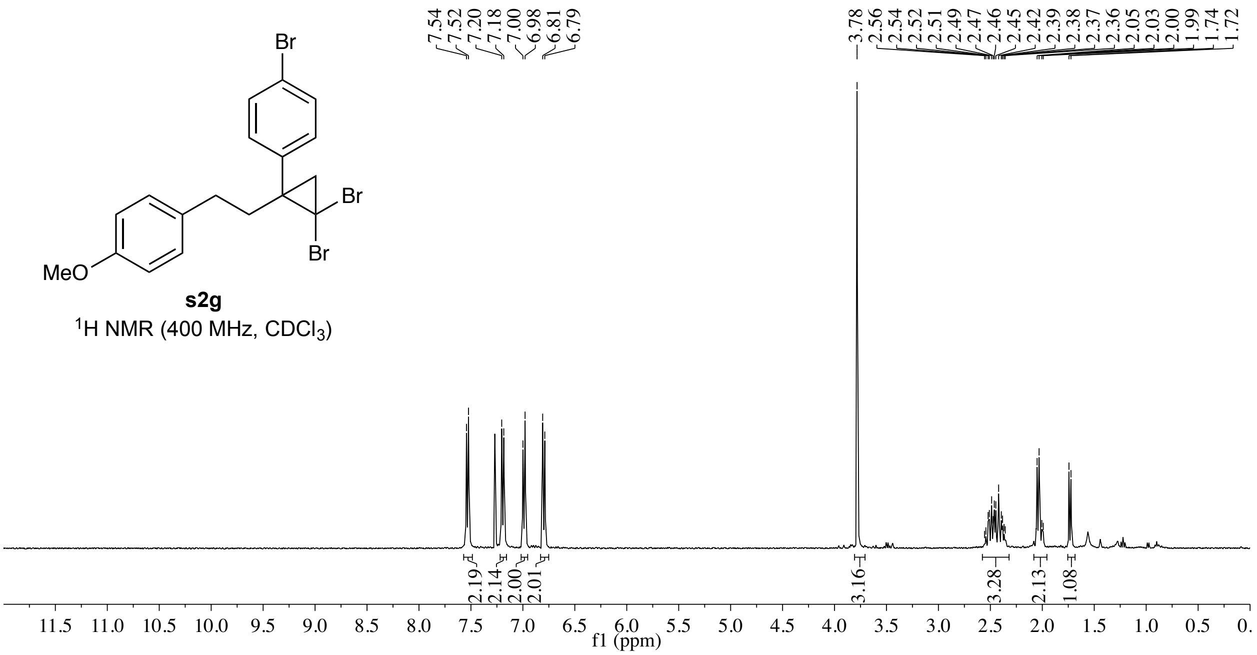

Supplementary Figure 72. ^{13}C NMR spectrum of s2g

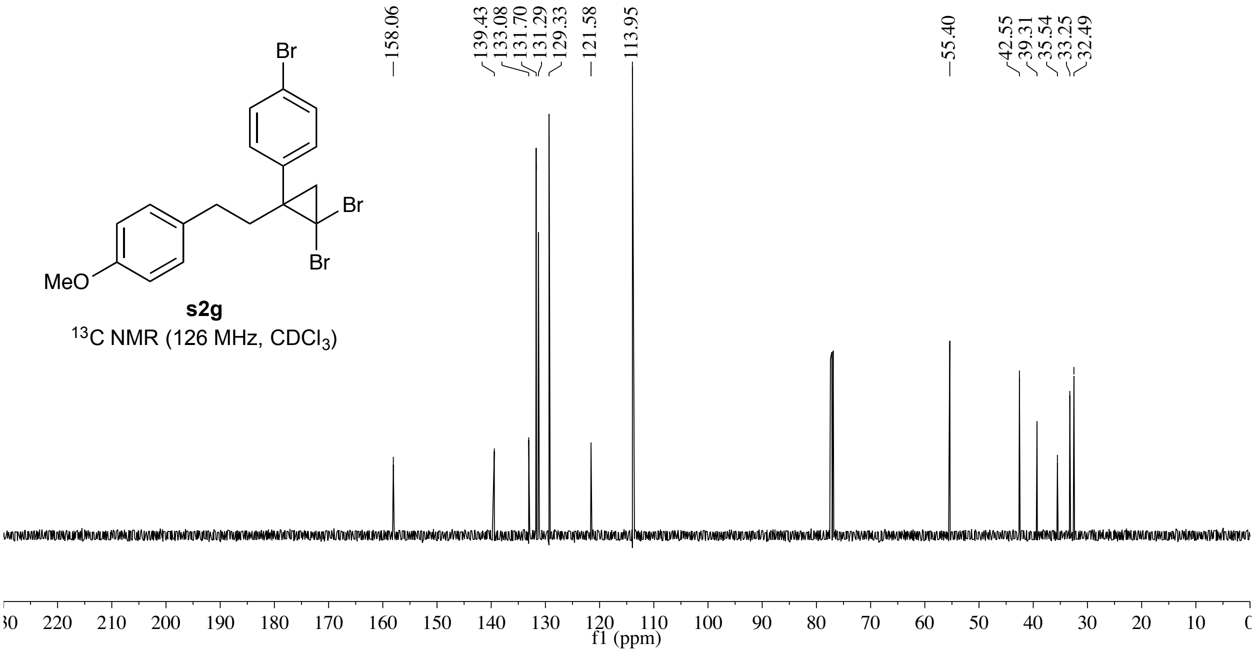

Supplementary Figure 73. ^{1}H NMR spectrum of s2h

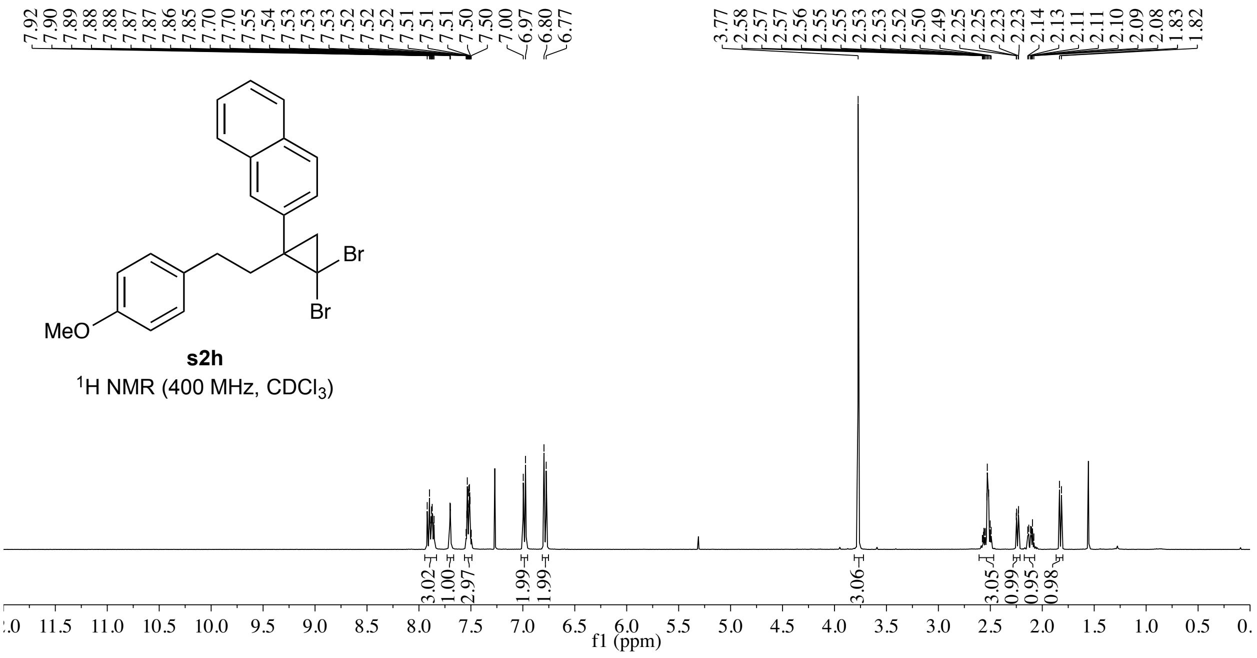

Supplementary Figure 74. ^{13}C NMR spectrum of s2h

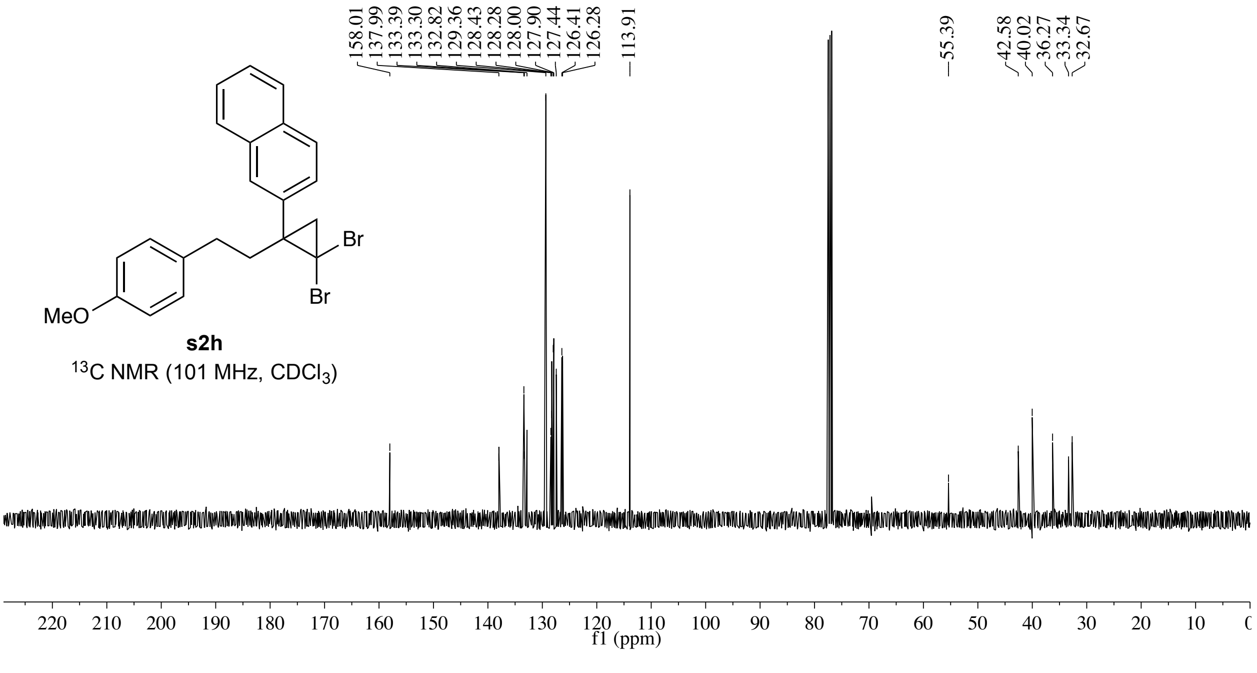

Supplementary Figure 75. ^{1}H NMR spectrum of s2i

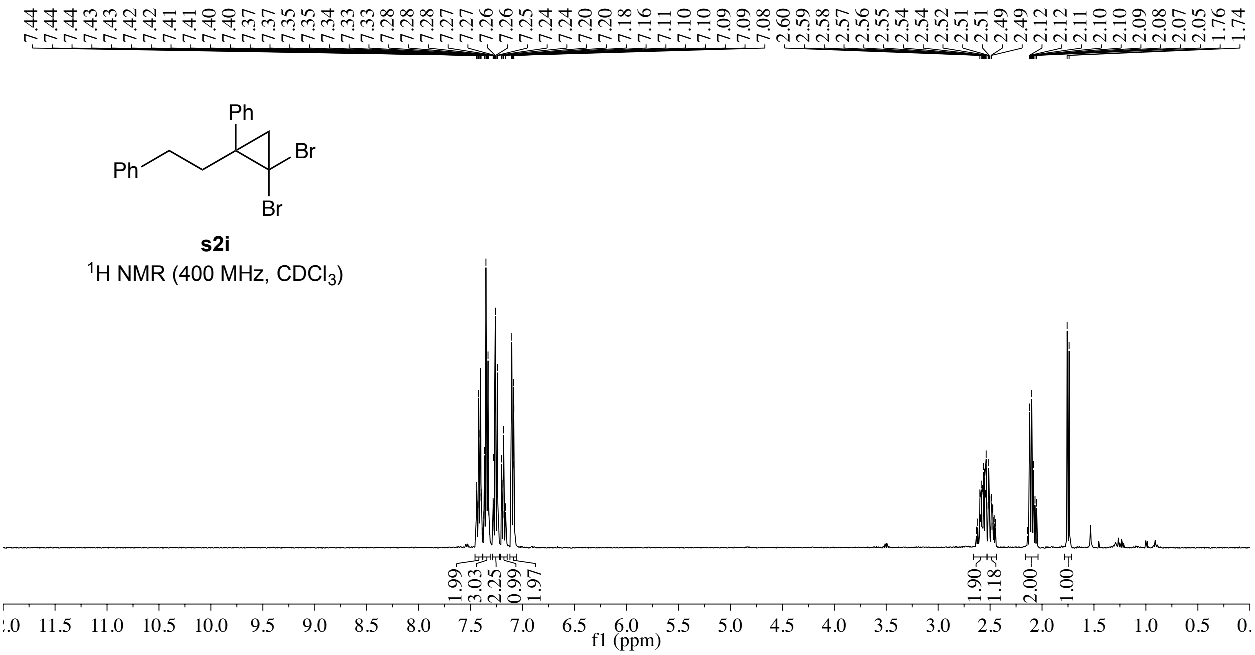

Supplementary Figure 76. ^{13}C NMR spectrum of s2i

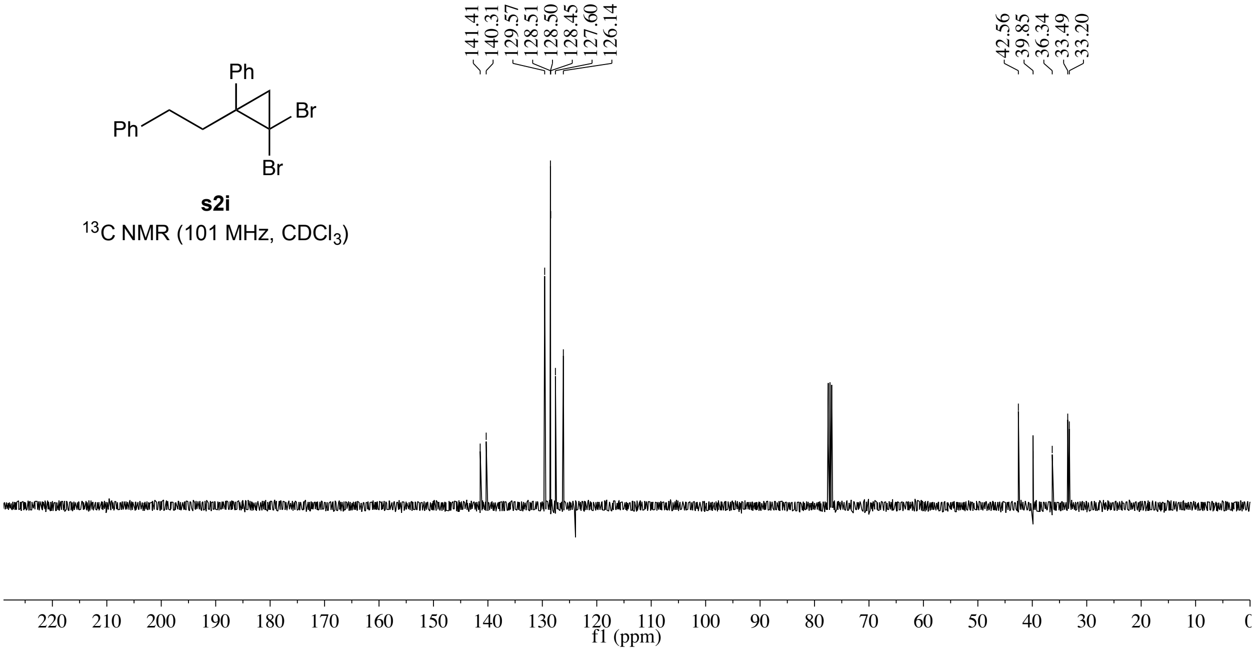

Supplementary Figure 77. ^{1}H NMR spectrum of 1a

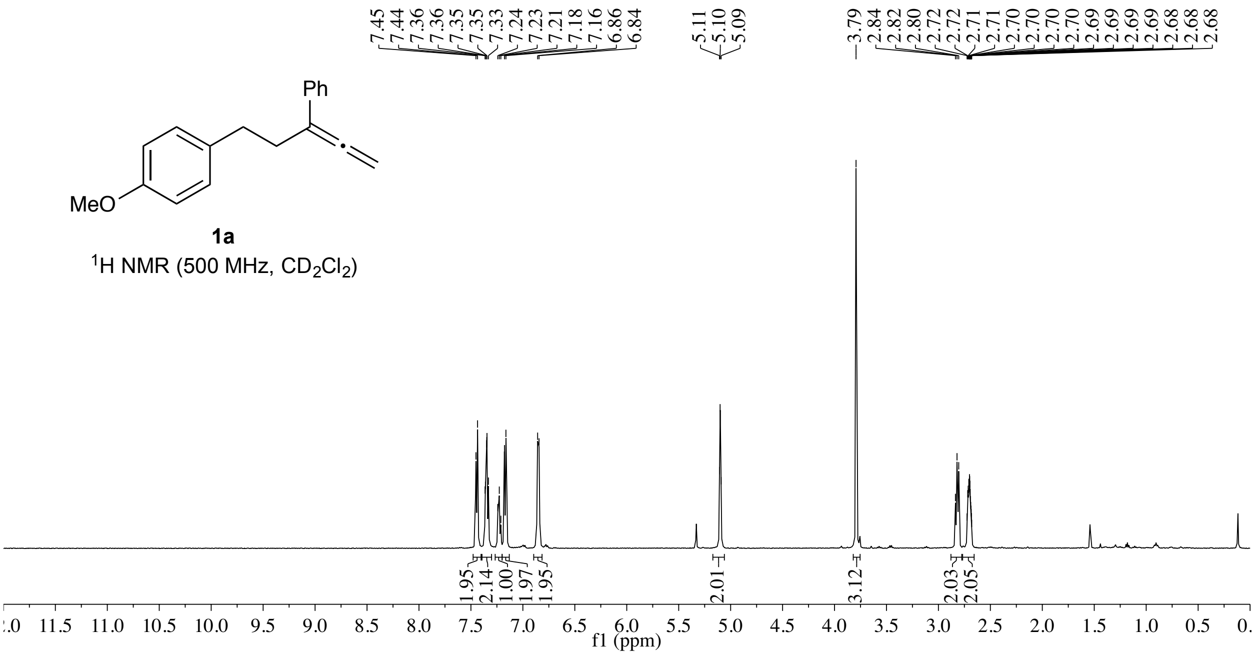

Supplementary Figure 78. ^{13}C NMR spectrum of 1a

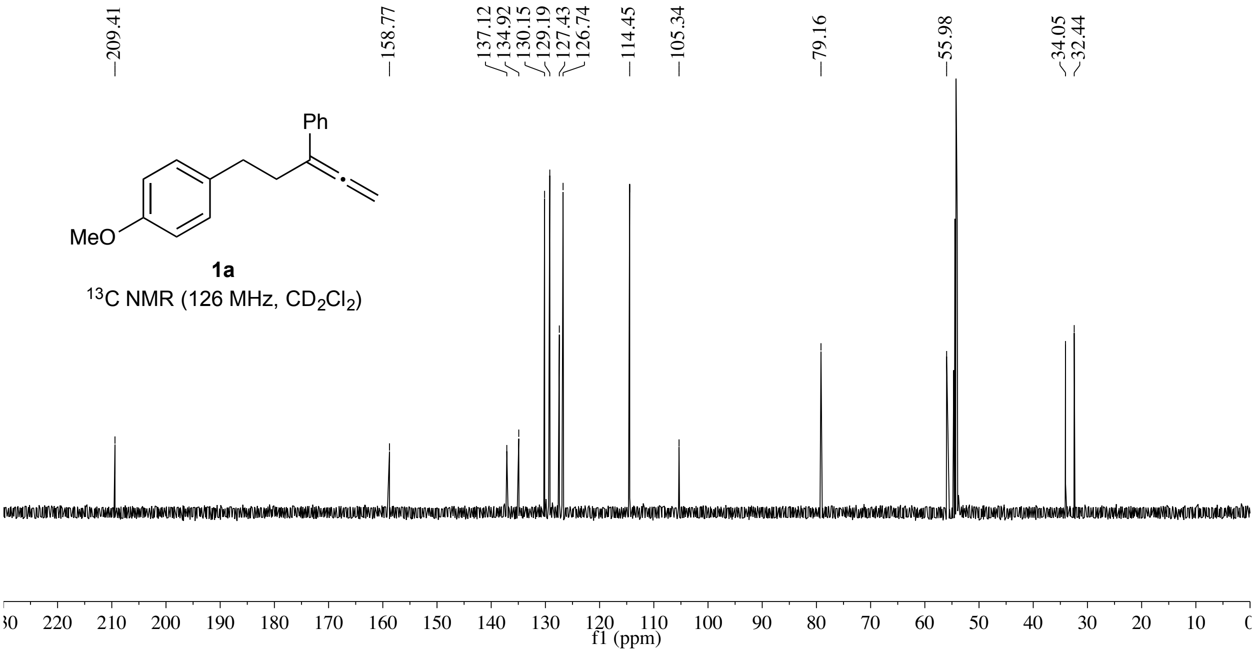

Supplementary Figure 79. ^{1}H NMR spectrum of 1c

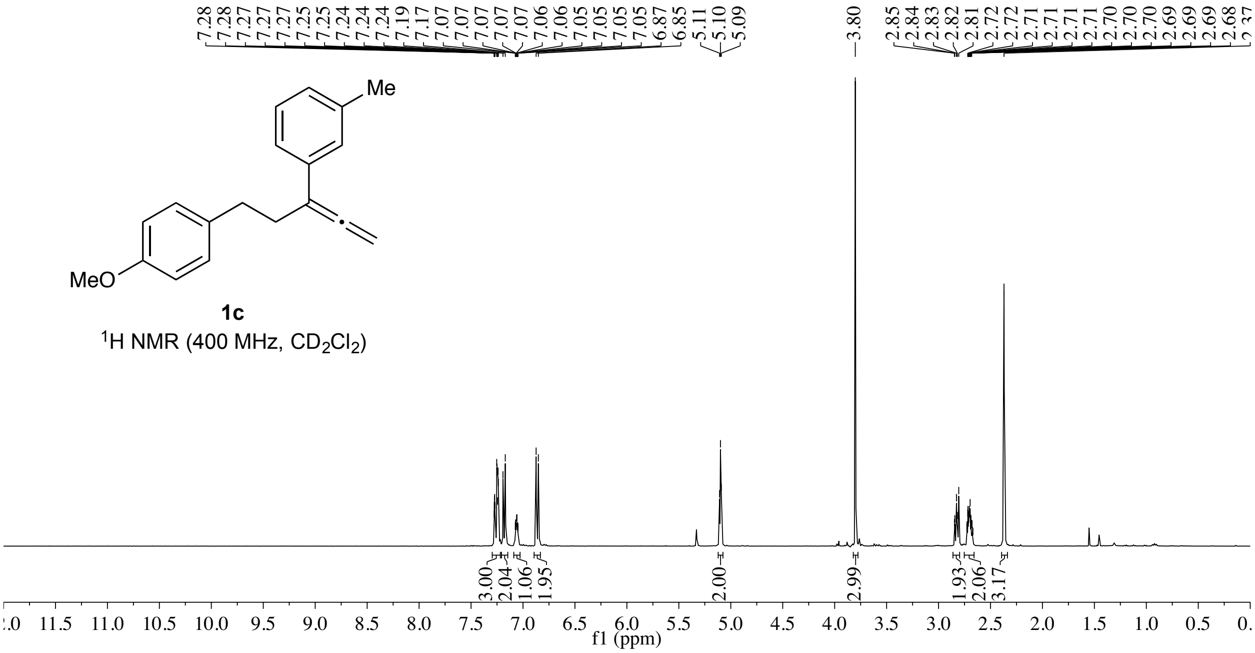

Supplementary Figure 80. ^{13}C NMR spectrum of 1c

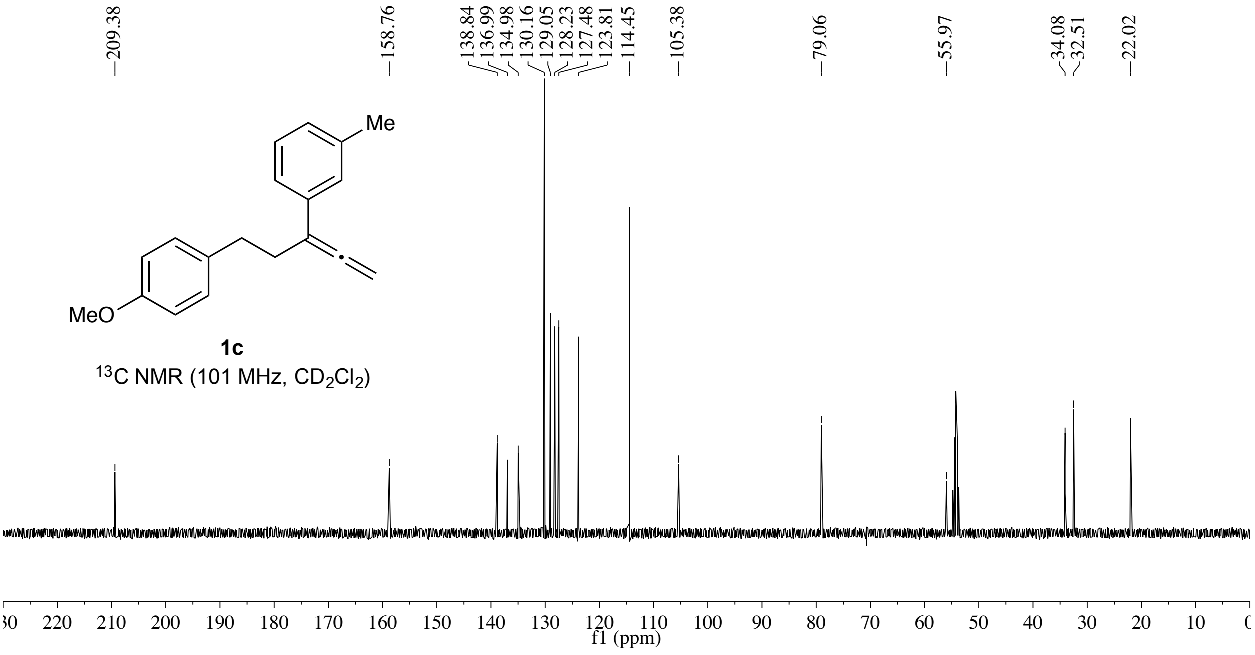

Supplementary Figure 81. ^{1}H NMR spectrum of 1d

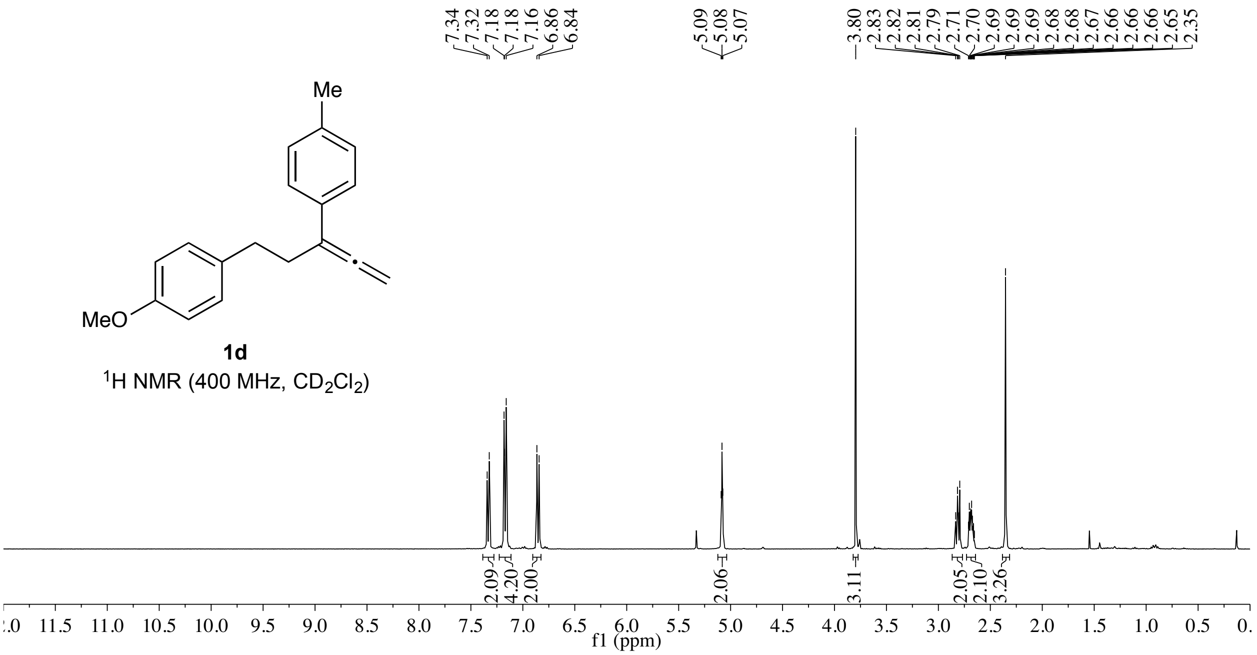

Supplementary Figure 82. ^{13}C NMR spectrum of 1d

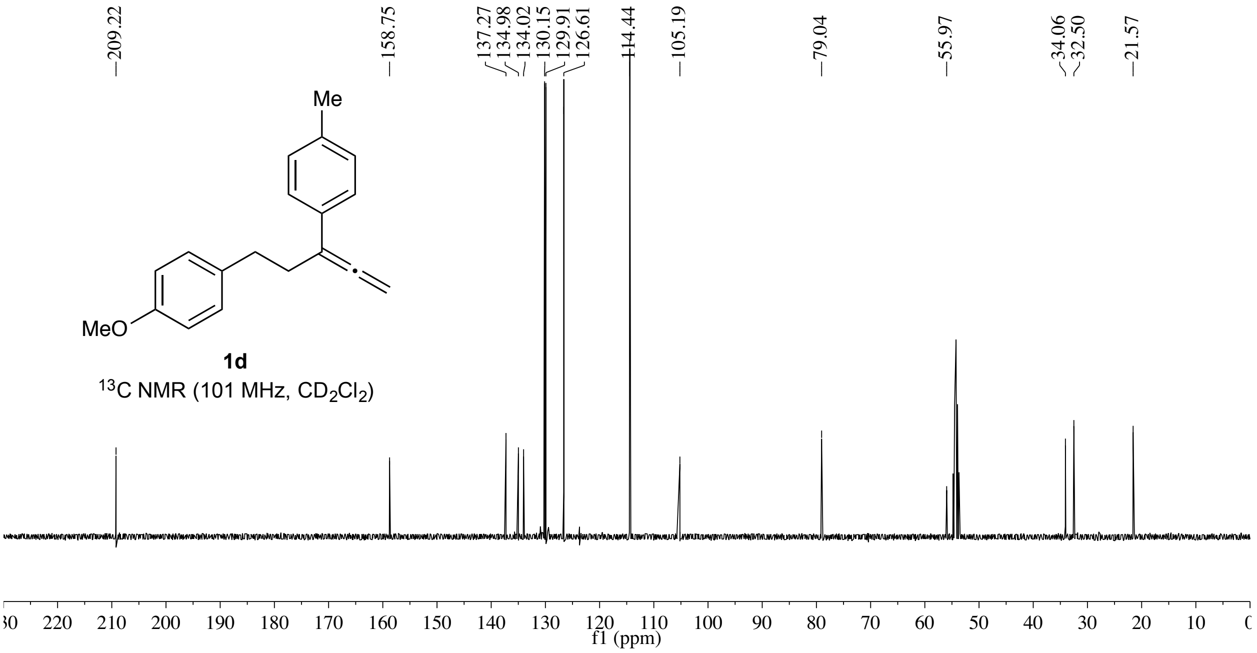

Supplementary Figure 83. ^{1}H NMR spectrum of 1e

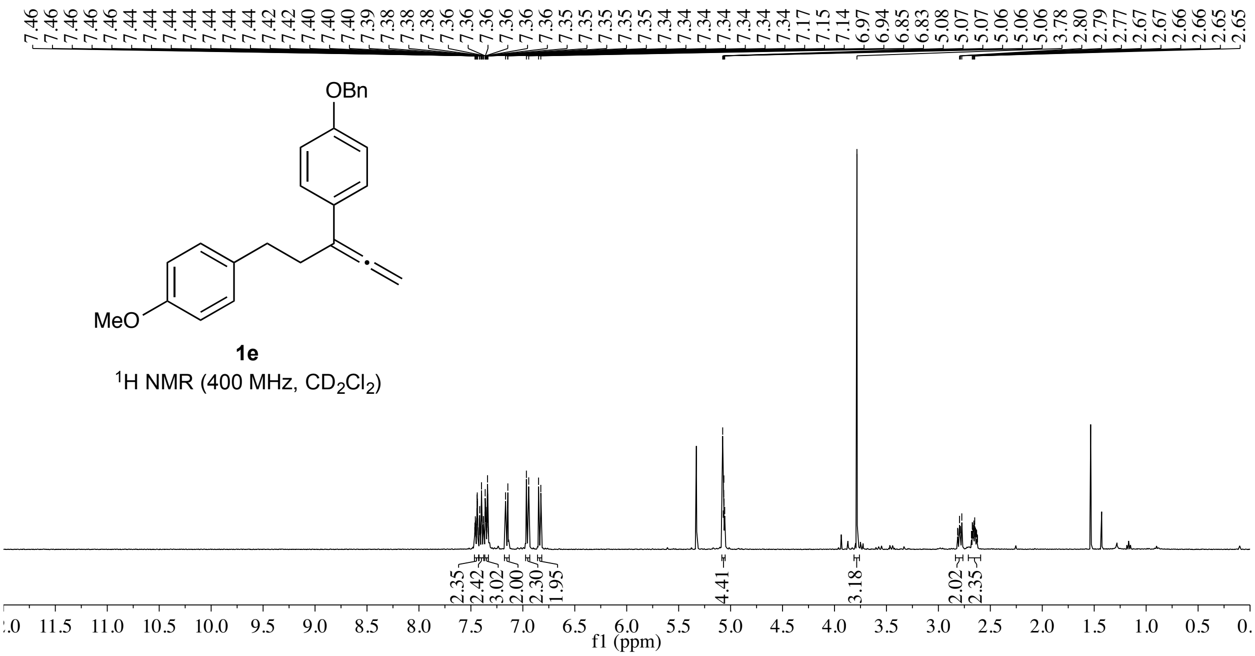

Supplementary Figure 84. ^{13}C NMR spectrum of 1e

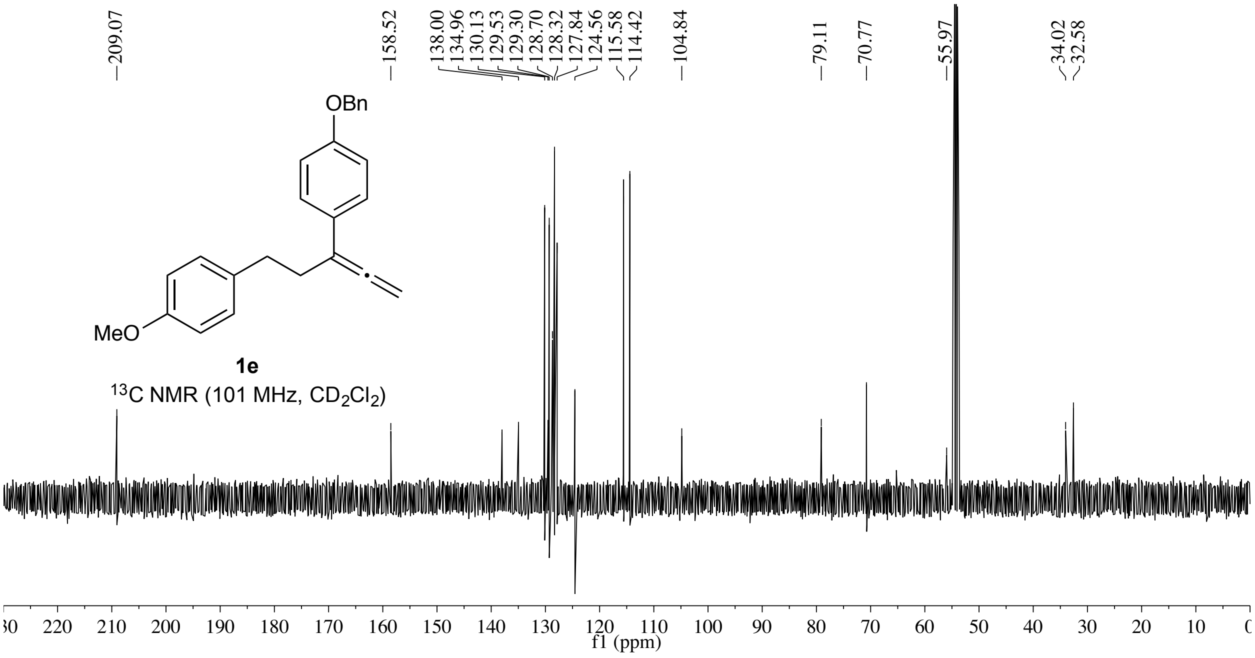

Supplementary Figure 85. ^1H NMR spectrum of 1f

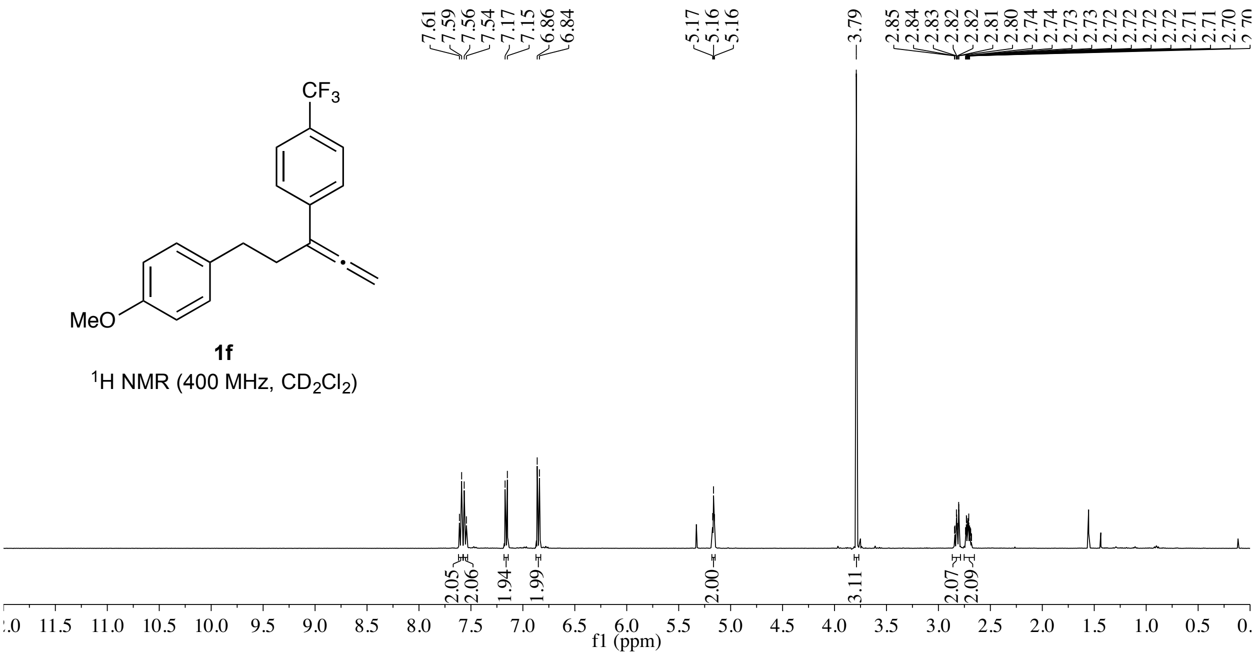

Supplementary Figure 86. ^{13}C NMR spectrum of 1f

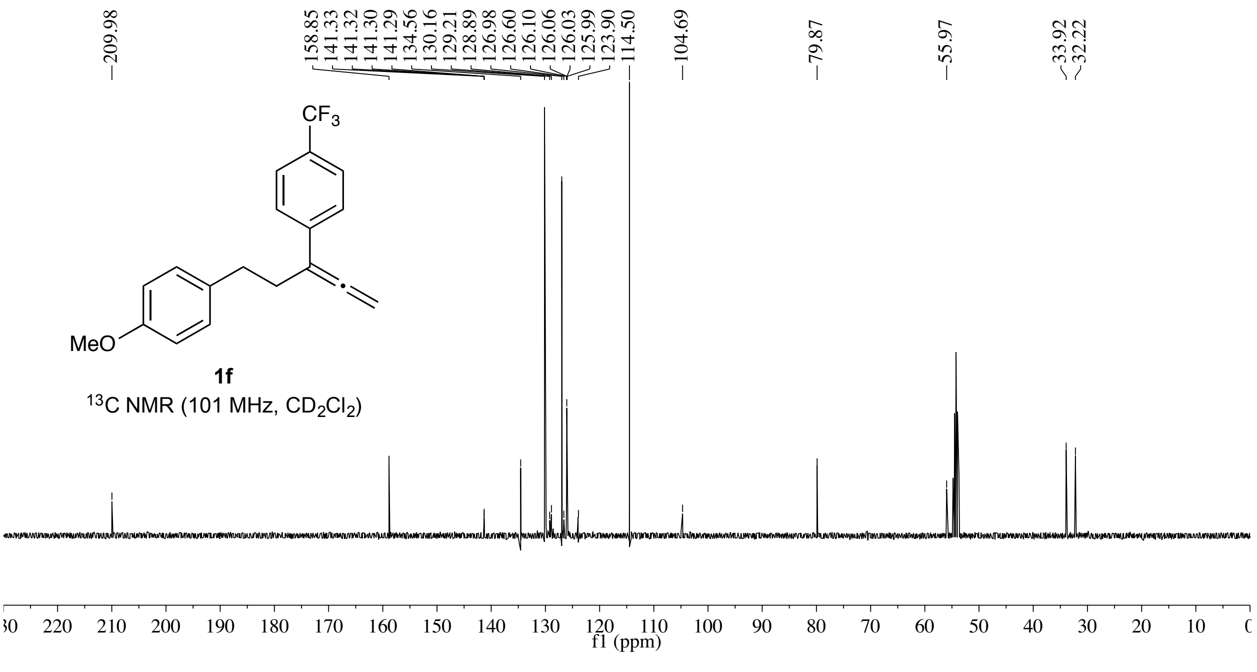

Supplementary Figure 87. ^{19}F NMR spectrum of 1f

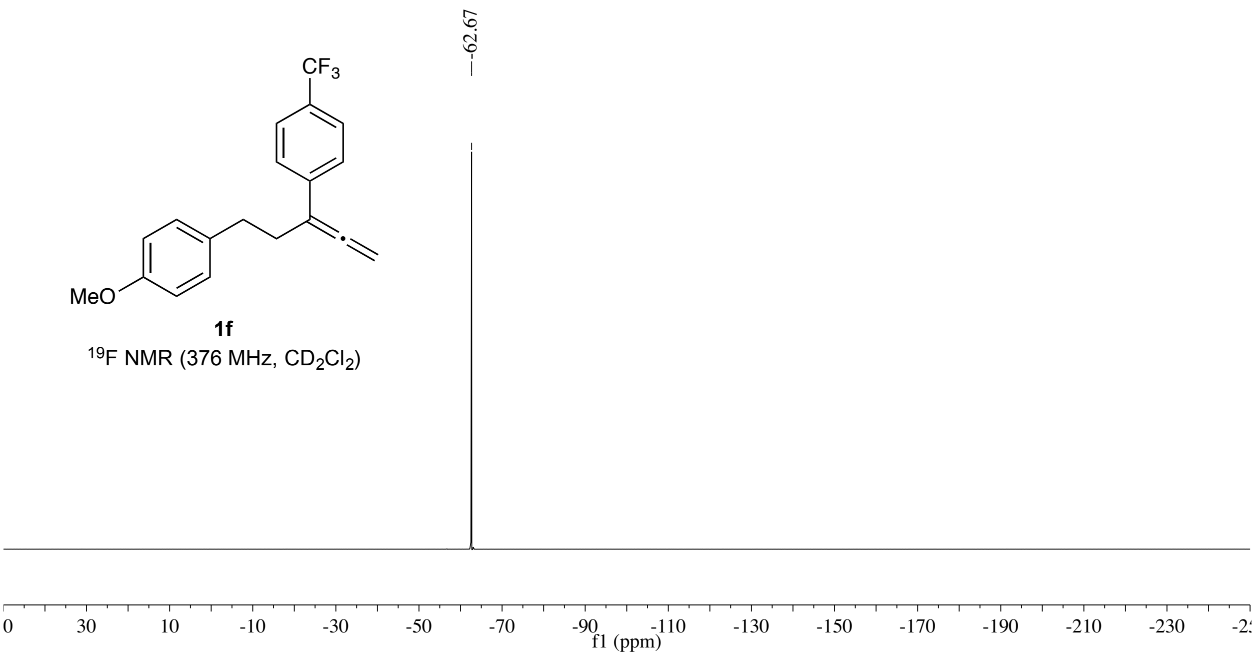

Supplementary Figure 88. ^{1}H NMR spectrum of 1g

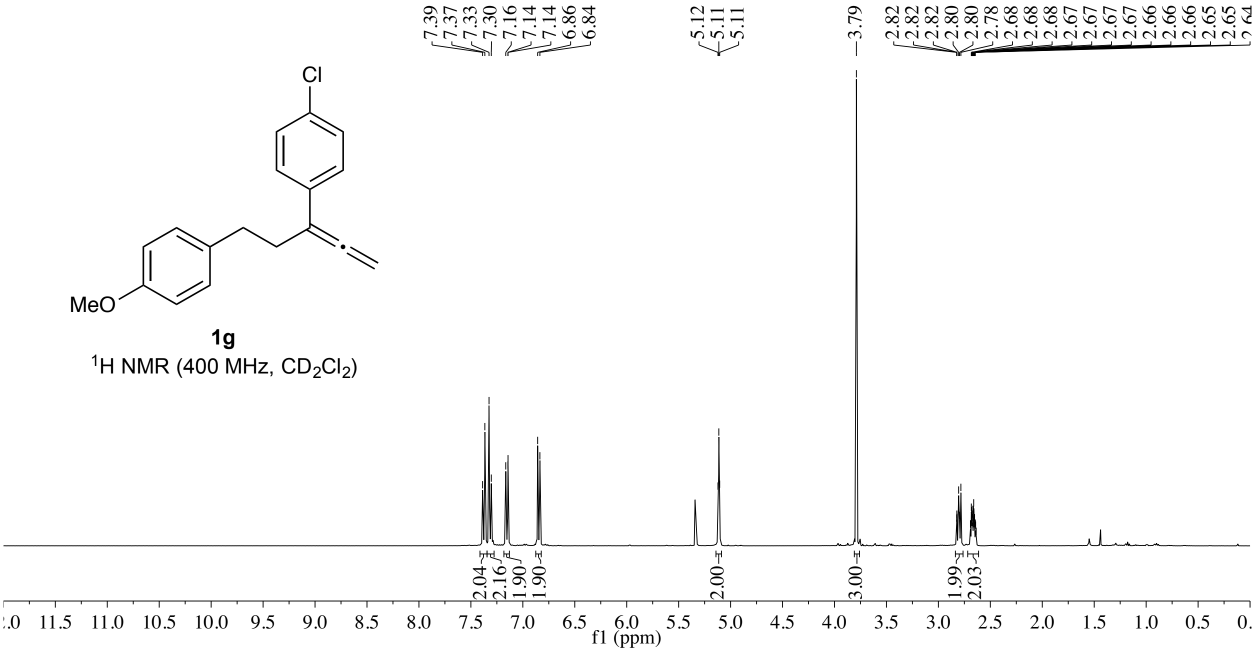

Supplementary Figure 89. ^{13}C NMR spectrum of 1g

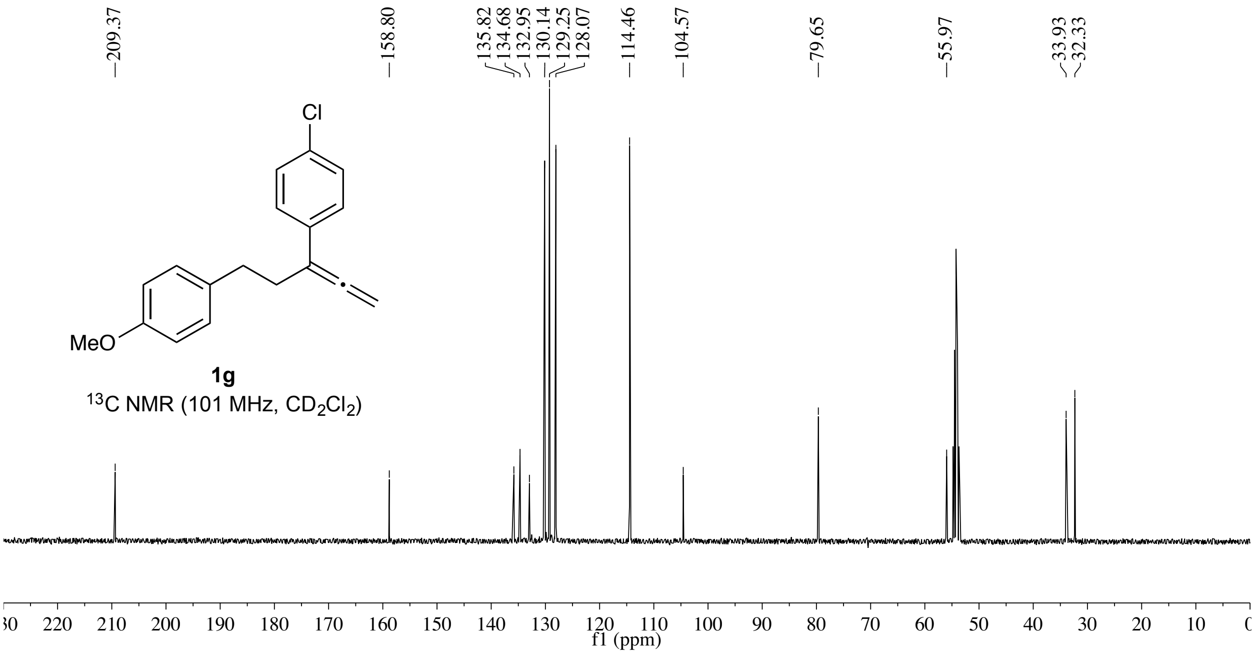

Supplementary Figure 90. ^{1}H NMR spectrum of 1h

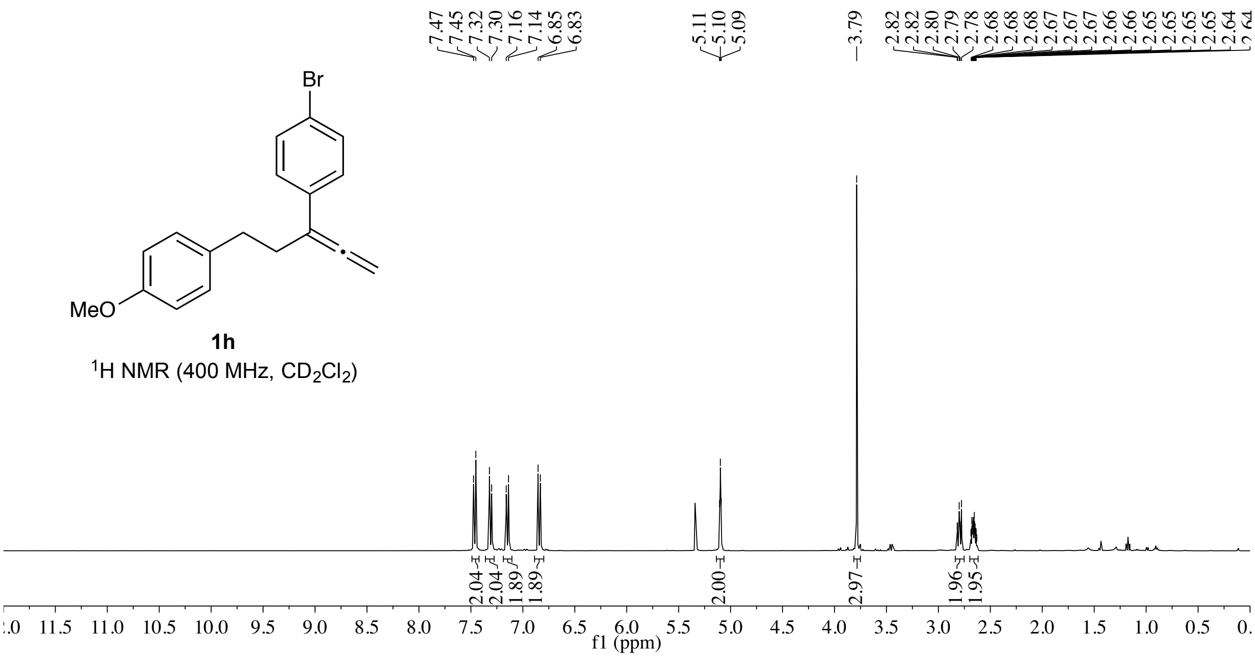

Supplementary Figure 91. ^{13}C NMR spectrum of 1h

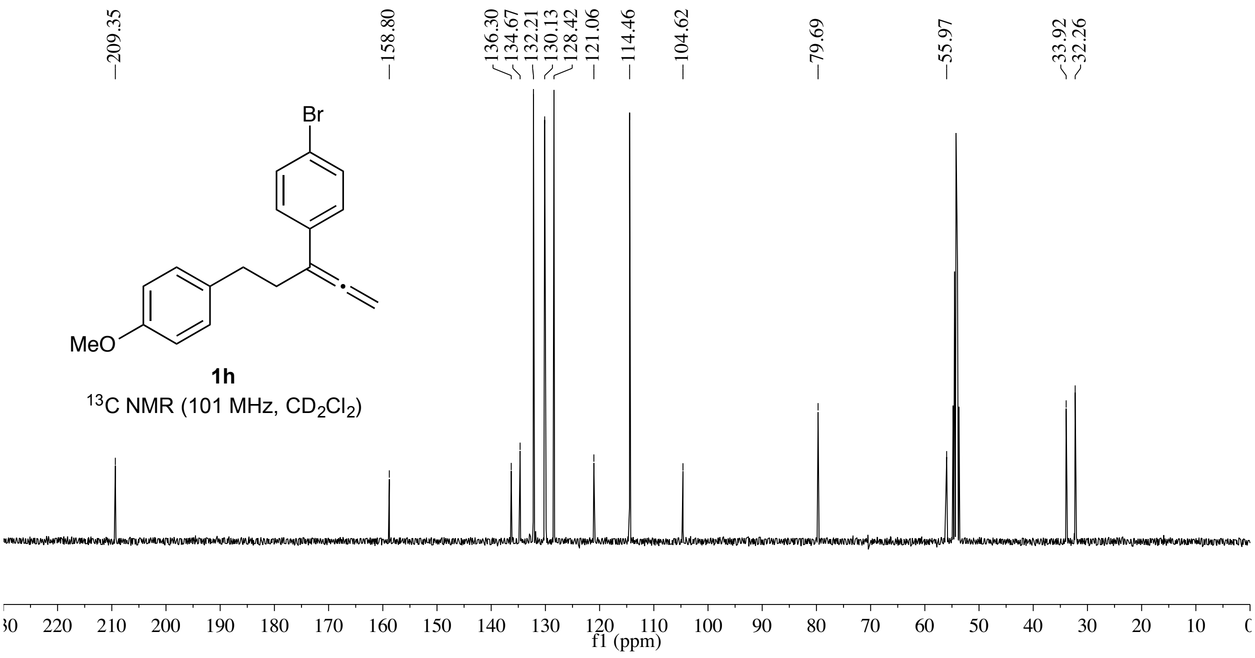

Supplementary Figure 92. ^{1}H NMR spectrum of 1i

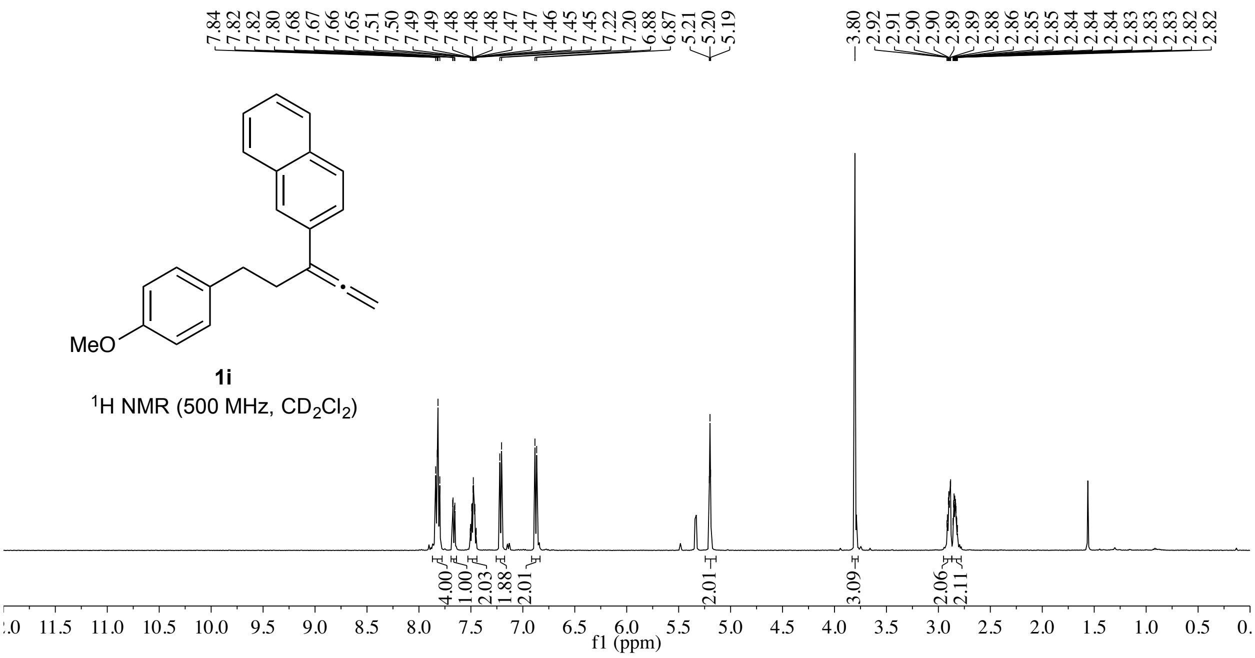

Supplementary Figure 93. ^{13}C NMR spectrum of 1i

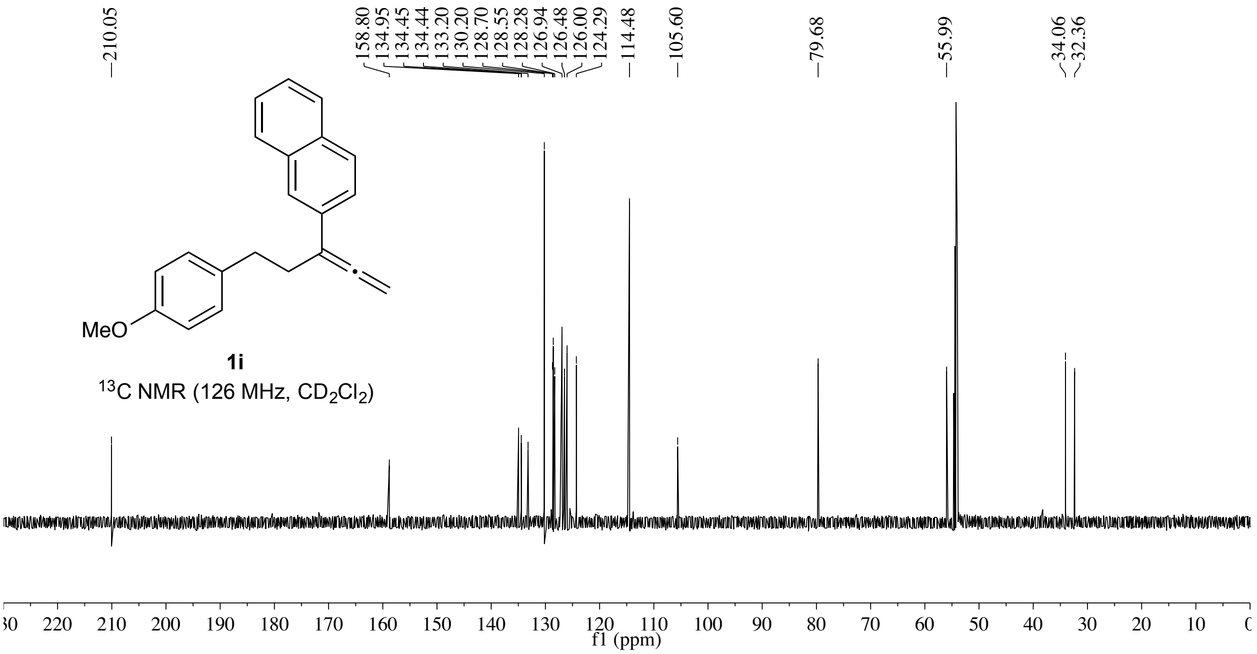

Supplementary Figure 94. ^{1}H NMR spectrum of 1n

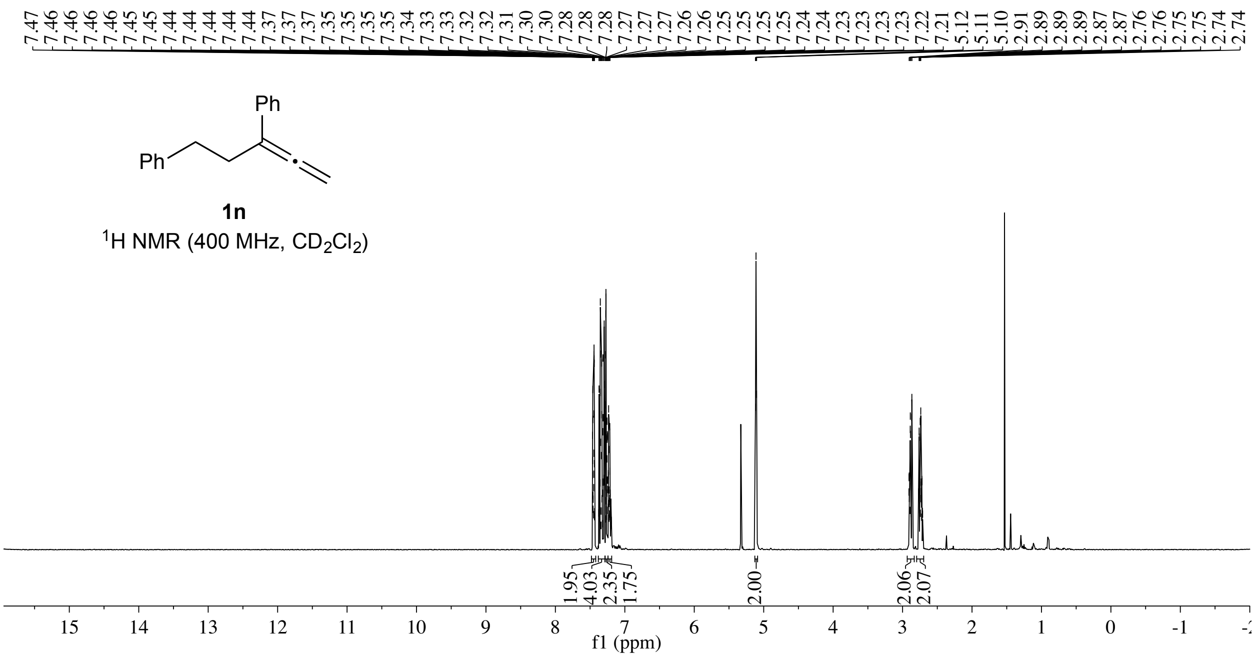

Supplementary Figure 95. ^{13}C NMR spectrum of 1n

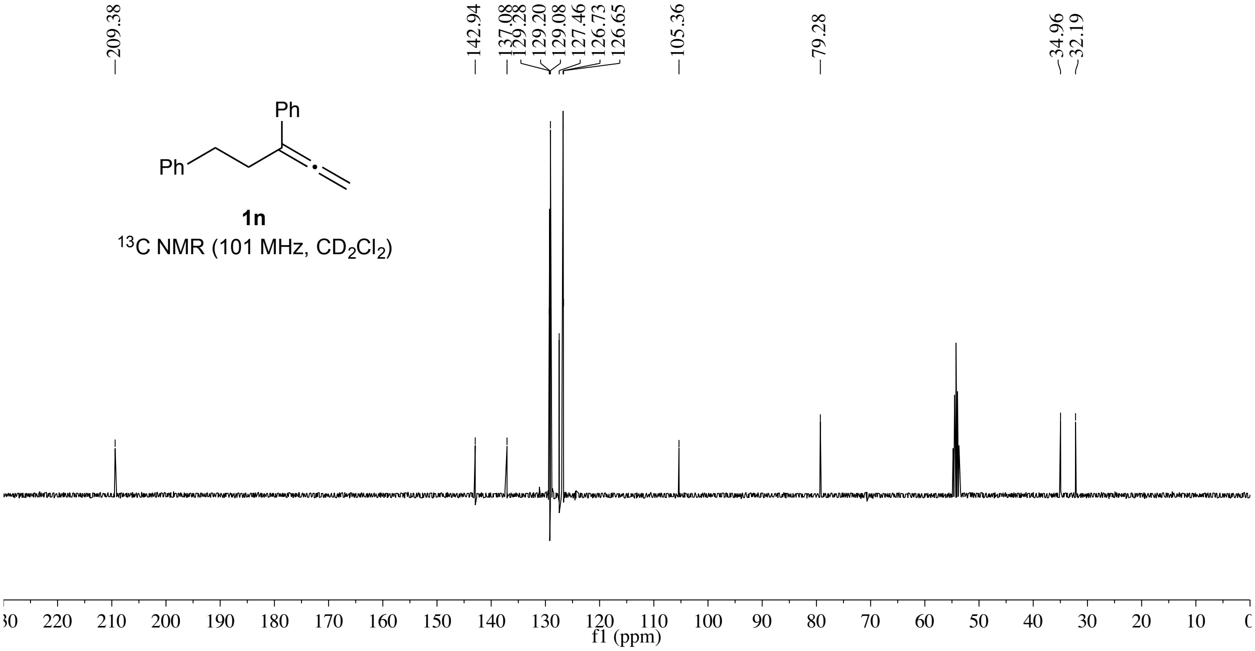

Supplementary Figure 96. ^{1}H NMR spectrum of s3a

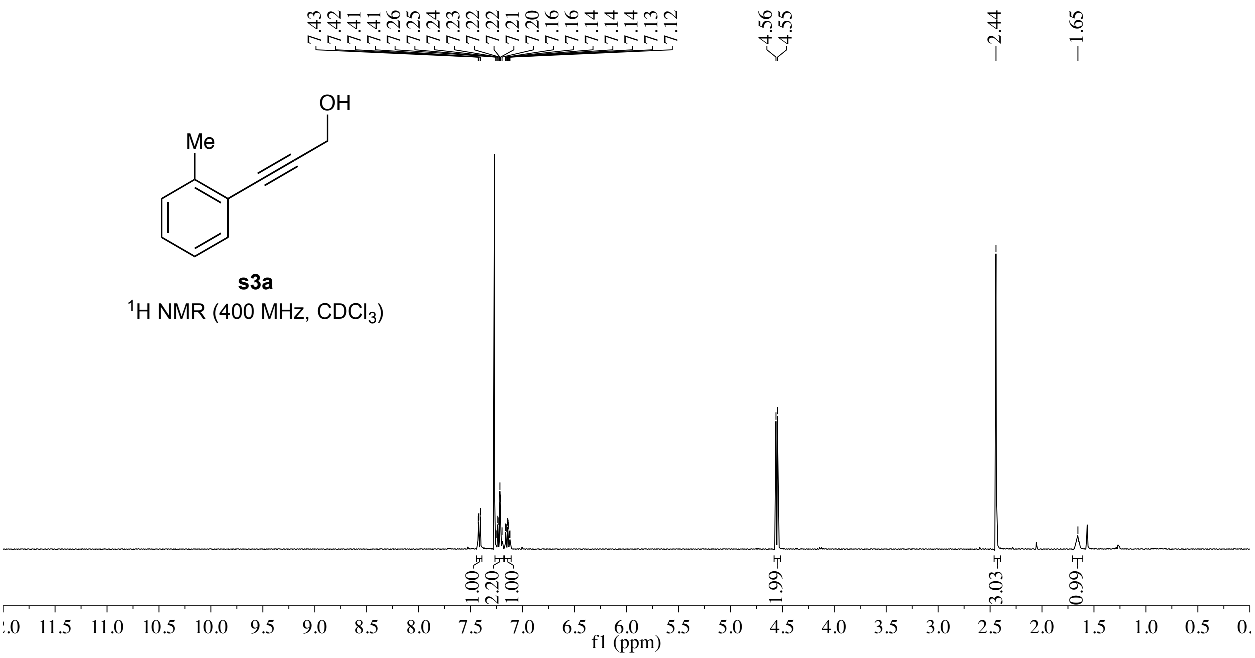

Supplementary Figure 97. ^{1}H NMR spectrum of s3b

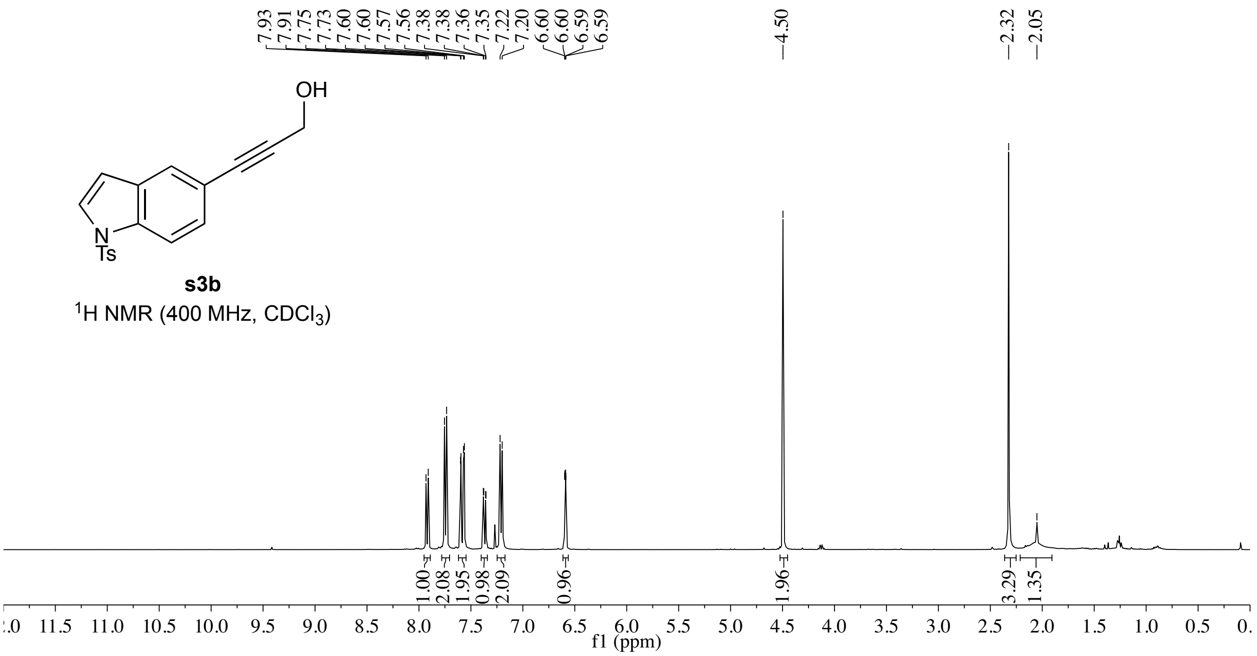

Supplementary Figure 98. ^{13}C NMR spectrum of s3b

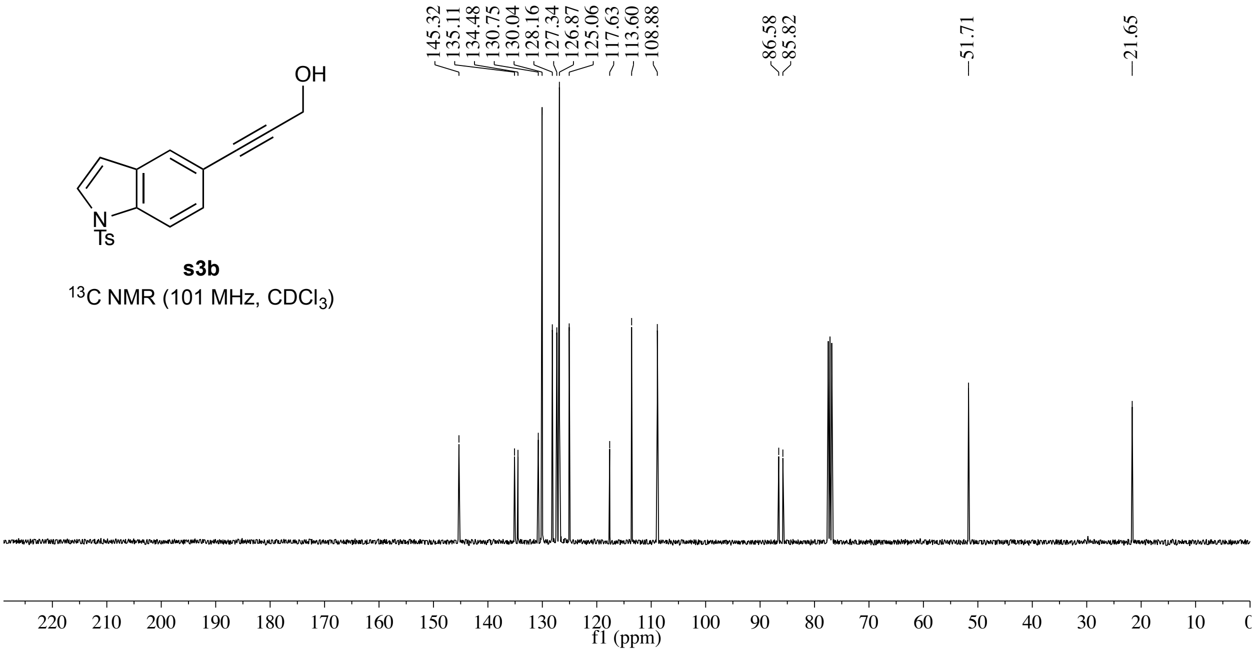

Supplementary Figure 99. ^{1}H NMR spectrum of s3c

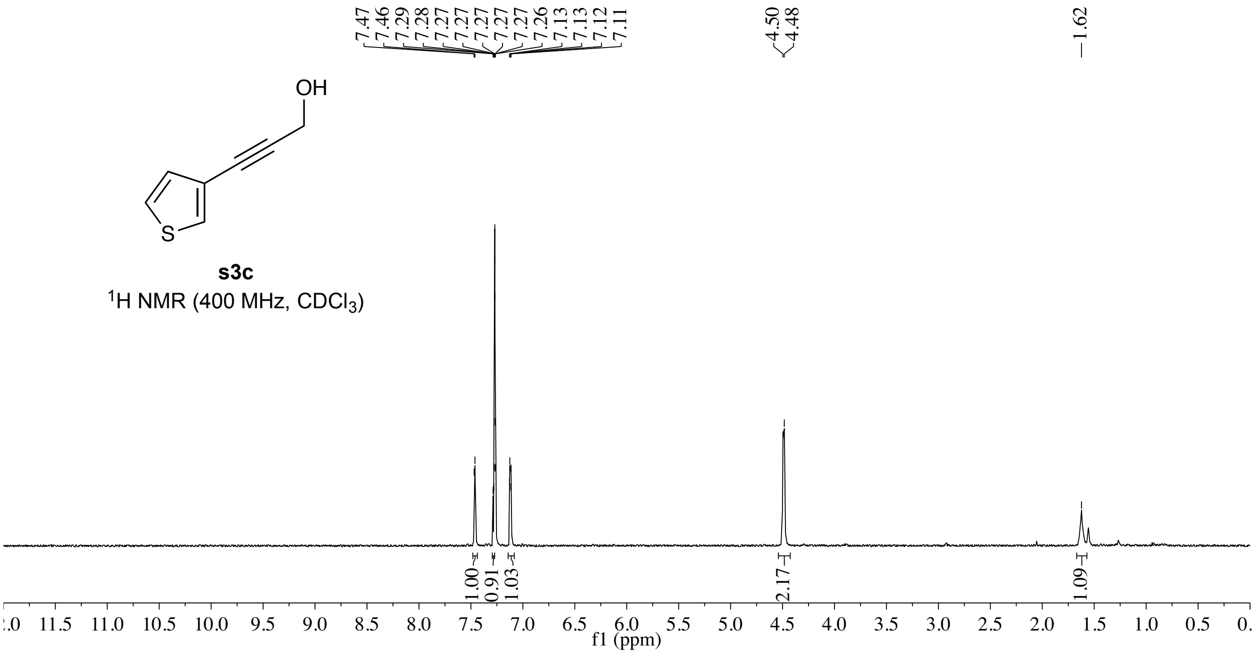

Supplementary Figure 100. ^{1}H NMR spectrum of s3d

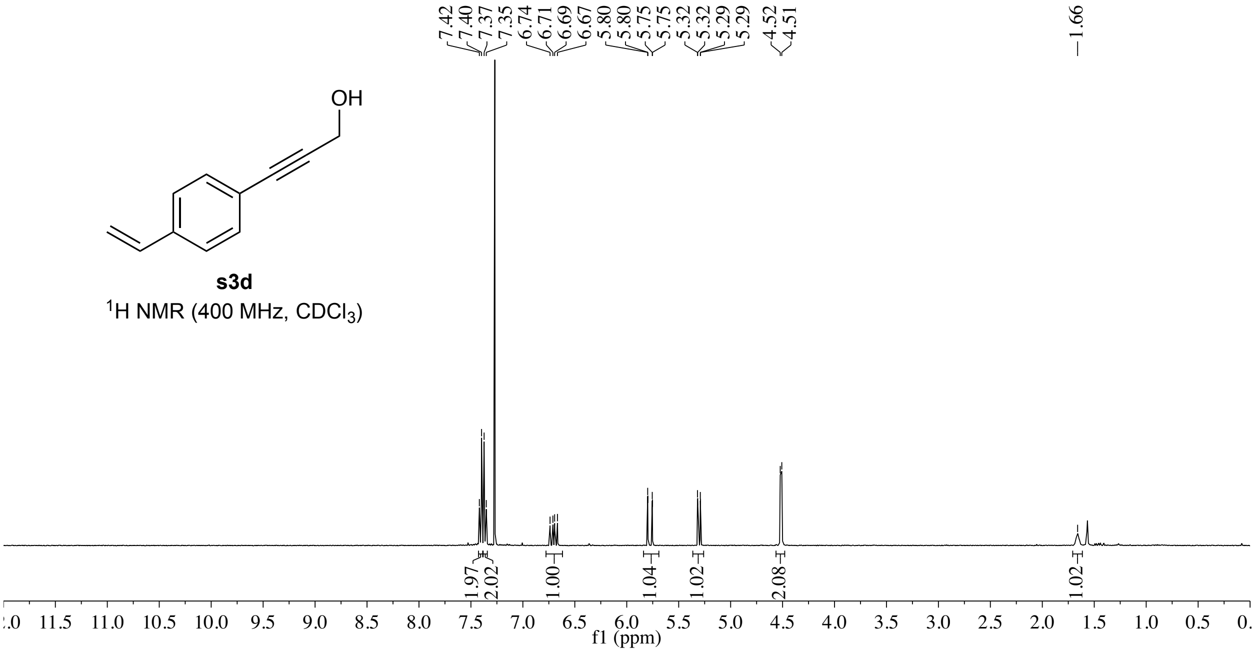

Supplementary Figure 101. ^{13}C NMR spectrum of s3d

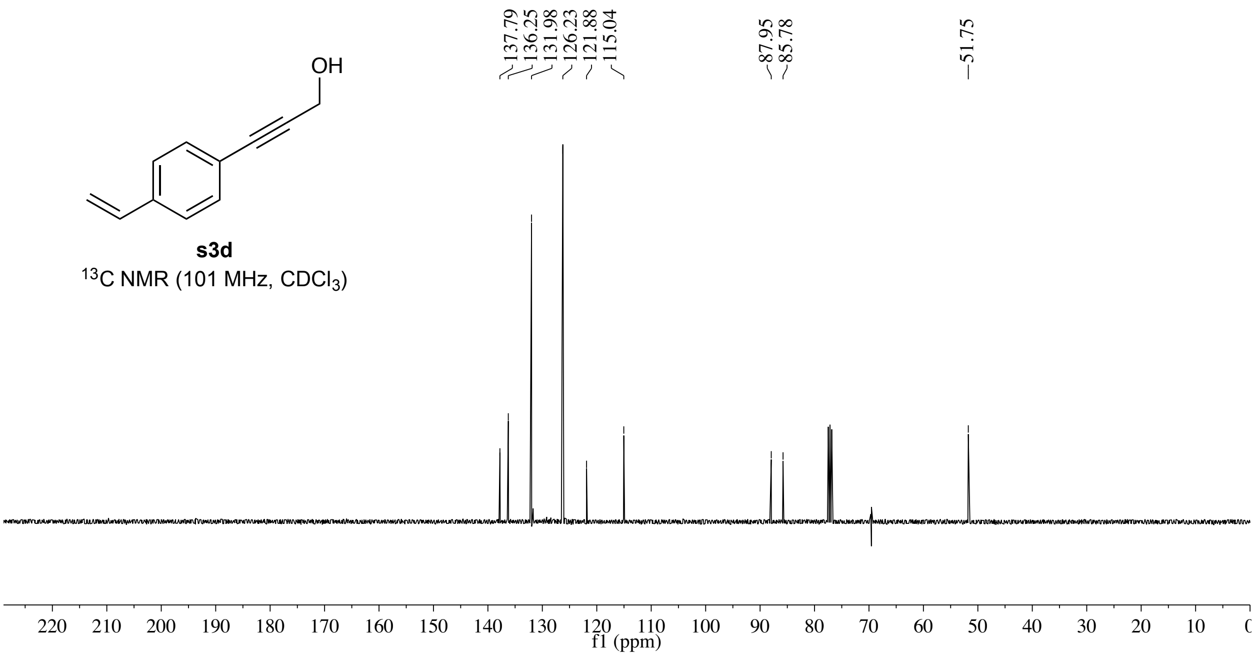

Supplementary Figure 102. ^{1}H NMR spectrum of s3e

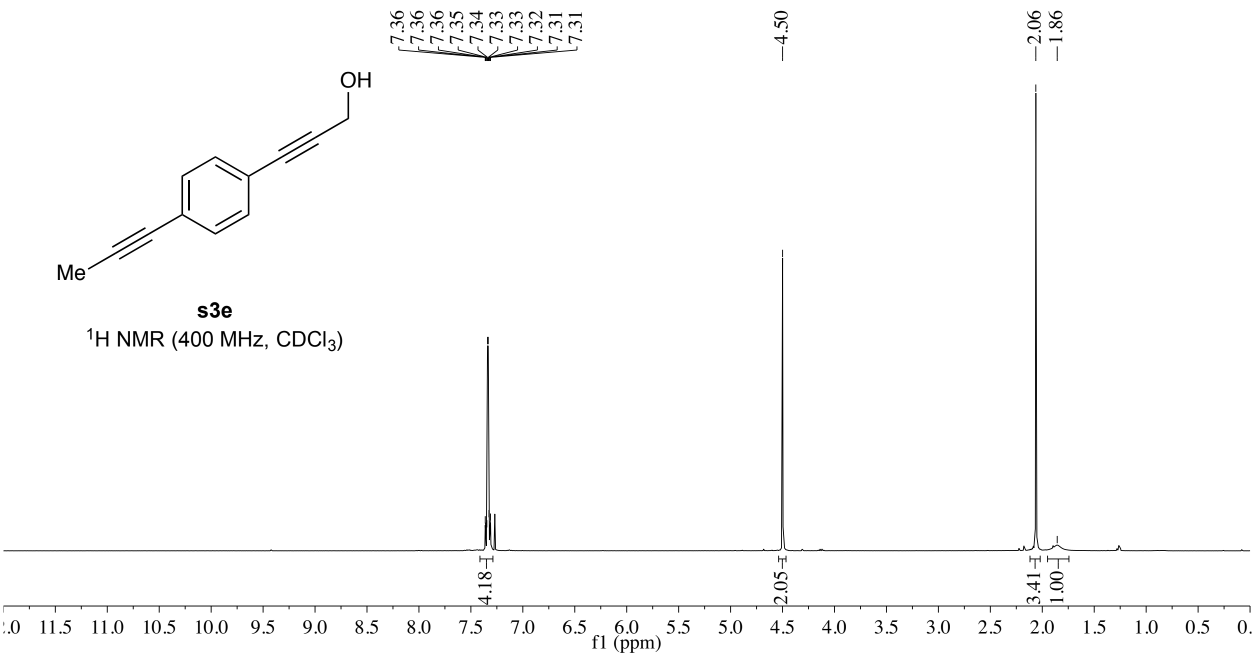

Supplementary Figure 103. ^{13}C NMR spectrum of s3e

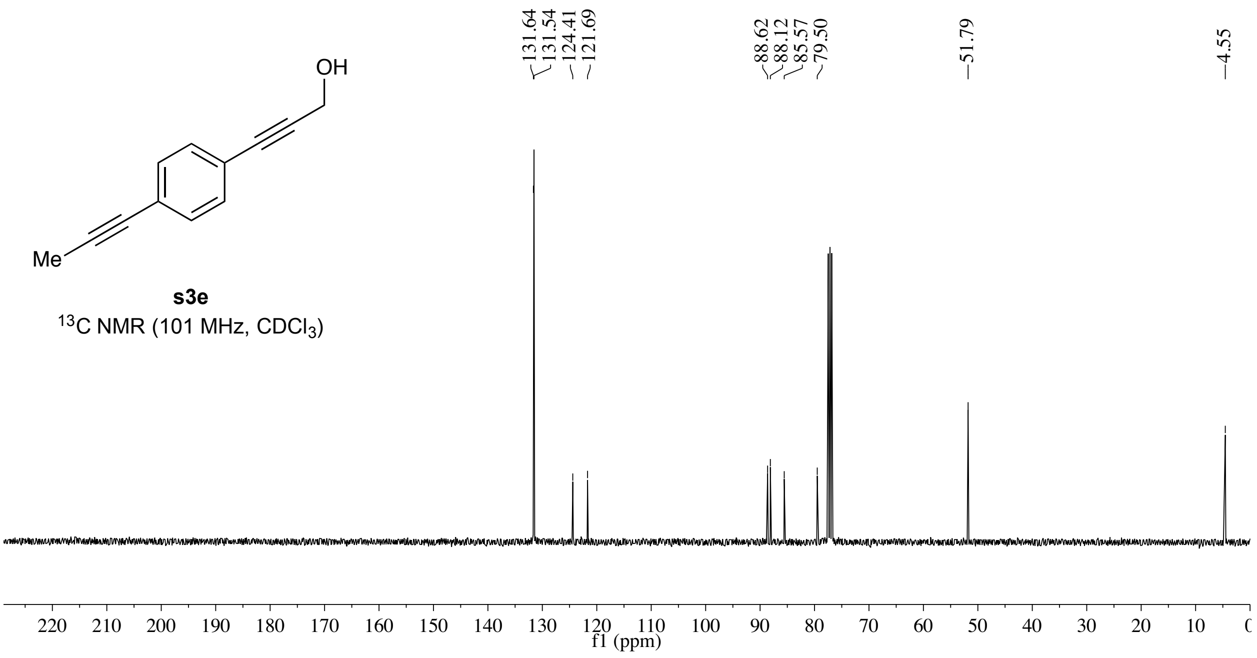

Supplementary Figure 104. ^{1}H NMR spectrum of s3f

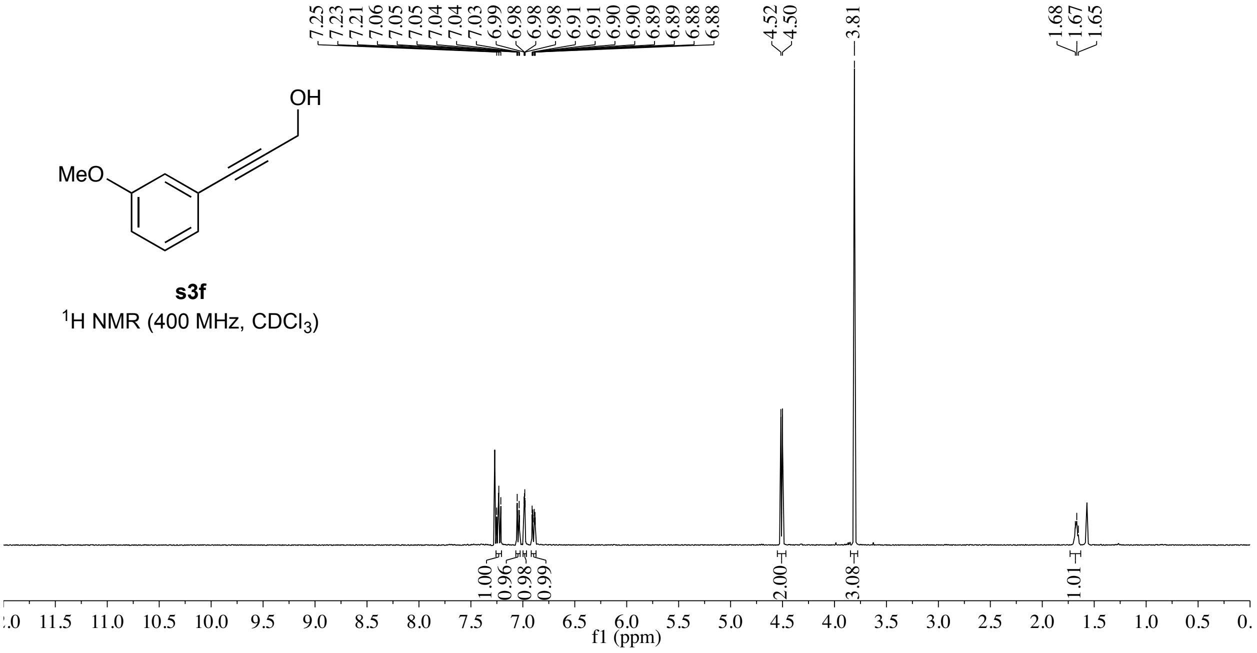

Supplementary Figure 105. ^{1}H NMR spectrum of 1b

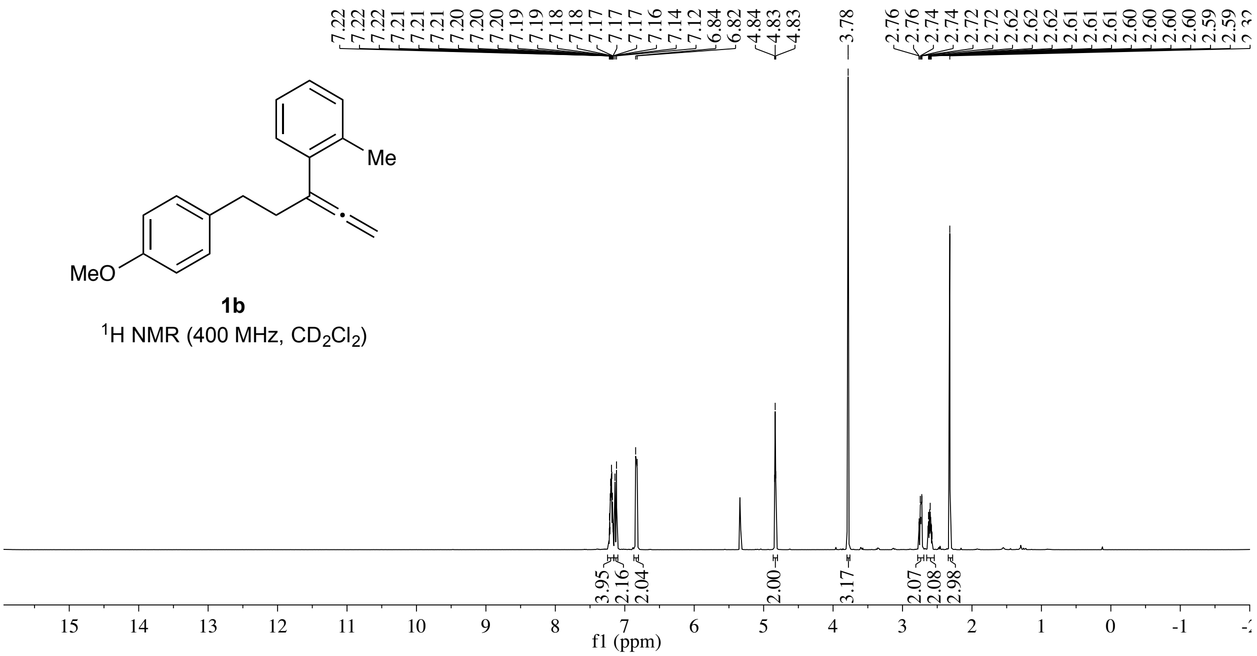

Supplementary Figure 106. ^{13}C NMR spectrum of 1b

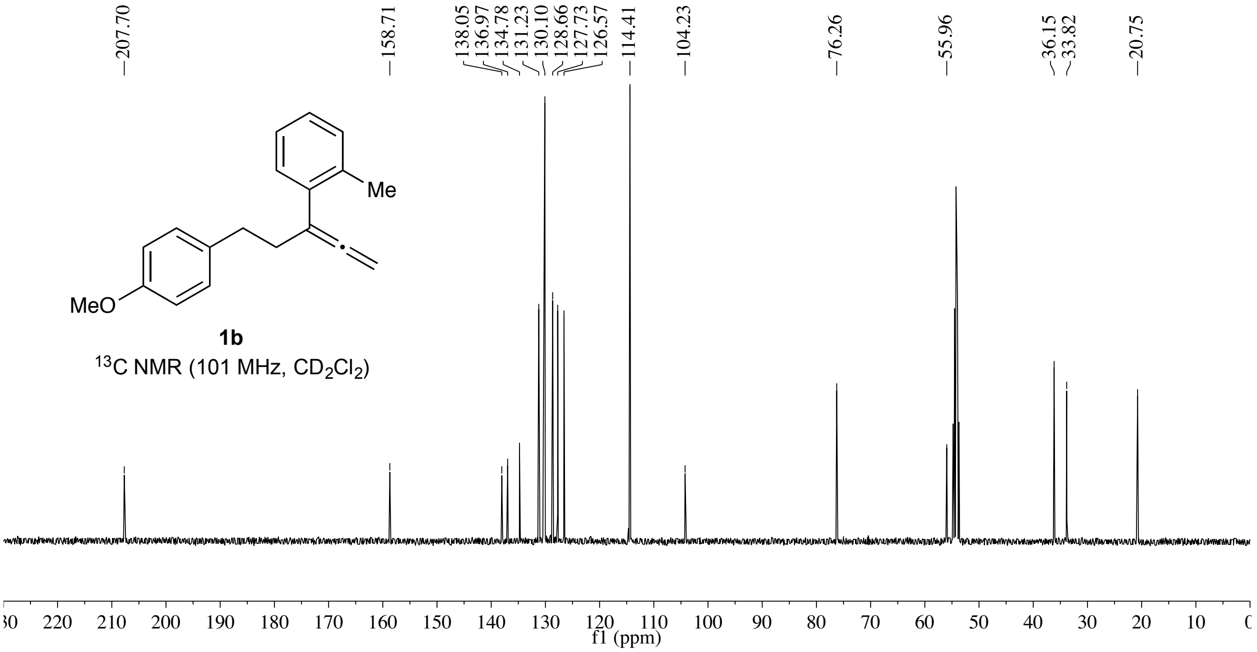

Supplementary Figure 107. ^{1}H NMR spectrum of 1j

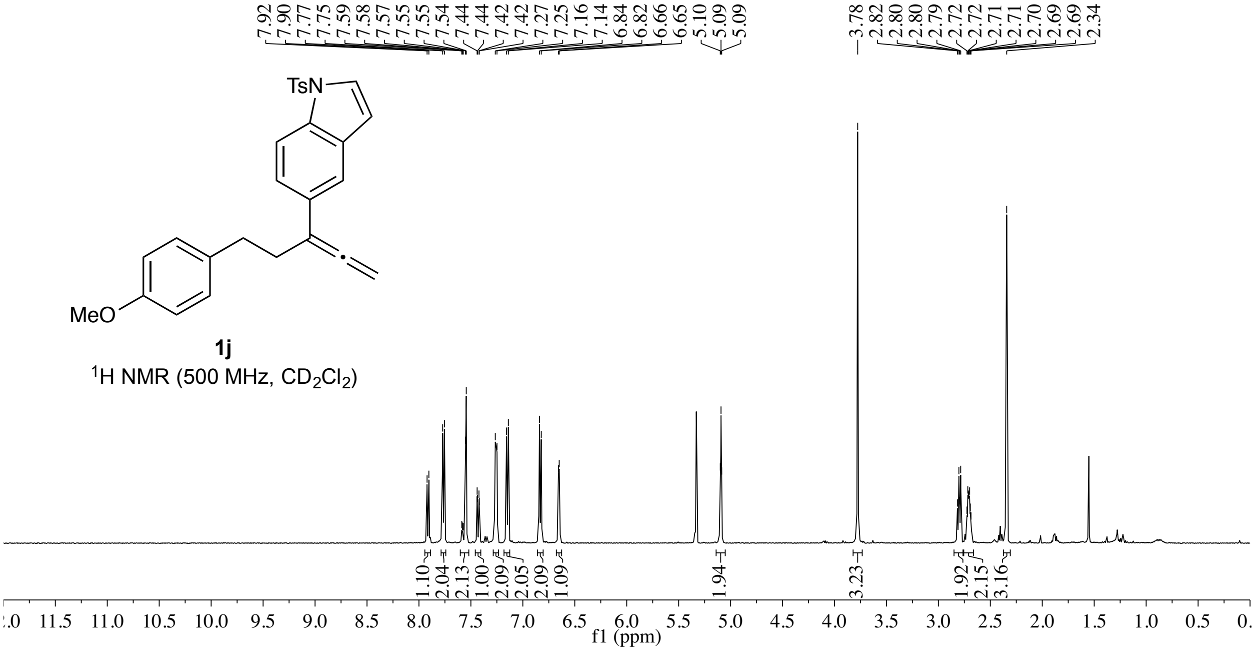

Supplementary Figure 108. ^{13}C NMR spectrum of 1j

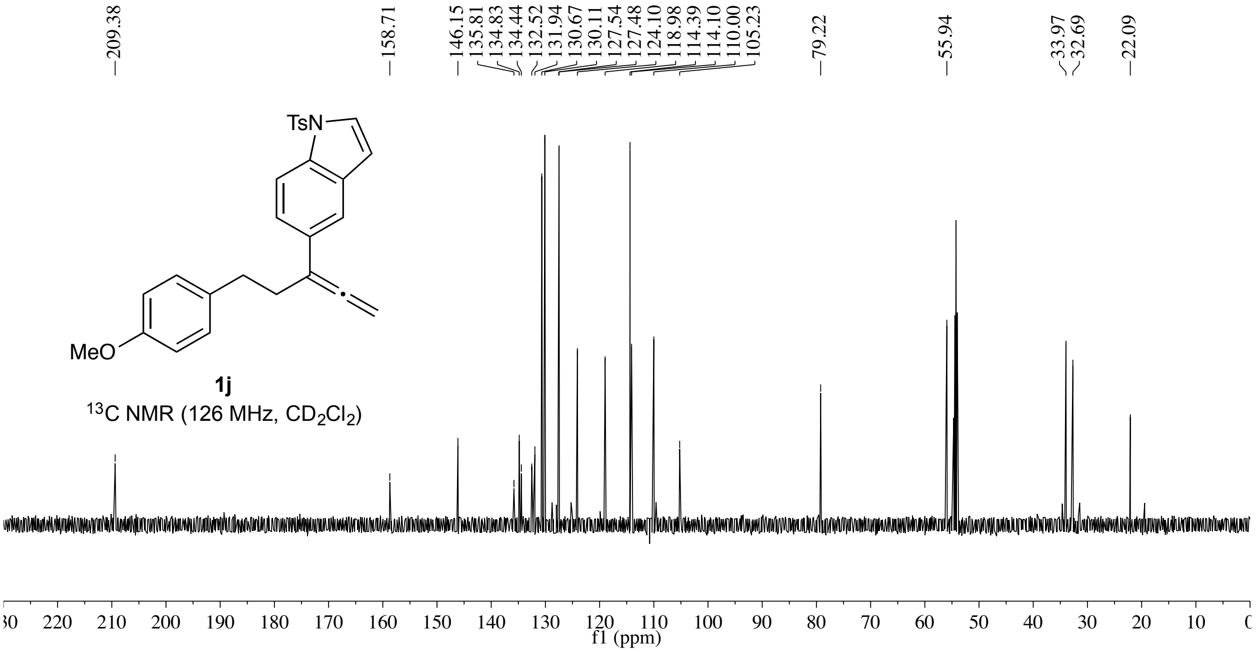

Supplementary Figure 109. ^{1}H NMR spectrum of 1k

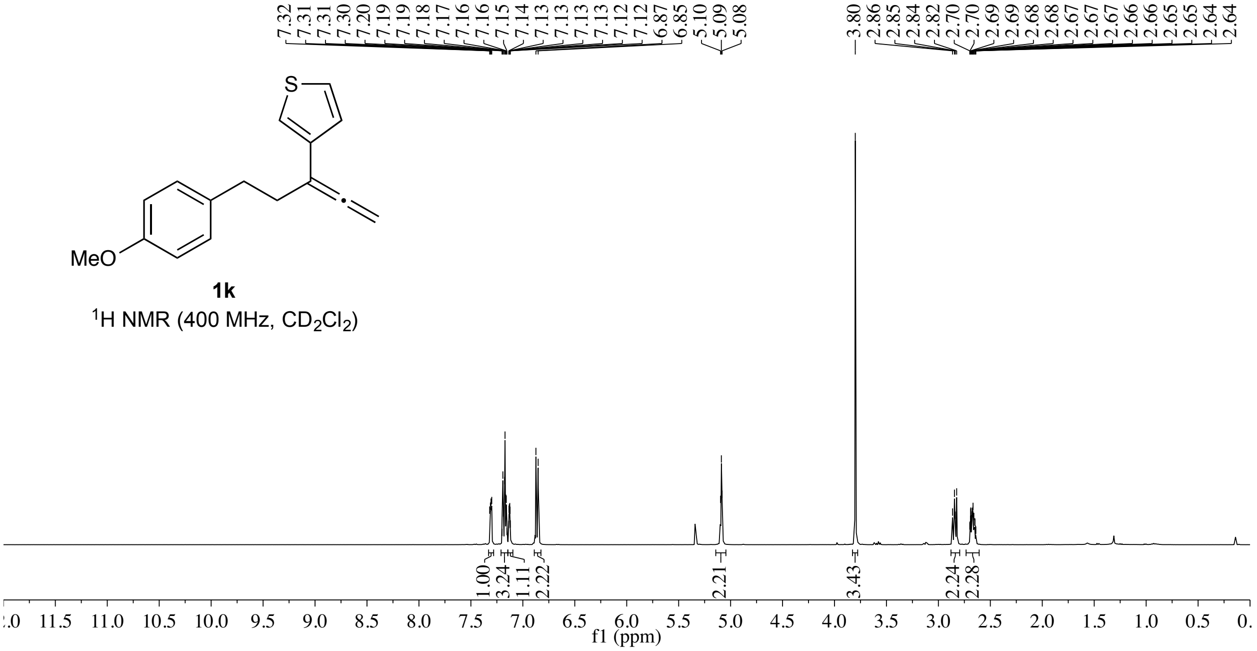

Supplementary Figure 110. ^{13}C NMR spectrum of 1k

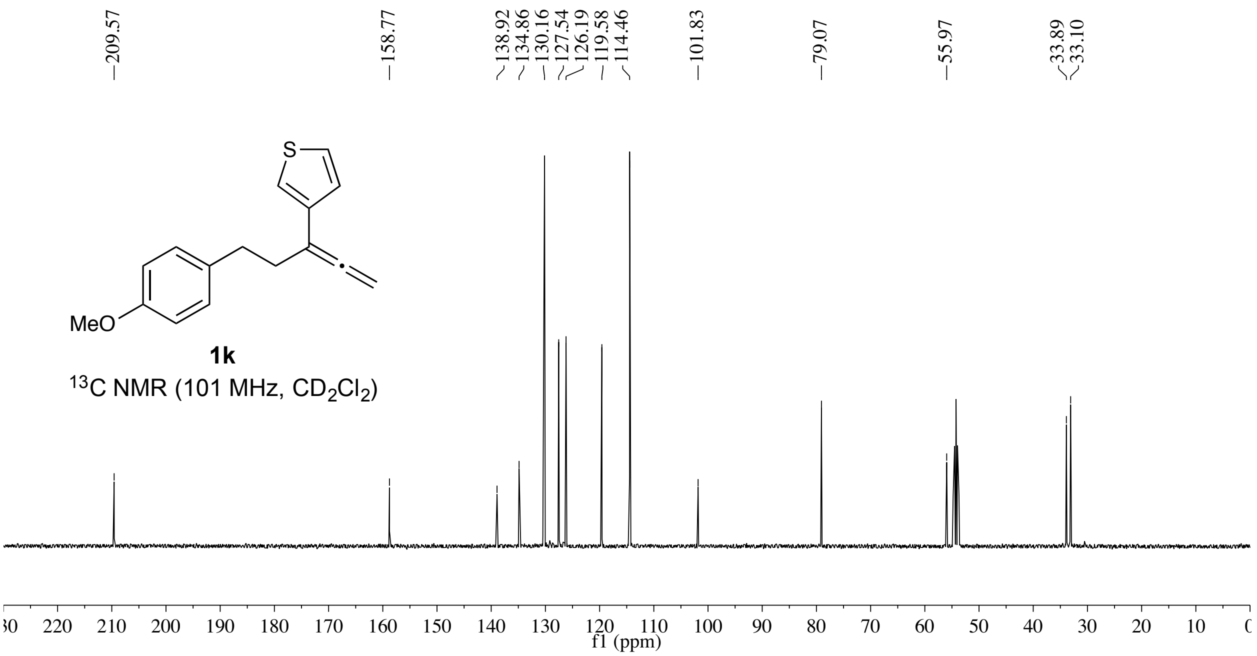

Supplementary Figure 111. ^{1}H NMR spectrum of 1l

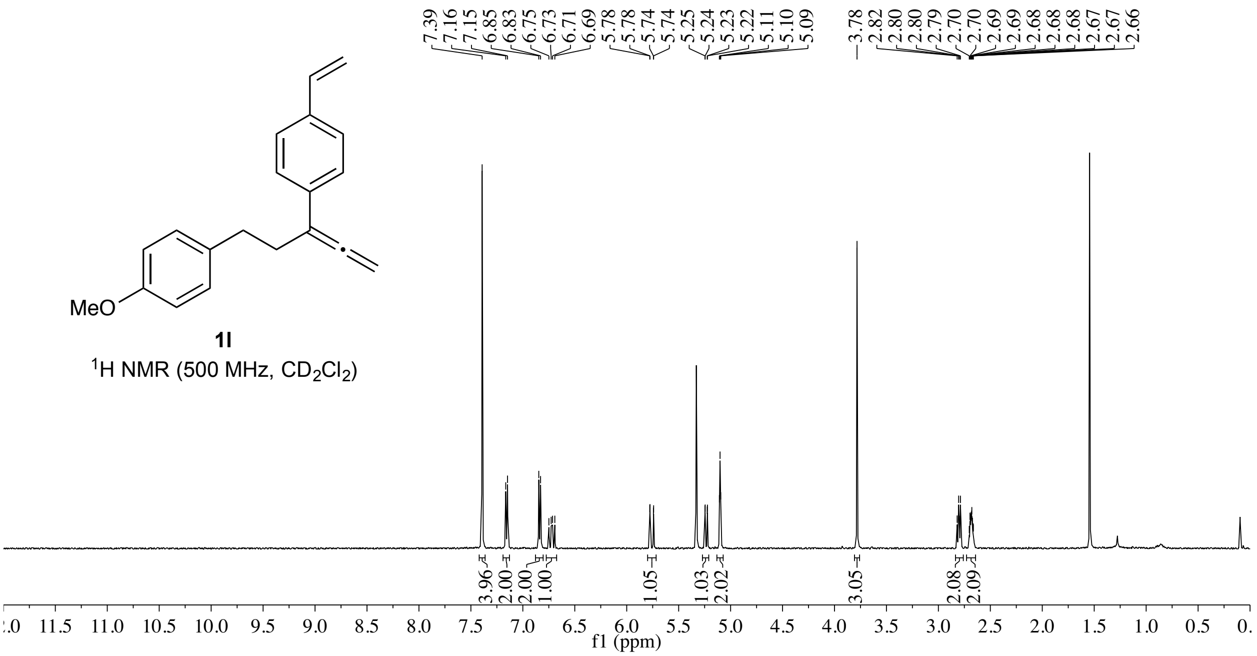

Supplementary Figure 112. ^{13}C NMR spectrum of 1l

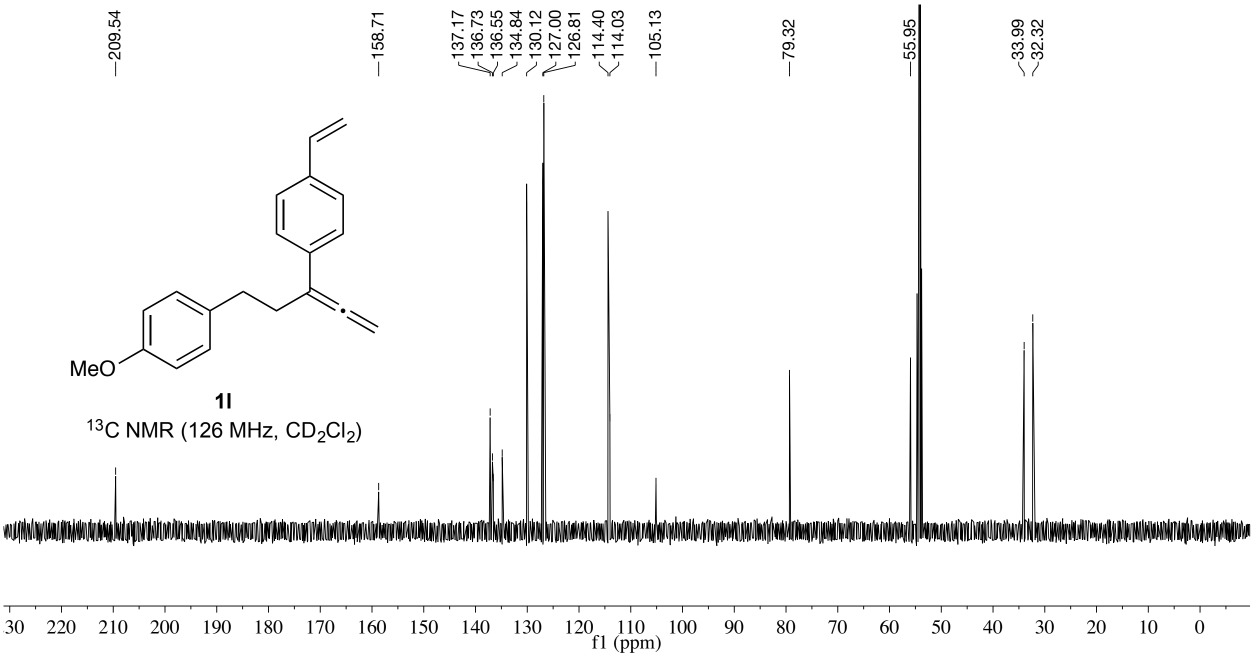

Supplementary Figure 113. ^{1}H NMR spectrum of 1m

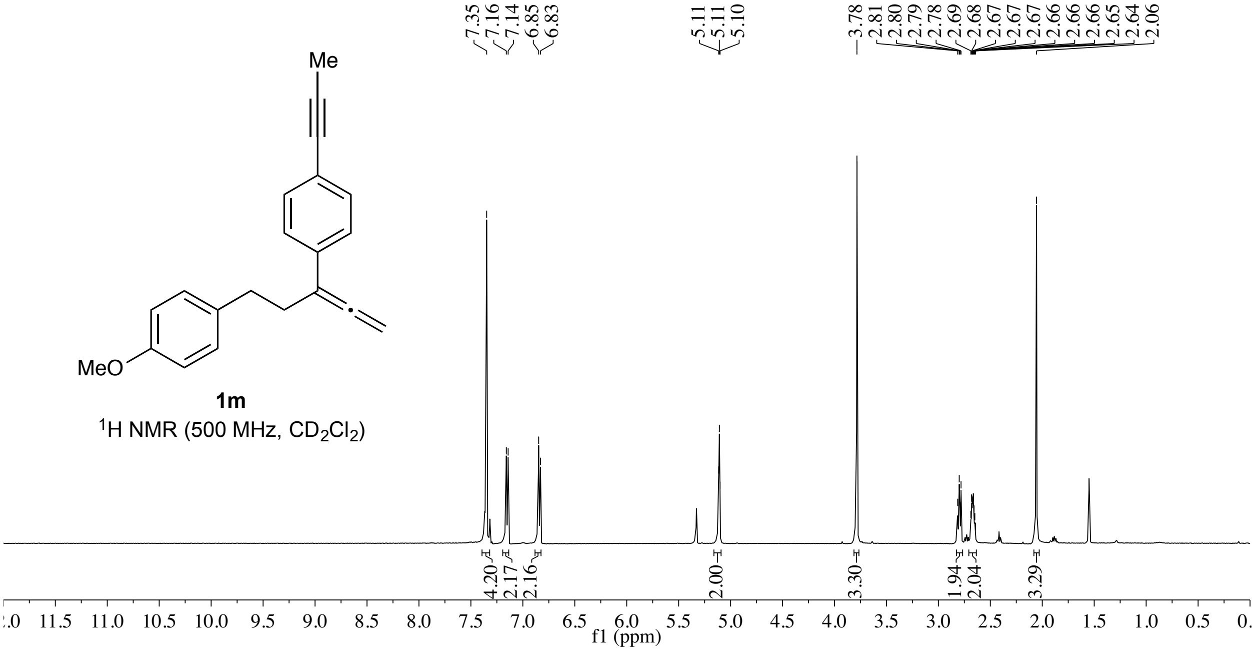

Supplementary Figure 114. ^{13}C NMR spectrum of 1m

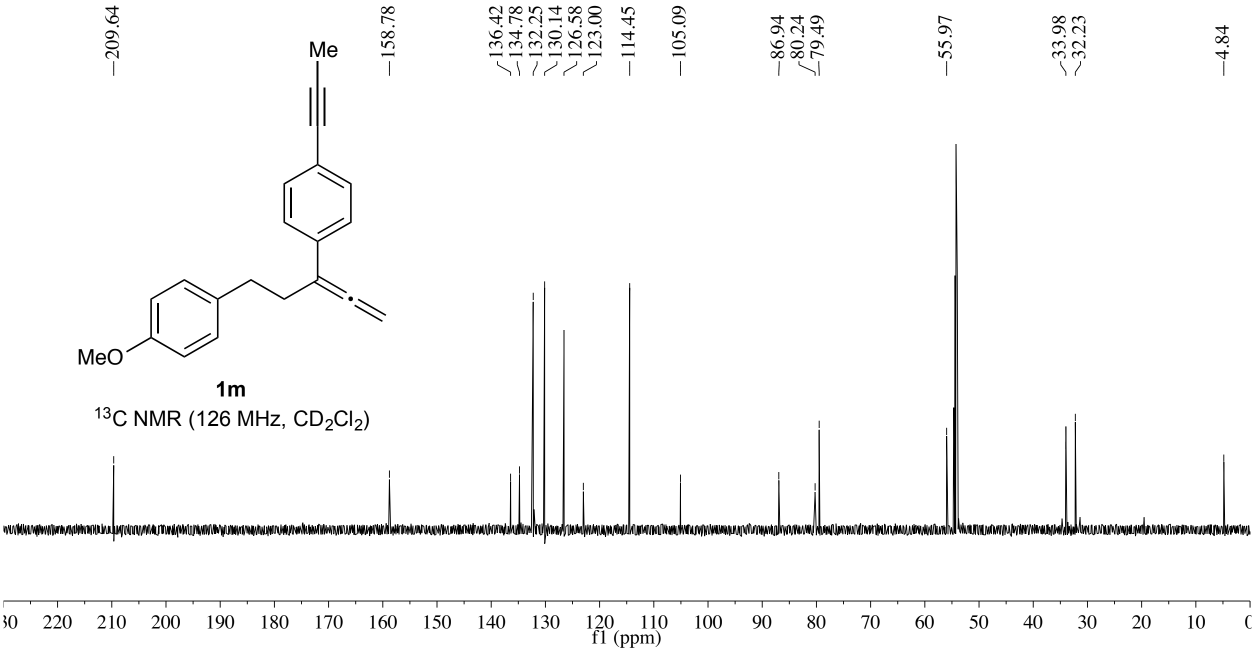

Supplementary Figure 115. ^{1}H NMR spectrum of 1p

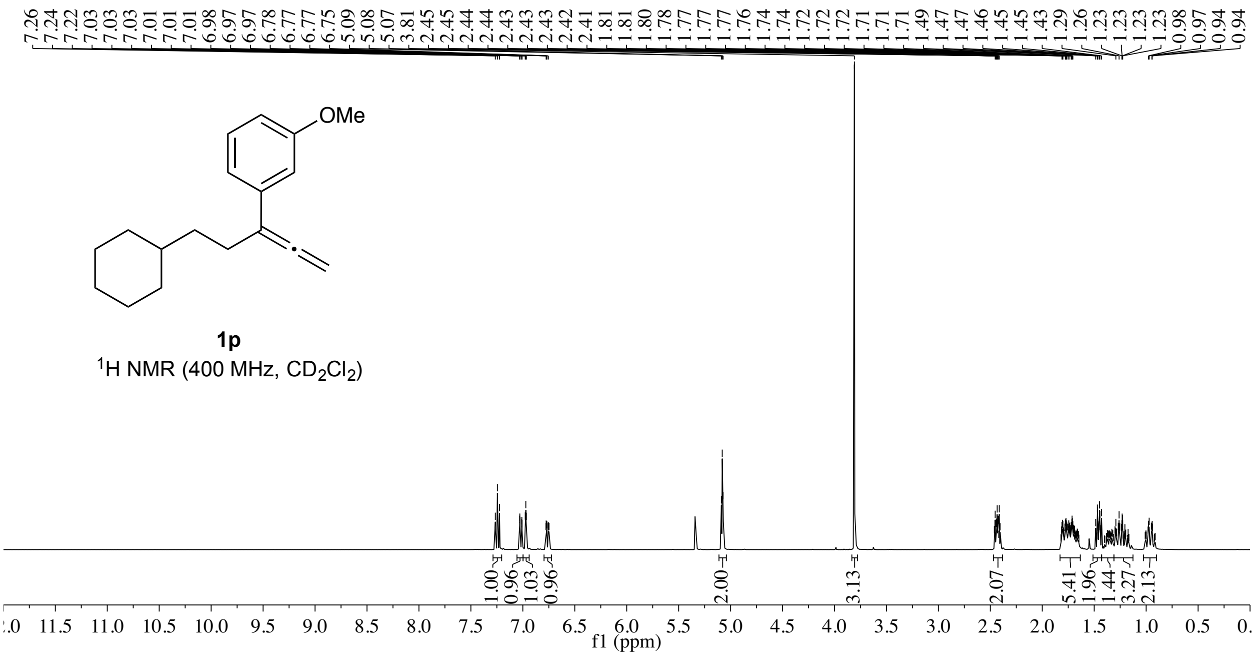

Supplementary Figure 116. ^{13}C NMR spectrum of 1p

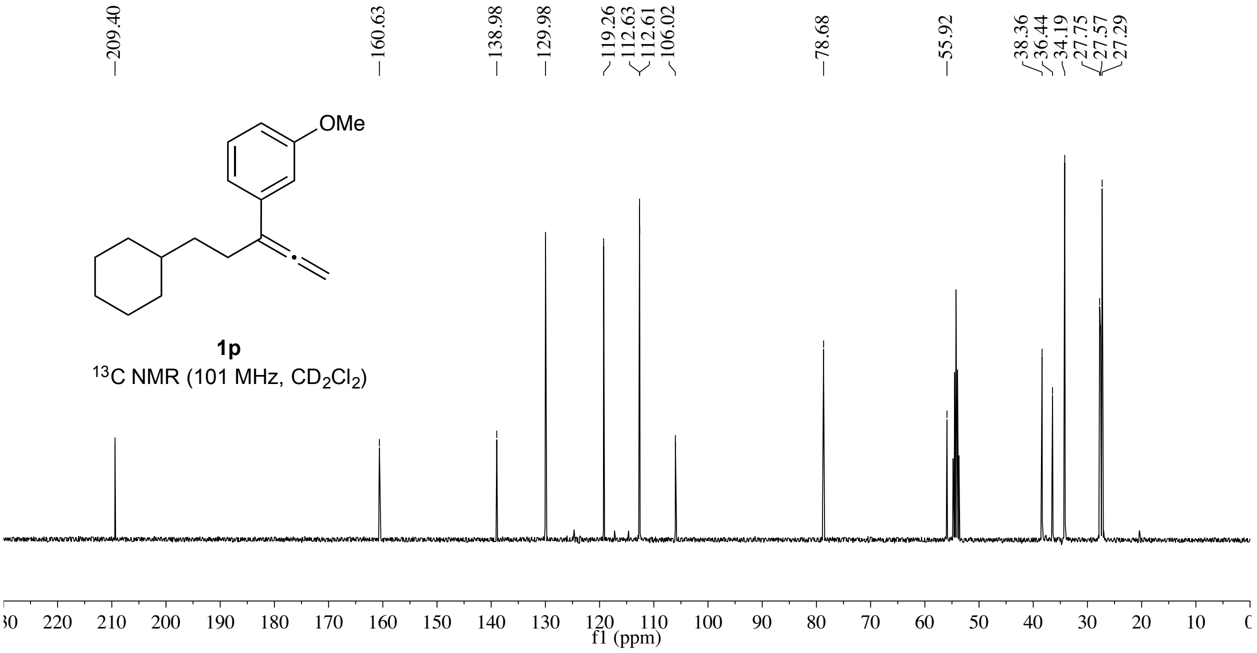

Supplementary Figure 117. ^{1}H NMR spectrum of 1s

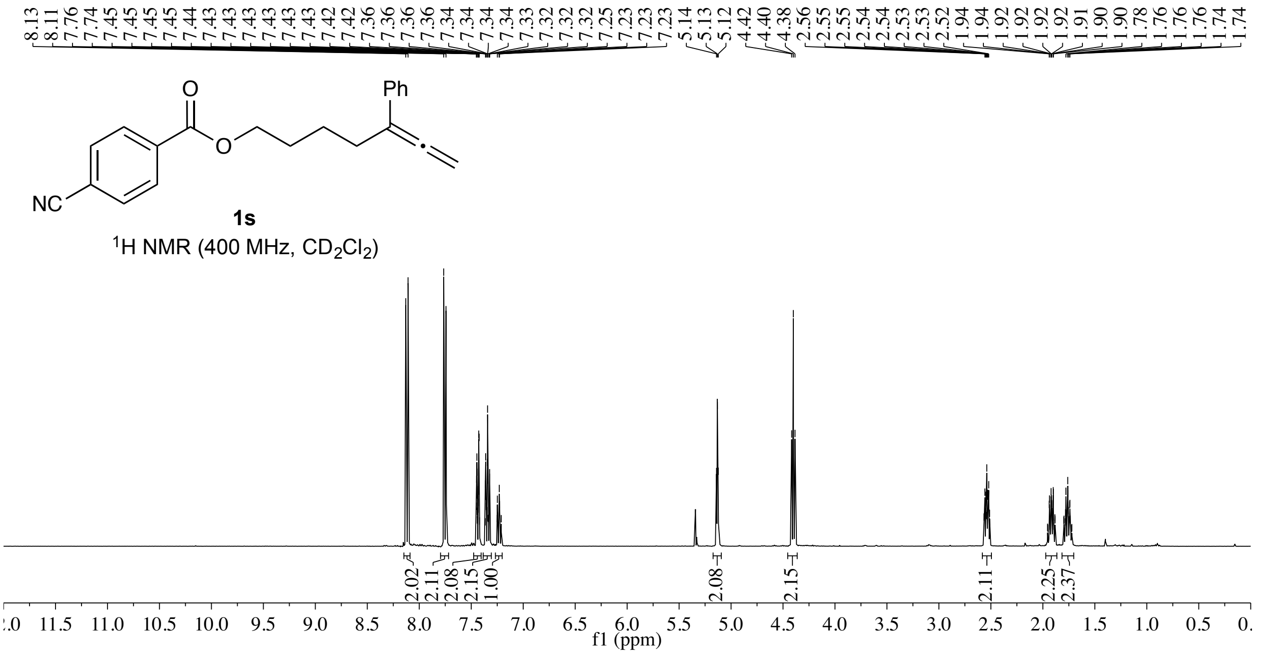

Supplementary Figure 118. ^{13}C NMR spectrum of 1s

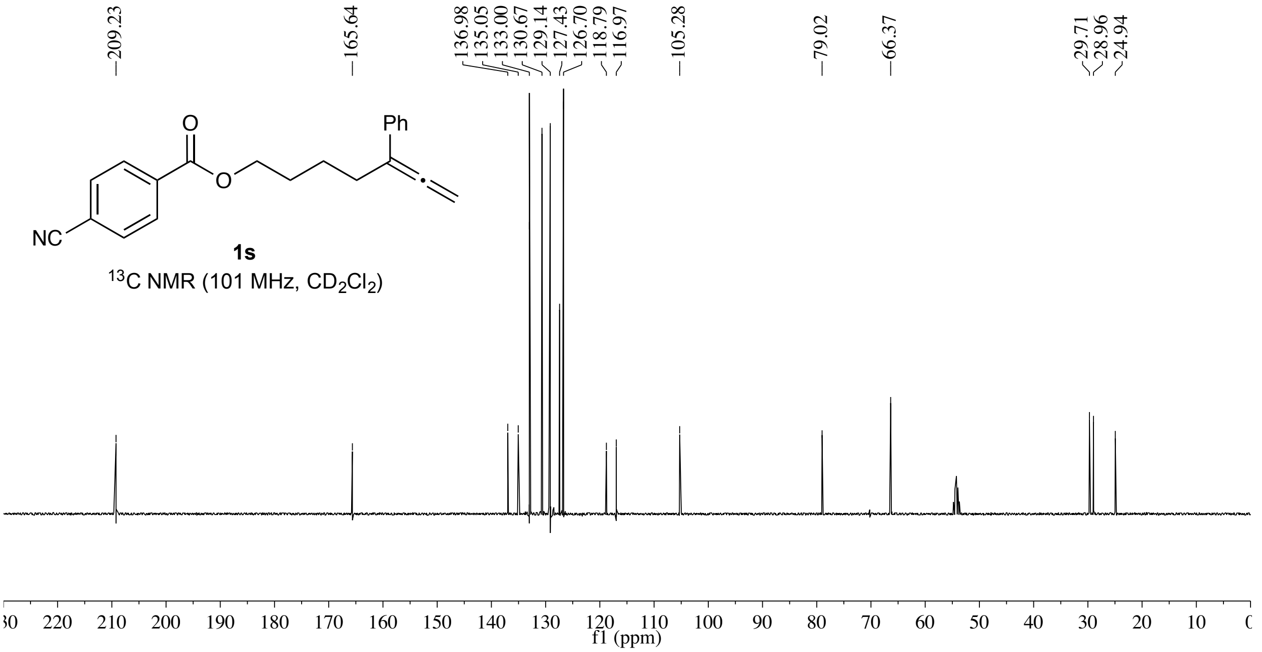

Supplementary Figure 119. ^{1}H NMR spectrum of L6

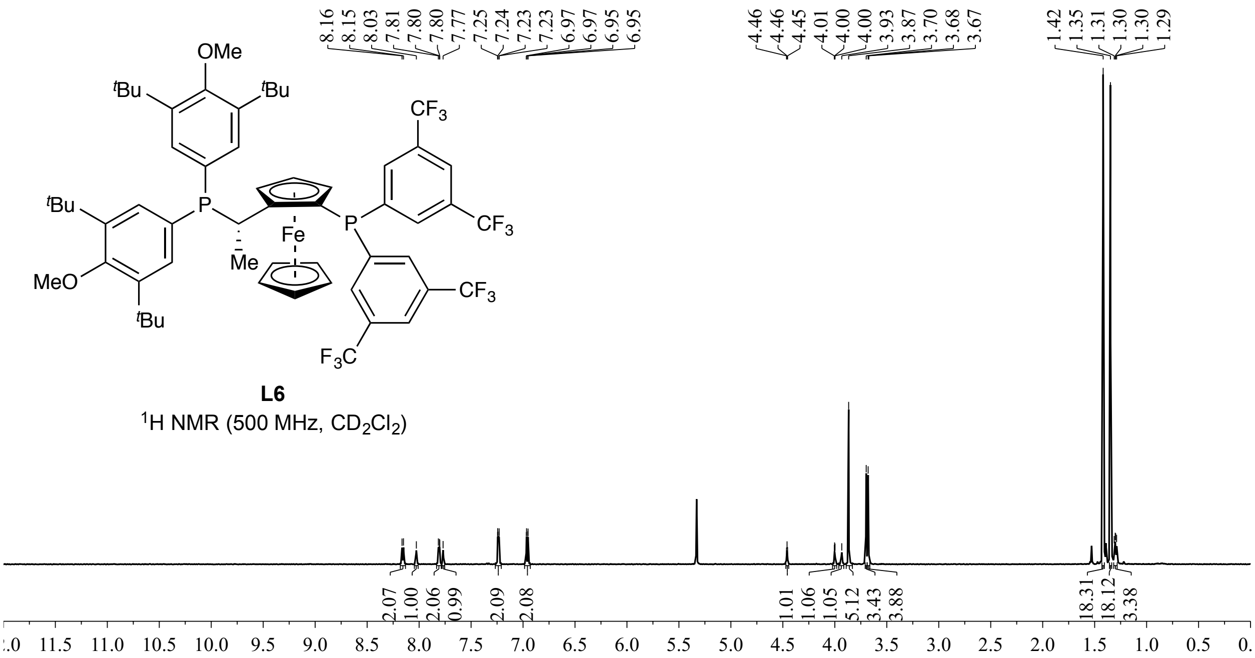

Supplementary Figure 120. ^{13}C NMR spectrum of L6

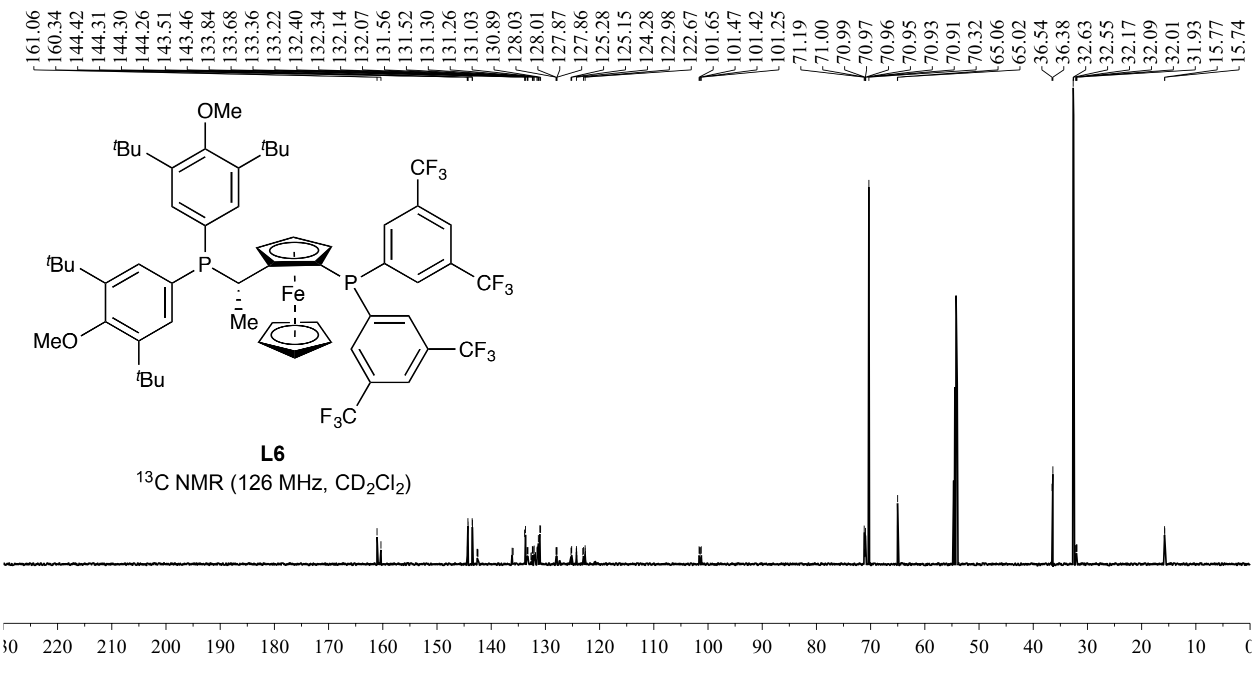

Supplementary Figure 121. ^{31}P NMR spectrum of L6

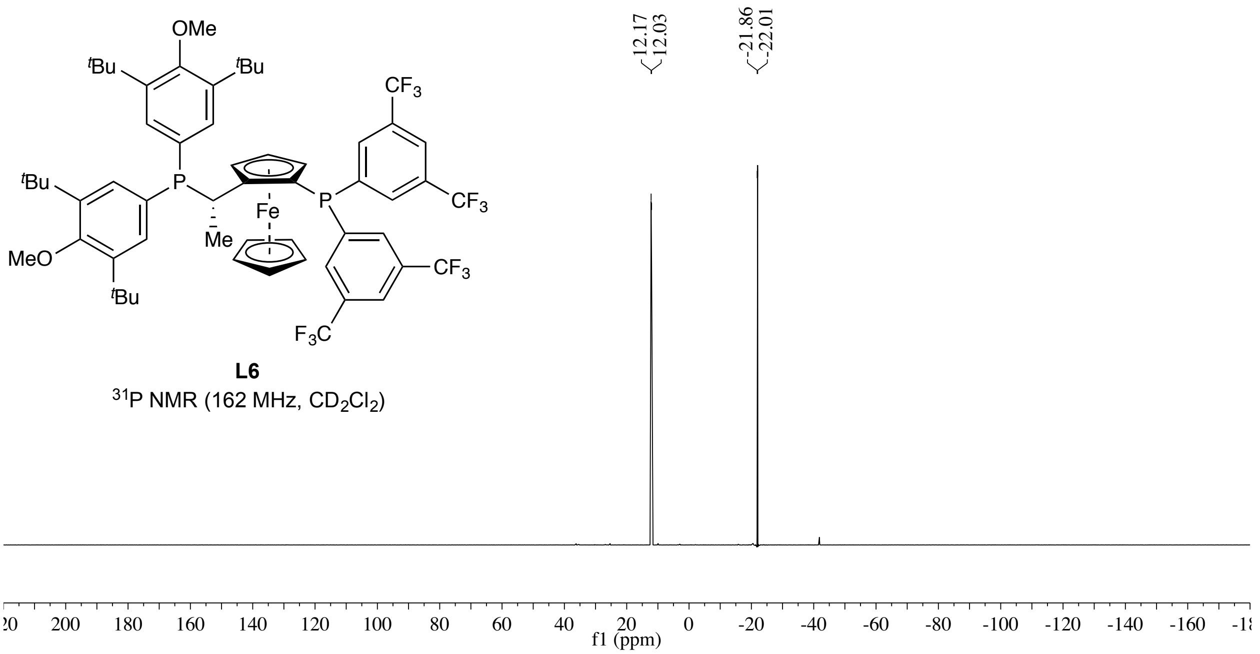

Supplementary Figure 122. ^{19}F NMR spectrum of L6

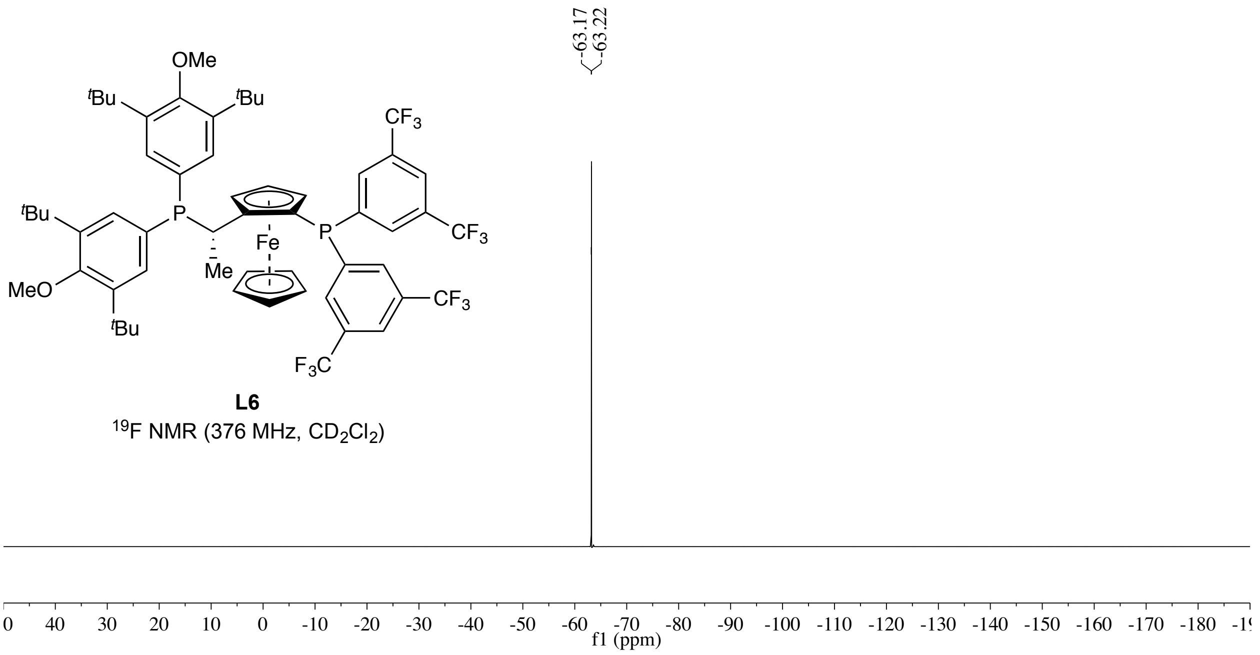

Supplementary Figure 123. ^{1}H NMR spectrum of 2ab

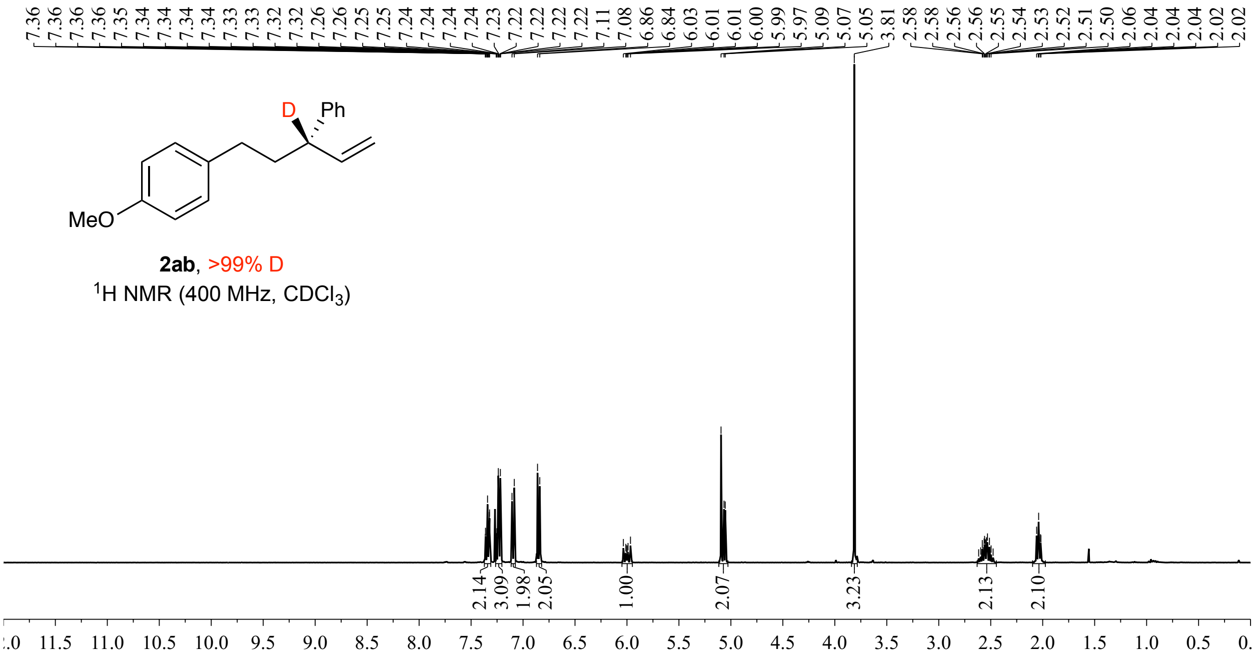

Supplementary Figure 124. ^{13}C NMR spectrum of 2ab

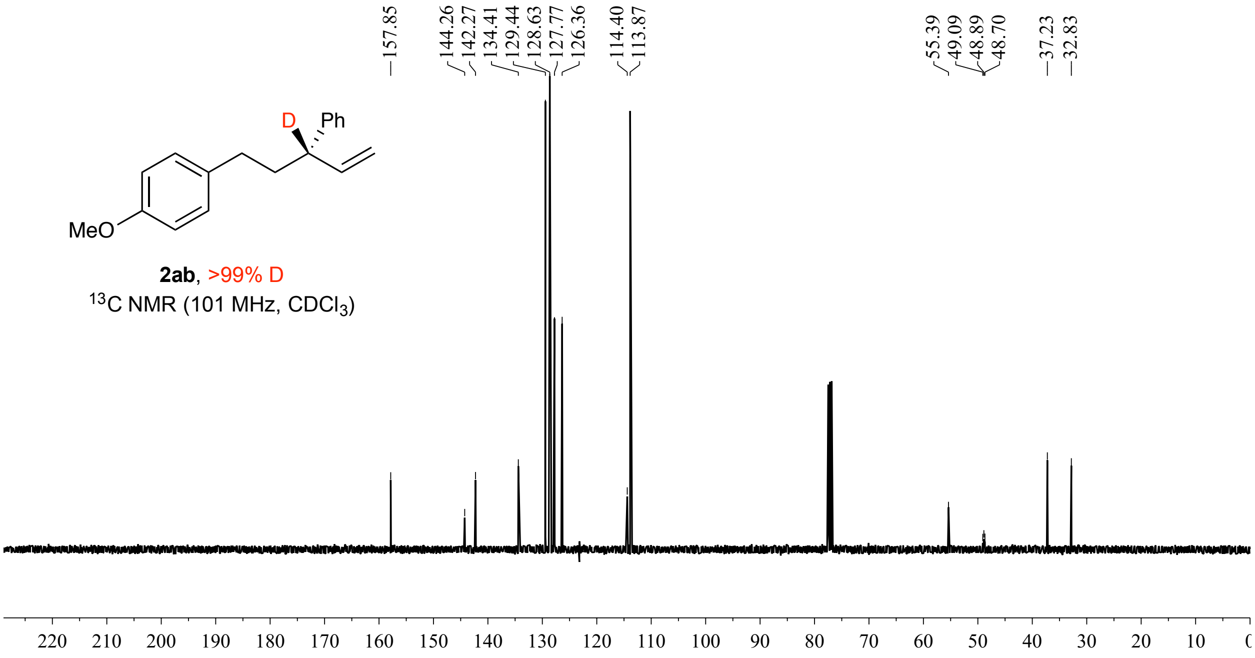

Supplementary Figure 125. ^{1}H NMR spectrum of 2ac

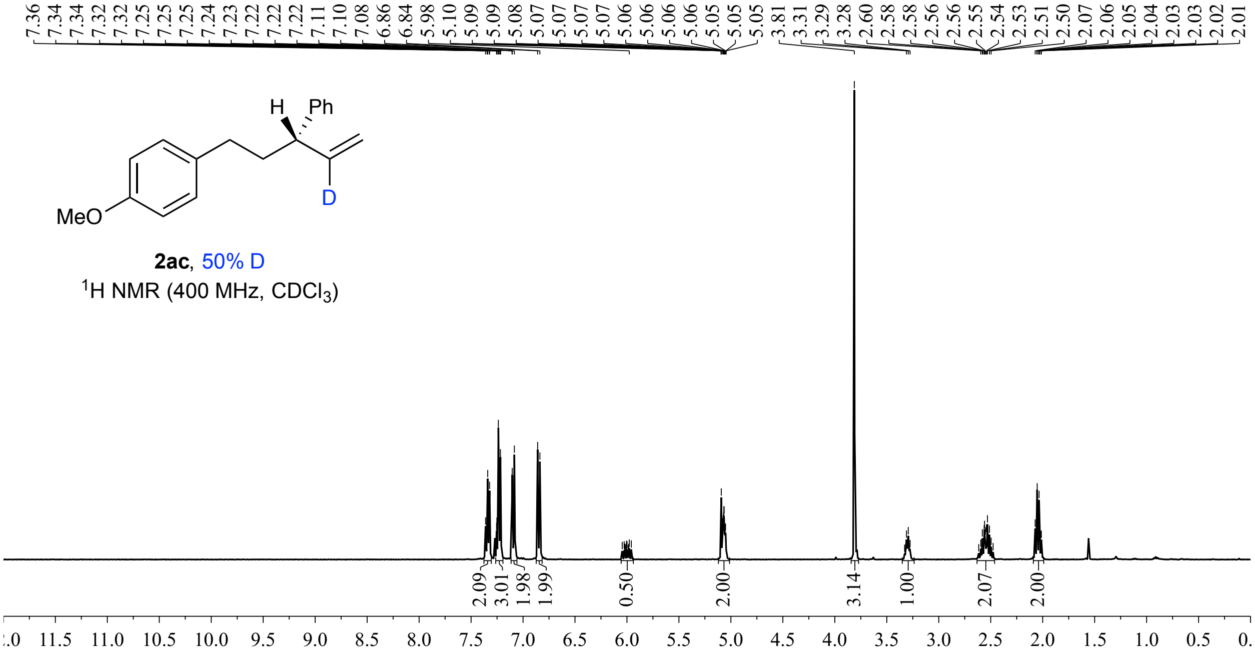

Supplementary Figure 126. ^{13}C NMR spectrum of 2ac

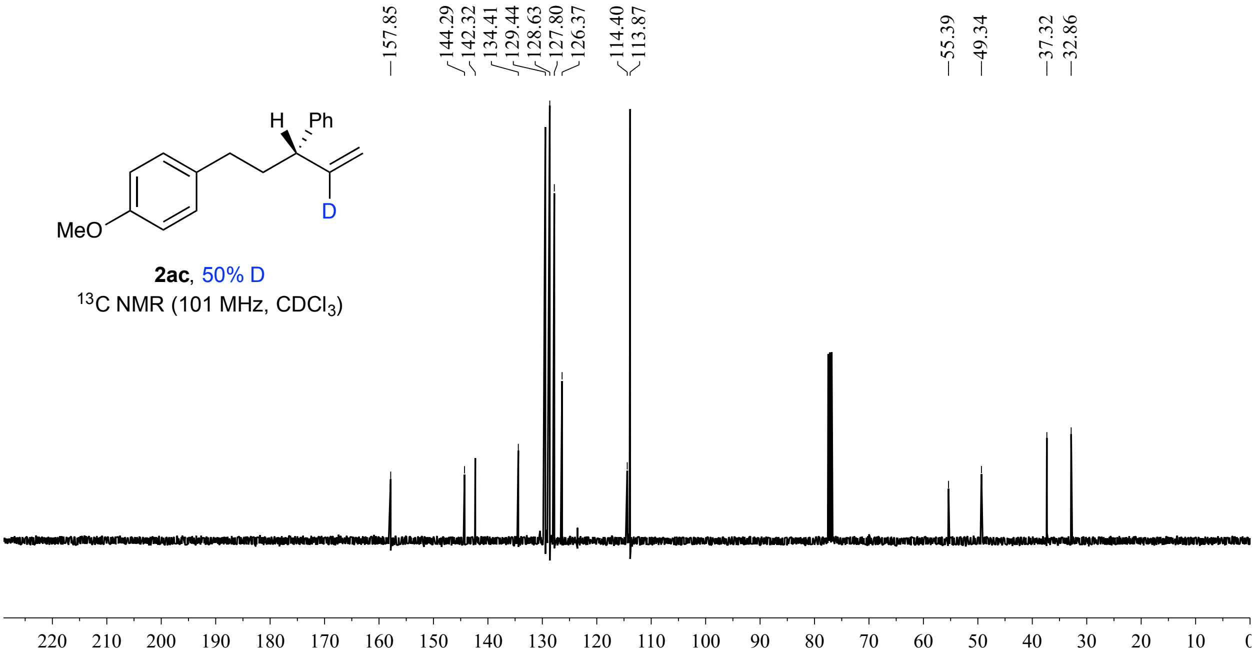

Supplementary Figure 127. SFC trace of (±)-2a

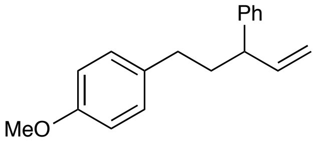

(±)-**2a**

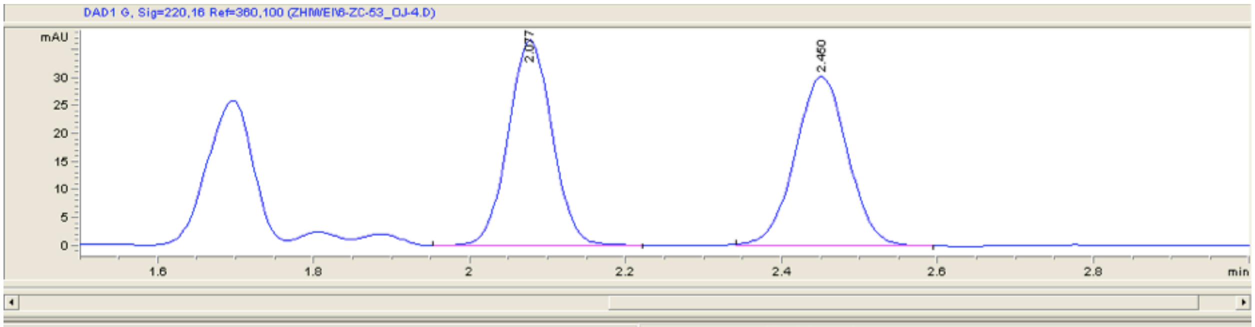

#	Time	Area	Height	Width	Area%	Symmetry
1	2.077	143.7	36.7	0.0617	50.463	0.938
2	2.45	141	30.2	0.0725	49.537	0.984

LC-File	6-ZC-53_OJ-4.D
File Path	C:\CHEM32\1\DATA\ZHIWEI\
Date	12-Aug-16, 22:59:01

* peak at 1.7 min is residual internal alkene that was not completely separated from the product

Supplementary Figure 128. SFC trace of (+)-2a

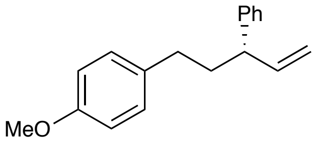

(+)-**2a**

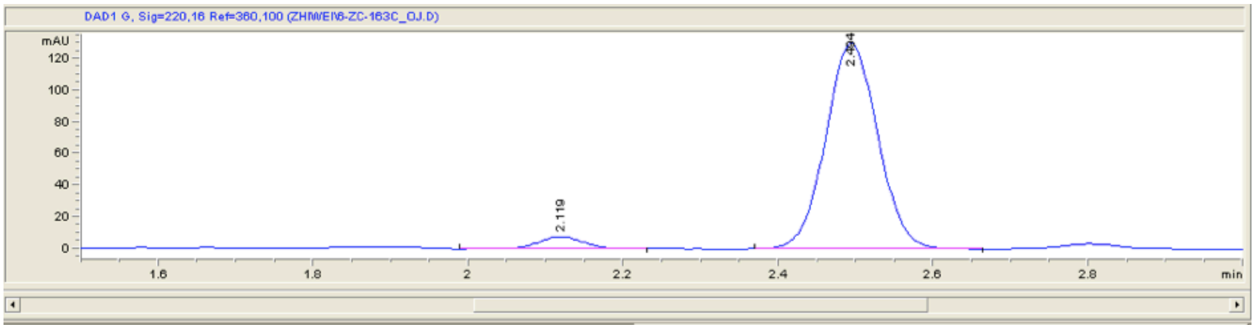

#	Time	Area	Height	Width	Area%	Symmetry
1	2.119	31.1	7.6	0.0635	4.887	0.914
2	2.494	604.4	129.4	0.0745	95.113	0.934

LC-File	6-ZC-163C_OJ.D
File Path	C:\CHEM32\1\DATA\ZHIWEI\
Date	03-Oct-16, 12:01:54

Supplementary Figure 129. SFC trace of (±)-2b

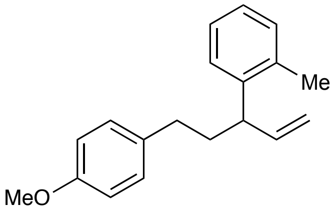

(±)-**2b**

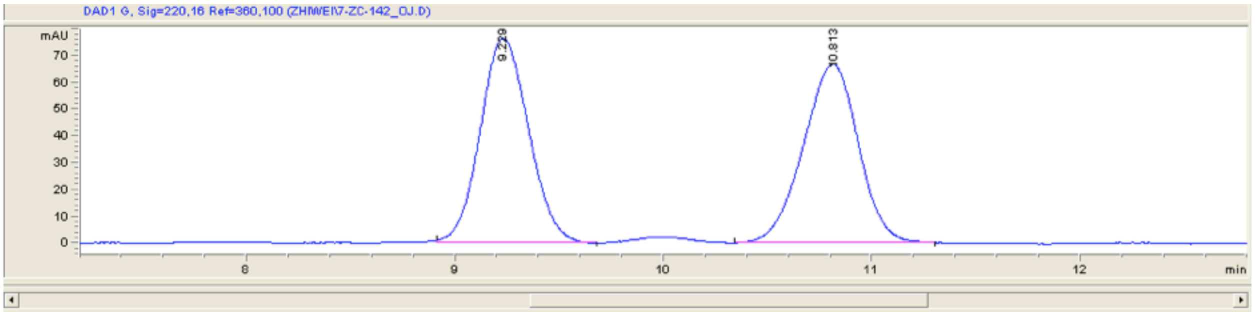

#	Time	Area	Height	Width	Area%	Symmetry
1	9.229	1231.4	76.3	0.2504	49.928	0.864
2	10.813	1235	66.9	0.2832	50.072	1.132

File Information	
LC-File	7-ZC-142_OJ.D
File Path	C:\CHEM32\1\DATA\ZHIWEI\
Date	15-Jan-17, 15:16:50

Supplementary Figure 130. SFC trace of (+)-2b

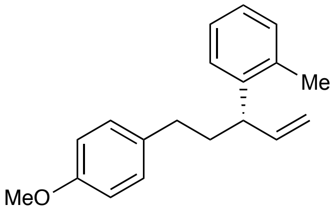

(+)-**2b**

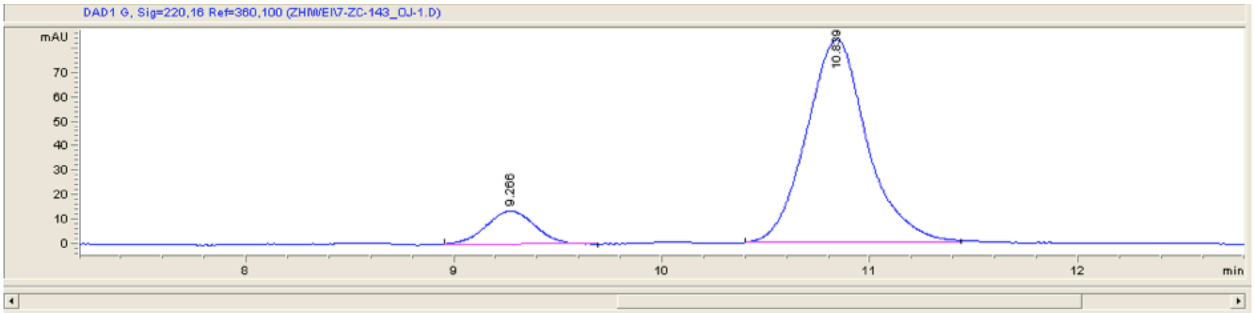

#	Time	Area	Height	Width	Area%	Symmetry
1	9.266	224.8	13.7	0.2138	11.686	0.914
2	10.839	1698.9	83.1	0.3084	88.314	0.939

File Information	
LC-File	7-ZC-143_OJ-1.D
File Path	C:\CHEM32\1\DATA\ZHIWEI\
Date	15-Jan-17, 16:08:21

Supplementary Figure 131. SFC trace of (±)-2c

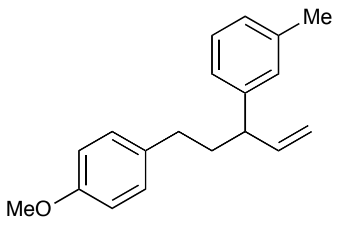

(±)-**2c**

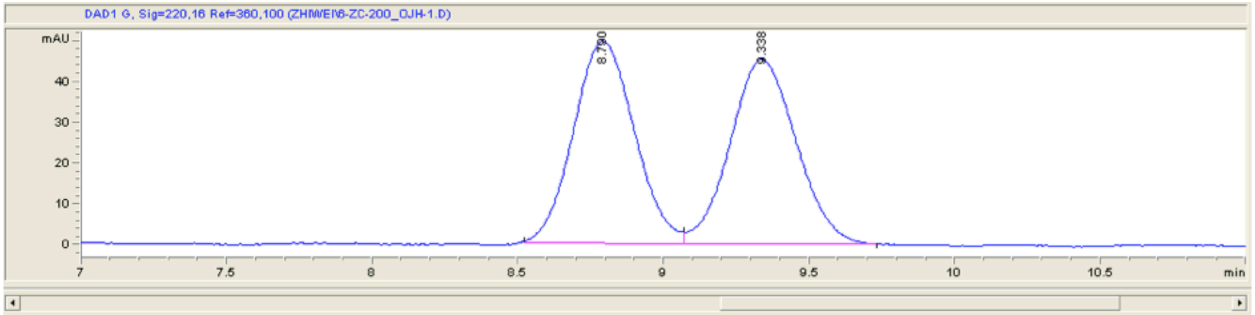

#	Time	Area	Height	Width	Area%	Symmetry
1	8.79	727.2	49.6	0.224	50.343	0.89
2	9.338	717.3	45.3	0.2379	49.657	0.904

File Information

LC-File	6-ZC-200_OJH-1.D
File Path	C:\CHEM32\1\DATA\ZHIWEI\
Date	30-Jan-17, 18:38:49

Supplementary Figure 132. SFC trace of (+)-2c

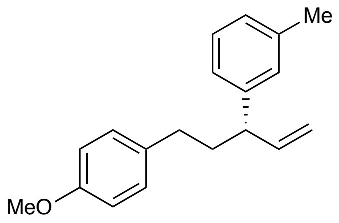

(+)-**2c**

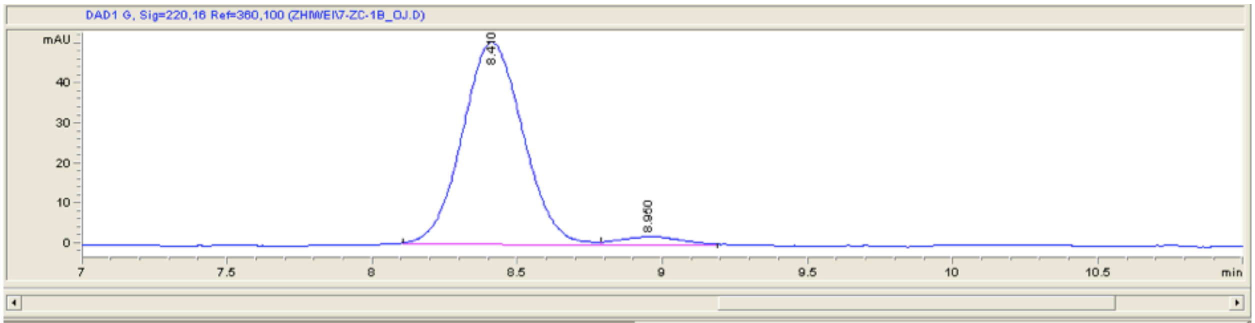

#	Time	Area	Height	Width	Area%	Symmetry
1	8.41	740.9	50.7	0.2235	95.896	0.911
2	8.95	31.7	2.1	0.1941	4.104	0.816

File Information

LC-File	7-ZC-1B_OJ.D
File Path	C:\CHEM32\1\DATA\ZHIWEI\
Date	21-Oct-16, 09:24:55
Sample	7-ZC-1b_OJ

Supplementary Figure 133. SFC trace of (±)-2d

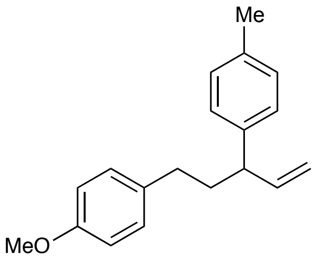

(±)-**2d**

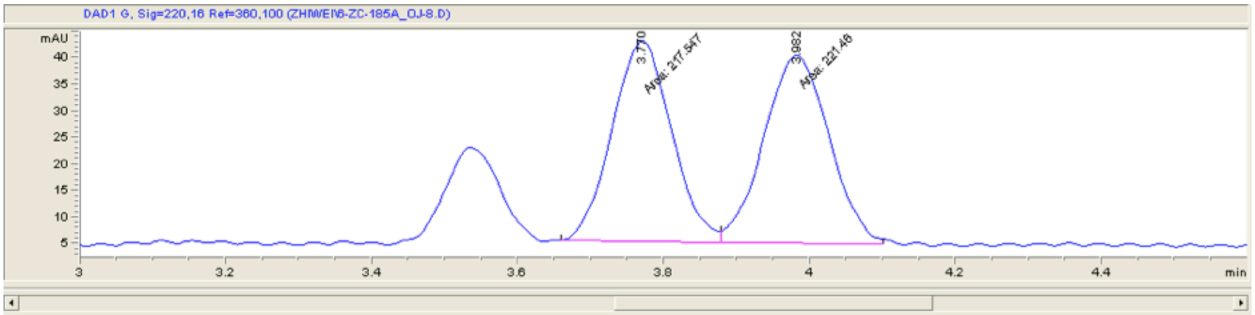

#	Time	Area	Height	Width	Area%	Symmetry
1	3.77	217.5	37.9	0.0956	49.554	0.965
2	3.982	221.5	35.6	0.1036	50.446	0.935

File Information	
LC-File	6-ZC-185A_OJ-8.D
File Path	C:\CHEM32\1\DATA\ZHIWEI\
Date	17-Oct-16, 12:53:33

* peak at 3.5 min is residual internal alkene that was not completely separated from the product

Supplementary Figure 134. SFC trace of (+)-2d

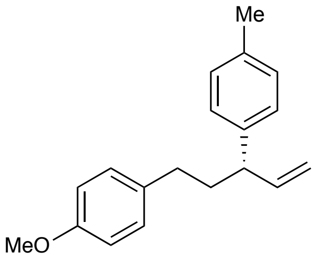

(+)-**2d**

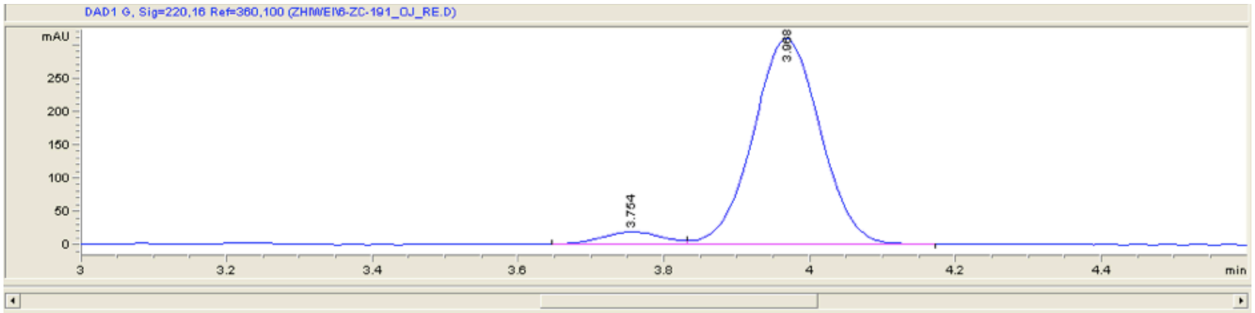

#	Time	Area	Height	Width	Area%	Symmetry
1	3.754	106.3	18.7	0.0865	5.113	0.912
2	3.968	1973.5	308.1	0.0997	94.887	0.977

File Information	
LC-File	6-ZC-191_OJ_RE.D
File Path	C:\CHEM32\1\DATA\ZHIWEI\
Date	17-Oct-16, 13:15:31
Sample	6-ZC-191_OJ_re

Supplementary Figure 135. SFC trace of (±)-2e

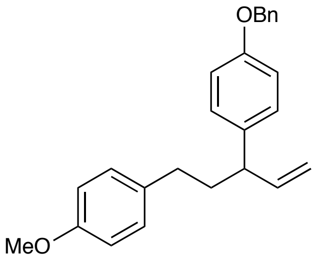

(±)-**2e**

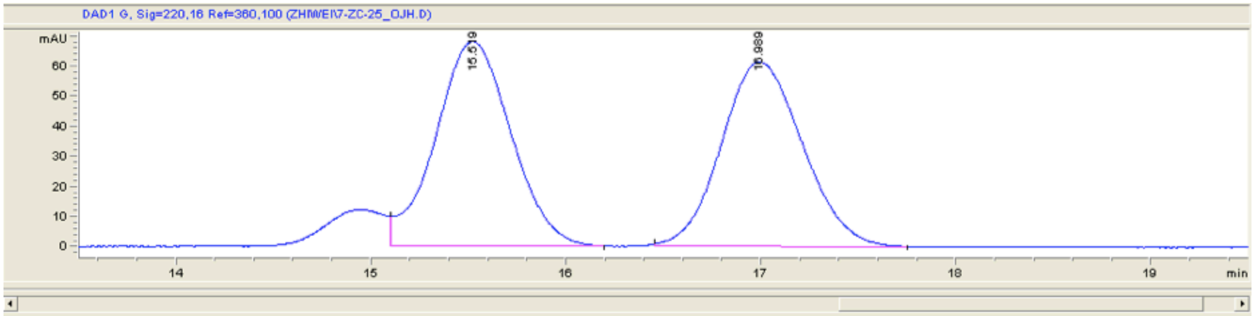

#	Time	Area	Height	Width	Area%	Symmetry
1	15.519	1826.3	68	0.3885	50.673	0.941
2	16.989	1777.8	61.5	0.3955	49.327	0.845

File Information

LC-File	7-ZC-25_OJH.D
File Path	C:\CHEM32\1\DATA\ZHIWEI\
Date	30-Jan-17, 20:32:55

* peak at 14.9 min is residual internal alkene that was not completely separated from the product

Supplementary Figure 136. SFC trace of (+)-2e

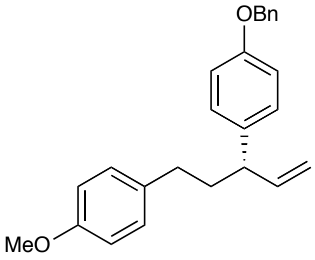

(+)-**2e**

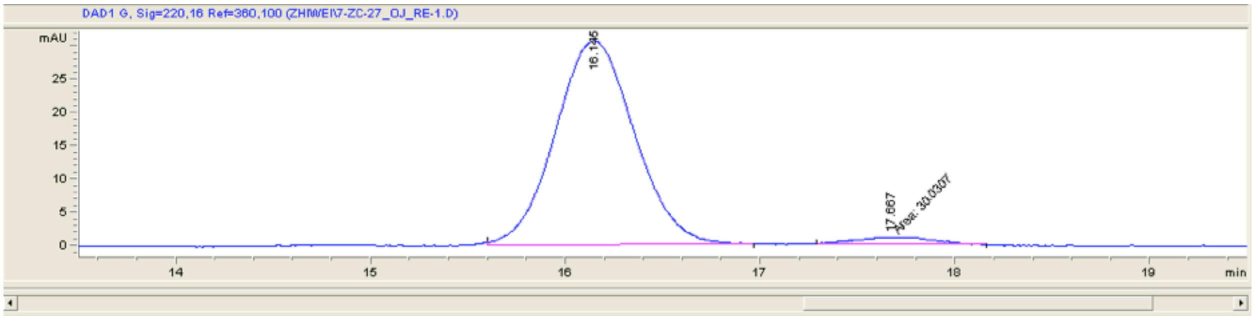

#	Time	Area	Height	Width	Area%	Symmetry
1	16.145	865.8	30.7	0.4414	96.648	0.921
2	17.667	30	1.1	0.4575	3.352	0.794

File Information

LC-File	7-ZC-27_OJ_RE-1.D
File Path	C:\CHEM32\1\DATA\ZHIWEI\
Date	01-Nov-16, 22:48:01

Supplementary Figure 137. SFC trace of (±)-2f

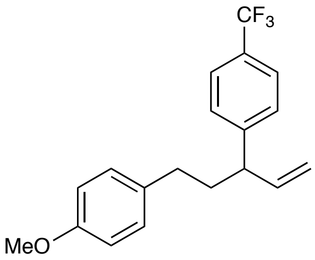

(±)-**2f**

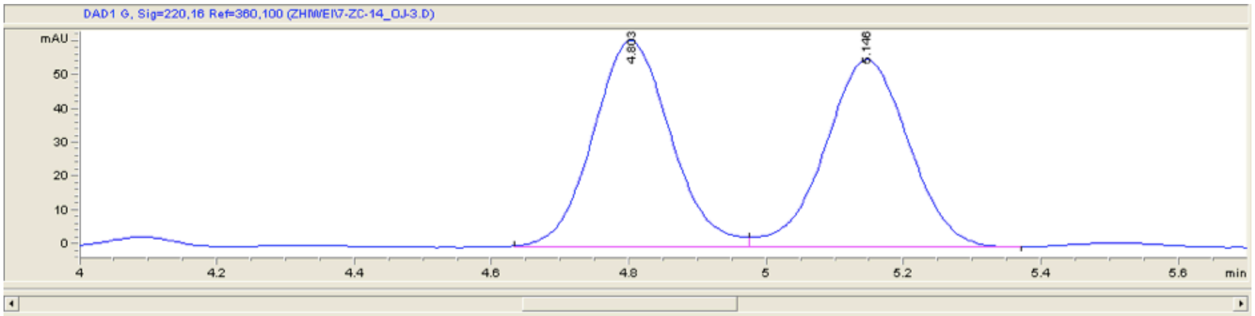

#	Time	Area	Height	Width	Area%	Symmetry
1	4.803	483.7	60.9	0.1247	50.558	0.917
2	5.146	473	55.3	0.1358	49.442	0.981

File Information

LC-File	7-ZC-14_OJ-3.D
File Path	C:\CHEM32\1\DATA\ZHIWEI\
Date	26-Oct-16, 21:53:32

Supplementary Figure 138. SFC trace of (+)-2f

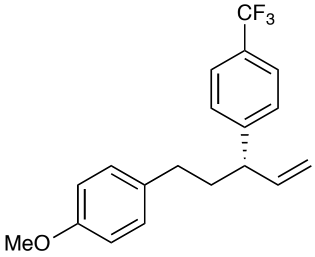

(+)-**2f**

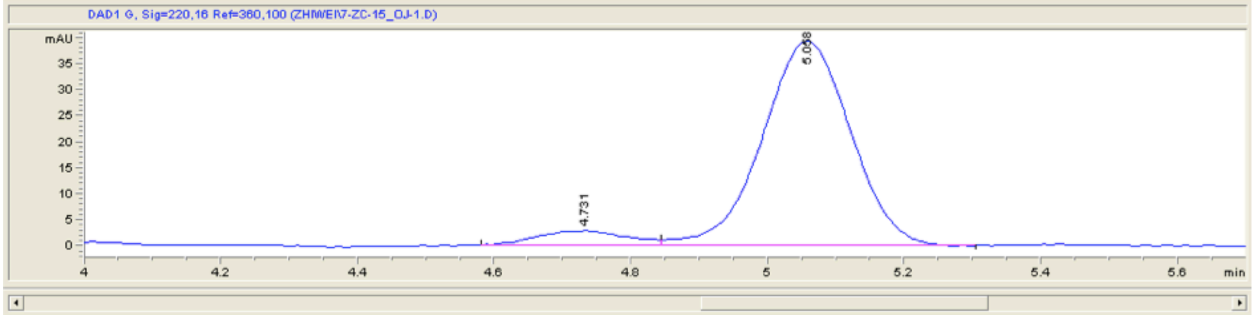

#	Time	Area	Height	Width	Area%	Symmetry
1	4.731	26.9	2.9	0.1171	7.093	1.095
2	5.058	352.9	39.2	0.139	92.907	1.023

File Information

LC-File	7-ZC-15_OJ-1.D
File Path	C:\CHEM32\1\DATA\ZHIWEI\
Date	27-Oct-16, 12:53:04

Supplementary Figure 139. SFC trace of (±)-2g

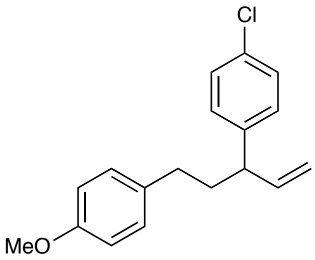

(±)-**2g**

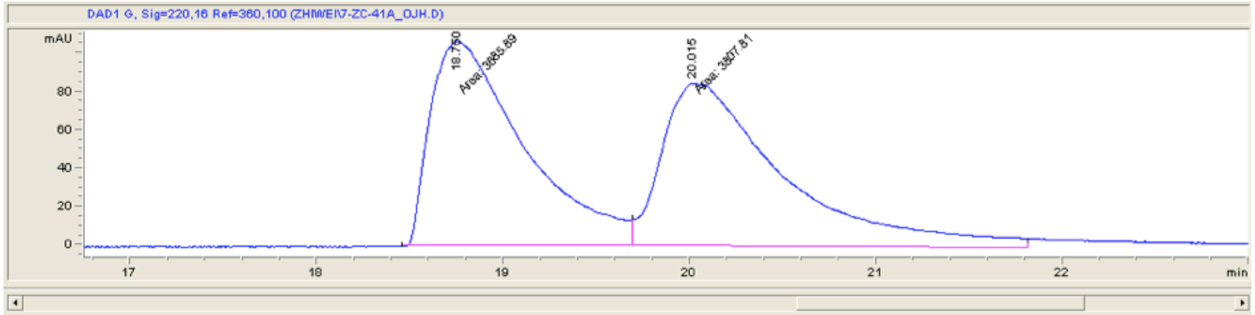

#	Time	Area	Height	Width	Area%	Symmetry
1	18.75	3685.9	106.3	0.5778	49.187	0.379
2	20.015	3807.8	84.4	0.7516	50.813	0.331

File Information

LC-File	7-ZC-41A_OJH.D
File Path	C:\CHEM32\1\DATA\ZHIWEI\
Date	29-Jan-17, 22:18:18

Supplementary Figure 140. SFC trace of (+)-2g

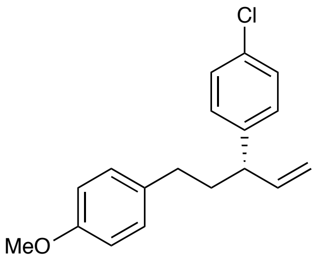

(+)-**2g**

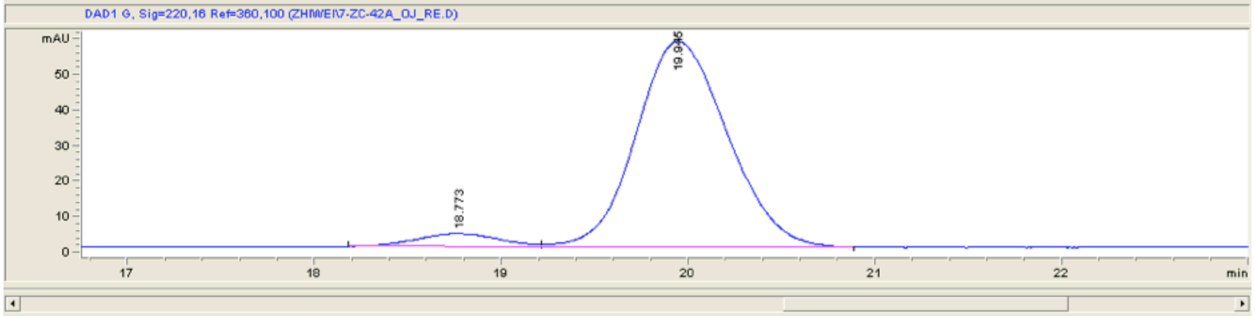

#	Time	Area	Height	Width	Area%	Symmetry
1	18.773	117.2	3.7	0.3904	5.620	1.052
2	19.945	1968.6	57.9	0.5245	94.380	0.83

File Information

LC-File	7-ZC-42A_OJ_RE.D
File Path	C:\CHEM32\1\DATA\ZHIWEI\
Date	09-Nov-16, 12:45:44

Supplementary Figure 141. SFC trace of (±)-2h

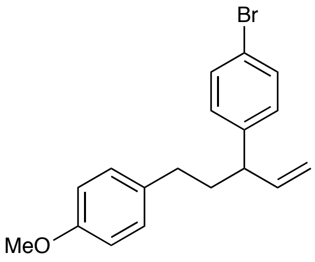

(±)-**2h**

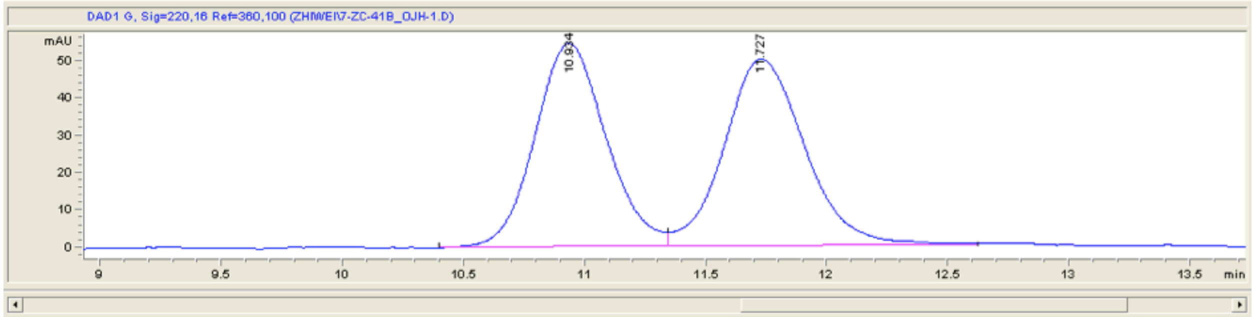

#	Time	Area	Height	Width	Area%	Symmetry
1	10.934	1117.8	54.5	0.3134	48.901	0.886
2	11.727	1168	50	0.3603	51.099	0.812

File Information	
LC-File	7-ZC-41B_OJH-1.D
File Path	C:\CHEM32\1\DATA\ZHIWEI\
Date	30-Jan-17, 00:37:46

Supplementary Figure 142. SFC trace of (+)-2h

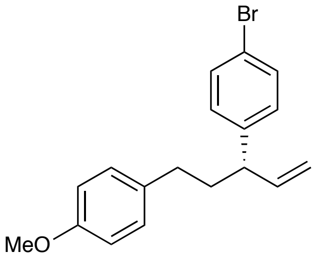

(+)-**2h**

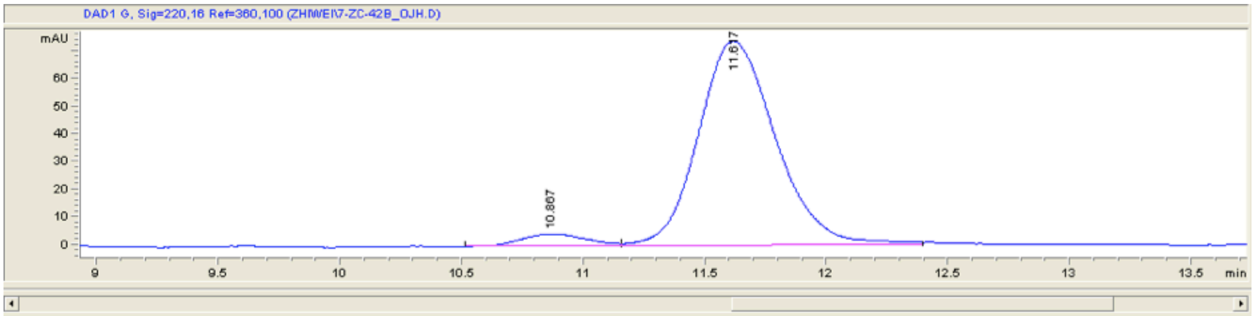

#	Time	Area	Height	Width	Area%	Symmetry
1	10.867	82.8	4.4	0.2764	4.821	0.872
2	11.617	1634	73.9	0.3401	95.179	0.796

File Information	
LC-File	7-ZC-42B_OJH.D
File Path	C:\CHEM32\1\DATA\ZHIWEI\
Date	30-Jan-17, 17:43:49

Supplementary Figure 143. SFC trace of (±)-2i

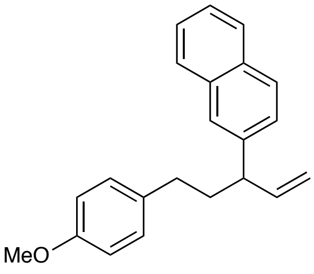

(±)-**2i**

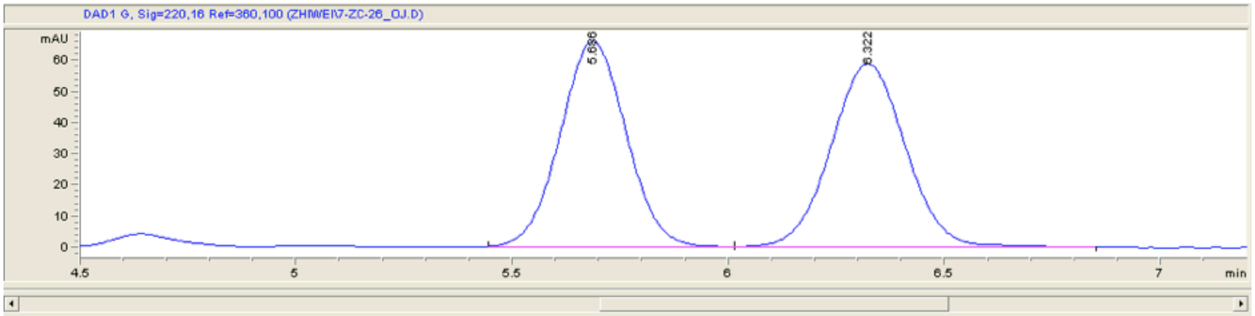

#	Time	Area	Height	Width	Area%	Symmetry
1	5.686	707.4	66.1	0.1668	49.739	0.957
2	6.322	714.9	59.1	0.1892	50.261	0.941

File Information	
LC-File	7-ZC-26_OJ.D
File Path	C:\CHEM32\1\DATA\ZHIWEI\
Date	01-Nov-16, 23:21:30

Supplementary Figure 144. SFC trace of (+)-2i

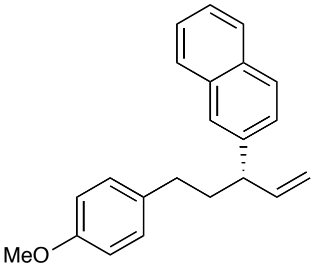

(+)-**2i**

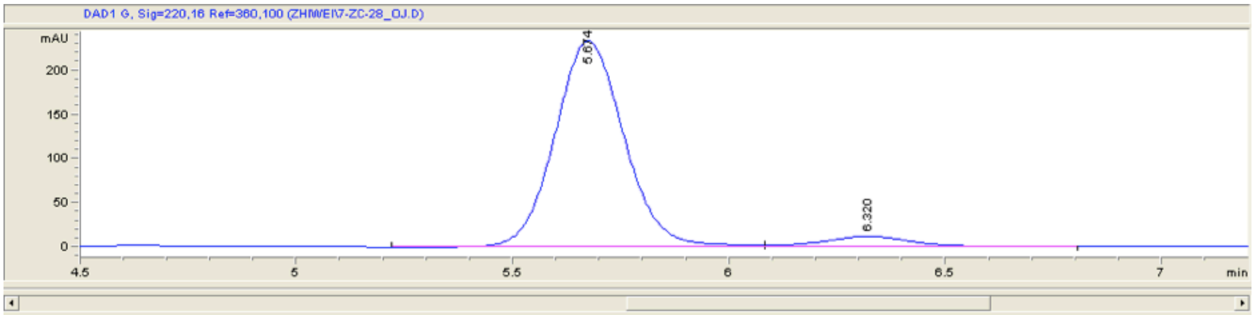

#	Time	Area	Height	Width	Area%	Symmetry
1	5.674	2593.9	232.8	0.174	93.782	0.892
2	6.32	172	11.7	0.2211	6.218	0.943

File Information	
LC-File	7-ZC-28_OJ.D
File Path	C:\CHEM32\1\DATA\ZHIWEI\
Date	01-Nov-16, 23:44:55

Supplementary Figure 145. SFC trace of (±)-2j

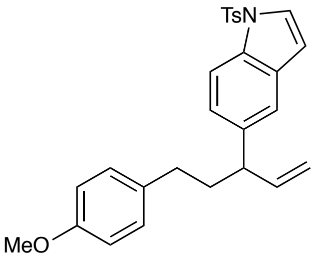

(±)-**2j**

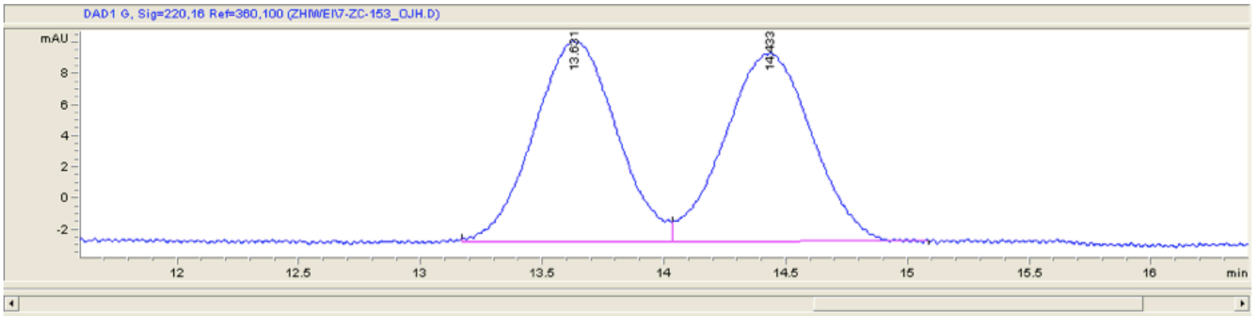

#	Time	Area	Height	Width	Area%	Symmetry
1	13.631	304.7	12.9	0.3582	50.074	0.936
2	14.433	303.8	12.1	0.3759	49.926	1.019

File Information	
LC-File	7-ZC-153_OJH.D
File Path	C:\CHEM32\1\DATA\ZHIWEI\
Date	30-Jan-17, 21:06:00

Supplementary Figure 146. SFC trace of (+)-2j

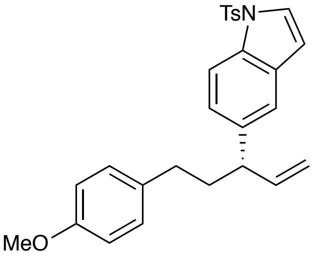

(+)-**2j**

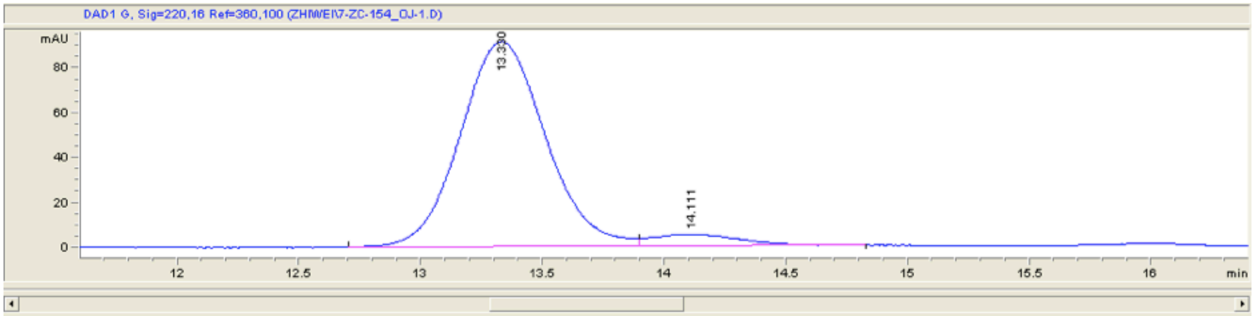

#	Time	Area	Height	Width	Area%	Symmetry
1	13.33	2230.2	91.5	0.3584	94.241	0.897
2	14.111	136.3	5.2	0.3175	5.759	0.702

File Information	
LC-File	7-ZC-154_OJ-1.D
File Path	C:\CHEM32\1\DATA\ZHIWEI\
Date	25-Jan-17, 12:23:44

Supplementary Figure 147. SFC trace of (±)-2k

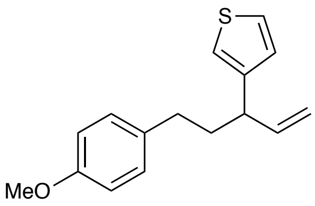

(±)-**2k**

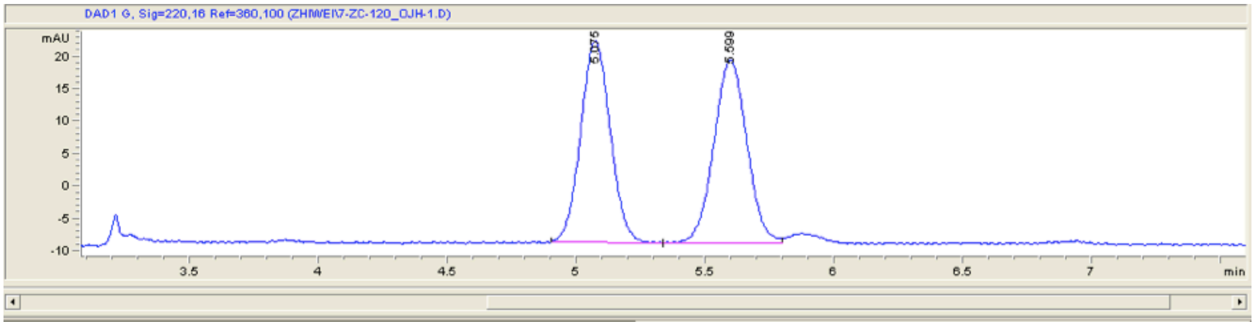

#	Time	Area	Height	Width	Area%	Symmetry
1	5.075	251.1	31.2	0.124	49.748	0.947
2	5.599	253.7	28.3	0.1407	50.252	0.968

File Information	
LC-File	7-ZC-120_OJH-1.D
File Path	C:\CHEM32\1\DATA\ZHIWEI\
Date	30-Jan-17, 19:26:06

* peak at 5.9 min is residual internal alkene that was not completely separated from the product

Supplementary Figure 148. SFC trace of (+)-2k

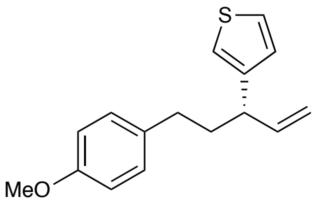

(+)-**2k**

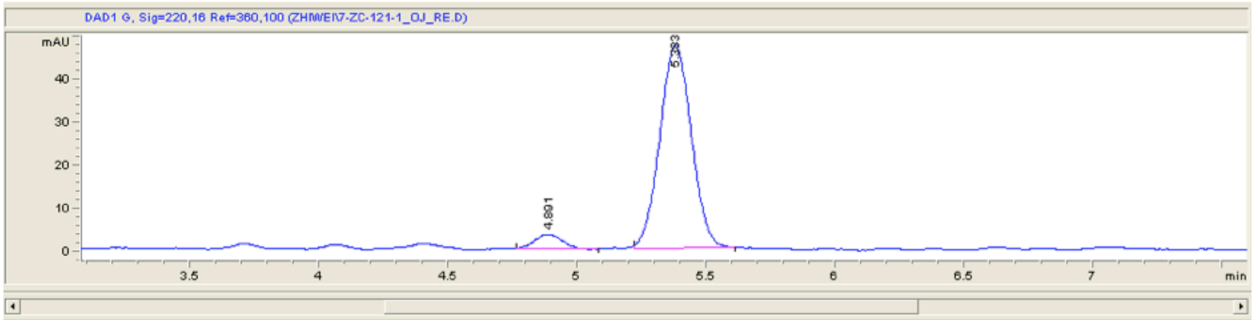

#	Time	Area	Height	Width	Area%	Symmetry
1	4.891	24.2	3.3	0.1095	5.633	1.025
2	5.383	405.1	47.4	0.1318	94.367	0.94

File Information	
LC-File	7-ZC-121-1_OJ_RE.D
File Path	C:\CHEM32\1\DATA\ZHIWEI\
Date	08-Jan-17, 16:41:29

Supplementary Figure 149. SFC trace of (±)-2l

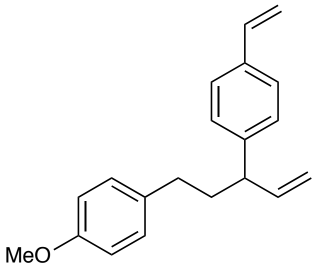

(±)-**2l**

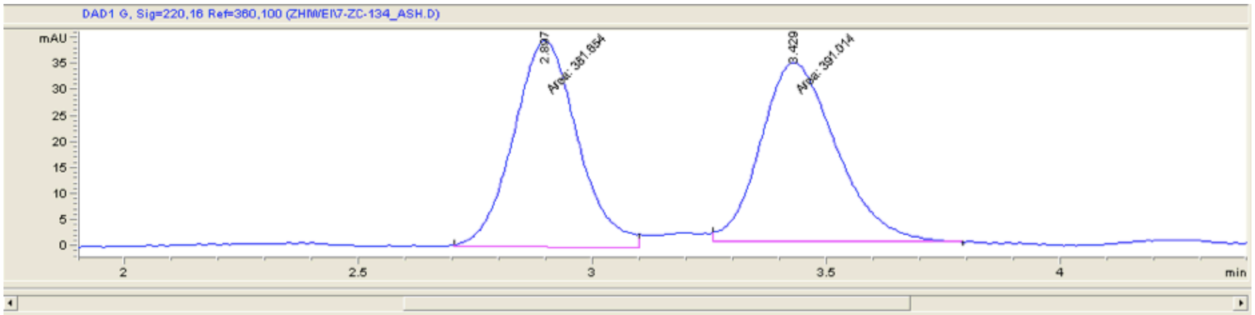

#	Time	Area	Height	Width	Area%	Symmetry
1	2.897	381.7	39.7	0.1603	49.394	0.892
2	3.429	391	34.5	0.1887	50.606	0.769

File Information

LC-File	7-ZC-134_ASH.D
File Path	C:\CHEM32\1\DATA\ZHIWEI\
Date	30-Jan-17, 21:49:06

Supplementary Figure 150. SFC trace of (+)-2l

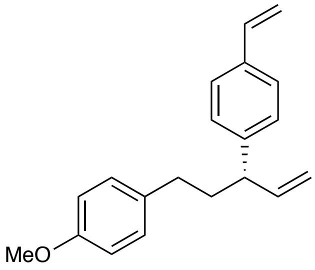

(+)-**2l**

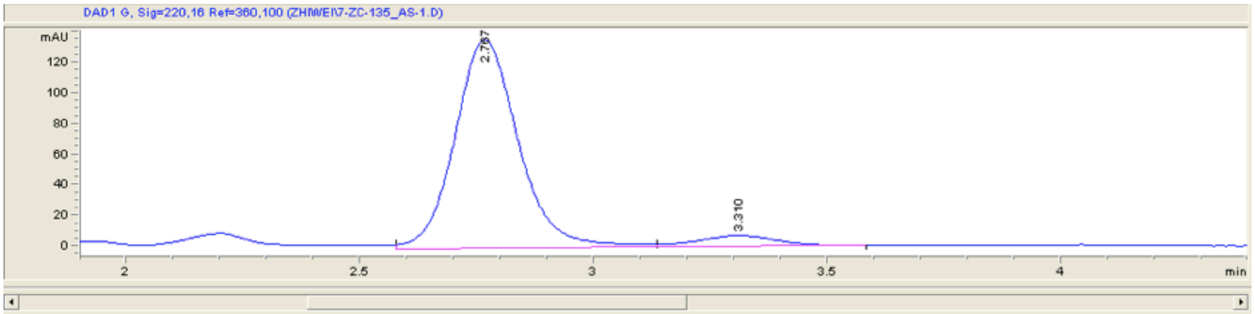

#	Time	Area	Height	Width	Area%	Symmetry
1	2.767	1304.8	136.4	0.1476	93.190	0.834
2	3.31	95.4	7.5	0.188	6.810	0.964

File Information

LC-File	7-ZC-135_AS-1.D
File Path	C:\CHEM32\1\DATA\ZHIWEI\
Date	14-Jan-17, 14:09:29

Supplementary Figure 151. SFC trace of (±)-2m

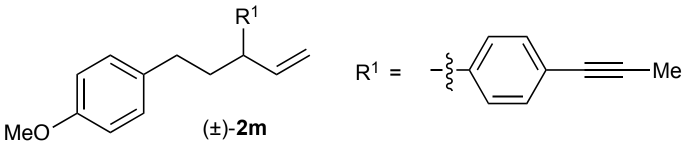

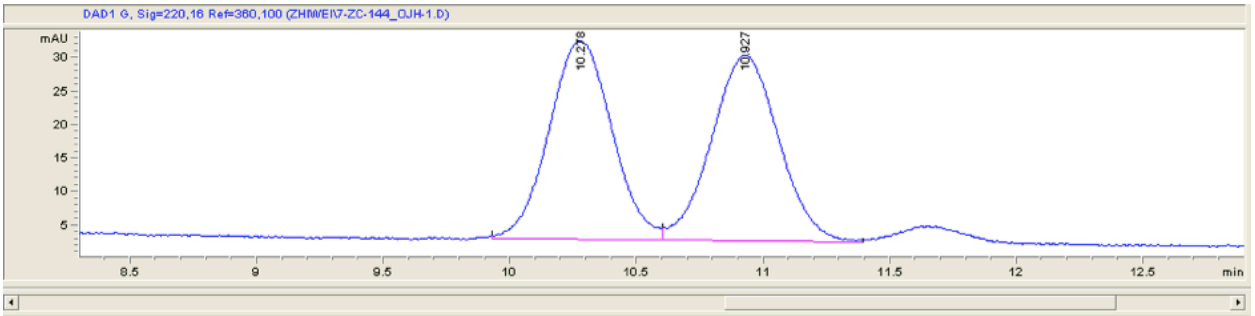

#	Time	Area	Height	Width	Area%	Symmetry
1	10.278	513	29.6	0.2744	49.899	0.96
2	10.927	515.1	27.7	0.2867	50.101	0.953

File Information	
LC-File	7-ZC-144_OJH-1.D
File Path	C:\CHEM32\1\DATA\ZHIWEI\
Date	30-Jan-17, 20:06:28

- peak at 11.7 min is residual internal alkene that was not completely separated from the product

Supplementary Figure 152. SFC trace of (+)-2m

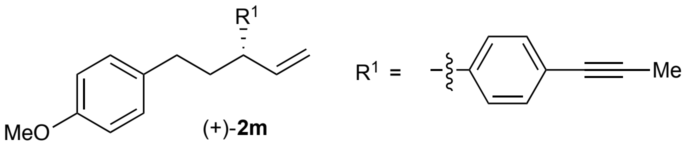

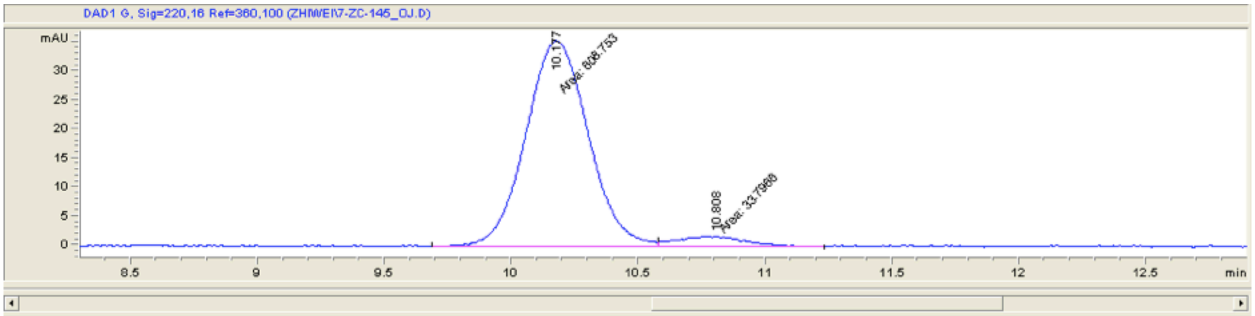

#	Time	Area	Height	Width	Area%	Symmetry
1	10.177	608.8	35.5	0.2858	94.740	0.94
2	10.808	33.8	1.8	0.3173	5.260	1.182

File Information	
LC-File	7-ZC-145_OJ.D
File Path	C:\CHEM32\1\DATA\ZHIWEI\
Date	16-Jan-17, 18:47:34

Supplementary Figure 153. SFC trace of (±)-2n

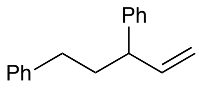

(±)-**2n**

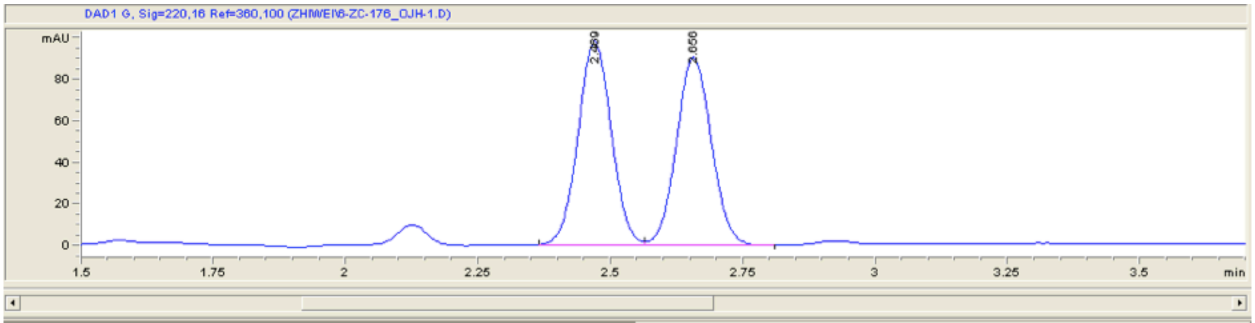

#	Time	Area	Height	Width	Area%	Symmetry
1	2.469	432.2	98.5	0.0682	50.969	0.955
2	2.656	415.7	90.7	0.0725	49.031	0.913

File Information

LC-File	6-ZC-176_OJH-1.D
File Path	C:\CHEM32\1\DATA\ZHIWEI\
Date	29-Jan-17, 21:55:38

Supplementary Figure 154. SFC trace of (+)-2n

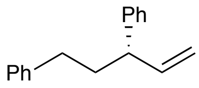

(+)-**2n**

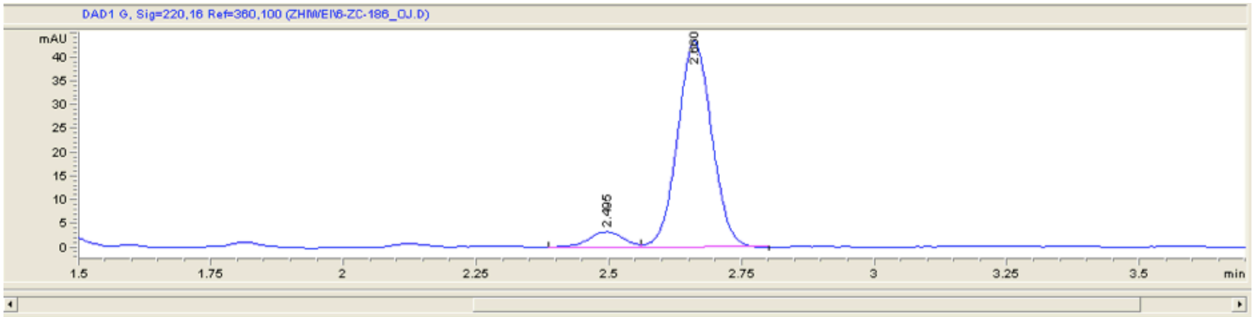

#	Time	Area	Height	Width	Area%	Symmetry
1	2.495	15.4	3.3	0.0719	7.338	1.057
2	2.66	194.6	43.3	0.0704	92.662	0.943

File Information

LC-File	6-ZC-186_OJ.D
File Path	C:\CHEM32\1\DATA\ZHIWEI\
Date	15-Oct-16, 14:10:02

Supplementary Figure 155. SFC trace of (±)-2o

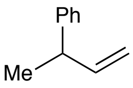

(±)-**2o**

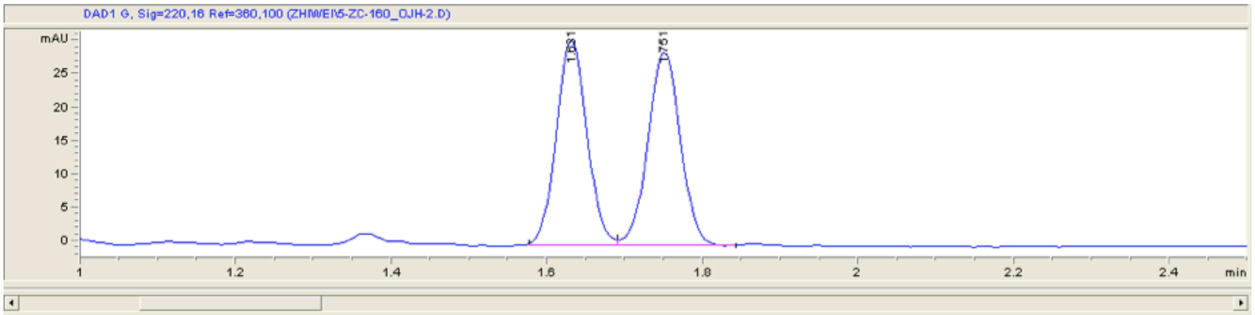

#	Time	Area	Height	Width	Area%	Symmetry
1	1.631	84.5	30.8	0.0441	49.909	0.926
2	1.751	84.8	29	0.0461	50.091	0.968

File Information	
LC-File	5-ZC-160_OJH-2.D
File Path	C:\CHEM32\1\DATA\ZHIWEI\
Date	10-Jun-16, 10:16:17

Supplementary Figure 156. SFC trace of (+)-2o

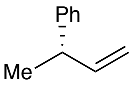

(+)-**2o**

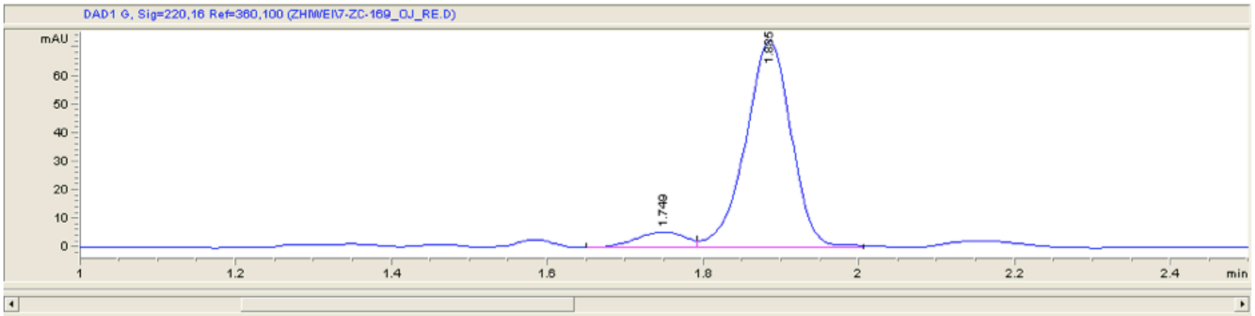

#	Time	Area	Height	Width	Area%	Symmetry
1	1.749	24	5.5	0.0693	7.533	1.271
2	1.885	295	72.2	0.0637	92.467	1.089

File Information	
LC-File	7-ZC-169_OJ_RE.D
File Path	C:\CHEM32\1\DATA\ZHIWEI\
Date	28-Jan-17, 21:45:00

Supplementary Figure 157. SFC trace of (±)-2p

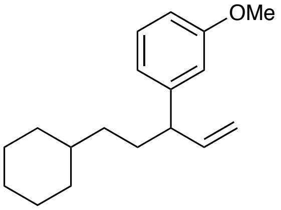

(±)-**2p**

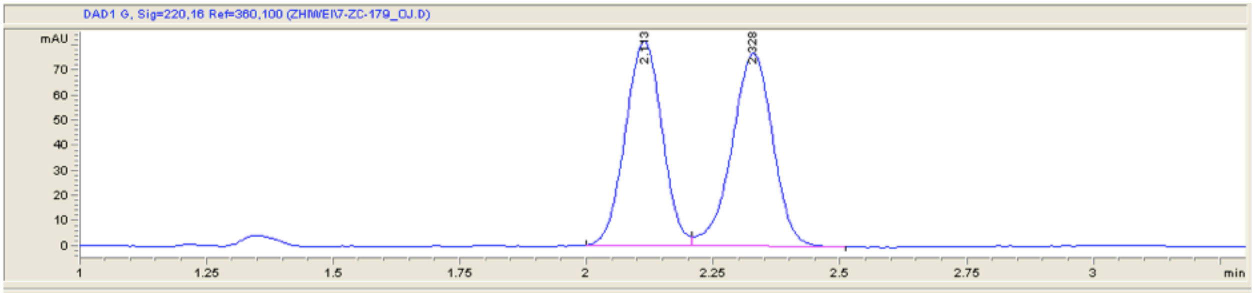

#	Time	Area	Height	Width	Area%	Symmetry
1	2.113	417.1	81.8	0.0805	49.027	1.013
2	2.328	433.7	77.3	0.0875	50.973	1.073

File Information

LC-File	7-ZC-179_OJ.D
File Path	C:\CHEM32\1\DATA\ZHIWEI\
Date	09-Feb-17, 09:52:21

Supplementary Figure 158. SFC trace of (+)-2p

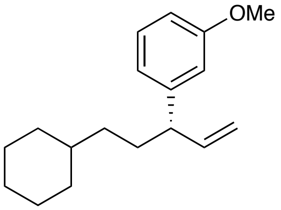

(+)-**2p**

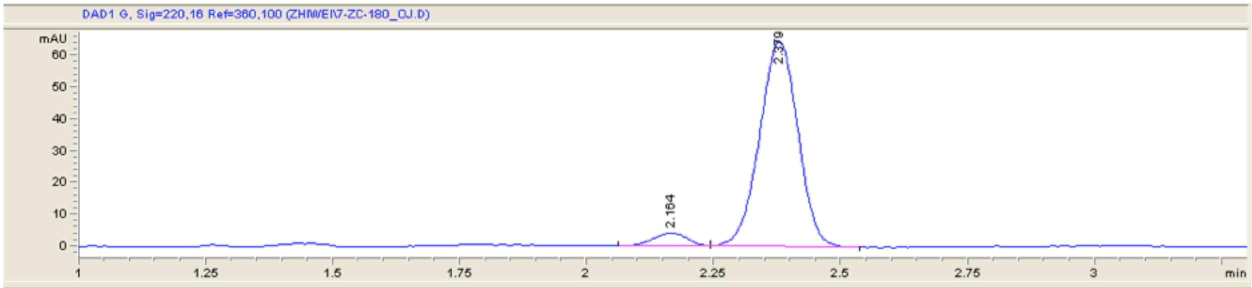

#	Time	Area	Height	Width	Area%	Symmetry
1	2.164	19.8	4.2	0.0757	5.503	1.041
2	2.379	340.4	64.7	0.0824	94.497	1.028

File Information

LC-File	7-ZC-180_OJ.D
File Path	C:\CHEM32\1\DATA\ZHIWEI\
Date	09-Feb-17, 10:09:23

Supplementary Figure 159. SFC trace of (±)-2q

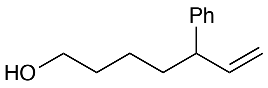

(±)-**2q**

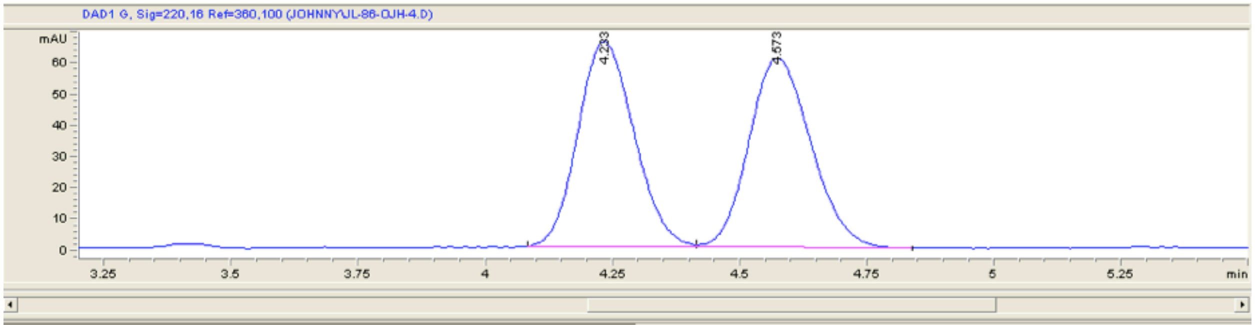

#	Time	Area	Height	Width	Area%	Symmetry
1	4.233	510.6	66	0.1223	49.692	0.839
2	4.573	517	60.8	0.1313	50.308	0.816

File Information	
LC-File	JL-86-OJH-4.D
File Path	C:\CHEM32\1\DATA\JOHNNY\
Date	18-Feb-17, 22:39:06

Supplementary Figure 160. SFC trace of (+)-2q

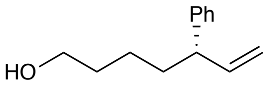

(+)-**2q**

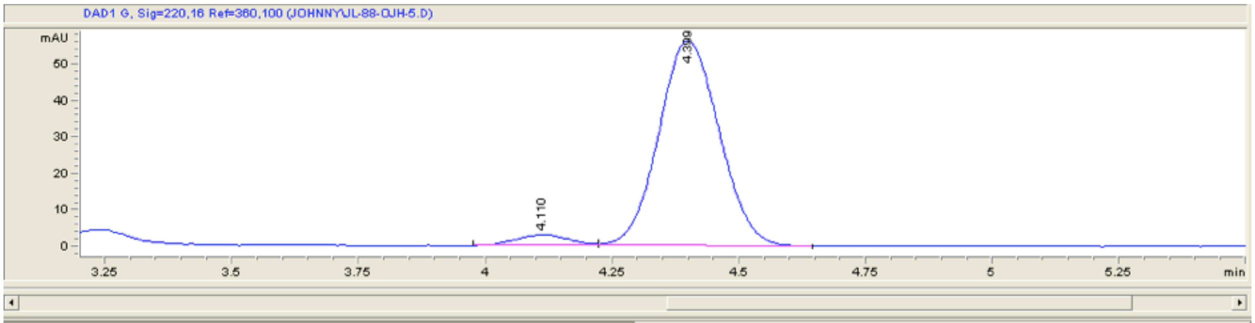

#	Time	Area	Height	Width	Area%	Symmetry
1	4.11	22	3	0.0997	4.490	0.959
2	4.399	467.7	56.3	0.1309	95.510	0.909

File Information	
LC-File	JL-88-OJH-5.D
File Path	C:\CHEM32\1\DATA\JOHNNY\
Date	18-Feb-17, 23:01:46

Supplementary Figure 159. SFC trace of (±)-2q

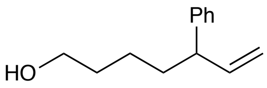

(±)-**2q**

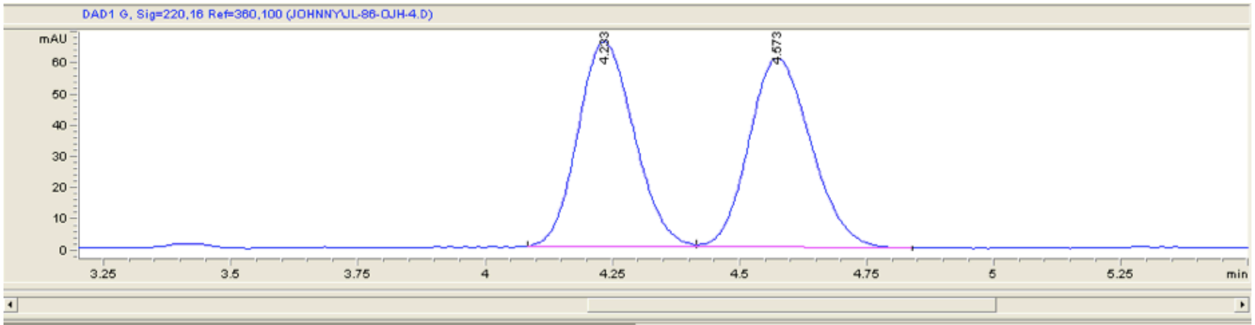

#	Time	Area	Height	Width	Area%	Symmetry
1	4.233	510.6	66	0.1223	49.692	0.839
2	4.573	517	60.8	0.1313	50.308	0.816

File Information	
LC-File	JL-86-OJH-4.D
File Path	C:\CHEM32\1\DATA\JOHNNY\
Date	18-Feb-17, 22:39:06

Supplementary Figure 161. SFC trace of (+)-2q from (+)-2r

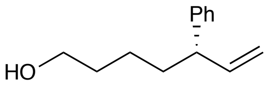

(+)-**2q** from (+)-**2r**

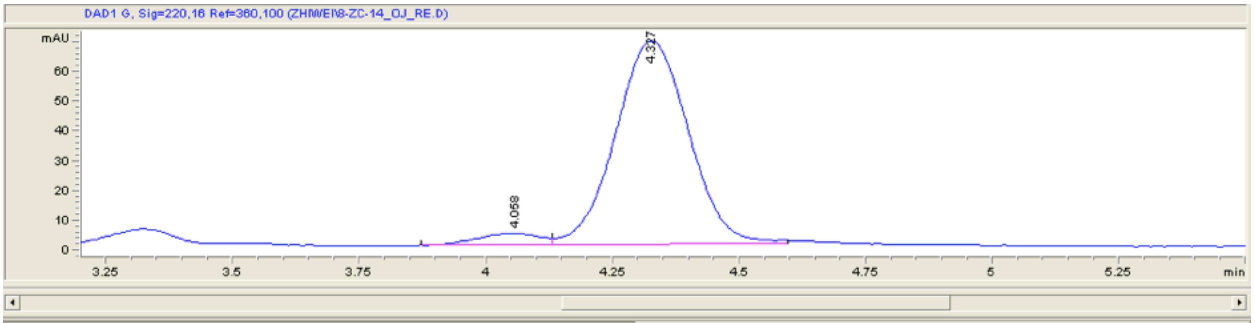

#	Time	Area	Height	Width	Area%	Symmetry
1	4.058	33.9	4	0.1063	4.699	1.51
2	4.327	687.8	68.5	0.1572	95.301	0.948

File Information	
LC-File	8-ZC-14_OJ_RE.D
File Path	C:\CHEM32\1\DATA\ZHIWEI\
Date	27-Feb-17, 13:26:04

Supplementary Figure 162. SFC trace of (±)-2r

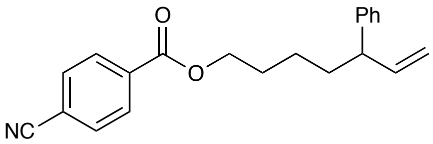

(±)-**2s**

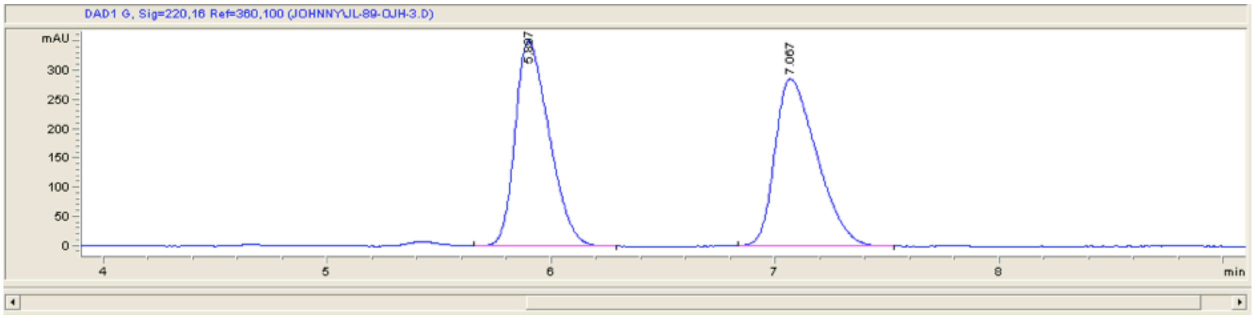

#	Time	Area	Height	Width	Area%	Symmetry
1	5.897	3726.3	351.3	0.1647	49.866	0.627
2	7.067	3746.3	286.1	0.2028	50.134	0.566

File Information

LC-File	JL-89-OJH-3.D
File Path	C:\CHEM32\1\DATA\JOHNNY\
Date	18-Feb-17, 22:49:13

Supplementary Figure 163. SFC trace of (+)-2s

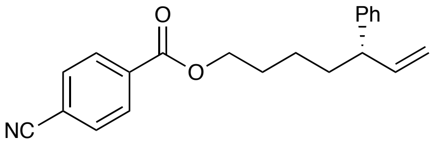

(+)-**2s**

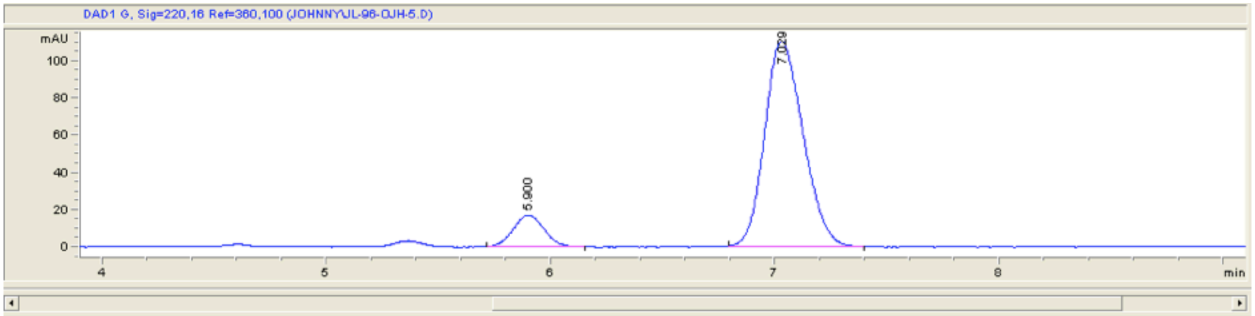

#	Time	Area	Height	Width	Area%	Symmetry
1	5.9	166.3	17.2	0.1479	11.173	0.947
2	7.029	1322.3	110.6	0.1845	88.827	0.758

File Information

LC-File	JL-96-OJH-5.D
File Path	C:\CHEM32\1\DATA\JOHNNY\
Date	18-Feb-17, 12:59:24

Supplementary Figure 164. SFC trace of (±)-2ab

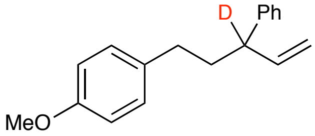

(±)-**2ab**

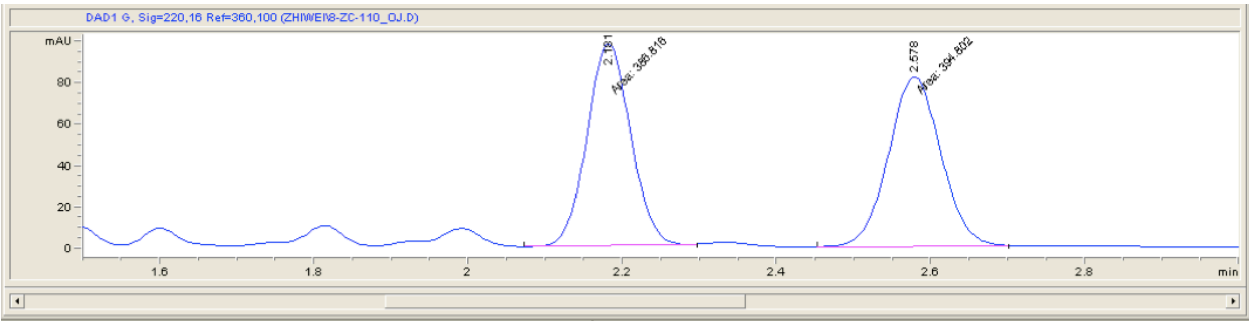

#	Time	Area	Height	Width	Area%	Symmetry
1	2.181	386.8	97.8	0.0659	49.489	0.929
2	2.578	394.8	82.4	0.0798	50.511	0.952

File Information

LC-File	8-ZC-110_OJ.D
File Path	C:\CHEM32\1\DATA\ZHIWEI\
Date	14-May-17, 15:24:47

Supplementary Figure 165. SFC trace of (+)-2ab

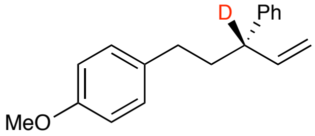

(+)-**2ab**

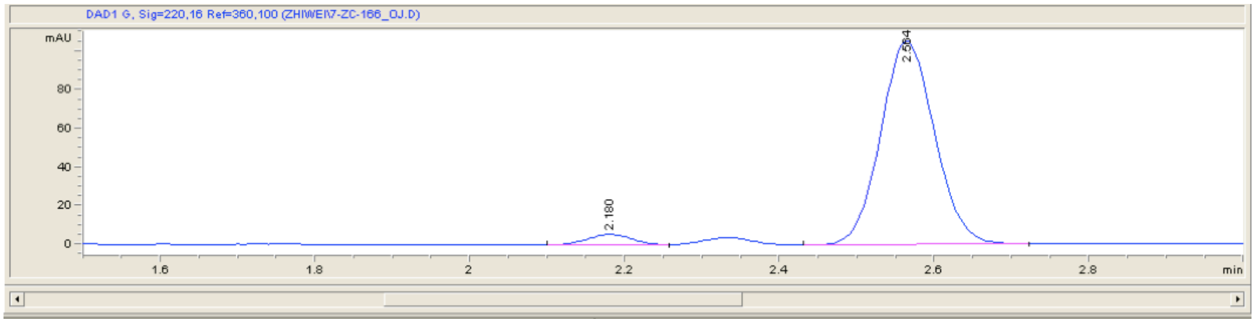

#	Time	Area	Height	Width	Area%	Symmetry
1	2.18	21.9	5.4	0.0621	4.103	0.95
2	2.564	512.2	105.5	0.0757	95.897	0.902

File Information

LC-File	7-ZC-166_OJ.D
File Path	C:\CHEM32\1\DATA\ZHIWEI\
Date	14-May-17, 13:59:54

Supplementary Figure 166. SFC trace of (±)-2ac

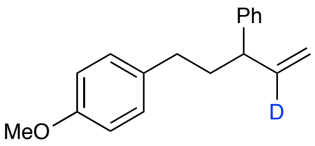

(±)-**2ac**

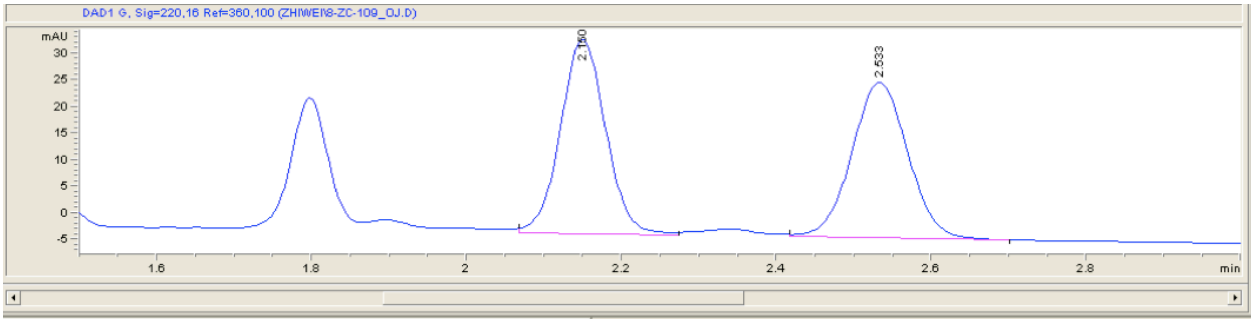

#	Time	Area	Height	Width	Area%	Symmetry
1	2.15	148.8	36.7	0.0634	50.130	0.926
2	2.533	148.1	29.3	0.0779	49.870	0.955

File Information

LC-File	8-ZC-109_OJ.D
File Path	C:\CHEM32\1\DATA\ZHIWEI\
Date	14-May-17, 13:25:43

* peak at 1.8 min is residual internal alkene that was not completely separated from the product

Supplementary Figure 167. SFC trace of (+)-2ac

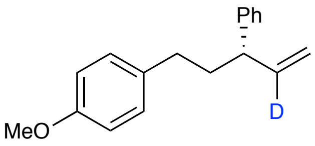

(+)-**2ac**

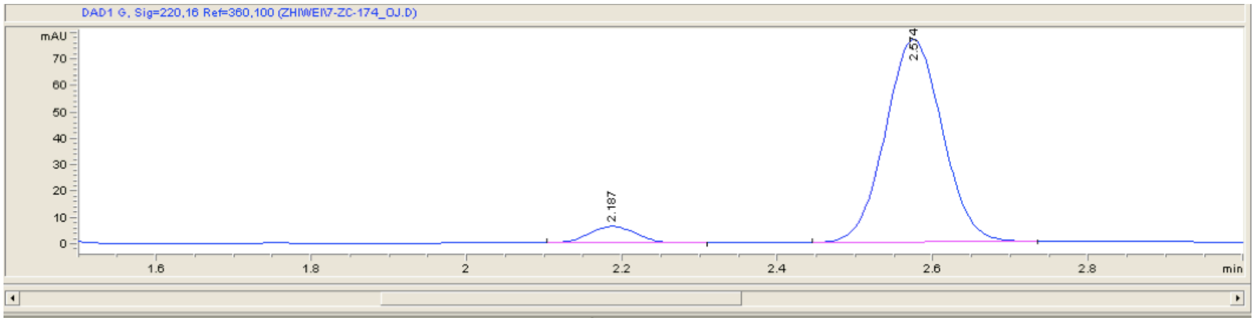

#	Time	Area	Height	Width	Area%	Symmetry
1	2.187	24.9	6.3	0.0643	5.943	0.983
2	2.574	394.1	77	0.0798	94.057	0.94

File Information

LC-File	7-ZC-174_OJ.D
File Path	C:\CHEM32\1\DATA\ZHIWEI\
Date	14-May-17, 14:38:49

Supplementary References

1. Gobé, V. & Guinchard, X. Stereoselective Synthesis of Chiral Polycyclic Indolic Architectures through Pd^0-Catalyzed Tandem Deprotection/Cyclization of Tetrahydro-β-carbolines on Allenes. *Chem. Eur. J.* **21**, 8511–8520 (2015).
2. Clavier, H., Le Jeune, K., de Riggi, I., Tenaglia, A. & Buono, G. Highly Selective Cobalt-Mediated [6 + 2] Cycloaddition of Cycloheptatriene and Allenes. *Org. Lett*. **13**, 308–311 (2011).
3. For **5b**, see Larraufie, M.-H., Pellet, R., Fensterbank, L., Goddard, J.-P., Lacôte, E., Malacria, M. & Ollivier, C. Visible-Light-Induced Photoreductive Generation of Radicals from Epoxides and Aziridines. *Angew. Chem. Int. Ed.* **50**, 4463–4466 (2011).
4. For **5c**, see Zhang, D., Wu, L.-Z., Zhou, L., Han, X., Yang, Q.-Z., Zhang, L.-P. & Tung, C.-H. Photocatalytic Hydrogen Production from Hantzsch 1,4-Dihydropyridines by Platinum(II) Terpyridyl Complexes in Homogeneous Solution. *J. Am. Chem. Soc.* **126**, 3440–3441 (2004).
5. Lee, Y., Li, B. & Hoveyda, A. H. Stereogenic-at-Metal Zn- and Al-Based N-Heterocyclic Carbene (NHC) Complexes as Bifunctional Catalysts in Cu-Free Enantioselective Allylic Alkylations. *J. Am. Chem. Soc.* **131**, 11625–11633 (2009).
6. How, R. C., Hembre, R., Ponasik, J. A., Tolleson, G. S. & Clarke, M. L. A modular family of phosphine-phosphoramidite ligands and their hydroformylation catalysts: steric tuning impacts upon the coordination geometry of trigonal bipyramidal complexes of type $[Rh(H)(CO)_2(P^\wedge P^*)]$. *Catal. Sci. Technol.* **6**, 118–124 (2016).
7. Gokel, G. W. & Ugi, I. K. Preparation and resolution of N,N-dimethyl-o-ferrocenylethylamine. An advanced organic experiment. *J. Chem. Educ*. **49**, 294–296 (1972).
8. Wright, J., Frambes, L. & Reeves, P. A simple route to chiral ferrocenyl alcohols. *J. Organomet*. *Chem*. **476**, 215–217 (1994).
9. Wu, Y., Lu, C., Shan, W. & Li, X. A practical process to chiral ferrocenyl alcohols via asymmetric transfer hydrogenation catalyzed with a PEG-bound Ru catalyst in water and its application in preparing Ugi's amine. *Tetrahedron: Asymmetry* **20**, 584–587 (2009).
10. Gourdet, B. & Lam, H. W. Catalytic Asymmetric Dihydroxylation of Enamides and Application to the Total Synthesis of (+)-Tanikolide. *Angew. Chem. Int. Ed.* **49**, 8733–8737 (2010).

11. Lee, H. W., Lee, L. N., Chan, A. S. C. & Kwong, F. Y. Microwave-Assisted Rhodium-Complex-Catalyzed Cascade Decarbonylation and Asymmetric Pauson–Khand-Type Cyclizations. *Eur. J. Org. Chem.* **2008**, 3403–3406 (2008).
12. Jui, N. T., Lee, E. C. Y. & MacMillan, D. W. C. Enantioselective Organo-SOMO Cascade Cycloadditions: A Rapid Approach to Molecular Complexity from Simple Aldehydes and Olefins. *J. Am. Chem. Soc.* **132**, 10015–10017 (2010).
13. Lee, H. W., Chan, A. S. C. & Kwong, F. Y. Formate as a CO surrogate for cascade processes: Rh-catalyzed cooperative decarbonylation and asymmetric Pauson–Khand-type cyclization reactions. *Chem. Commun.* 2633–2635 (2007).
